# Asymmetric Synthesis of Tertiary Alcohols and Thiols via Nonstabilized Tertiary α‐Oxy‐ and α‐Thio‐Substituted Organolithium Species

**DOI:** 10.1002/anie.201706722

**Published:** 2017-08-07

**Authors:** Alexander P. Pulis, Ana Varela, Cinzia Citti, Pradip Songara, Daniele Leonori, Varinder K. Aggarwal

**Affiliations:** ^1^ School of Chemistry University of Bristol Cantock's Close Bristol BS8 1TS UK; ^2^ School of Chemistry University of Manchester Oxford Road Manchester M13 9PL UK

**Keywords:** chiral organolithium species, IR spectroscopy, tertiary alcohols, tertiary thiols

## Abstract

Nonstabilized α‐O‐substituted tertiary organolithium species are difficult to generate, and the α‐S‐substituted analogues are configurationally unstable. We now report that they can both be generated easily and trapped with a range of electrophiles with high enantioselectivity, providing ready access to a range of enantioenriched tertiary alcohols and thiols. The configurational stability of the α‐S‐organolithium species was enhanced by using a less coordinating solvent and short reaction times.

Chiral α‐heteroatom (O, N, and S)‐substituted organolithium compounds are a versatile class of nucleophiles that are useful in the asymmetric synthesis of chiral alcohols, amines, and thiols.^1^ Although the use of secondary and mesomerically stabilized (e.g., benzylic and allylic) tertiary α‐O‐ and α‐S‐substituted organolithium reagents in synthesis is well established,1c–1e the use of non‐mesomerically stabilized (i.e., dialkyl‐substituted) tertiary reagents is not. This discontinuity is due to contrasting problematic features governing α‐O‐ and α‐S‐substituted organolithium species. Nonstabilized tertiary α‐O‐organolithium compounds are configurationally stable but are difficult to generate owing to their reduced kinetic acidity (Scheme 1 B);1e, 2 tertiary α‐S‐organolithium species are easily formed but are not configurationally stable (Scheme 1 A). To apply α‐O/S‐substituted organolithium compounds in asymmetric synthesis, both ease of generation and configurational stability are essential requirements.1a–1d, 3

**Scheme 1:**
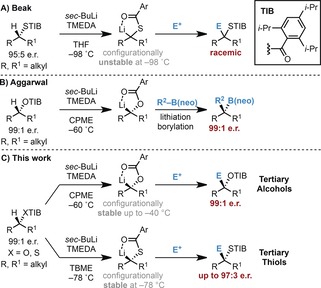
A, B) Previous studies regarding nonstabilized tertiary α‐S‐ and α‐O‐substituted organolithium compounds. C) This work, reporting their straightforward generation, configurational stability, and electrophilic trapping. CPME=cyclopentyl methyl ether, TBME=*tert*‐butyl methyl ether, TMEDA=tetramethylethylenediamine.

Through variation of the directing group, base, solvent, and additives we discovered reaction conditions enabling the stereospecific deprotonation of secondary dialkyl benzoates (TIB esters)^4^ and demonstrated their configurational stability in lithiation–borylation reactions.^5^ Herein, we report the broad applicability of these novel enantioenriched nucleophiles in reactions with a broad range of electrophiles. In addition, we have discovered reaction conditions for the generation of enantioenriched, tertiary, nonstabilized α‐S‐organolithium compounds and report their subsequent trapping with electrophiles in high enantioselectivity (Scheme 1 C).

Boronic esters represent a niche class of electrophiles,^6^ and therefore, we initially embarked on a study of the trapping of tertiary α‐O‐substituted organolithium species, which were generated by lithiation of the enantioenriched TIB esters **1 a**–**1 e**, with a range of electrophile classes (Scheme 2). Upon exposure of a variety of enantioenriched benzoates to *sec*‐BuLi and TMEDA in CPME at −60 °C, the corresponding organolithium species Li‐**1 a**–**1 e** were generated. Pleasingly, Li‐**1 a**–**1 e** were successfully trapped with a range of electrophiles, including methyl chloroformate (**2 aa**), benzoyl chloride (**2 ab**), isocyanates (**2 ac** and **2 ad**), aldehydes (**2 ae** and **2 af**), and trialkyl tin chlorides (**2 ag**, **2 ah**, **2 ba**–**2 ea**). Reactions with aldehydes gave mixtures of diastereomers (see **2 ae** and **2 af**) but in the case of PhCHO, high diastereoselectivity was observed (11:1 d.r.). In all cases, the desired tertiary alcohol derivatives **2** were obtained in good to high yields and, importantly, with universally complete enantioselectivity and retention of configuration, as determined by X‐ray crystallographic analysis of **2 ad** and **2 af** (see the Supporting Information). In addition, the TIB group could be easily removed upon reduction with LiAlH_4_ to give the tertiary alcohol **3**.

**Scheme 2:**
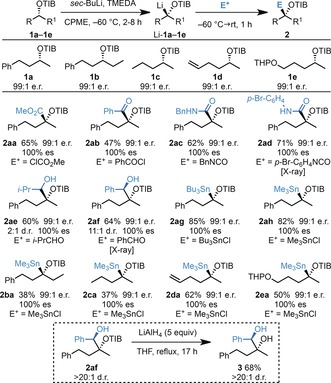
Scope of the electrophilic trapping of nonstabilized, tertiary α‐O‐substituted organolithium species.

Having demonstrated the scope of the electrophilic trapping of nonstabilized, tertiary α‐O‐substituted organolithium intermediates, we conducted studies to evaluate whether stannanes **2 ag** and **2 ah** could serve as bench‐stable organolithium precursors by tin–lithium exchange (Scheme 3).^7^ However, treatment of the tributyltin derivative **2 ag** with *n*‐BuLi with or without TMEDA followed by quenching with CH_3_OD only gave [D]‐**1 a** with poor conversion, albeit with excellent stereoselectivity (with retention of configuration, see entries 1 and 2). In contrast, treatment of the less hindered trimethyltin derivative **2 ah** with *n*‐BuLi/TMEDA was much more successful and yielded [D]‐**1 a** in excellent yield and with complete stereoselectivity and retention of configuration (entries 3 and 4), indicating that **2 ah** could indeed serve as a useful precursor to the corresponding organolithium species.^8^

**Scheme 3:**
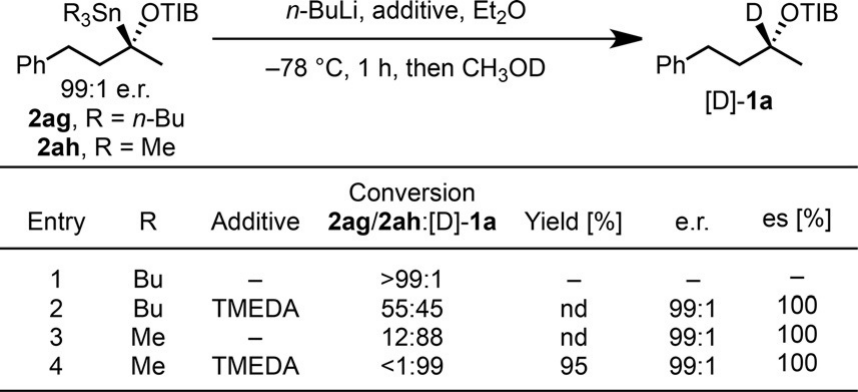
Investigation of the tin–lithium exchange with the tertiary α‐O‐substituted organostannanes **2 ag** and **2 ah**.

An understanding of the configurational stability of chiral organolithium species is crucial for exploiting their use in synthesis.^3^, ^9^ Interestingly, the tertiary α‐O‐substituted organolithium species Li‐**1** were found to be chemically and configurationally stable below −40 °C but at higher temperatures, decomposition rather than racemization occurred. Similar observations have been made with secondary α‐O‐substituted organolithium TIB esters whilst the corresponding carbamates are slightly more stable and only decompose above −20 °C.2b, 7c, 10 All of these nonstabilized α‐O‐organolithium species decompose before they racemize.

Having demonstrated the broad applicability of tertiary nonstabilized chiral α‐O‐organolithium species, we then embarked on a study of the more challenging sulfur analogues.1c, 11 Pioneering work by Beak had revealed that while the α‐deprotonation of dialkyl‐substituted tertiary thiobenzoates (such as **4**) was facile at low temperature, they were configurationally unstable even at −98 °C in THF (Scheme 1 A).^12^

Only tertiary or hindered, branched α‐S‐substituted organolithium compounds (e.g., **5 a**–**5 c**) have been reported to be configurationally stable;^13^ all others are unstable (Scheme 4 A).^14^ This can be explained based on the mechanism of racemization of α‐S‐substituted organolithium compounds, which involves solvent separation of the ion pair, rate‐determining rotation of the hyperconjugated C−S bond, and recombination of the ion pair (Scheme 4 B).^15^ Thus configurational stability in α‐S‐organolithium compounds is only observed with hindered substrates where there is a high barrier to C−S bond rotation. Given this observation, we proposed that nonstabilized, tertiary α‐S‐substituted organolithium species derived from thiobenzoates **4** should be configurationally stable and therefore re‐examined the conditions for lithiation.

**Scheme 4:**
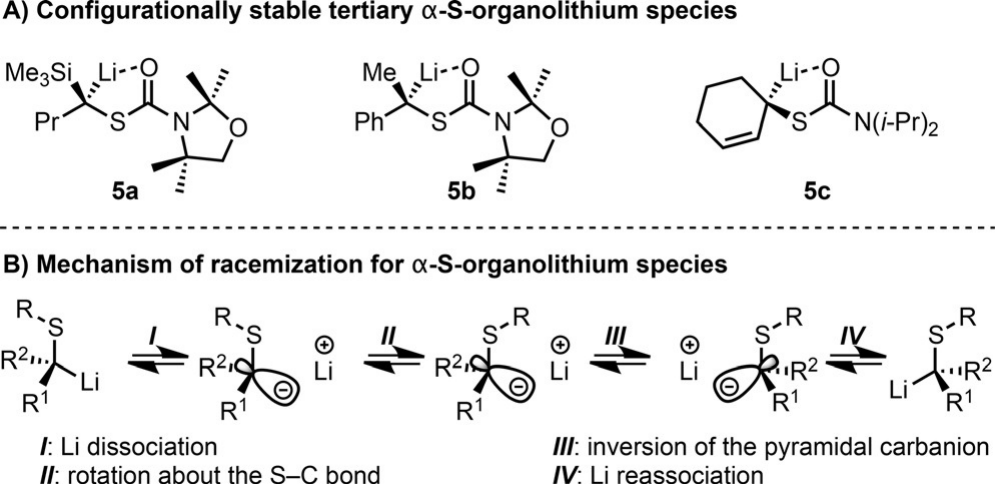
Previously reported configurationally stable tertiary α‐S‐substituted organolithium compounds and mechanism of racemization.^15^.

Enantiomerically enriched thiobenzoates **4 a**, which were synthesized from the corresponding secondary alcohols and 2,4,6‐triisopropylthiobenzoic acid by a Mitsunobu reaction, were lithiated under a variety of reaction conditions and subsequently reacted with CH_3_OD (Scheme 5). Using THF as the solvent gave the product [D]‐**4 a** as a racemate (entry 1), thus confirming Beak's observations. Gratifyingly, the use of Et_2_O as the solvent generated [D]‐**4 a** in 90:10 e.r. and complete conversion (entry 2). TMEDA was crucial to facilitate deprotonation as in its absence, [D]‐**4 a** was formed only in low yield (entry 3).^16^ TBME was found to be the most suitable solvent (entry 4), and even a very short deprotonation time (5 min) was sufficient to give the desired product [D]‐**4 a** with 100 % conversion and 97:3 e.r. with retention of configuration (entry 5). These results show that the racemization of the tertiary α‐S‐substituted organolithium species can be minimized when less coordinating solvents and short reaction times are employed, reaction conditions that maintain a tight ion pair.

**Scheme 5:**
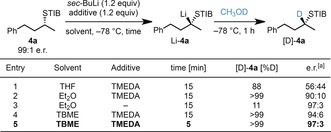
Optimization of the lithiation reaction conditions for the generation of nonstabilized, tertiary α‐S‐substituted organolithium species. [a] Determined on the crude reaction mixture by HPLC analysis on a chiral stationary phase.

Having identified the most suitable reaction conditions for the α‐deprotonation of STIB ester **4** and trapping with CH_3_OD, we evaluated the scope of electrophiles that could be employed. The same range of electrophiles that were compatible with the lithiated OTIB esters Li‐**1 a**–**1 e** were also successful in trapping nonstabilized, tertiary α‐S‐substituted organolithium species derived from **4 a**–**4 e**, and gave the tertiary thiol derivatives **6** in good yield and very high enantioselectivity in all cases with the exception of Si‐ and Sn‐based electrophiles (**6 ae**–**ag**, Scheme 6; see below). In the case of ClSnMe_3_ (**6 ag**), the predominant enantiomer arose from retentive addition to the organolithium (S_E_2 ret), whereas for ClSnBu_3_ (**6 af**), inversion was observed (S_E_2 inv).^17^

**Scheme 6:**
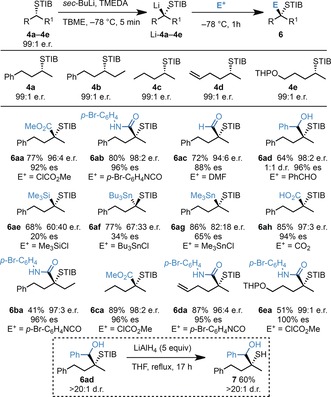
Scope of the electrophilic trapping of nonstabilized, tertiary α‐S‐substituted organolithium species. THP=tetrahydropyranyl.

We extended the method further by preparing the STIB cholesterol derivative **8**, which upon exposure to the optimized reaction conditions gave **9** (ClCO_2_Me quench) in excellent yield and as a single diastereoisomer (Scheme 7).^18^ This example highlights two key features in the generation of nonstabilized, tertiary α‐S‐substituted organolithium intermediates under our conditions. 1) Although the Li atom adopts an equatorial position in Li‐**8**, an orientation that according to Beak^12^ and Reich^19^ will favor epimerization by forming the more stable configuration with an axially positioned lithium (where the bulky TIB group is placed at an equatorial position), epimerization was not observed, underscoring the remarkable configurational stability of nonstabilized tertiary α‐S‐substituted organolithium species generated under our conditions. 2) The kinetic acidity of the α‐S‐proton in STIB esters **4** and **8** is remarkably high and outcompetes the lithiation of the allylic position.

**Scheme 7:**
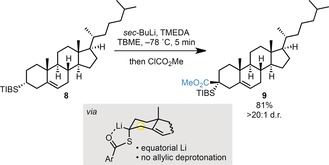
Electrophilic trapping of cholesterol‐derived nonstabilized, tertiary α‐S‐substituted organolithium species.

To place these newly reported nonstabilized, enantioenriched tertiary α‐S‐substituted organolithium species on firmer foundations, we determined the thermodynamic parameters, Δ*H* ^≠^ and Δ*S* ^≠^, of racemization.^20^ Initial studies by Hoffmann and co‐workers identified values of Δ*G* ^≠^_*rac*(−78 °C)_=+15 kcal mol^−1^ and Δ*G* ^≠^_*rac*(−90 °C)_=+13 kcal mol^−1^ for the racemization of **10 b** and **11 b**, respectively (Scheme 8 A).14a,14b,14d As previous studies were conducted on the *S*‐Ph and *S*‐duryl substrates **10** and **11**, we performed kinetic studies on model compound Li‐**4 a**. Thus, lithiation of STIB ester **4 a**, followed by equilibration at −78, −70, −65, or −60 °C, followed by cooling to −78 °C, and quenching with *p*‐Br‐C_6_H_4_‐NCO gave carbamate **6 ab** with different levels of enantioenrichment (see the Supporting Information). The erosion of e.r. observed in these experiments gave rise to an Eyring plot from which the parameters Δ*H* ^≠^ and Δ*S* ^≠^ were determined to be +13 kcal mol^−1^ and +14 cal mol^−1^, respectively^21^ (Scheme 8 B). This equates to a Δ*G* ^≠^_*rac*(‐78 °C)_ value of +10 kcal mol^−1^, which is in line with Hoffmann's results. Aside from providing the thermodynamic parameters for racemization, these studies also showed that racemization of Li‐**4** occurred at −78 °C over an extended period of time, which is in contrast to the behavior of oxygen analogues Li‐**1**, which do not racemize.

**Scheme 8:**
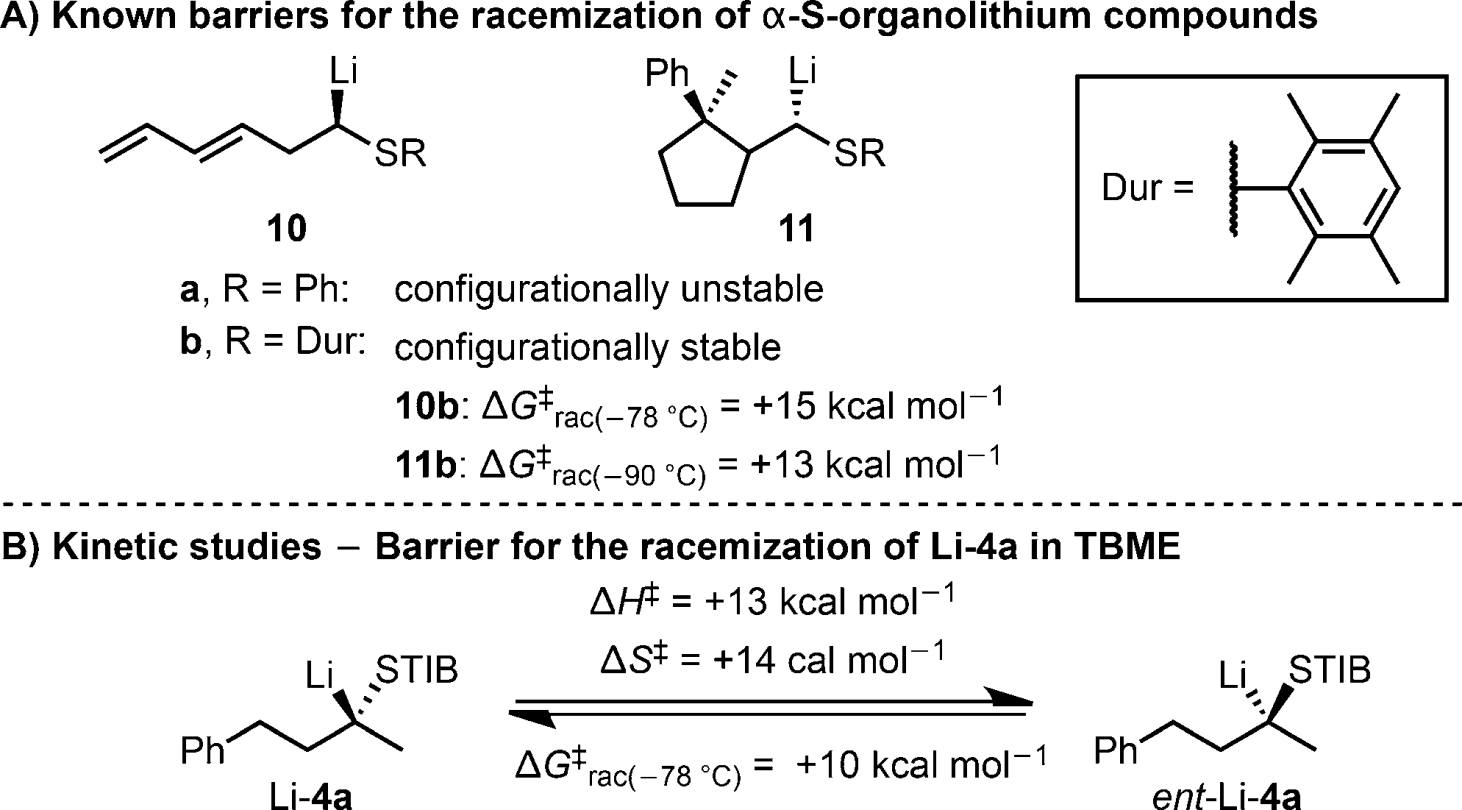
Barriers of racemization of known α‐S‐substituted organolithium species (A) and dialkyl‐substituted tertiary α‐S‐organolithium Li‐**4 a** (B).

Finally, we decided to investigate the factors that are responsible for the low selectivity observed in the reaction of nonstabilized, tertiary α‐S‐substituted organolithium species with Si‐ and Sn‐based electrophiles (Scheme 6, **6 ae**–**ag**). We speculated that a slow electrophilic quench might have resulted in partial racemization of Li‐**4** and therefore monitored the reaction by in situ IR spectroscopy (Scheme 9).^22^, ^23^ With the test electrophile ClCO_2_Me, where high e.r. values were observed, the in situ IR studies revealed that both lithiation of **4 a** and subsequent electrophilic quenching were extremely rapid at −78 °C.

**Scheme 9:**
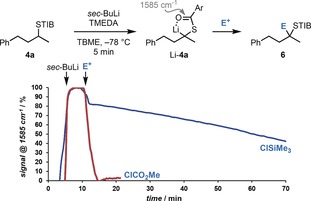
In situ IR spectroscopy studies of the electrophilic quenching of Li‐**4 a** with ClSiMe_3_ and ClCO_2_Me.

In the case of Me_3_SiCl, the electrophilic trapping was slow with only 50 % of the organolithium compound quenched after 1 h, thus leaving time for competing racemization.^24^ However, in the case of the Bu_3_SnCl and Me_3_SnCl electrophiles (**6 af** and **6 ag**), where poor e.r. values were observed, the IR profile revealed that quenching of Li‐**4 a** was rapid, as in the case of ClCO_2_Me. This excluded racemization of Li‐**4 a** as the sole cause of the poor e.r. observed. These unusual findings can be rationalized on the basis of competing retentive (S_E_2 ret) and invertive (S_E_2 inv) electrophilic substitution pathways in the quenching of Li‐**4 a** with tin electrophiles,^25^ where the more hindered Bu_3_SnCl electrophile favors the invertive pathway.

In conclusion, we have found that difficult‐to‐generate, nonstabilized, tertiary, α‐O‐substituted lithiated secondary dialkyl benzoates (OTIB esters) and previously regarded as configurationally unstable α‐S‐substituted lithiated secondary dialkyl thiobenzoates (STIB esters) can be generated, and are configurationally stable. Key to success in both cases was the use of mildly coordinating solvents together with TMEDA to enable deprotonation and, in the case of the α‐S‐organolithium species, short reaction times. The subsequent trapping of these rare, nonstabilized tertiary organolithium intermediates with electrophiles proceeded with excellent enantioselectivity, enabling the synthesis of highly enantioenriched tertiary alcohol and tertiary thiol derivatives. Therefore, we have established a new class of organolithium reagents for asymmetric synthesis.

## Conflict of interest

The authors declare no conflict of interest.

## Supporting information

As a service to our authors and readers, this journal provides supporting information supplied by the authors. Such materials are peer reviewed and may be re‐organized for online delivery, but are not copy‐edited or typeset. Technical support issues arising from supporting information (other than missing files) should be addressed to the authors.

Supplementary
