# Supplementary material for: Asymmetric Synthesis of Tertiary Alcohols and Thiols via Nonstabilized Tertiary α‐Oxy‐ and α‐Thio‐Substituted Organolithium Species
Source: Angew Chem Int Ed Engl. 2017 Aug 7;56(36):10835–9. doi: 10.1002/anie.201706722 (PMC5601220; doi:10.1002/anie.201706722)

Angewandte Chemie
Eine Zeitschrift der Gesellschaft Deutscher Chemiker

Supporting Information

Asymmetric Synthesis of Tertiary Alcohols and Thiols via Nonstabilized Tertiary α-Oxy- and α-Thio-Substituted Organolithium Species

*Alexander P. Pulis, Ana Varela, Cinzia Citti, Pradip Songara, Daniele Leonori, and Varinder K. Aggarwal**

anie_201706722_sm_miscellaneous_information.pdf

1 General Information 1

2 Experimental Procedures for Dialkyl Benzoates 1 3

2.1 Lithiation and Electrophilic Trapping of Dialkyl Benzoates 1 3

General Procedures for Lithiation and Electrophilic Trapping of Dialkyl Benzoates 1 (GP1 and GP2) 3

Tin-lithium exchange of stannyl dialkyl benzoates 2ag and 2ah (GP3) 3

Lithiation and Electrophilic Trapping of Dialkyl Benzoates 1 4

Removal of TIB group in benzoate 2af 17

Derivatization of the secondary alcohol 3 18

3 Experimental Procedures for Dialkyl *S*-Thiobenzoates 4 19

3.1 Synthesis of Dialkyl *S*-Thiobenzoates 4 20

General Procedure for the Synthesis of *S*-Thiobenzoates from Secondary Alcohols (GP4) 21

3.2 Optimisation of the Lithiation Conditions of 4 27

General Procedure for Lithiation/Deuteration of *S*-Thiobenzoates 4a (GP5) 27

3.3 Lithiation and Electrophilic Trapping of Dialkyl *S*-Thiobenzoates 4 28

General Procedure for Lithiation/Electrophilic Trapping of Dialkyl *S*-Thiobenzoates 4 (GP6) 28

Determination of stereochemistry of 9 42

Removal of TIB Group in thiobenzoate 6ad 46

3.4 Studies on enantiomerisation of Li-4a 47

3.5 In situ IR studies 50

4 References 51

5 NMR Spectra 52

1 General Information

All reagents were sourced from commercial suppliers and were used without further purification unless stated otherwise. Where anhydrous conditions were necessary, standard syringe-septa techniques were used with oven dried glassware under a positive pressure of nitrogen. Anhydrous THF, CH_2Cl_2, toluene, hexane, acetonitrile and Et_2O were dried by passing through a modified Grubbs system of alumina columns, manufactured by Anhydrous Engineering. Anhydrous *tert*-butyl methylether (TBME) and cyclopentyl methyl ether (CPME) was purchased from Aldrich and stored over activated 3Å molecular sieves. *N,N,N',N'*-tetramethylethane-1,2-diamine (TMEDA) was distilled from CaH_2. *s*BuLi solutions were purchased from Acros and periodically titrated by ^{1}H NMR.[1] All stated temperatures below ambient are the temperatures of the cooling baths, unless otherwise stated. A Labplant RP100CD refrigerated immersion probe was used for cooling bath temperatures between −70-−50 °C using acetone as the coolant which was mixed via a mechanical overhead stirrer.

Flash column chromatography was performed using silica gel 60 (Aldrich) and a suitable eluent. TLC was performed with aluminium backed silica TLC plates (Meck-Kieselgel 60 F_{254}) with a suitable

solvent system and was visualised using UV fluorescence (254 & 366 nm) and/or developed with phosphomolybdic acid or potassium permanganate. Infrared spectra were recorded on a Perkin Elmer Spectrum 100 FTIR with an ATR accessory and frequencies are reported in wavenumbers (cm^{-1}). ^{1}H, ^{13}C and ^{11}B NMR spectra were recorded using Jeol Lambda 300 MHz, Jeol ECS 300 MHz, Jeol ECP 400 MHz, Jeol ECS 400 MHz, Varian 400 MR (400 MHz), and Varian VMR S500 MHz spectrometers at ambient temperature. Chemical shifts (δ) are quoted in parts per million (ppm) and coupling constants (*J*) are in hertz (Hz). Tetramethylsilane or residual solvent peaks were used as the internal reference for proton and carbon chemical shifts. HRMS EI and CI were performed on VG Analytical Autospec three sector mass spectrometer at 70eV. For CI, methane was used as the reagent gas. HRMS ESI were performed on either a Bruker Daltonics Apex 4, 7 Tesla FTICR or microTOF II. Samples were submitted in MeOH or DCM. Chiral HPLC was performed on a HP Agilent 1100 with a Chiralpak IA, IB or IC column eluting *i*PrOH/hexane at ambient temperature, unless otherwise stated, and monitored by DAD (Diode Array Detector). Chiral SFC was performed using Daicel Chiralpak IA, IB, and IC columns (4.6 × 250 mm × 5 μm) or a Whelk O-1 column (4.6 × 250 mm × 5 μm) on a Waters TharSFC system and monitored by DAD (Diode Array Detector). Retentions times (T_R) are quoted in minutes. Optical rotation ($[\alpha]_D^T$) was measured on a Bellingham and Stanley Ltd. ADP220 polarimeter and is quoted in (° ml)(g dm)$^{-1}$.

2 EXPERIMENTAL PROCEDURES FOR DIALKYL BENZOATES 1

Benzoates **1a-e** (99:1 *er*) were synthesised according to literature methods.[2]

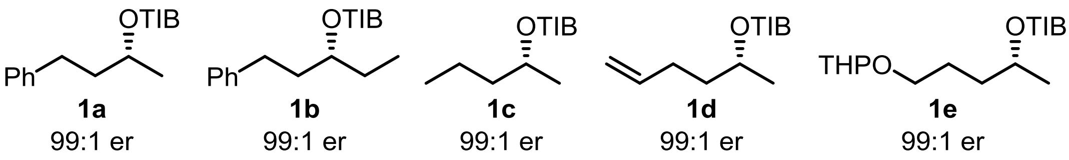

2.1 Lithiation and Electrophilic Trapping of Dialkyl Benzoates 1

General Procedures for Lithiation and Electrophilic Trapping of Dialkyl Benzoates 1 (GP1 and GP2)

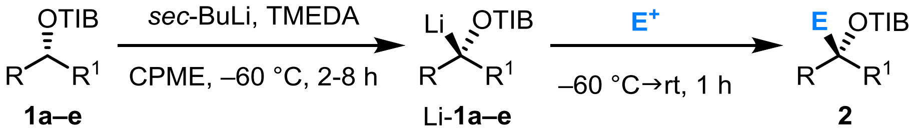

GP1

Following a procedure from Pulis *et al*,[1] *s*BuLi (1.3 M in hexane, 1.6 eq, 0.62 ml, 0.80 mmol) was added dropwise over 10 min to a vigorously stirred solution (without splashing) of benzoate **1** (1.0 eq, 0.50 mmol) and TMEDA (6.0 eq, 0.46 ml, 3.00 mmol) in anhydrous CPME (3 ml) at −60 °C (internal temperature) under a nitrogen atmosphere. The reaction mixture was stirred for 2 h for benzoate **1a** and **1c**, 6 h for benzoate **1b**, 4 h for benzoate **1d**, and 8 h for benzoate **1e**. A solution of the electrophile (2.0 eq, 1.00 mmol) in CPME (1 ml) was added dropwise over 10 min. The reaction mixture was stirred at −60 °C for 1 h, then the cooling bath was removed and the reaction mixture was stirred at room temperature for 1 h. The reaction was quenched with H_2O (10 ml) and saturated $NH_4Cl_{(aq)}$ (10 ml) (or 2 M NaOH for stannane electrophiles). The reaction mixture was extracted with Et_2O (4 × 20 ml) and the combined organic phases were washed with brine and dried over $MgSO_4$. The solvent was removed *in vacuo* and the crude material purified by flash chromatography to give pure benzoates **2**.

GP2

As in **GP1**, except cooling the reaction to −78 °C before the addition of the electrophile.

Tin-lithium exchange of stannyl dialkyl benzoates 2ag and 2ah (GP3)

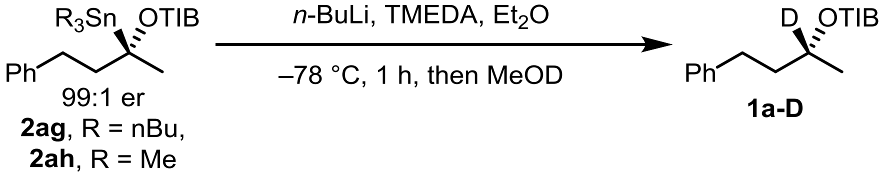

*n*BuLi (1.57 M in hexanes, 1.10 eq, 64.5 µl, 0.10 mmol) was added dropwise to a stirring solution of stannanes **2ag** or **2ah** (1.00 eq, 0.09 mmol) and TMEDA (1.10 eq, 15.0 µl, 0.10 mmol) in anhydrous Et_2O (1.00 ml) at −78 °C under a nitrogen atmosphere. After 1 hour, MeOD (3 M solution in Et_2O, 3.00 eq, 92.0 µl, 0.27 mmol) was added dropwise and the cooling bath was removed. The reaction mixture was filtered through silica and the solvent evaporated to give the pure α-deuterated benzoate D-**1a**.

Lithiation and Electrophilic Trapping of Dialkyl Benzoates 1

(*R*)-1-Methoxy-2-methyl-1-oxo-4-phenylbutan-2-yl 2,4,6-triisopropylbenzoate 2aa

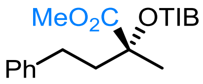

Following a **GP2** using benzoate **1a** (95.0 mg, 0.25 mmol, 99:1 *er*) and methyl chloroformate (38.6 µl, 0.50 mmol) gave (*R*)-tertiary benzoate **2aa** (75.8 mg, 65%, 99:1 *er*) as a white amorphous solid.

R_f (45%CH_2Cl_2/pentane) 0.29. δ_H (400 MHz, $CDCl_3$): 1.29 (6H, d, *J* 6.9), 1.30 (6H, d, *J* 6.9), 1.32 (6H, d, *J* 6.9), 1.84 (3H, s), 2.13-2.31 (2H, m), 2.68-2.81 (2H, m), 2.93 (1H, sept, *J* 6.9), 3.14 (2H, sept, *J* 6.9), 3.82 (3H, s), 7.06 (2H, s), 7.17-7.22 (3H, m), 7.27-7.31 (2H, m). δ_C (100 MHz, $CDCl_3$): 21.2 (CH_3), 24.1 (CH_3), 24.3 (CH_3), 24.5 (CH_3), 29.8 (CH_2), 30.8 (CH), 34.5 (CH), 40.6 (CH_2), 52.3 (CH_3), 81.3 (4° C), 121.0 (CH), 126.2 (CH), 128.4 (CH), 128.6 (CH), 129.7 (4° C), 141.0 (4° C), 145.5 (4° C), 150.5 (4° C), 169.7 (4° C), 172.4 (4° C). ν_{max} (neat): 703, 738, 1878, 1064, 1251, 1455, 1718, 1746, 2962. **HRMS** (CI) calc. for $[C_{28}H_{38}O_4 + Na]^+$ 461.2662. Found 461.2658. **Chiral HPLC** (IB with guard, 0.5% *i*PrOH/hexane, 0.7 ml/min) T_R 13.47 min (minor), 16.22 min (major). $[\alpha]_D^{22}$ −21.8 (*c* 1.01, $CHCl_3$).

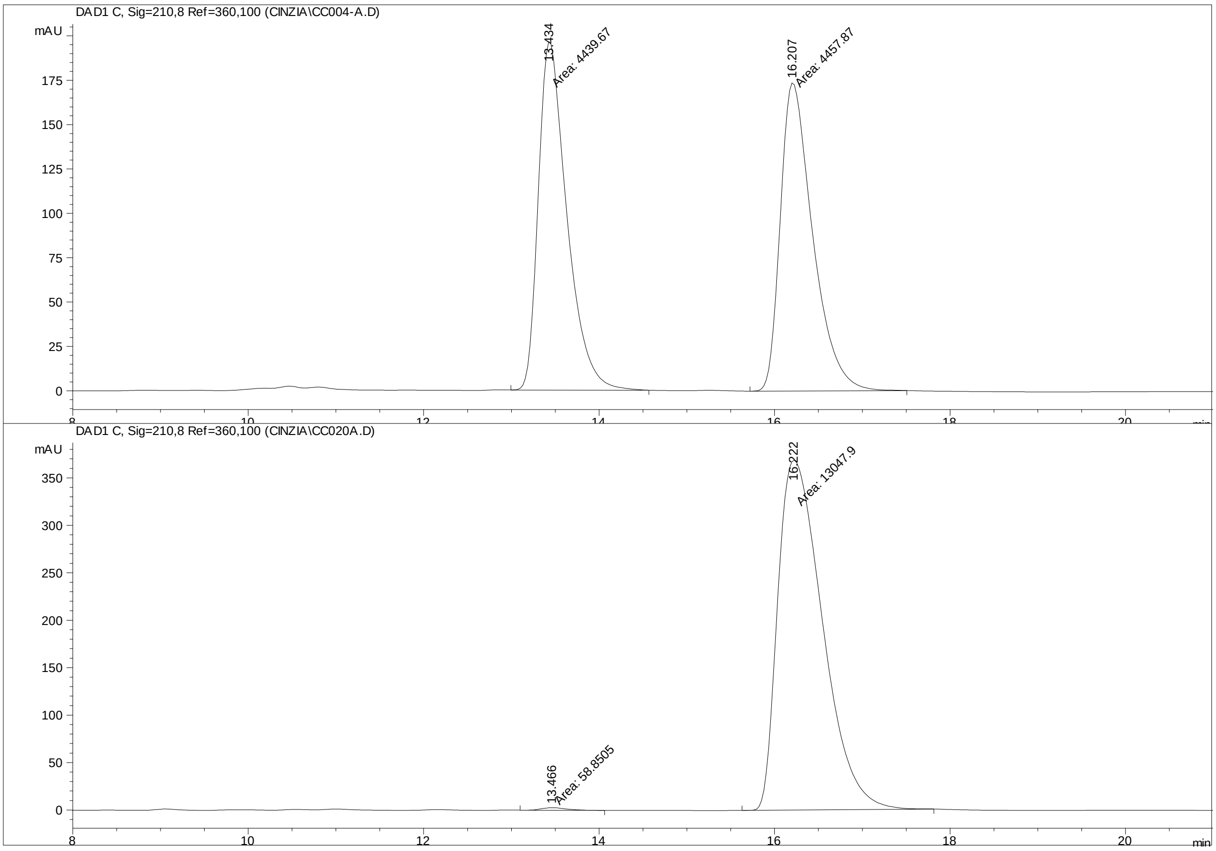

(*R*)-2-Methyl-1-oxo-1,4-diphenylbutan-2-yl 2,4,6-triisopropylbenzoate 2ab

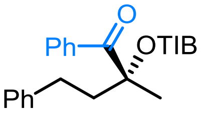

Following a **GP1** using benzoate **1a** (95.0 mg, 0.25 mmol, 99:1 *er*) and benzoyl chloride (58.0 µl, 0.50 mmol) gave (*R*)-tertiary benzoate **2ab** (56.7 mg, 47%, 99:1 *er*) as a colourless oil.

$\boldsymbol{R_f}$ (4% TBME/pentane) 0.35. $\boldsymbol{\delta_H}$ (400 MHz, $CDCl_3$): 1.15 (12H, d, *J* 6.8), 1.26 (6H, d, *J* 6.8), 2.01 (3H, s), 2.44 (1H, td, *J* 13.9, 5.2), 2.50-2.65 (3H, m), 2.73 (1H, td, *J* 12.7, 5.1), 2.83-2.93 (2H, m), 7.00 (2H, s), 7.15-7.22 (3H, m), 7.27-7.30 (2H, m), 7.43-7.47 (2H, m), 7.52-7.57 (1H, m), 8.04-8.06 (2H, m). $\boldsymbol{\delta_C}$ (100 MHz, $CDCl_3$): 21.6 (CH_3), 24.0 (CH_3), 24.2 (CH_3), 24.9 (CH_3), 30.0 (CH_2), 30.7 (CH), 34.5 (CH), 41.2 (CH_2), 87.6 (4° C), 121.2 (CH), 126.3 (CH), 128.4 (CH), 128.5 (CH), 128.7 (CH), 128.9 (CH), 132.3 (CH), 136.0 (4° C), 141.0 (4° C), 146.2 (4° C), 150.8 (4° C), 169.5 (4° C), 199.6 (4° C). $\boldsymbol{\nu_{max}}$ (neat): 697, 751, 1063, 1249, 1460, 1686, 1721, 2962. **HRMS** (CI) calc. for $[C_{33}H_{40}O_3 + Na]^+$ 507.2870. Found 507.2857. **Chiral HPLC** (IB with guard, 0.3% *i*PrOH/hexane, 0.7 ml/min) T_R 16.16 min (minor), 18.50 min (major). $[\alpha]_D^{22}$ –8.3 (*c* 0.72, $CHCl_3$).

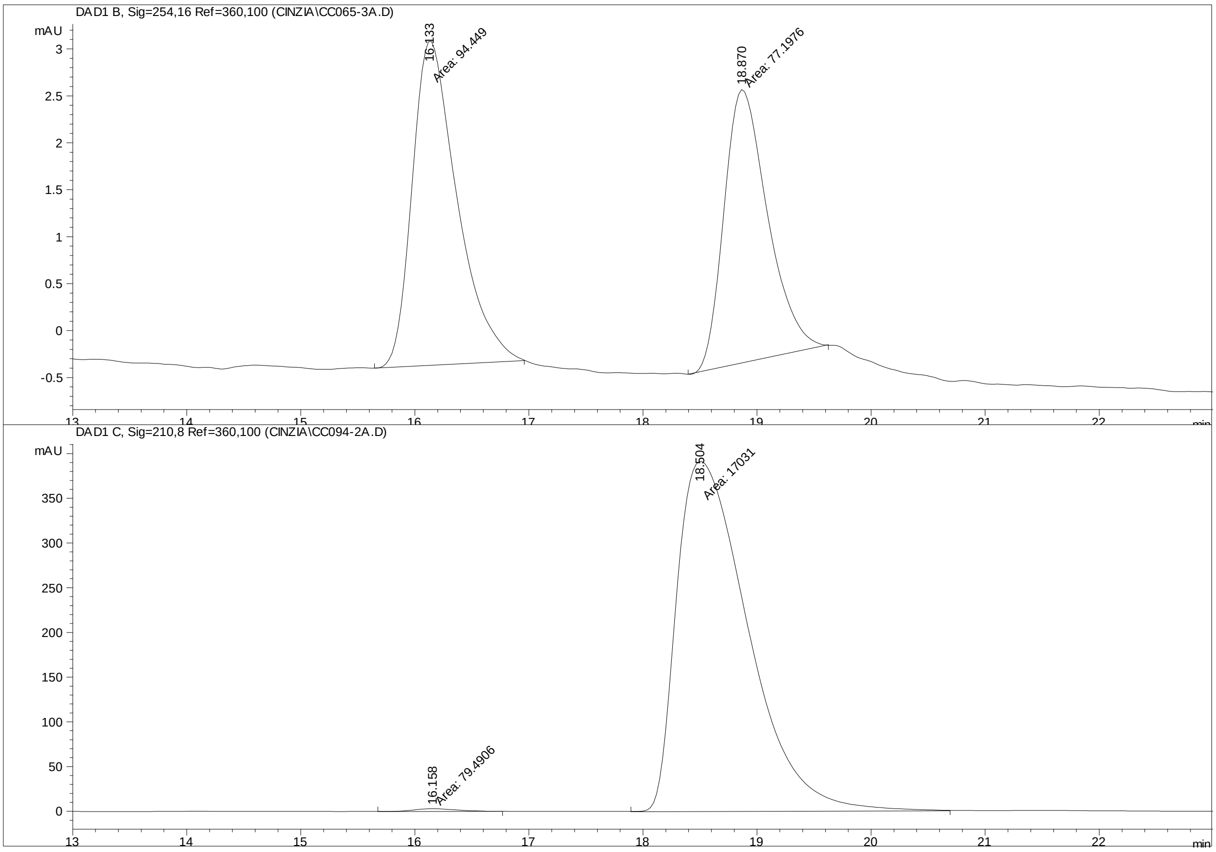

(*R*)-1-(Benzylamino)-2-methyl-1-oxo-4-phenylbutan-2-yl 2,4,6-triisopropylbenzoate 2ac

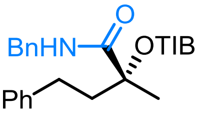

Following a **GP1** using benzoate **1a** (190 mg, 0.50 mmol, 99:1 *er*) and benzylisocyanate (124 µl, 1 mmol) gave (*R*)-tertiary benzoate **2ac** (159 mg, 62%, 99:1 *er*) as a white amorphous solid.

$\boldsymbol{R_f}$ (50%CH_2Cl_2/pentane) 0.31. $\boldsymbol{\delta_H}$ (400 MHz, $CDCl_3$): 1.23 (6H, d, *J* 6.8), 1.24 (6H, d, *J* 6.8), 1.26 (6H, d, *J* 6.8), 1.97 (3H, s), 2.39-2.46 (1H, m), 2.58-2.77 (3H, m), 2.91 (1H, sept, *J* 6.8), 3.03 (2H, sept, *J* 6.8), 4.51-4.54 (2H, m), 6.69 (1H, t, *J* 5.8), 7.03 (2H, s), 7.17-7.23 (3H, m), 7.27-7.35 (7H, m). $\boldsymbol{\delta_C}$ (100 MHz, $CDCl_3$): 22.6 (CH_3), 24.0 (CH_3), 24.3 (CH_3), 24.4 (CH_3), 30.3 (CH_2), 31.4 (CH), 34.5 (CH), 39.5 (CH_2), 43.6 (CH_2), 86.4 (4° C), 121.2 (CH), 126.2 (CH), 127.7(CH), 127.8 (CH), 128.4 (CH), 128.6 (CH), 128.8 (CH), 130.2 (4° C), 138.1 (4° C), 141.2 (4° C), 145.0 (4° C), 150.6 (4° C), 168.9 (4° C), 172.0 (4° C). $\boldsymbol{\nu_{max}}$ (neat): 649, 699, 873, 1076, 1252, 1454, 1535, 1651, 1726, 2959, 3340. **HRMS** (CI) calc. for $[C_{34}H_{43}O_3 + Na]^+$ 536.3135. Found 536.3119. **Chiral HPLC** (IB with guard, 1.5% *i*PrOH/hexane, 0.7 ml/min) T_R 35.28 min (major), 49.04 min (minor). $[\alpha]_D^{22}$ +14.1 (*c* 0.92, $CHCl_3$).

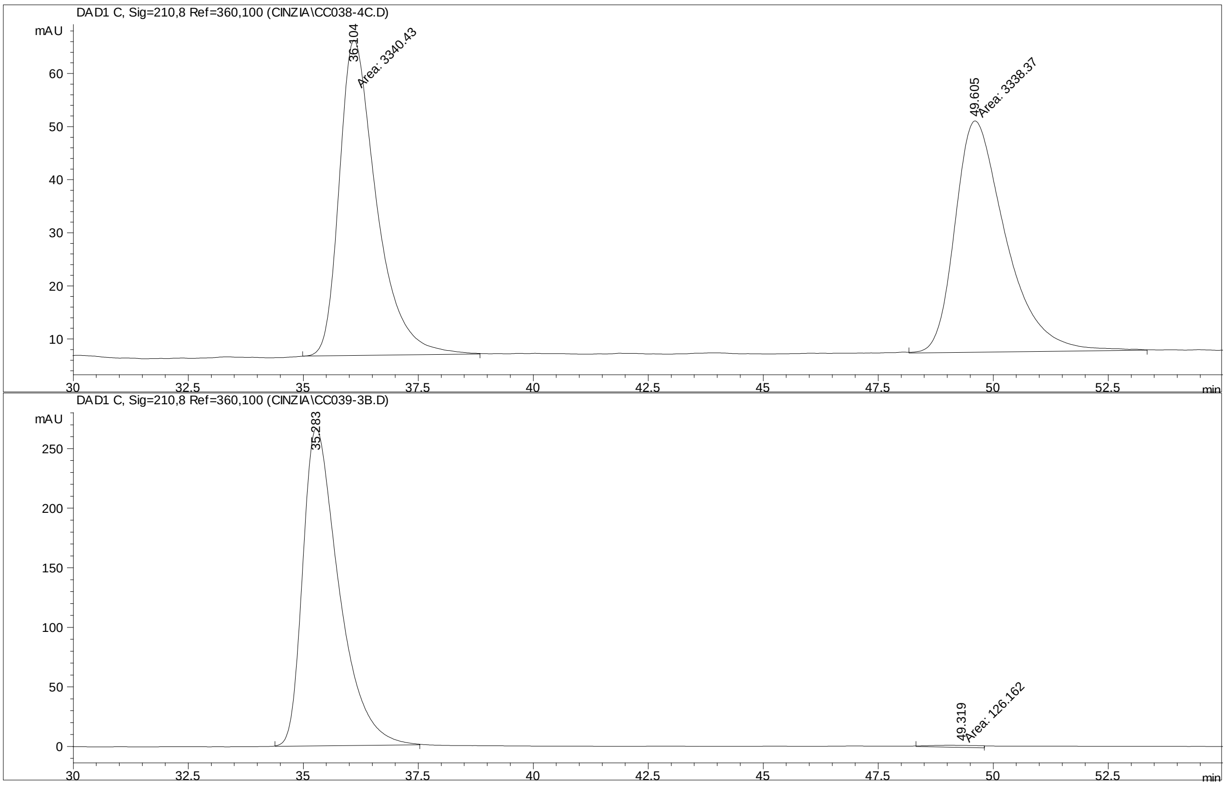

(*R*)-1-((4-Bromophenyl)amino)-2-methyl-1-oxo-4-phenylbutan-2-yl 2,4,6-triisopropylbenzoate 2ad

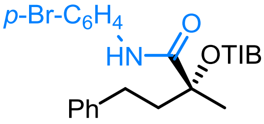

Following a **GP1** using benzoate **1a** (95.0 mg, 0.25 mmol, 99:1 *er*) and *p*-bromophenyl isocyanate (99.0 mg, 0.50 mmol) gave (*R*)-tertiary benzoate **2ad** (103 mg, 71%, 99:1 *er*) as a white amorphous solid.

R_f (8% Et_2O/pentane) 0.28. δ_H (400 MHz, $CDCl_3$): 1.29 (6H, d, *J* 6.8), 1.30 (6H, d, *J* 6.8), 1.32 (6H, d, *J* 6.8), 2.00 (3H, s), 2.48 (1H, td, *J* 12.6, 5.4), 2.61-2.83 (3H, m), 2.94 (1H, sept, *J* 6.8), 3.06 (2H, sept, *J* 6.8), 7.08 (2H, s), 7.16-7.21 (3H, m), 7.27-7.30 (2H, m), 7.39-7.46 (4H, m), 8.08 (1H, br. s). δ_C (100 MHz, $CDCl_3$): 22.6 (CH_3), 24.1 (CH_3), 24.5 (CH_3), 24.6 (CH_3), 30.3 (CH_2), 31.6 (CH), 34.6 (CH), 39.3 (CH_2), 86.6 (4° C), 117.7 (4° C), 121.3 (CH), 122.0 (CH), 126.3 (CH), 128.4 (CH), 128.7 (CH), 129.9 (4° C), 132.2 (CH), 136.2 (4° C), 140.8 (4° C), 145.1 (4° C), 150.9 (4° C), 168.7 (4° C), 170.4 (4° C). ν_{max} (neat): 695, 832, 1066, 1255, 1488, 1529, 1728, 2960, 3367. **HRMS** (CI) calc. for $[C_{33}H_{40}BrO_3 + Na]^+$ 600.2084. Found 600.2084. **Chiral HPLC** (IB with guard, 1.5% *i*PrOH/hexane, 0.7 ml/min) T_R 9.61 min (minor), 11.59 min (major). $[\alpha]_D^{22}$ +32.4 (*c* 1.67, $CHCl_3$).

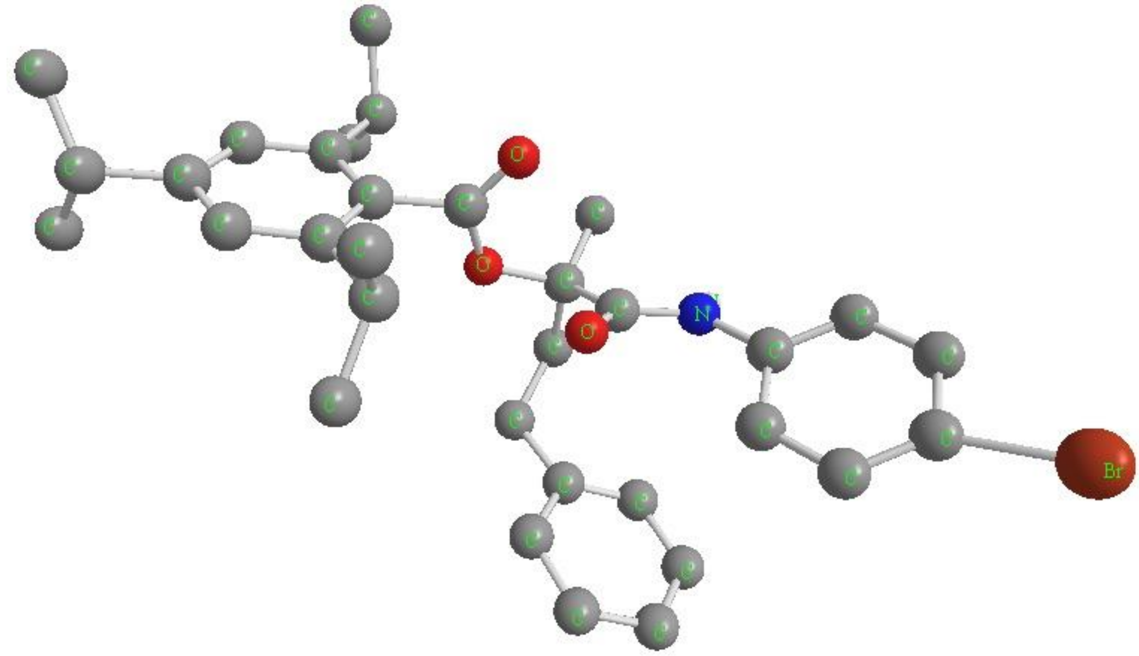

X-ray analysis of **2ad** from ^{i}PrOH/H_2O (CCDC 1557929)

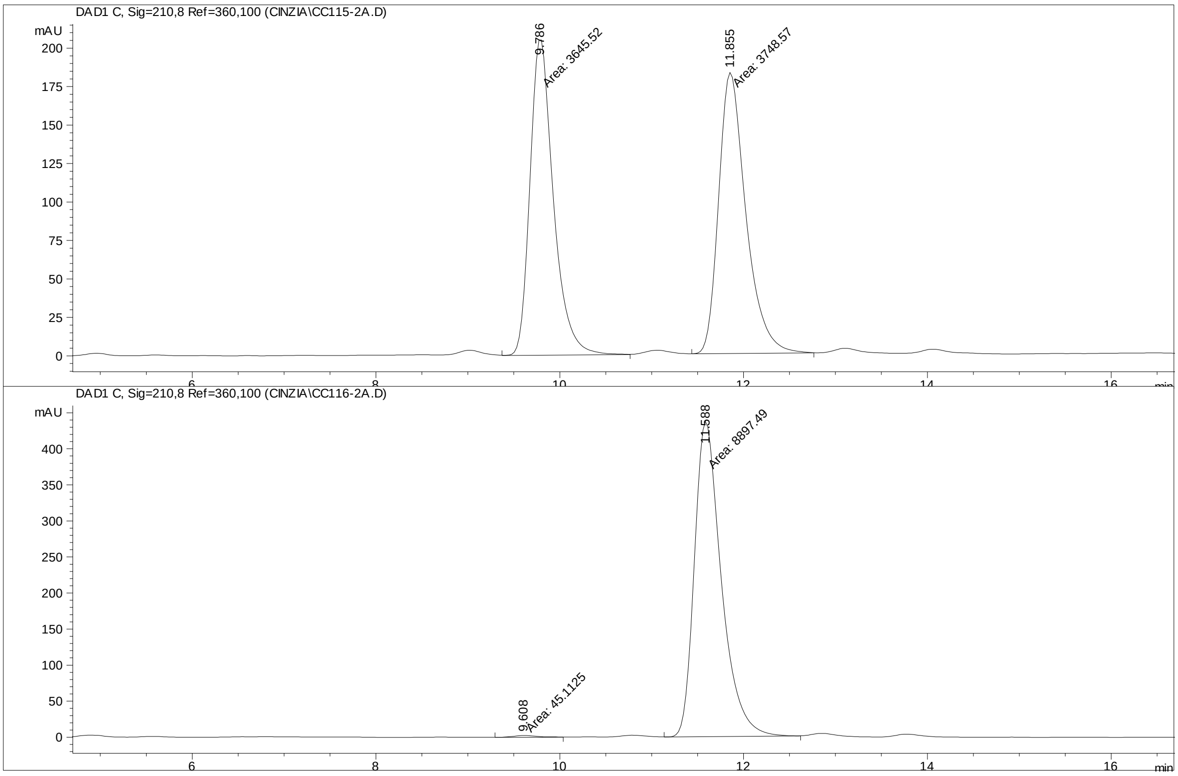

(4*R*)-4-Hydroxy-3,5-dimethyl-1-phenylhexan-3-yl 2,4,6-triisopropylbenzoate 2ae

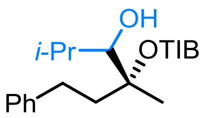

Following a **GP2** using benzoate **1a** (190 mg, 0.50 mmol, 99:1 *er*) and isobutyraldehyde (91.3 µl, 1.00 mmol) gave (*R*)-tertiary benzoate **2ae** (2:1 *dr**) which after purification gave the major diastereomer (41.0 mg, 18%, >20:1 *dr*, 99:1 *er*) as a white amorphous solid and a mixture of diasteromers (95.3 mg, 42%, 1.3:1 *dr*, 99:1 *er*).

For major diastereomer: $\boldsymbol{R_f}$ (60% DCM/pentane) 0.38. $\boldsymbol{\delta_H}$ (400 MHz, $CDCl_3$): 1.04 (3H, d, *J* 6.9), 1.12 (3H, d, *J* 6.9), 1.20 (6H, d, *J* 6.8), 1.24 (6H, d, *J* 6.8), 1.26 (6H, d, *J* 6.7), 1.32 (3H, s), 1.86 (2H, ddd, *J* 8.6, 6.8, 5.1), 2.18 (1H, sept.d, *J* 6.8, 1.8), 2.76-2.83 (2H, m), 2.90 (1H, sept, *J* 6.8), 3.04 (2H, sept, *J* 6.8), 5.17 (1H, d, *J* 1.9), 7.03 (2H, s), 7.17-7.22 (3H, m), 7.27-7.31 (2H, m). *Distinct signals for minor diasteromer*: 5.09 (1H, d, *J* 2.3). $\boldsymbol{\delta_C}$ (100 MHz, $CDCl_3$): 17.2 (CH_3), 22.4 (CH_3), 22.6 (CH_3), 24.1 (CH_3), 24.5 (CH_3), 24.6 (CH_3), 29.0 (CH), 30.0 (CH_2), 31.5(CH), 34.5 (CH), 43.1 (CH_2), 75.4 (4° C), 83.8 (CH), 121.2 (CH), 126.0 (CH), 128.5 (CH), 128.6 (CH), 130.5 (4° C), 142.3 (4° C), 145.2 (4° C), 150.2 (4° C), 172.0 (4° C). $\boldsymbol{\nu_{max}}$ (neat): 696, 746, 929, 1062, 1244, 1458, 1694, 2958, 3510. **HRMS** (CI) calc. for $[C_{30}H_{44}O_3 + Na]^+$ 475.3183. Found 475.3191. **Chiral HPLC** (major diastereomer, IB, 0.2% *i*PrOH/hexane, 0.7 ml/min) T_R 24.04 min (minor), 48.97 min (major). $[\alpha]_D^{22}$ −26.4 (major diasteromer, >20:1 *dr*, *c* 0.87, $CHCl_3$); −17.5 (major of diasteromers, 1.3:1 *dr*, *c* 1.03, $CHCl_3$). *Determined by ^{1}H-NMR of crude reaction mixture

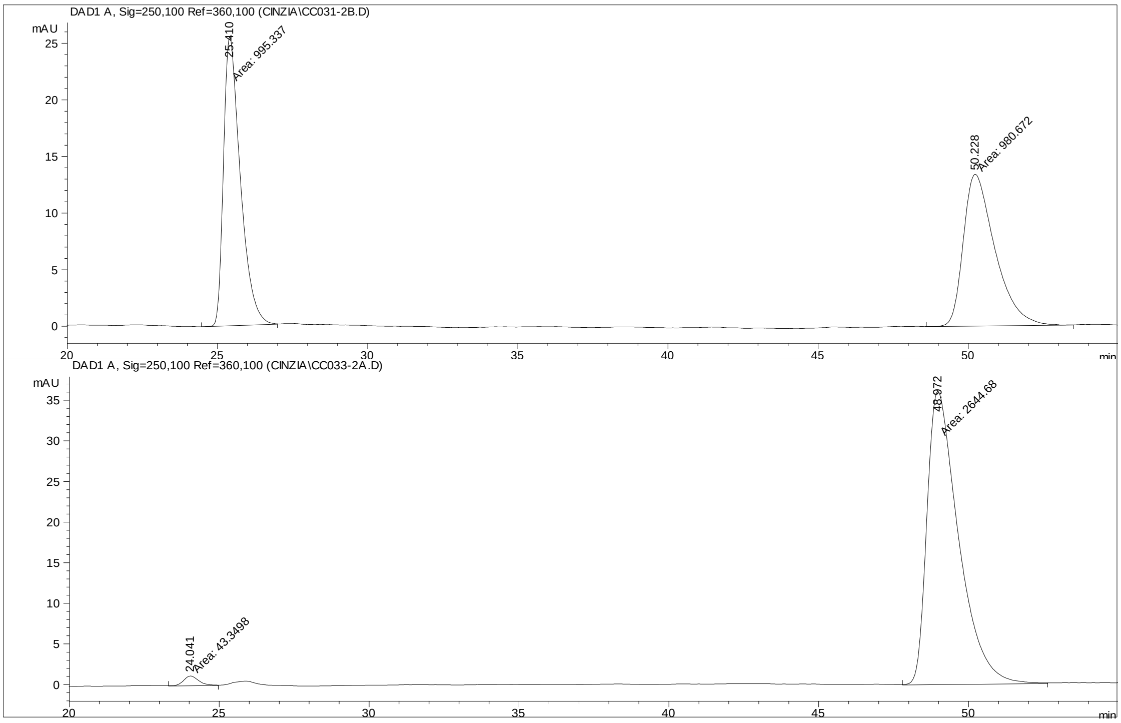

(2*R*)-1-Hydroxy-2-methyl-1,4-diphenylbutan-2-yl 2,4,6-triisopropylbenzoate 2af

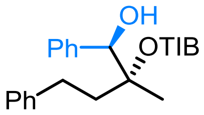

Following procedure **GP2** using benzoate **1a** (190 mg, 0.50 mmol, 99:1 *er*) and benzaldehyde (102 µl, 1.00 mmol), gave (*R*)-tertiary benzoate **2af** (11:1 *dr*) which after purification gave the major diastereomer (78.6 mg, 32%, >20:1 *dr*, 99:1 *er*) as a white amorphous solid and a mixture of diasteromers (84.2 mg, 35%, 5:1 *dr*, 99:1 *er*) a white amorphous solid.

$\boldsymbol{R_f}$ (50% CH_2Cl_2/pentane) 0.27. δ_H (400 MHz, $CDCl_3$): 1.06 (6H, d, *J* 6.7), 1.15 (6H, d, *J* 6.7), 1.21 (3H, s), 1.24 (6H, d, *J* 6.7), 1.80-1.99 (2H, m), 2.61 (2H, sept, *J* 6.7), 2.75-2.93 (3H, m), 5.94 (1H, s), 6.98 (2H, s), 7.17-7.21 (3H, m), 7.27-7.31 (2H, m), 7.34-7.38 (3H, m), 7.47-7.51 (2H, m). *Distinct signal for minor diasteromer*: 5.96 (1H, s). δ_C (100 MHz, $CDCl_3$): 22.4 (CH_3), 24.1 (CH_3), 24.4 (CH_3), 30.1 (CH_2), 31.4 (CH), 34.5 (CH), 41.2 (CH_2), 74.4 (4° C), 82.1 (CH), 121.1 (CH), 126.0 (CH), 128.1 (CH), 128.5 (CH), 128.6 (CH), 128.9 (CH), 130.1 (4° C), 136.8 (4° C), 142.2 (4° C), 145.1 (4° C), 150.4 (4° C), 170.2 (4° C). ν_{max} (neat): 696, 746, 959, 1066, 1248, 1458, 1708, 2957, 3513. **HRMS** (CI) calc. for $[C_{33}H_{42}O_3 + Na]^+$ 509.3026. Found 509.3012 (major diasteromer). **Chiral HPLC** (major diasteromer, IB with guard, 0.75% *i*PrOH/hexane, 0.7 ml/min) T_R 22.20 min (minor), 32.21 min (major). $[\alpha]_D^{22}$ −26.5 (major diastereomer, >20:1 *dr*, *c* 2.15, $CHCl_3$); −13.5 (mixture of diastereomers, 5:1 *dr*, *c* 0.96, $CHCl_3$). *Determined by 1H-NMR of crude reaction mixture.

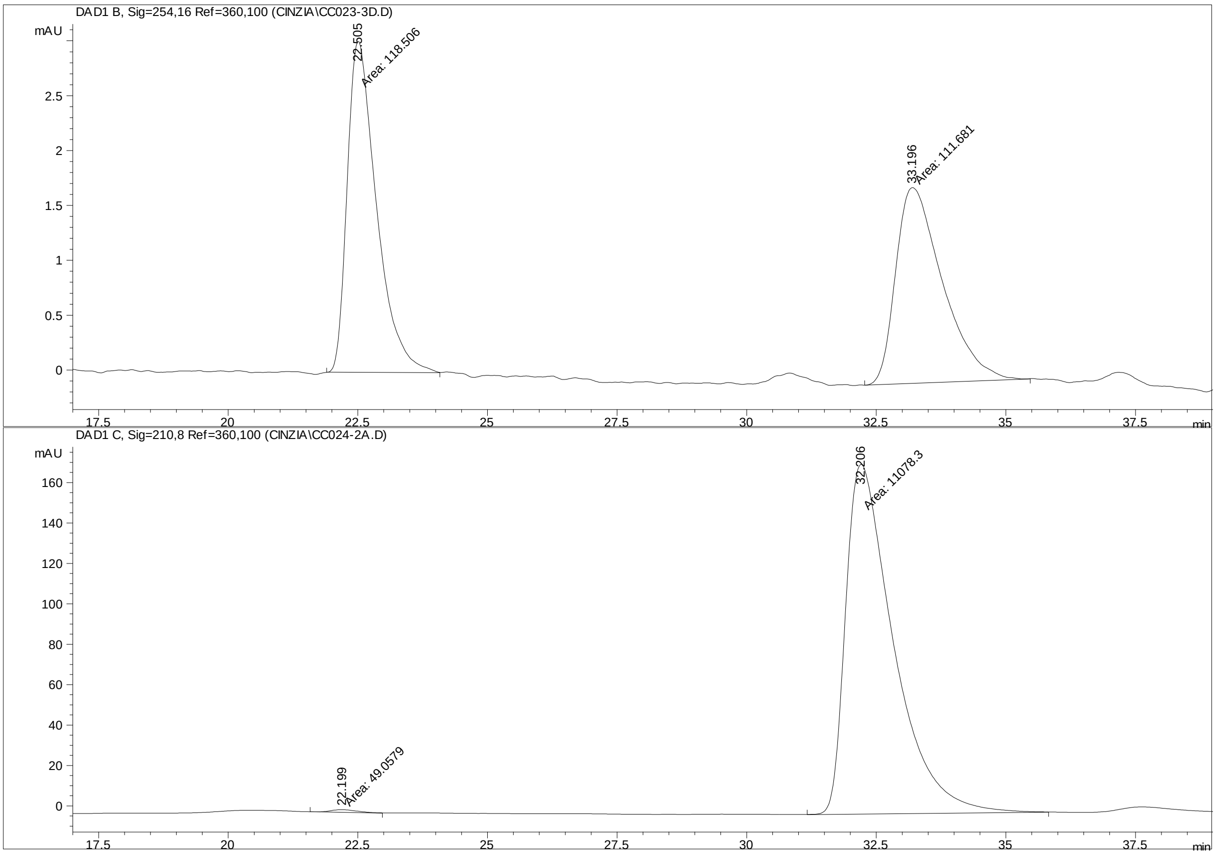

(*S*)-4-Phenyl-2-(tributylstannyl)butan-2-yl 2,4,6-triisopropylbenzoate 2ag

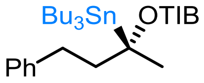

Following a **GP1** using benzoate **1a** (190 mg, 0.50 mmol, 99:1 *er*) and tributyltin chloride (271 µl, 1.00 mmol) gave (*S*)-tertiary benzoate **2ag** (286 mg, 85%, 99:1 *er**) as a white amorphous solid.

R R_f (5% CH_2Cl_2/pentane) 0.41. δ_H (400 MHz, $CDCl_3$): 0.95 (9H, t, *J* 7.4), 1.04-1.08 (6H, m), 1.27 (6H, d, *J* 6.8), 1.28 (6H, d, *J* 6.8), 1.31 (6H, d, *J* 6.8), 1.40 (6H, tq, *J* 7.4, 7.4), 1.57-1.67 (6H, m), 1.62 (3H, s), 2.02-2.19 (2H, m), 2.63 (1H, td, *J* 12.8, 4.6), 2.85 (1H, td, *J* 12.8, 4.6), 2.88-3.02 (3H, m), 7.04 (2H, s), 7.19-7.23 (3H, m), 7.29-7.32 (2H, m). δ_C (100 MHz, $CDCl_3$): 11.8 (CH_2, d, J_{Sn} 328.1, 313.7), 13.9 (CH_3), 24.1 (CH_3), 24.4 (CH_3), 24.7 (CH_3), 26.8 (CH_3), 27.9 (CH_2), 29.5 (CH_2), 31.3 (CH), 33.2 (CH_2), 34.5 (CH), 44.5 (CH_2), 81.9 (4° C), 121.0 (CH), 126.1 (CH), 128.4 (CH), 128.5 (CH), 130.7 (4° C), 142.3 (4° C), 145.1 (4° C), 150.1 (4° C), 172.0 (4° C). ν_{max} (neat): 662, 756, 873, 1047, 1139, 1263, 1460, 1688, 2955. **HRMS** (CI) calc. for $[C_{38}H_{62}O_2Sn + Na]^+$ 693.3672. Found 693.3667. $[\alpha]_D^{22}$ +24.2 (*c* 1.2, $CHCl_3$). *determined after tin-lithium exchange according to **GP3**, which gave (*R*)-benzoate **1a** (99:1 *er*) [**Chiral HPLC of obtained (*R*)-benzoate 1a** (IB, 0.3% *i*PrOH/hexane, 0.3 ml/min, 5 °C) T_R 27.4 min (minor), 29.6 min (major)].

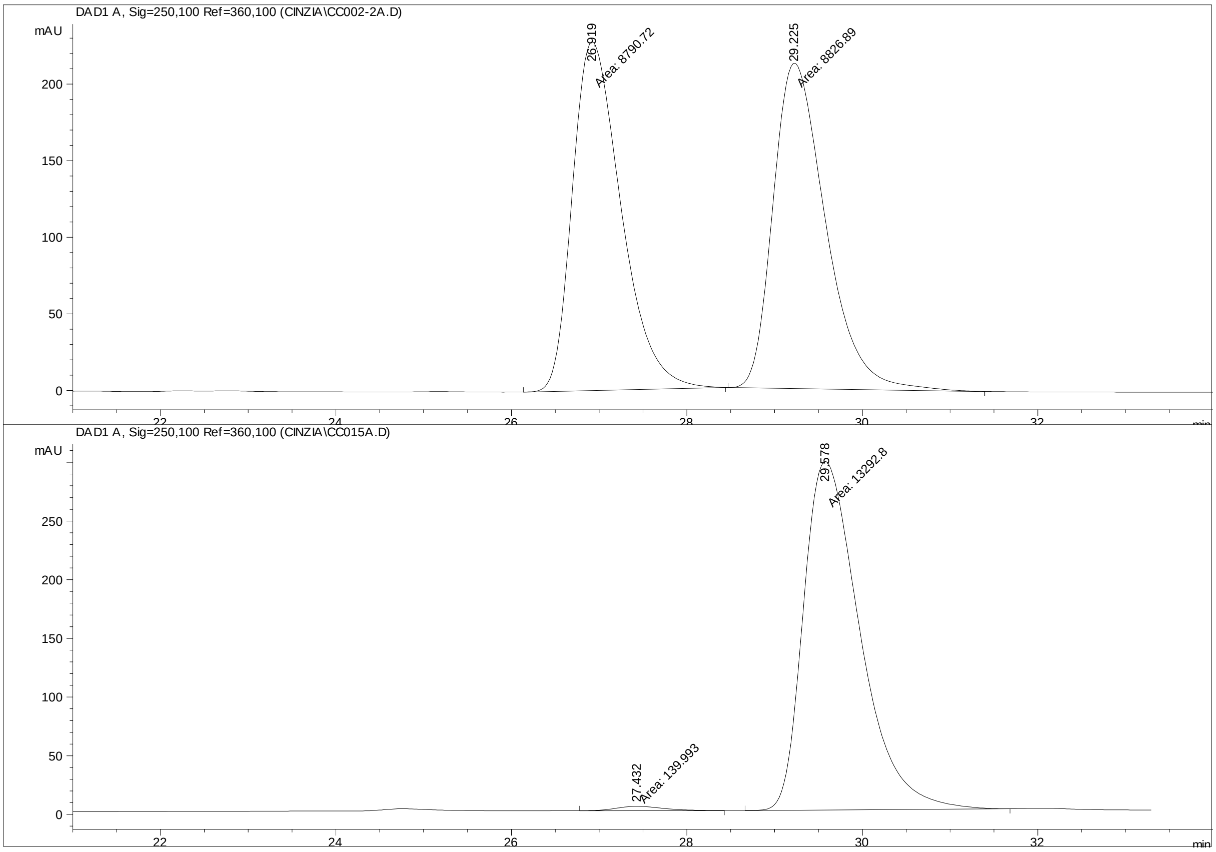

(*S*)-4-Phenyl-2-(trimethylstannyl)butan-2-yl 2,4,6-triisopropylbenzoate 2ah

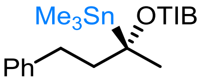

Following **GP1** using benzoate **1a** (760 mg, 2.00 mmol, 99:1 *er*) and trimethyltin chloride (1 M solution in hexanes, 4.00 mmol, 4.00 ml), gave (*S*)-tertiary benzoate **2ah** (896 mg, 82%, 99:1 *er**) as a white amorphous solid.

R_f (0.8%Et_2O/pentane) 0.37. δ_H (400 MHz, $CDCl_3$): 0.27 (9H, s, J_{Sn} 52.3, 50.2), 1.26 (12H, d, *J* 6.9), 1.29 (6H, d, *J* 6.9), 1.59 (3H, s), 2.04 (1H, ddd, *J* 13.9, 12.9, 4.4), 2.16 (1H, ddd, *J* 13.9, 12.9, 4.4), 2.64 (1H, td, *J* 12.9, 4.4), 2.85 (1H, td, *J* 12.9, 4.4), 2.94 (3H, sept, *J* 6.9), 7.03 (2H, s), 7.17-7.21 (3H, m), 7.27-7.31 (2H, m). δ_C (100 MHz, $CDCl_3$): -6.9 (CH_3, d, J_{Sn} 339.7, 324.9), 24.1 (CH_3), 24.3 (CH_3), 24.7 (CH_3), 26.0 (CH_3), 31.3 (CH_3), 33.1 (CH_2), 34.5 (CH), 44.1 (CH_2), 80.7 (4° C), 121.0 (CH), 126.1 (CH), 124.8 (CH), 128.6 (CH), 130.5 (4° C), 142.1 (4° C), 145.0 (4° C), 150.2 (4° C), 172.3 (4° C). ν_{max} (neat): 700, 765, 876, 1054, 1137, 1260, 1455, 1688, 2955. **HRMS** (CI) calc. for $[C_{29}H_{44}O_2Sn + Na]^+$ 567.2261. Found 567.2263. $[\alpha]_D^{22}$ +33.9 (*c* 1.15, $CHCl_3$). ***determined after tin-lithium exchange according to **GP3**, which gave (*R*)-benzoate **1a** (99:1 *er*).

(*S*)-4-Phenyl-2-(trimethylstannyl)pentan-2-yl 2,4,6-triisopropylbenzoate 2ba

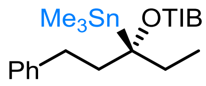

Following a **GP1** (6h lithiation) using benzoate **1b** (98.7 mg, 0.25 mmol, 99:1 *er*) and trimethyltin chloride (0.50 ml, 0.50 mmol), gave (*S*)-tertiary benzoate **2ba** (52.9 mg, 38%, 99:1 *er*)* as a colourless oil.

R R_f (1%Et_2O/pentane) 0.31. δ_H (400 MHz, $CDCl_3$): 0.27 (9H, s, J_{Sn} 52.0, 49.8), 0.99 (3H, t, *J* 7.5), 1.26 (12H, d, *J* 6.9), 1.28 (6H, d, *J* 6.9), 1.82-1.94 (1H, m), 2.08-2.28 (3H, m), 2.63 (1H, td, *J* 13.0, 5.6), 2.81 (1H, td, *J* 13.0, 5.6), 2.92 (3H, sept, *J* 6.9), 7.02 (2H, s), 7.18-7.22 (3H, m), 7.27-7.32 (2H, m). δ_C (100 MHz, $CDCl_3$): −6.0 (CH_3, d, J_{Sn} 338.5, 323.7), 10.1 (CH_3), 24.1 (CH_3), 24.5 (CH_3), 24.6 (CH_3), 30.4 (CH), 31.4 (CH), 31.5 (CH_2), 32.5 (CH_2), 34.5 (CH), 40.5 (CH_2), 86.3 (4° C), 121.0 (CH), 126.1 (CH), 128.4 (CH), 128.6 (CH), 130.5 (4° C), 142.2 (4° C), 145.0 (4° C), 150.1 (4° C), 172.4 (4° C). ν_{max} (neat): 701, 756, 873, 1047, 1074, 1139, 1460, 1689, 2956. **HRMS** (CI) calc. for $[C_{30}H_{46}O_2Sn + Na]^+$ 581.2418. Found 581.2421. *Determined after tin-lithium exchange according to **GP3**, which gave (*R*)-benzoate **1b** (99:1 *er*). **Chiral HPLC** (IB with guard, 0.3% *i*PrOH/hexane, 0.3 ml/min, 5 °C) T_R 20.09 min (minor), 22.66 min (major). $[\alpha]_D^{22}$ +18.5 (*c* 0.81, $CHCl_3$).

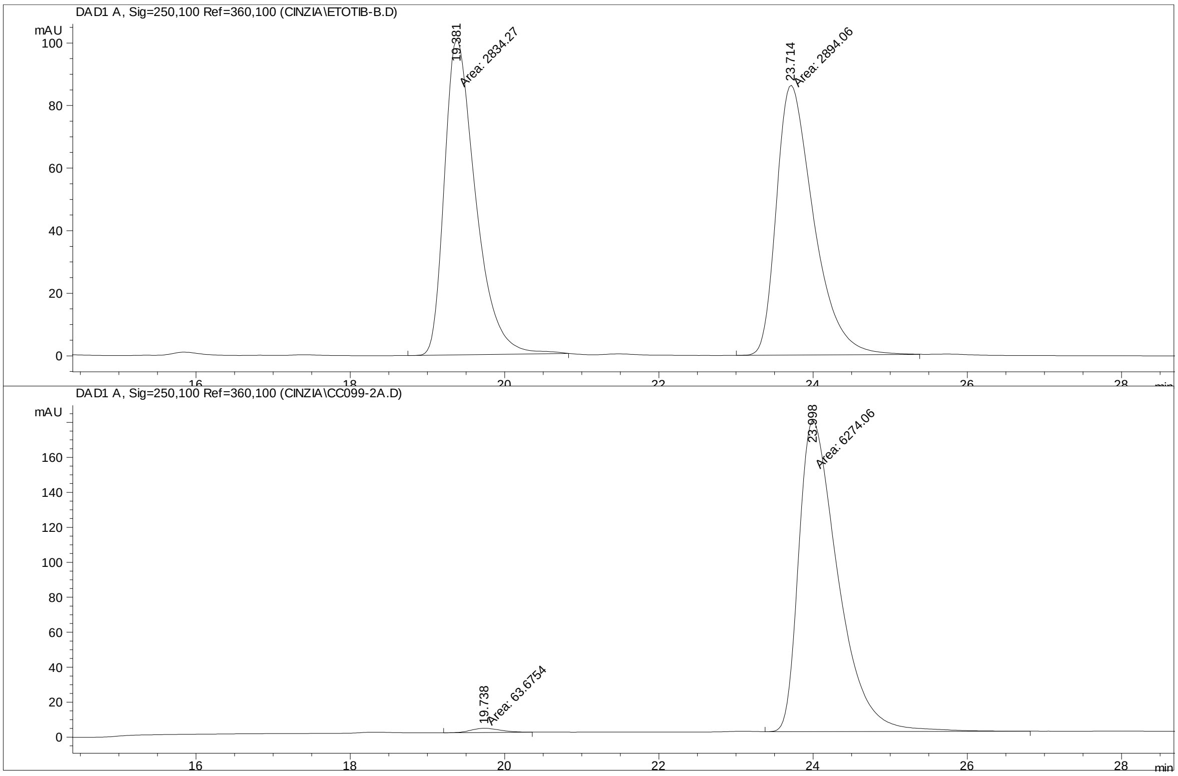

(*S*)-2-(Trimethylstannyl)pentan-2-yl 2,4,6-triisopropylbenzoate 2ca

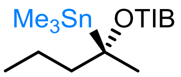

Following a **GP1** using benzoate **1c** (955 mg, 3.00 mmol, 99:1 *er*), *s*Buli (1.3 M, 6.92 ml, 9.00 mmol), TMEDA (5.40 ml, 36.0 mmol) and trimethyltin chloride (12.0 ml, 12.0 mmol), gave (*S*)-tertiary benzoate **2ca** (538 mg, 37%, 99:1 *er**) as a colourless oil.

Rf (1%Et_2O/pentane) 0.33. δ_H (400 MHz, $CDCl_3$): 0.18 (9H, s, J_{Sn} 52.5, 50.1), 0.92 (3H, t, *J* 7.2), 1.23 (6H, d, *J* 6.8), 1.24 (6H, d, *J* 6.8), 1.25 (6H, d, *J* 6.8), 1.28-1.41 (1H, m), 1.46-1.59 (1H, m), 1.52 (3H, s), 1.69 (1H, ddd, *J* 13.7, 12.3, 4.2), 1.85 (1H, ddd, *J* 13.7, 12.3, 4.2), 2.88 (1H, sept, *J* 6.8), 2.89 (2H, sept, *J* 6.8), 6.99 (2H, s). δ_C (100 MHz, $CDCl_3$): −7.0 (CH_3, d, J_{Sn} 338.3, 323.6), 14.6 (CH_3), 19.9 (CH_2), 24.1 (CH_3), 24.2 (CH_3), 24.6 (CH_3), 26.0 (CH), 31.2 (CH), 34.5 (CH), 44.1 (CH_2), 81.3 (4° C), 120.9 (CH), 130.7 (4° C), 144.9 (4° C), 150.1 (4° C), 172.2 (4° C). ν_{max} (neat): 761, 876, 1077, 1102, 1261, 1289, 1460, 1695, 2960. **HRMS** (CI) calc. for $[C_{24}H_{42}O_2Sn + Na]^+$ 505.2103. Found 505.2089. *Determined after tin-lithium exchange according to **GP3**, which gave (*R*)-benzoate **1c** (99:1 *er*).

(*S*)-2-(Trimethylstannyl)hex-5-en-2-yl 2,4,6-triisopropylbenzoate 2da

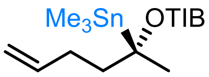

Following a **GP1** (4h lithiation) using benzoate **1d** (82.6 mg, 0.25 mmol, 99:1 *er*) and trimethyltin chloride (0.50 ml, 0.50 mmol), gave (*S*)-tertiary benzoate **2da** (77.0 mg, 62%, 99:1 *er**) as a colourless oil.

Rf (1%Et_2O/pentane) 0.34. δ_H (400 MHz, $CDCl_3$): 0.20 (9H, s, J_{Sn} 52.4, 50.3), 1.23 (6H, d, *J* 6.9), 1.24 (6H, d, *J* 6.9), 1.25 (6H, d, *J* 6.9), 1.54 (3H, s), 1.80 (1H, ddd, *J* 13.7, 12.4, 4.3), 1.96 (1H, ddd, *J* 13.7, 12.4, 4.3), 2.02-2.11 (1H, m), 2.23-2.32 (1H, m), 2.88 (3H, sept, *J* 6.9), 4.93-5.04 (2H, m), 5.80 (1H, ddt, *J* 16.9, 10.3, 6.6), 6.99 (2H, s). δ_C (100 MHz, $CDCl_3$): −7.0 (CH_3, dd, J_{Sn} 339.5, 324.3), 24.1 (CH_3), 24.2 (CH_3), 24.6 (CH_3), 25.9 (CH_3), 30.8 (CH_2), 31.3 (CH), 34.5 (CH), 41.0 (CH_2), 80.6 (4° C), 114.8 (CH_2), 121.0 (CH), 130.5 (4° C), 138.2 (CH), 144.9 (4° C), 150.1 (4° C), 172.2 (4° C). ν_{max} (neat): 763, 876, 910, 1077, 1260, 1288, 1460, 1697, 2961. **HRMS** (CI) calc. for $[C_{25}H_{42}O_2Sn + Na]^+$ 517.2104. Found 517.2108. *Determined after tin-lithium exchange according to **GP3**, which gave (*R*)-benzoate **1d** (99:1 *er*). **Chiral SFC** (*determined after ozonolysis/$NaBH_4$: Chiracel IC, 4% IPA:hexane (1:1, v:v), 4 mL/min, 125 bar, 40 °C, 254 nm) T_R 7.92 min (major), T_R 9.29 min (minor). $[\alpha]_D^{22}$ +25.0 (*c* 0.88, $CHCl_3$).

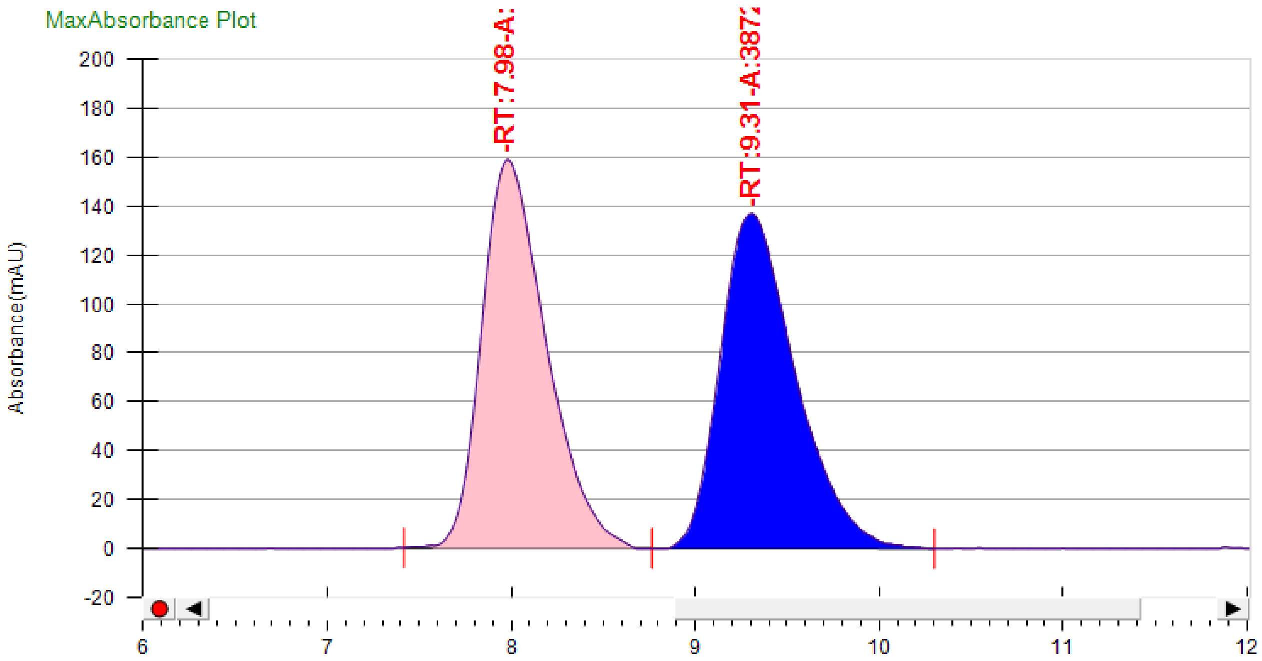

Peak n.	% Area	Area	Ret. Time	Hight	Cap. Factor
1	49.8379	3847.3637	7.98 min	158.6179	7982.2167
2	50.1621	3872.3843	9.31 min	136.6983	9307.2

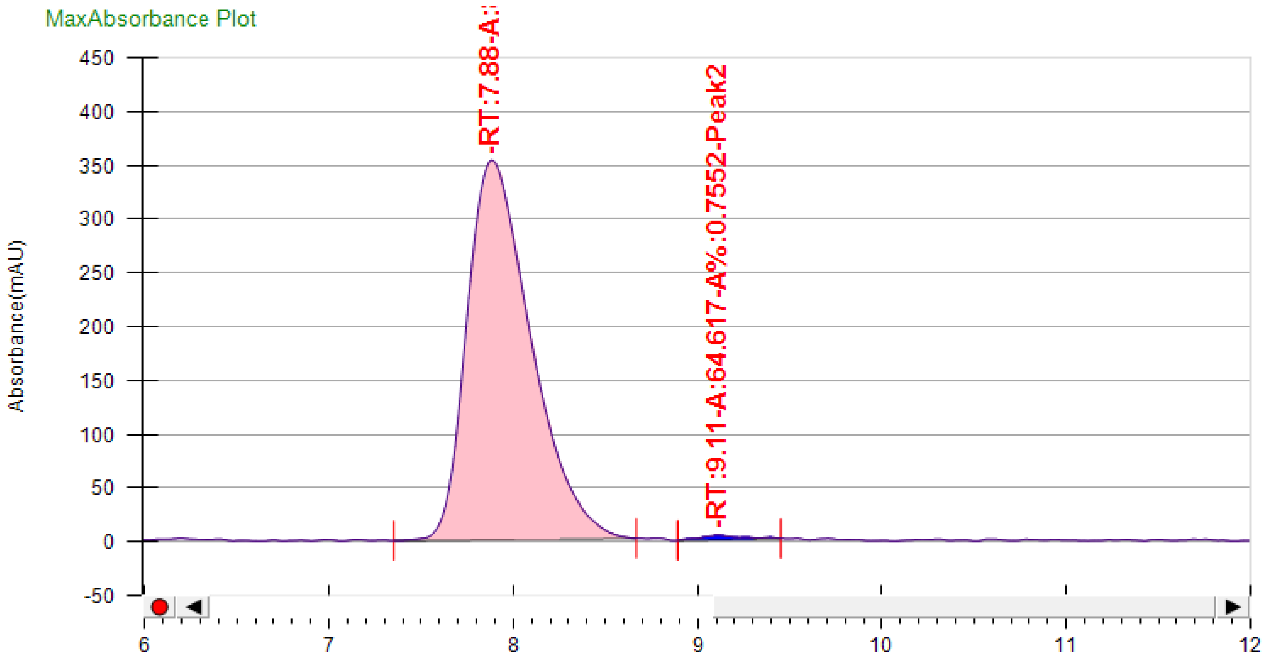

Peak n.	% Area	Area	Ret. Time	Height	Cap. Factor
1	99.2448	8492.0023	7.88 min	352.7652	7882.2167
2	0.7552	64.617	9.11 min	4.4371	9107.2

(2*S*)-5-((Tetrahydro-2*H*-pyran-2-yl)oxy)-2-(trimethylstannyl)pentan-2-yl 2,4,6-triisopropylbenzoate 2ea

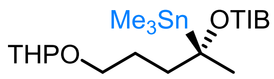

Following a **GP1** (8h lithiation) using benzoate **1e** (105 mg, 0.25 mmol, >99:1 *er*) and trimethyltin chloride (0.50 ml, 0.50 mmol), gave (*S*)-tertiary benzoate **2ea** (73.0 mg, 50%, >99:1 *er**) as a white amorphous solid.

R_f (5%Et_2O/pentane) 0.35. δ_H (400 MHz, $CDCl_3$): 0.21 (9H, s, app. J_{Sn} 51.8), 1.23 (6H, d, *J* 6.9), 1.24 (6H, d, *J* 6.9), 1.25 (6H, d, *J* 6.9), 1.49-2.05 (10H, m), 1.55 (3H, s), 2.88 (1H, hept, *J* 6.9), 2.89 (2H, hept, *J* 6.9), 3.32-3.45 (1H, m), 3.46-3.50 (1H, m), 3.66-3.77 (1H, m), 3.80-3.86 (1H, m), 4.56 (1/2H, t, *J* 3.7), 4.57 (1/2H, t, *J* 3.7), 6.99 (2H, s). δ_C (100 MHz, $CDCl_3$): −7.1 (CH_3, d, J_{Sn} 339.0, 323.5), 19.5 (CH_2), 19.6 (CH_2), 24.1 (CH_3), 24.2 (CH_3), 24.6 (CH_3), 25.6 (CH_2), 25.7 (CH_2), 25.94 (CH_3), 25.98 (CH_3), 26.8 (CH_2), 26.9 (CH_2), 30.8 (CH_2), 30.9 (CH_2), 31.2 (CH), 34.5 (CH), 38.5 (CH_2), 62.1 (CH_2), 62.2 (CH_2), 67.48 (CH_2), 67.49 (CH_2), 80.82 (4° C), 80.84 (4° C), 98.7 (CH), 98.8 (CH), 120.9 (CH), 130.57 (4° C), 130.58 (4° C), 144.9 (4° C), 150.1 (4° C), 172.09 (4° C), 170.1 (4° C). ν_{max} (neat): 737, 764, 1033, 1076, 1263, 1288, 1461, 1696, 2960. **HRMS** (CI) calc. for $[C_{29}H_{50}O_4Sn + Na]^+$ 605.2629. Found 605.2623. ***Determined after tin-lithium exchange according to **GP3** which gave (*R*)-benzoate **1e**, and deprotection of the THP group. **Chiral SFC:** Chiracel IC, 4% IPA:hexane (1:1, v:v), 4 mL/min, 125 bar, 40 °C, 254 nm) T_R 7.92 min (major), T_R 9.29 min (minor). $[\alpha]_D^{22}$ +12.8 (*c* 0.86, $CHCl_3$).

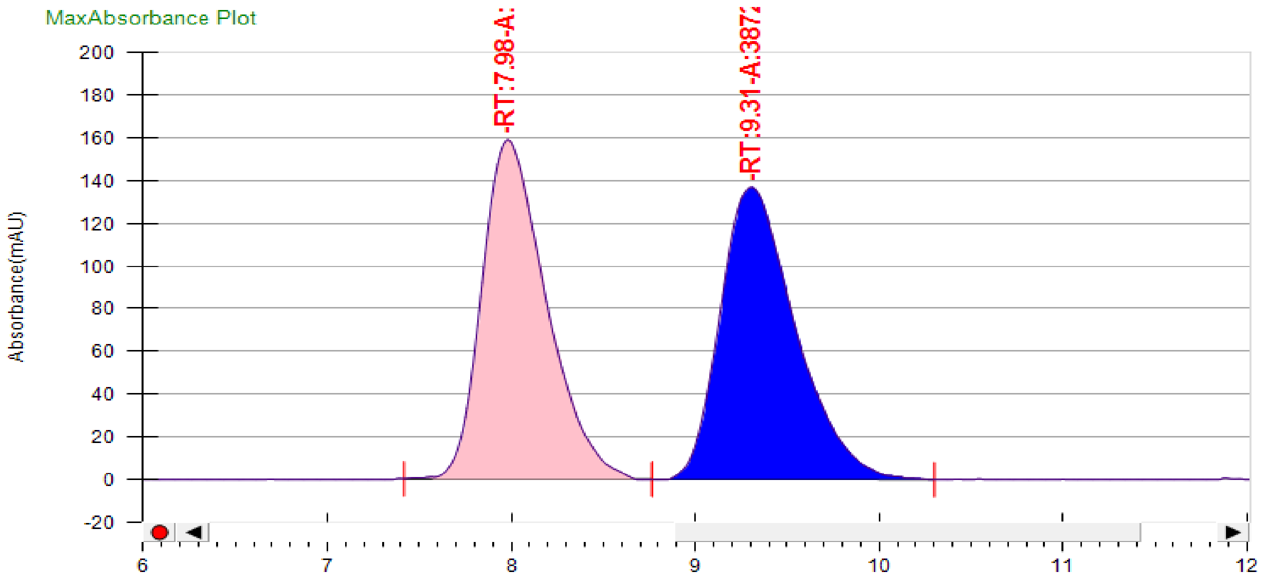

Peak n.	% Area	Area	Ret. Time	Hight	Cap. Factor
1	49.8379	3847.3637	7.98 min	158.6179	7982.2167
2	50.1621	3872.3843	9.31 min	136.6983	9307.2

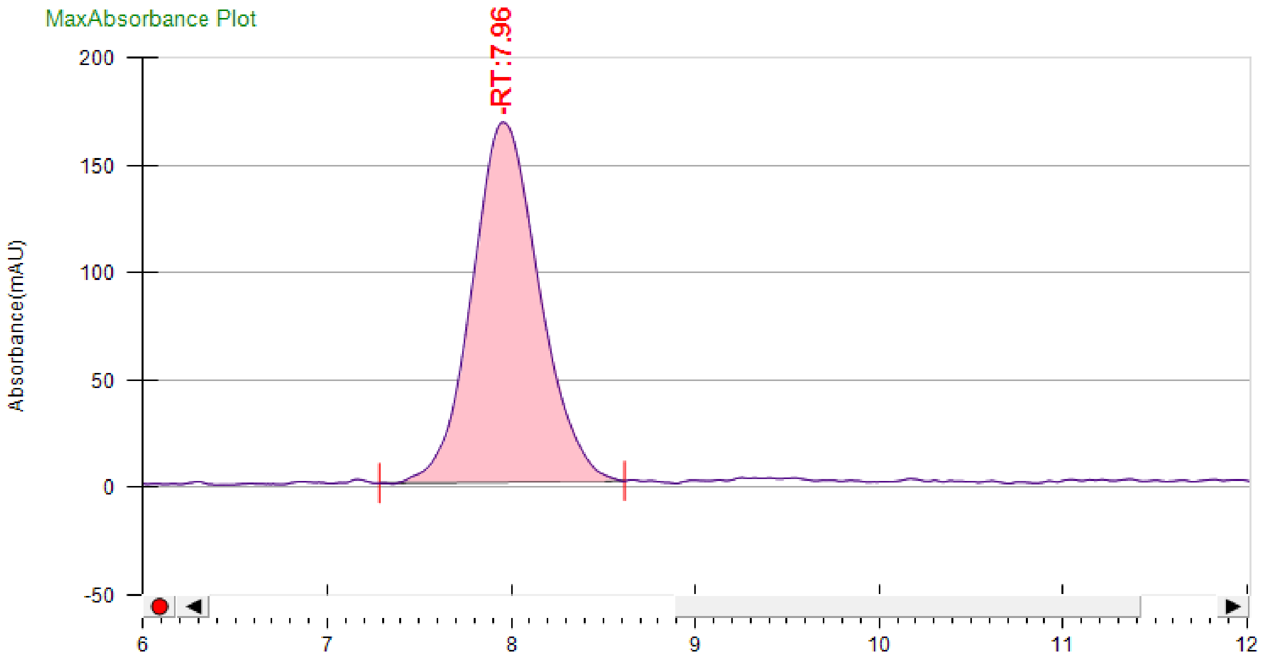

Peak n.	% Area	Area	Ret. Time	Hight	Cap. Factor
1	100	4241.2469	7.96 min	167.3323	0

Removal of TIB group in benzoate 2af

(1*R*,2*R*)-2-Methyl-1,4-diphenylbutane-1,2-diol 3

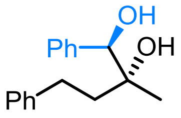

A dry Schlenk tube was charged with $LiAlH_4$ (5.0 eq, 21 mg, 0.55 mmol) and THF (1 ml) under nitrogen atmosphere. A solution of benzoate **2af** (1.0 eq, 53.8 mg, 0.11 mmol, 99:1 er, 20:1 *dr*) in THF (1 ml) was added dropwise to the suspension of $LiAlH_4$ and the mixture was heated at reflux overnight (~15 h). The reaction mixture was cooled to 0 °C and H_2O (38 µl) was added slowly. Then 15% NaOH solution (38 µl) and H_2O (114 µl) were added and the reaction mixture was stirred at ambient temperature for 20 minutes. Diethyl ether (2 ml) was added and the mixture was stirred for 30 minutes. The mixture was filtered through Celite, washing the filter cake with Et_2O (20 ml). H_2O (2 ml) was added, the phases were separated and the aqueous phase was extracted with EtOAc (6 × 10 ml). The combined organic phases were washed with brine and dried over $MgSO_4$. The solvent was removed *in vacuo* and the crude material was purified by flash chromatography eluting 25% EtOAc/pentane to give the pure diol **3** (19.2 mg, 68%, >20:1 *dr*, 99:1 *er*) as a white amorphous solid.

***R**_f* (25%EtOAc/pentane) 0.30. **δ_H** (400 MHz, $CDCl_3$): 1.12 (3H, s), 1.75-1.92 (2H, m), 2.21 (1H, br. s), 2.64 (1H, br. s), 2.67-2.86 (2H, m), 4.59 (1H, s), 7.17 (3H, m), 7.27-7.40 (7H, m). **δ_C** (100 MHz, $CDCl_3$): 21.4 (CH_3), 30.0 (CH_2), 40.9 (CH_2), 75.2 (4° C), 80.0 (CH), 125.9 (CH), 127.7 (CH), 128.0 (CH), 128.2 (CH), 128.5 (CH), 128.6 (CH), 140.5 (4 ° C), 142.5 (4° C). **ν_{max}** (neat): 695, 733, 1024, 1041, 1379, 1454, 1494, 2912, 3026, 3353, 3426. **HRMS** (CI) calc. for $[C_{17}H_{20}O_2 + Na]^+$ 279.1356. Found 279.1353. **Chiral HPLC** (IC with guard, 4% *i*PrOH/hexane, 0.6 ml/min) T_R 26.97 min (minor), 49.07 min (major). $[\alpha]_D^{22}$ −21.5 (*c* 0.65, $CHCl_3$).

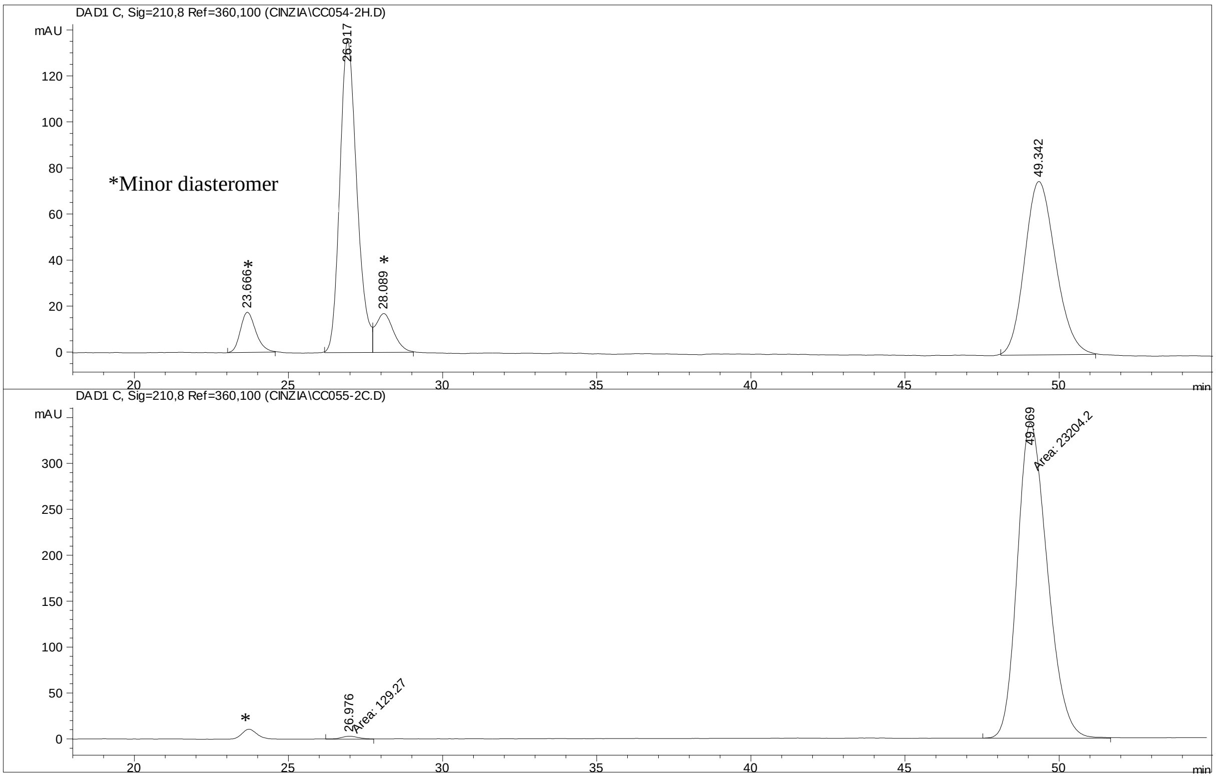

Derivatization of the secondary alcohol 3

(1*R*,2*R*)-2-Hydroxy-2-methyl-1,4-diphenylbutyl (*S*)-3,3,3-trifluoro-2-methoxy-2-phenylpropanoate SI12

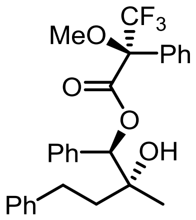

A dry flask was charged with alcohol **3** (1.00 eq, 12.0 mg, 0.05 mmol), CH_2Cl_2 (1 ml) and 4-(dimetheylamino)pyridine (2.00 eq, 11.4 mg, 0.09 mmol) under nitrogen atmosphere. The mixture was cooled to 0 °C, and pyridine (8.00 eq, 30.5 µl, 0.38 mmol) and (*R*)-(−)MTPA-Cl [(R)-(−)-α-Methoxy-α-(trifluoromethyl)phenylacetyl chloride, 4.50 eq, 39.6 µl, 0.21 mmol] were successively added. The

mixture was stirred at ambient temperature overnight. The mixture was diluted with Et_2O (5.00 ml) and washed with 1 M HCl (8.00 eq, 0.38 ml, 0.38 mmol), saturated Na_2CO_3 solution (1.00 ml) and brine. and the combined organic phases were washed with brine and dried over $MgSO_4$. The solvent was removed *in vacuo* and the crude material was purified by flash chromatography eluting 10% EtOAc/pentane to give pure ester **SI12** (12.3 mg, 55%, >20:1 *dr*, 99:1 *er*) as a white amorphous solid.

R_f (10%EtOAc/pentane) 0.33. **δ_H** (500 MHz, $CDCl_3$): 1.13 (3H, s), 1.67 (1H, ddd, *J* 13.8, 11.8, 5.8), 1.72 (1H, ddd, *J* 13.8, 11.8, 5.8), 1.71 (1H, br. s), 2.64 (1H, ddd, *J* 13.8, 11.8, 5.8), 2.74 (1H, ddd, *J* 13.8, 11.8, 5.8), 3.48 (3H, s), 5.88 (1H, s), 7.09-7.11 (2H, m), 7.17-7.21 (1H, m), 7.26-7.31 (4H, m), 7.34-7.36 (4H, m), 7.37-7.40 (2H, m), 7.41-7.43 (2H, m). **δ_C** (125 MHz, $CDCl_3$): 22.0 (CH_3), 29.8 (CH_2), 40.5 (CH_2), 55.7 (CH_3), 74.5 (4° C), 83.4 (CH), 84.7 (4° C, q, *J* 27.3), 123.6 (CF_3, q, *J* 288.5), 126.0 (CH), 127.45 (CH), 127.47 (CH), 128.4 (CH), 128.5 (CH), 128.56 (CH), 128.57 (CH), 128.9 (CH), 129.8 (CH), 132.3 (4° C), 135.7 (4° C), 142.0 (4° C), 165.9 (4° C). **ν_{max}** (neat): 703, 919, 1017, 1174, 1263, 1454, 1500, 1739, 2953, 3424. **HRMS** (CI) calc. for $[C_{27}H_{27}F_3O_4 + Na]^+$ 495.1754. Found 495.1740.

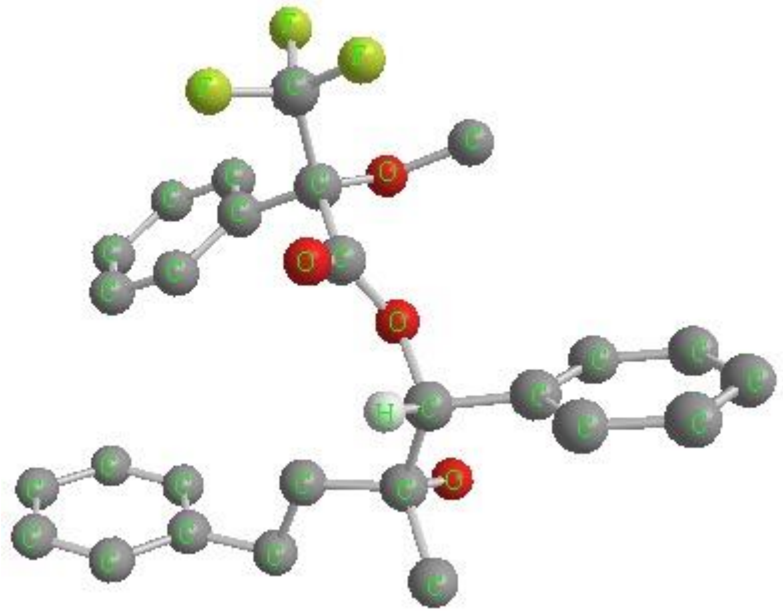

X-ray analysis of **SI12** from ^{i}PrOH/H_2O (CCDC 1557928)

3 EXPERIMENTAL PROCEDURES FOR DIALKYL *S*-THIOBENZOATES 4

Enantiomerically enriched secondary alcohols used below were synthesised via know procedures.[2]

3.1 Synthesis of Dialkyl *S*-Thiobenzoates 4

2,4,6-Triisopropylbenzothioic *S*-acid SI13

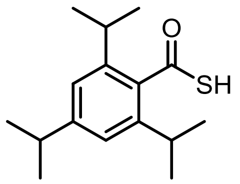

NOTE: Exclusion of oxygen from the reaction mixture and crude product at all stages is crucial to avoid significant formation of the corresponding disulfide of **SI13**. A suspension of Na_2S (3.29 g, 42.2 mmol) and 2,4,6-triisopropylbenzoyl chloride (5.62 g, 21.1 mmol) in THF (45 ml) was heated at reflux overnight (~16 h) under a nitrogen atmosphere. The reaction was allowed to reach ambient temperature and the reaction vessel was connected to a gas trap containing 6 M $NaOH_{(aq)}$. A long needle attached to a nitrogen line was inserted through a septa of the reaction vessel. The reaction vessel was placed under a positive pressure of nitrogen (through the long needle) so as to create a steady stream of bubbles through the NaOH trap. The needle attached to the nitrogen line was positioned below the level of the solvent so as to create a steady stream of nitrogen bubbles though the reaction mixture. The reaction was quenched with the slow addition of 2 M HCl in Et_2O (55 ml) (see Figure S1 below). The reaction was stirred for 1 h while nitrogen was bubbled through the reaction mixture. The solvent was then removed *in vacuo*. Pentane and Et_2O (1:1 v.v, 50 ml) was added to the yellow solid and the suspension was transferred via cannula onto a silica pad under nitrogen and the filtrate collected into a pre-weighed Schenk flask (see Figure S1 below). The reaction flask was washed with a mixture of pentane and Et_2O (1:1 v.v, 2 × 50 ml), transferring the washings onto the silica filter cake and collecting the filtrate into the same Schenk flask as before. The solvent was removed *in vacuo* to give *thiobenzoic acid* **SI13** (5.01 g, 89%) as a pale green solid that contained approximately 5 mol% of the corresponding disulfide **SI14**. Thio acid **SI13** was stored under nitrogen to prevent disulphide formation. ***Thioacid* SI13*:*** δ_H (400 MHz, $CDCl_3$): 1.26 (6H, d, *J* 7.0), 1.27 (12H, d, *J* 7.0), 2.90 (1H, sept, *J* 7.0), 3.16 (2H, sept, *J* 7.0), 7.02 (2H, s). δ_C (100 MHz, $CDCl_3$): 23.9 (CH_3), 24.3 (CH_3), 30.8 (CH), 34.4 (CH), 121.2 (CH), 137.1 (4° C), 143.5 (4° C), 150.7 (4° C), 196.8 (4° C). ν_{max} (neat): 825, 947, 1458, 1604, 1669, 1682, 2869, 2929, 2961. **HRMS** (CI) calc. for $[C_{16}H_{24}OS + H]^+$ 265.1626. Found 265.1622. **LRMS** (CI): 265.2, 231.2 (basepeak).

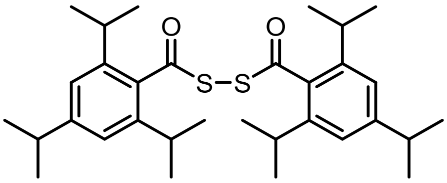

***Disulfide* SI14:** δ_H (400 MHz, $CDCl_3$): 1.27-1.37 (36H, m), 2.94 (2H, sept, *J* 7.0), 3.21 (4H, sept, *J* 6.8), 7.08 (4H, s). δ_C (100 MHz, $CDCl_3$): 23.9 (CH_3), 24.4 (CH_3), 30.7 (CH), 34.5 (CH), 121.3 (CH), 133.1 (4° C), 145.6 (4° C), 151.5 (4° C), 192.4 (4° C). ν_{max} (neat): 865, 1462, 1603, 1717, 2870, 2929, 2962. **HRMS** (ESI) calc. for $[C_{32}H_{46}O_2S_2 + Na]^+$ 549.2816. Found 549.2831.

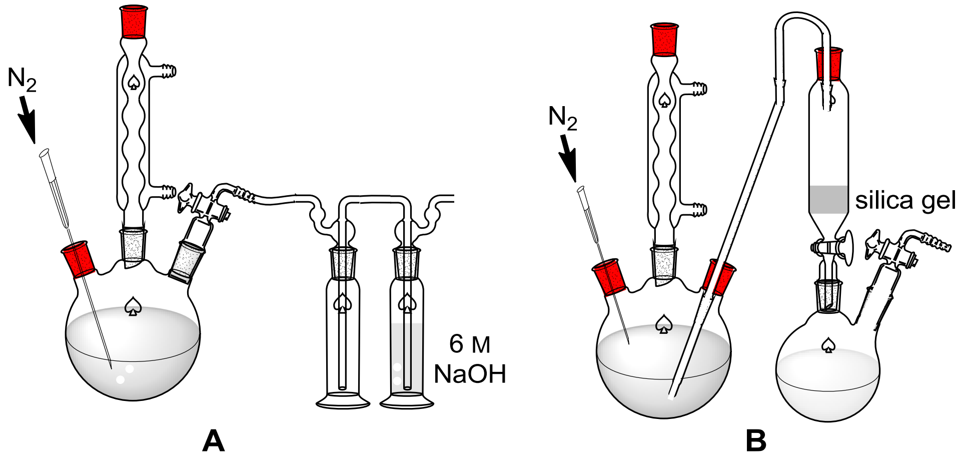

Figure S1. Apparatus and set up for workup during the synthesis of thio acid **SI13**. A: during HCl quench. B: for filtration through silica gel.

General Procedure for the Synthesis of *S*-Thiobenzoates from Secondary Alcohols (GP4)

DIAD (1.10 eq, 325 µl, 1.65 mmol) was added dropwise over 5 min to a stirred solution of PPh_3 (1.20 eq, 472 mg, 1.80 mmol) in THF (8 ml) at 0 °C (ice) under a nitrogen atmosphere. After stirring for 20 min at 0 °C a solution of alcohol (1.20 eq, 1.80 mmol) and 2,4,6-triisopropylbenzothioic *S*-acid **SI13** (1.00 eq, 476 mg, 1.50 mmol; containing ca. 10 mol% of 2,4,6-triisopropylbenzoic dithioperoxyanhydride **SI14**) in THF (3.00 ml) was added and the mixture stirred at 0 °C for 2-5 h. The volatiles were removed *in vacuo* and the residue was triturated with pentane (20 ml). The white suspension was filtered and the filter cake washed with pentane (3 × 20 ml). The solvent was remove *in vacuo* and the residue purified by flash column chromatography eluting 10-30% toluene/pentane to give pure *S*-thiobenzoates.

(*R*)-*S*-(4-Phenylbutan-2-yl) 2,4,6-triisopropylbenzothioate 4a

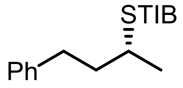

Following **GP4** using (*S*)-4-phenylbutan-2-ol (3.11 g, 20.7 mmol, >99:1 *er*) gave (*R*)-*S*-thiobenzoate **4a** (4.67 g, 63%, 99:1 *er*) as a viscous colourless oil and *O*-thiobenzoate **SI15** (1.34 g, 18%) as a yellow oil. ***S-thiobenzoate* 1a*:*** ***R_f*** (30% toluene/pentane) 0.28. δ_H (400 MHz, $CDCl_3$): 1.27 (18H, br. d, *J* 7.0), 1.48 (3H, d, *J* 7.0), 2.00 (2H, m), 2.72-3.13 (5H, m), 3.85 (1H, app. br. sext., *J* 7.0), 7.03 (2H, s), 7.20-7.25 (3H, m, ArH), 7.29-7.35 (2H, m, ArH). δ_C (100 MHz, $CDCl_3$): 21.5 (CH_3), 23.9 (CH_3), 24.5 (br. s, CH_3), 30.6 (CH), 33.4 (CH_2), 34.4 (CH), 38.1 (CH_2), 40.1 (CH), 121.0 (CH), 126.0 (CH), 128.4 (CH), 128.5 (CH), 135.8 (4° C), 141.6 (4° C), 144.4 (4° C), 150.3 (4° C), 198.1 (4° C). ν_{max} (neat): 698, 895, 1206, 1456, 1657, 1675, 2868, 2927, 2960. **Elemental analysis**: calc. for $C_{26}H_{36}OS$: C, 78.73; H, 9.15; Found: C, 78.25; H, 9.00. **HRMS** (CI) calc. for $[C_{26}H_{36}OS + H]^+$ 397.2565. Found 397.2569. **LRMS** (CI): 397.3, 231.2 (basepeak). **Chiral HPLC** (IB, 0.1% *i*PrOH/hexane, 0.35 ml/min, 0 °C) T_R 16.0 min (minor), 16.8 (major). $[\alpha]_D^{25}$ −22.0 (*c* 3.0, $CHCl_3$).

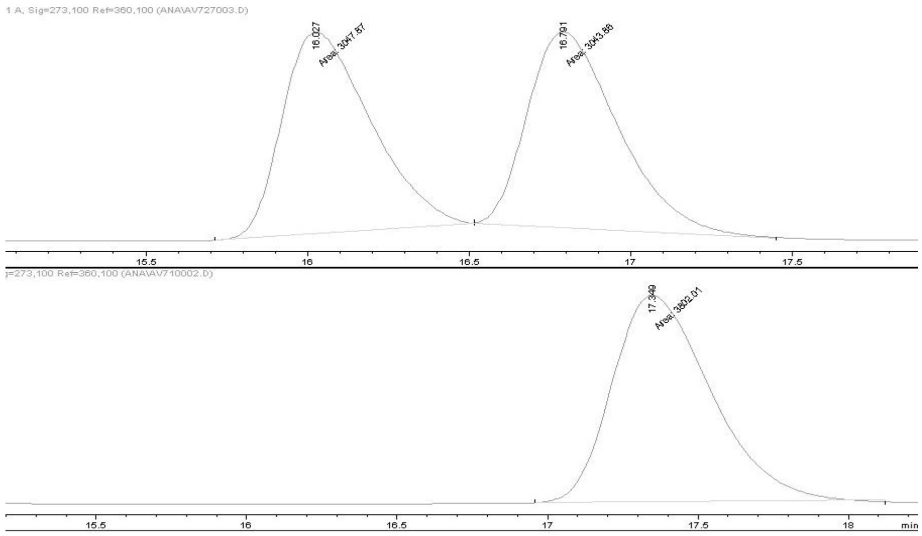

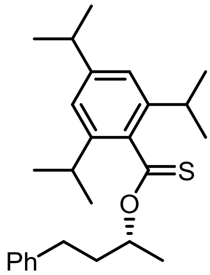

O-thiobenzoate **SI15**: ***R_f*** (30% toluene/pentane) 0.65. δ_H (400 MHz, $CDCl_3$): 1.23-1.30 (18H, m), 1.49 (3H, d, *J* 6.2), 1.96-2.06 (1H, m), 2.16-2.26 (1H, m), 2.67-2.85 (2H, m), 2.90 (1H, sept., *J* 7.0), 3.01 (1H, sept., *J* 7.0), 3.13 (1H, sept., *J* 7.0), 5.90 (1H, app. br. sext., *J* 6.2), 7.01 (1H, s), 7.02 (1H, s),

7.20-7.25 (3H, m), 7.29-7.35 (2H, m). **δ*C*** (100 MHz, $CDCl_3$): 18.8 (CH_3), 23.8 (CH_3), 24.0 (CH_3), 24.3 (CH_3), 24.4 (CH_3), 24.5 (CH), 30.4 (CH), 30.8 (CH), 31.7 (CH_2), 34.3 (CH), 37.3 (CH_2), 78.6 (CH), 120.98 (CH), 121.03 (CH), 126.0 (CH), 128.3 (CH), 128.5 (CH), 138.8 (4 °C), 141.3 (4 °C), 143.3 (4 °C), 143.4 (4 °C), 149.0 (4 °C), 218.9 (4 °C). **ν_{max}** (neat): 696, 1223, 1457, 1605, 2868, 2927, 2959. **HRMS** (CI) calc. for $[C_{26}H_{36}OS + H]^+$ 397.2565. Found 397.2551. **LRMS** (CI): 397.3, 265.2 (basepeak), 231.2. $[\alpha]_D^{25}$ +9.8 (*c* 3.06, $CHCl_3$).

(*R*)-*S*-(1-Phenylpentan-3-yl) 2,4,6-triisopropylbenzothioate 4b

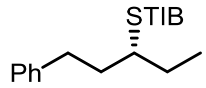

Following **GP4** using (*S*)-1-phenylpentan-3-ol (295 mg, 1.80 mmol, 99:1 *er*) gave (*R*)-*S*-thiobenzoate **4b** (416 mg, 54%, 99:1 *er*) as a colourless oil.

R_f (25% toluene/pentane). **δ*H*** (400 MHz, $CDCl_3$): 1.04 (3H, t, J 7.1), 1.25 (18H, d, J 6.9), 1.60-1.86 (2H, m), 1.86-2.10 (2H, m), 1.57- 1.70 (2H, m), 2.66-2.87 (2H, m), 2.89 (1H, app. sept, J 6.9), 3.05 (2H, app. sept, J 6.9), 3.76 (1H, m), 7.01 (2H, s), 7.16-7.23 (3H, m), 7.27-7.33 (2H, m). **δ*C*** (100 MHz, $CDCl_3$): 11.4 (CH_3), 24.1 (CH_3), 24.6 (CH_3), 28.2 (CH_2), 30.9 (CH_3), 33.5 (CH_2), 34.5 (CH), 36.6 (CH_2), 47.0 (CH), 121.2 (CH), 126.1 (CH), 128.5 (CH), 128.6 (CH), 136.2 (4° C), 141.9 (4° C), 144.6 (4° C), 150.4 (4° C), 198.3 (4° C). **ν_{max}** (neat): 696, 897, 1210, 1455, 1653, 1678, 2865, 2929, 2957 . **HRMS** (ESI) calc. for $[C_{27}H_{38}OS + Na]^+$ 433.2536. Found 433.2521. **Chiral HPLC**: (IB with guard, 0.1% IPA/hexane, 0.7 ml/min, 0 °C) T_R 12.35 min (major), 12.91 min (minor). $[\alpha]_D^{22}$ –12.2 (c 1.0, $CHCl_3$).

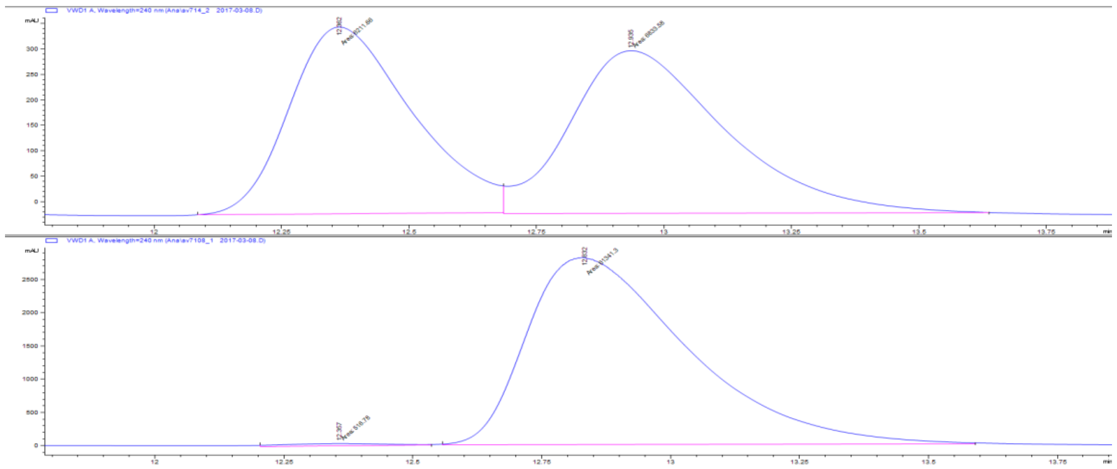

(*R*)-*S*-(Pentan-2-yl) 2,4,6-triisopropylbenzothioate 4c

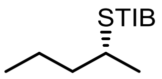

Following a **GP4** using (*S*)-2-pentanol (196 µl, 1.80 mmol, 99:1 *er*) gave a mixture of (*R*)-secondary *S*-thiobenzoate **4c** and disulfide **SI14** (338 mg, 60% spectroscopic yield of *S*-thiobenzoate **4c**) as a colourless oil. To remove the disulfide: The mixture of *S*-thiobenzoate **4c** and disulfide **SI14** was dissolved in MeOH (12.0 ml) and treated with $NaBH_4$ (489 mg, 13.0 mmol). The reaction was stirred at room temperature for 2 h. The solvent was removed *in vacuo* and the crude material was purified by flash column chromatography under a nitrogen atmosphere, eluting 2% Et_2O/pentane to give the pure (*R*)-secondary *S*-benzothioate 4c (197 mg, 40% over 2 steps, 99:1 *er*).

$\boldsymbol{R_f}$ (2% Et_2O/pentane) 0.33. $\boldsymbol{\delta_H}$ (400 MHz, $CDCl_3$): 0.96 (3H, t, *J* 7.3), 1.25 (18H, d, *J* 6.9), 1.40 (3H, d, *J* 6.9), 1.43-1.53 (2H, m), 1.57-1.70 (2H, m), 2.88 (1H, sept, *J* 6.9), 3.01 (2H, sept, *J* 6.9), 3.80 (1H, sext, *J* 6.9), 6.99 (2H, s). $\boldsymbol{\delta_C}$ (100 MHz, $CDCl_3$): 14.0 (CH_3), 20.4 (CH_2), 21.5 (CH_3), 24.1 (CH_3), 24.6 (CH_3), 30.7 (CH), 34.5 (CH), 38.5 (CH_2), 40.2 (CH), 121.1 (4° C), 136.1 (4° C), 144.6 (4° C), 150.4 (4° C), 198.5 (4° C). $\boldsymbol{\nu_{max}}$ (neat): 766, 877, 895, 943, 1205, 1460, 1658, 1676, 2959. **HRMS** (CI) calc. for $[C_{21}H_{34}OS + Na]^+$ 357.2223. Found 357.2216. **Chiral HPLC** (IB with guard, neat hexane, 0.3 ml/min, 0 °C) T_R 15.35 min (major), 17.08 min (minor). $[\alpha]_D^{22}$ –14.9 (*c* 0.34, $CHCl_3$).

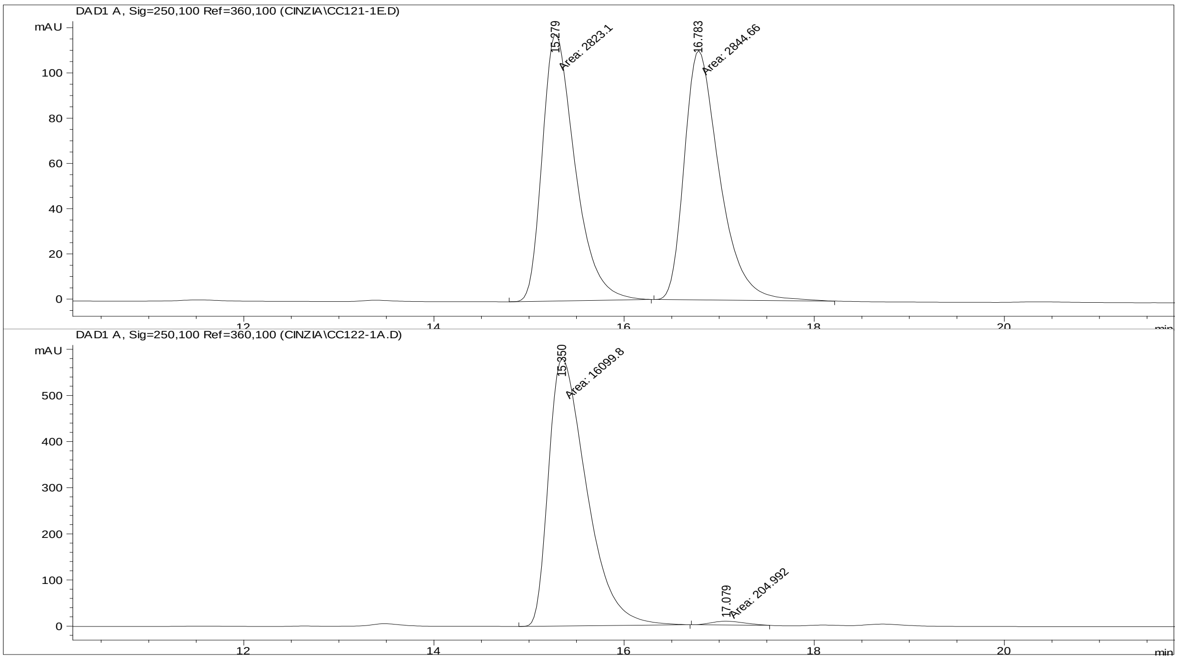

(*R*)-*S*-(Hex-5-en-2-yl) 2,4,6-triisopropylbenzothioate 4d

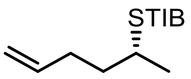

Following **GP4** using (*S*)-hex-5-en-2-ol (180 mg, 1.80 mmol, 99:1 *er**) gave (*R*)-*S*-thiobenzoate **4d** (370 mg, 60%, 99:1 *er*) as a colourless oil.

Rf (25% toluene/pentane) 0.33. $\boldsymbol{\delta_H}$ (400 MHz, $CDCl_3$): 1.26 (18H, d, J 6.9), 1.43 (3H, d, J 7.2), 1.70-1.82 (2H, m), 2.19-2.26 (2H, m), 2.90 (1H, sept, J 6.9), 3.02 (2H, sept, J 6.9), 3.81 (1H, sext, J 6.9), 5.02 (1H, ddt, J 10.1, 1.9, 1.2), 5.09 (1H, ddt, J 16.9, 1.9), 5.85 (1H, ddt, J 16.9, 10.1, 6.7) 7.00 (2H, s). $\boldsymbol{\delta_C}$ (100 MHz, $CDCl_3$): 21.3 (CH_3), 23.9 (CH_3), 24.4 (CH_3), 30.6 (CH), 31.3 (CH_2), 34.4 (CH), 35.4 (CH_2), 39.8 (CH), 115.2 (CH_2), 121.0 (CH), 135.8 (4° C), 137.7 (CH), 144.4 (4° C), 150.3 (4° C), 198.2 (4° C). $\boldsymbol{\nu_{max}}$ (neat): 766, 876, 896, 944, 1210, 1460, 1658, 1677, 2959. **HRMS** (ESI) calc. for $[C_{22}H_{34}OS + Na]^+$ 369.2223. Found 369.2216. $[\alpha]_D^{22}$ –18.3 (c 1.0, $CHCl_3$). *Determined after ozonolysis, see below.

(*R*)-S-(5-Hydroxypentan-2-yl) 2,4,6-triisopropylbenzothioate (SI16)

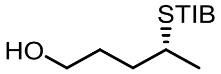

Ozone was bubbled through a solution of thiobenzoate **4d** (50 mg, 0.14 mmol, 1 eq.) in CH_2Cl_2 (2 mL) at −78 °C for less than 10 min. The reaction mixture was degassed at −78 °C with bubbling nitrogen. The ozonide was cleaved through addition of $NaBH_4$ (55 mg, 1.4 mmol, 10 eq.) at 0 °C. The CH_2Cl_2 was removed in vacuo and the residue dissolved in water (25 mL), the aqueous phase was washed with Et_2O (3 × 25 mL), dried over $MgSO_4$ filtered and concentrated in vacuo to give alcohol **SI16** as an oil (49 mg, 85%, >99:1 *er*) which was used without further purification.

Rf (CH_2Cl_2) 0.20. $\boldsymbol{\delta_H}$ (400 MHz, $CDCl_3$): 1.25 (18H, d, *J* 6.8), 1.37 (3H, d, *J* 6.3), 1.48 – 1.86 (4H, m), 2.80-2.93 (3H, m), 3.68 (2H, m), 3.81 (1H, sext, *J* 6.6), 7.00 (2H, s). $\boldsymbol{\delta_C}$ (100 MHz, $CDCl_3$): 21.0 (CH_3), 23.9 (CH_3), 24.3 (CH_3), 24.4 (CH_3), 30.1 (CH_2), 30.6 (CH), 32.6 (CH_2), 34.4 (CH), 39.9 (CH_2), 62.4 (CH), 120.8 (CH), 130.7 (4° C), 144.5 (4° C), 150.0 (4° C), 198.2 (4° C). $\boldsymbol{\nu_{max}}$ (neat): 766, 876, 896, 944, 1210, 1460, 1658, 1677, 2959, 3411. **HRMS (ESI):** calc. for $[C_{21}H_{34}O_2S + Na]^+$ 350.2342. Found 350.2339 **Chiral SFC:** Chiracel IA, iso 5% (10% IPA:hexane), 4 mL/min, 100 bar, 40 °C, 254 nm, T_R = 4.12 min (major) and 4.76 min (minor). $[\alpha]_D^{22}$. –9 (c 1.0, $CHCl_3$).

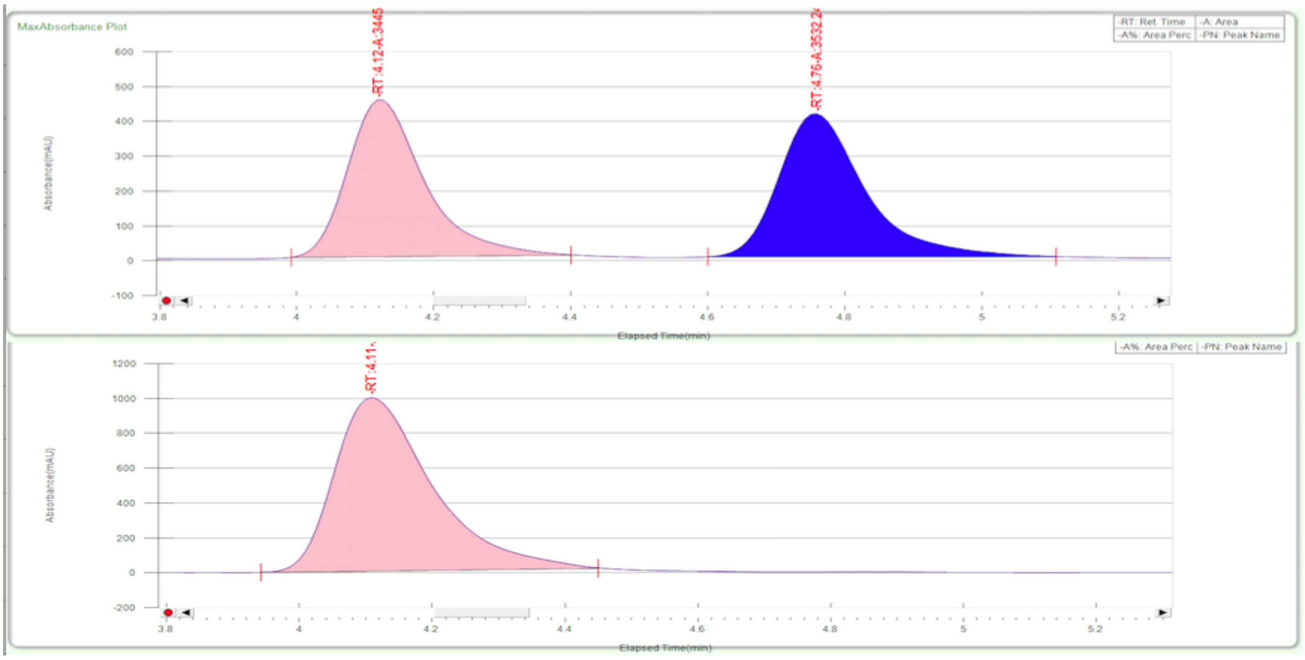

***S*-((2*R*)-5-((Tetrahydro-2H-pyran-2-yl)oxy)pentan-2-yl) 2,4,6-triisopropylbenzothioate 4e**

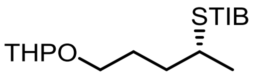

A catalytic amount of PTSA was added to a solution of alcohol **SI16** (40 mg, 0.11 mmol) and dihydropyran (1.10 mL, 0.28 mmol) in dichloromethane (5 mL) and stirred at RT overnight. The reaction mixture was quenched though addition of water (2 mL) and the aqueous layer extracted with CH_2Cl_2 (3 x 5 mL). The organics were combined, dried ($MgSO_4$) and concentrated in vacuo. Purification by column chromatography eluting CH_2Cl_2 gave **4e** as an oil (70%, 35 mg, >99:1 *er*, 1:1 *dr*).

$\boldsymbol{R_f}$ (CH_2Cl_2) 0.58. $\boldsymbol{\delta_H}$ (400 MHz, $CDCl_3$): 1.26 (18H, d, *J* 6.8), 1.38 (3H, d, *J* 6.2), 1.43 - 1.93 (10H, m), 2.80 - 2.98 (3H, m), 3.35 - 3.57 (2H, m), 3.70 - 3.93 (3H, m), 4.60 (1H, br. s), 7.01 (2H, s). $\boldsymbol{\delta_C}$ (100 MHz, $CDCl_3$): (100 MHz, $CDCl_3$): 19.5 (CH_2), 19.8 (CH_3), 24.0 (CH_3), 24.0 (CH_3), 24.3 (CH_3), 25.5 (CH_2), 25.7 (CH_2), 25.8 (CH_2), 30.7 (CH_2), 30.7 (CH_2), 31.3 (CH_3), 32.7 (CH_2), 39.4 (CH), 62.2 (CH_2), 67.1 (CH_2), 67.1 (CH_2), 71.7 (CH), 71.7 (CH), 98.7 (CH), 98.7 (CH), 120.8 (CH), 130.9 (4° C), 144.5 (4° C), 149.9 (4° C), 198.3 (4° C). $\boldsymbol{\nu_{max}}$ (neat): 766, 877, 895, 943, 1205, 1460, 1658, 1676, 2959. **HRMS** (ESI) calc. for $[C_{26}H_{42}O_3S + Na]^+$ 457.2477. Found 457.2474. $[\alpha]_D^{22}$ –15.

***S*-((3*R*,8*S*,9*S*,10*R*,13*R*,14*S*,17*R*)-10,13-Dimethyl-17-((*R*)-6-methylheptan-2-yl)-2,3,4,7,8,9,10,11,12,13,14,15,16,17-tetradecahydro-1*H*-cyclopenta[a]phenanthren-3-yl) 2,4,6-triisopropylbenzothioate 8**

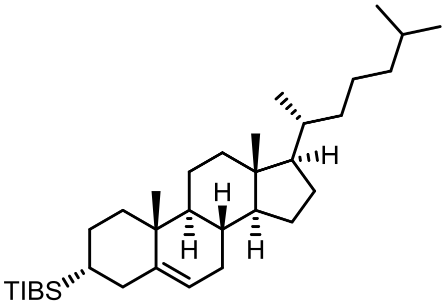

Following **GP4** using cholesterol (1.0 g, 2.59 mmol) gave (*R*)-*S*-thiobenzoate **8** (540 mg, 32%) as a white foam.

R R_f (pentane:toluene 80:20) 0.45. δ_H (400 MHz, $CDCl_3$): 0.67 (3H, s), 0.87 (6H, dd, *J* 6.7, 1.6 Hz), 0.92 (3H, d, *J* 6.7 Hz), 0.99 (3H, m), 1.03 (3H, s), 1.11 (4H, m), 1.24 (20H, m), 1.34 (4H, m), 1.53 (4H, m), 1.72 (1H, m), 1.83 (2H, m), 1.99 (2H, m), 2.09 (2H, m), 2.25 (1H, m), 2.85 (2H, m), 2.99 (2H, m), 4.21 (1H, m), 5.31 (1H, m), 6.97 (2H, s). δ_C (100 MHz, $CDCl_3$): 11.9 (CH_3), 18.7 (CH_3), 19.3 (CH_3), 20.7 (CH_2), 22.6 (CH_3), 22.8 (CH_3), 23.8 (CH_3), 24.0 (2 x CH_3), 24.4 (4 x CH_3), 27.3 (CH_2), 28.0 (CH), 28.2 (CH_2), 30.6 (2 x CH), 31.7 (CH), 31.9 (CH_2), 34.4 (CH), 35.7 (4° C), 35.8 (CH_2), 36.2 (CH_2), 37.2 (CH_2), 37.6 (CH_2), 39.5 (CH_2), 39.7 (CH_2), 42.2 (4° C), 43.9 (CH), 50.2 (CH), 56.1 (CH), 56.7 (CH), 121.0 (CH), 122.8 (CH), 136.1 (4° C), 139.0 (2 x 4° C), 144.4 (4° C), 150.2 (4° C), 198.2 (C=O). ν_{max} (neat): 2958, 1676, 1460, 1206, 896. **HRMS** (ESI) calc. for $[C_{43}H_{68}OS + Na]^+$ 655.4883. Found 655.4853. $[\alpha]_D^{22}$ +20 (*c* 1.0, $CHCl_3$).

3.2 Optimisation of the Lithiation Conditions of 4

General Procedure for Lithiation/Deuteration of *S*-Thiobenzoates 4a (GP5)

*s*BuLi (1.3 M in hexane, 1.20 eq, 115 µl, 0.150 mmol) was added dropwise over 5 min to a vigorously stirred solution (without splashing) of *S*-thiobenzoate **4a** (1.00 eq, 50.0 mg, 0.125 mmol) and the additive (1.20 eq, 0.30 mmol) in anhydrous solvent (0.75 ml) at –78 °C under a nitrogen atmosphere. After the specified time, a solution of the MeOD (2.00 eq, 0.50 mmol) in the specified solvent (0.1 ml) was added dropwise over 2 min. The reaction mixture was stirred at –78 °C for 1 h and then the cooling bath was removed. The reaction was allowed to reach room temperature and the mixture was passed through a silica plug in a Pasteur pipette, washing the silica with Et_2O (5 ml). The solvent was removed

in vacuo and the crude mixture, containing (*R*)-**4a**-D and (*R*)-**4a** was analysed by ^{1}H-NMR and Chiral HPLC.

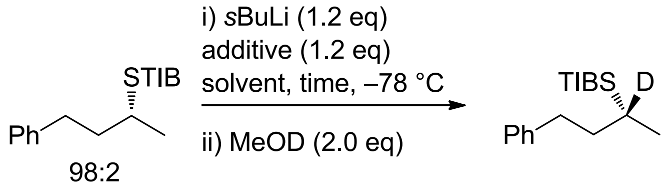

Entry	Solvent	Lithiation time (min)	Additive	%D[a]	*er*[b]
1	THF	15	TMEDA	88	56:44
2	Et_2O	15	-	11	97:3
3	Et_2O	15	TMEDA	100	90:10
4	Et_2O	60	TMEDA	100	84:16
5	CPME	15	TMEDA	100	94:6
6	CPME	60	TMEDA	100	92:8
7	TBME	5	TMEDA	100	97:3
8	TBME	15	TMEDA	100	96:4
9	TBME	60	TMEDA	100	93:7
10	Toluene	15	TMEDA	78	94:6
11	Toluene	60	TMEDA	85	95:5
12	Et_2O/hexane (1:10)	15	TMEDA	100	94:6
13	Et_2O/hexane (1:10)	60	TMEDA	100	92:8
14	TBME	15	(−)-sparteine	7	98:2
15	TBME	15	TMCDA	58	97:3
16	TBME	15	PMDTA	6	ND
17	TBME	15	DME	14	93:7

Yield of **4a** and **4a**-D was ≥90% in all cases. [a]Determined by ^{1}H-NMR. [b]Determined by HPLC on the crude mixture of **4a** and **4a**-D.
TMEDA = *N*,*N*,*N'*,*N'*-tetramethylethylene diamine; TMCDA = (*rac*,*trans*)-*N*,*N*,*N'*,*N'*-tetramethylcyclohexane-1,2-diamine; PMDTA = *N*,*N*,*N'*,*N'*,*N''*-pentamethyldiethylenetriamine; DME = dimethoxyethane.

3.3 Lithiation and Electrophilic Trapping of Dialkyl *S*-Thiobenzoates 4

General Procedure for Lithiation/Electrophilic Trapping of Dialkyl *S*-Thiobenzoates 4 (GP6)

*s*BuLi (1.3 M in hexane, 1.20 eq, 230 µl, 0.30 mmol) was added dropwise over 2 min to a vigorously stirred solution (without splashing) of *S*-thiobenzoate **4** (1.00 eq, 0.250 mmol) and TMEDA (1.20 eq,

0.30 mmol, 35 µl) in anhydrous TBME (1.5 ml) at –78 °C under a nitrogen atmosphere. After the specified time, a solution of the electrophile (2.00 eq, 0.50 mmol) in TBME (0.25 ml) was added dropwise over 2 min. The reaction mixture was stirred at –78 °C for 1 h and then the cooling bath was removed. The reaction was allowed to reach room temperature and was quenched with H_2O (2 ml) or sat. $NH_4Cl_{(aq)}$ (2 ml). The phases were separated and the aqueous phase was extracted with Et_2O (4 × 15 ml). The combined organic phases were washed with brine and dried over $MgSO_4$. The solvent was removed *in vacuo* and the crude material purified by flash chromatography to give pure *S*-benzothioate.

Methyl (*R*)-2-methyl-4-phenyl-2-((2,4,6-triisopropylbenzoyl)thio)butanoate 6aa

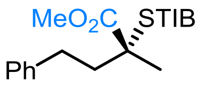

Following **GP6** using (*R*)-*S*-benzothioate **4a** (41 mg, 0.12 mmol, 99:1 *er*) and methyl chloroformate (57 µl, 0.74 mmol) and purification by flash chromatography (5% Et_2O/pentane) gave thiobenzoate **6aa** (42 mg, 77%, 96:4 *er*) as a colourless oil.

***R**f* (5% Et_2O/pentane) 0.42. **δ_H** (400 MHz, $CDCl_3$): 1.25 (18H, d, *J* 6.9), 1.81 (3H, s), 2.25 (2H, m), 2.67 (1H, ddd, *J* 13.5, 10.2, 6.9), 2.75 (1H, ddd, *J* 13.5, 10.2, 7.1), 2.89 (1H, hept., *J* 6.9), 3.11 (2H, hept., *J* 6.9), 3.79 (3H, s), 7.00 (2H, s), 7.15 – 7.22 (3H, m) 7.29 (2H, m). **δ_C** (100 MHz, $CDCl_3$): 23.5 (CH_3), 23.9 (CH_3), 30.3 (CH), 31.2 (CH_2), 34.4 (CH), 39.9 (CH_2), 52.7 (4 °C), 55.9 (CH), 121.0 (CH), 126.1 (CH), 128.3 (CH), 128.5 (CH), 134.50 (4 °C), 141.0 (4 °C), 144.9 (4 °C), 150.8 (4 °C), 173.5 (4 °C), 197.1 (4 °C). **ν_{max}** (neat): 1459 (Ar), 1678 ($C{=}O_{TIB}$), 1739 ($C{=}O_{ester}$). **HRMS (ESI)** calc. for $[C_{28}H_{38}O_3S + Na]^+$ 477.2434. Found 477.2441. **Chiral HPLC** (IA, 2% *i*PrOH/hexane, 0.5 ml/min) T_R 9.42 min (minor), 313.71 (major). $[\alpha]_D^{22}$ +11 (*c* 1.0, $CHCl_3$).

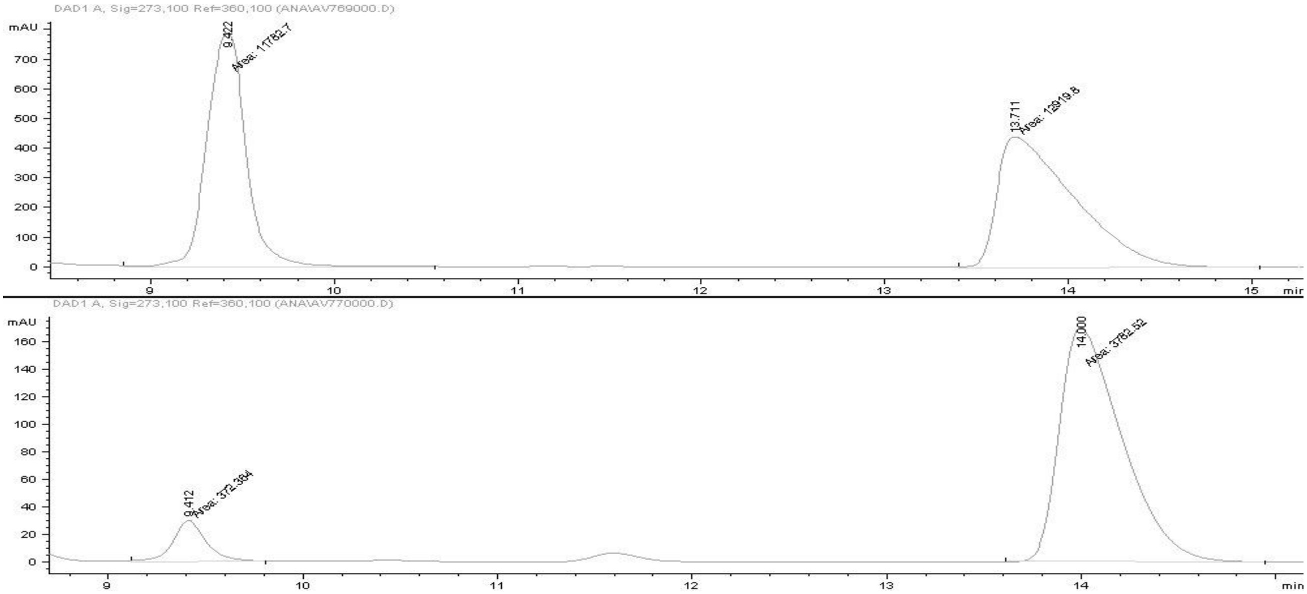

(*R*)-S-(1-((4-Bromophenyl)amino)-2-methyl-1-oxo-4-phenylbutan-2-yl)2,4,6 triisopropylbenzothioate 6ab

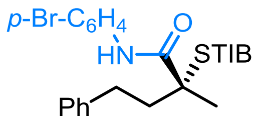

Following a **GP6** using (*R*)-*S*-benzothioate **4a** (50 mg, 0.13 mmol, >99:1 *er*) and 4-Bromophenyl isocyanate (50 mg, 0.26 mmol) and purification by flash chromatography (2% Et_2O/pentane) gave thiobenzoate **6ab** (60 mg, 80%, 98:2 *er*) as a white solid.

R_f (2% Et_2O/pentane) 0.40. **δ_H** (400 MHz, $CDCl_3$): 1.17 (12H, d, *J* 7.0), 1.24 (6H, d, *J* 7.0), 9.18 (1H, s, NH), 7.51-7.43 (4H, m, ArH), 7.33 – 7.27 (2H, m, ArH), 7.24 – 7.18 (3H, m, ArH), 7.00 (2H, s, ArH), 2.89 (3H, hept., *J* 7.0. ArCH), 2.78 (1H, m, $ArCH_AH_B$), 2.74 (1H, ddd, *J* 13.3, 11.2, 5.9, $ArCH_AH_B$), 2.79 – 2.68 (2H, m, $ArCH_2CH_2$), 1.82 (3H, s, CCH_3),. **δ_C** (100 MHz, $CDCl_3$): 198.2 (4° C), 172.3 (4° C), 151.1 (4° C), 144.6 (4° C), 141.0 (4° C), 138.1 (4° C), 134.4 (4° C), 128.8 (CH), 128.5 (CH), 128.4(CH), 127.9 (CH), 127.5 (CH), 126.1 (CH), 121.1 (CH), 57.7 (4° C), 44.4 (CH_2), 40.3 (CH_2), 34.4 (CH), 31.1 (CH), 30.7 (CH_2), 23.9 (CH_3), 22.7 (CH_3). **ν_{max}** (neat): 2960 (Ar), 1680 ($C=O_{CONH}$), 1675 ($C=O_{TIB}$), 1489, 896. **HRMS (EI)** calc. for $[C_{33}H_{40}O_2NSBr]^+$ 593.1963. Found 593.1971. **Chiral HPLC** (IB, 2% *i*PrOH/hexane, 0.5 ml/min, rt) T_R 13.7 min (minor), 15.6 (major). $[\alpha]_D^{22}$ +5 (*c* 1.0, $CHCl_3$).

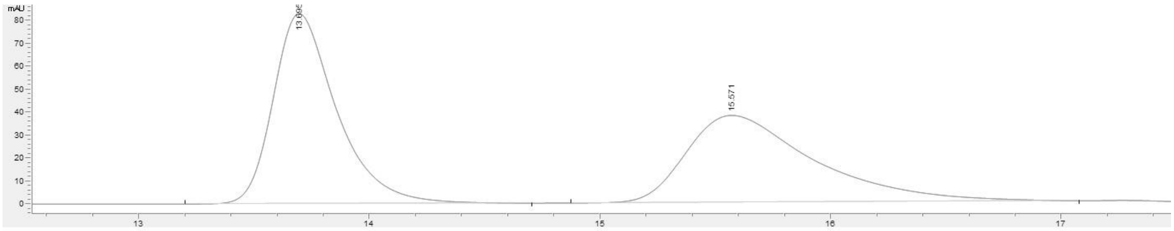

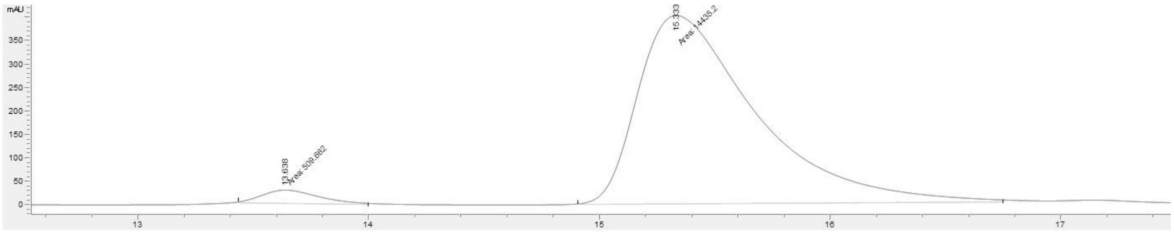

(*R*)-S-(2-Methyl-1-oxo-4-phenylbutan-2-yl) 2,4,6-triisopropylbenzothioate 6ac

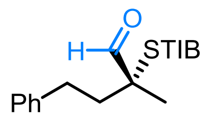

Following a **GP6** using (*R*)-*S*-benzothioate **4a** (99 mg, 0.25 mmol, >99:1 *er*) and *N*,*N*-dimethyl formamide (39 µl, 0.5 mmol) and purification by flash chromatography (5% Et_2O/ pentane) gave thiobenzoate **6ac** (76 mg, 72%, 94:6 *er*) as an oil.

δ_H (400 MHz, $CDCl_3$): 1.25 (18H, br. d., *J* = 6.7), 1.64 (3H, s), 2.14 (1H, ddd, *J* = 14.3, 11.8, 5.3), 2.25 (1H, ddd, *J* 14.3, 11.8, 5.3), 2.69 (1H, td, *J* = 12.9, 5.3), 2.77 (1H, td, *J* = 12.9, 5.3), 2.89 (1H, hept., *J* = 6.9), 3.05 (2H, hept., *J* = 6.9), 7.01 (2H, s), 7.15 – 7.24 (3H, m), 7.27 – 7.33 (2H, m), 9.69 (1H, s). **δ_C** (100 MHz, $CDCl_3$): 19.6 (CH_3), 24.0 (CH_3), 24.6 (CH_3), 30.6 (CH_2), 30.9 (CH), 34.6 (CH), 36.7 (CH_2), 60.7 (4° C), 121.3 (CH), 126.5 (CH), 128.4 (CH), 128.7 (CH), 134.1 (4° C), 141.0 (4° C), 144.8 (4° C), 151.2 (4° C), 197.1 (4° C), 198.4 (CH). **ν_{max}** (neat): 1456 (Ar), 1654 (Ar), 1675 ($C{=}O_{TIB}$), 1727 ($C{=}O_{ALD}$). **HRMS (ESI)** calc. for $[C_{27}H_{36}O_2S + Na]^+$ 447.2328. Found 447.2327. **Chiral HPLC** (IC, 4% *i*PrOH/hexane, 0.5 mL/min, T_R 9.25 min (major), 13.86 min (minor). $[\alpha]_D^{22}$ +11.3 (*c* 1.0, $CHCl_3$).

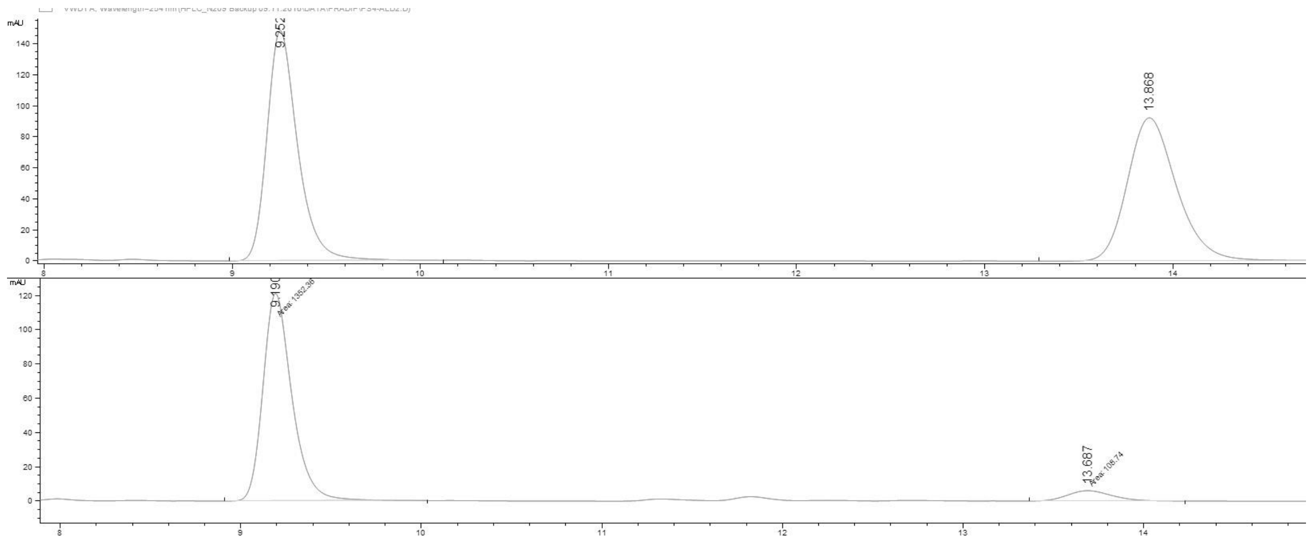

(*R*)-(1-Hydroxy-2-methyl-1,4-diphenylbutan-2-yl) 2,4,6-triisopropylbenzothioate 6ad

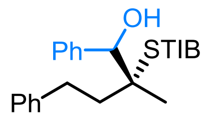

Following a **GP6** using (*R*)-*S*-benzothioate **4a** (99 mg, 0.25 mmol, >99:1 *er*) and benzaldehyde (50 µl, 0.50 mmol) and purification by flash chromatography (10% EtOAc/petroleum ether) gave thiobenzoate **6ad** (80 mg, 64%, 1:1.5 *dr*) as an oil. Further purification by preparative HPLC (5%

EtOAc/hexane) gave diastereomer A (22 mg, 23%, 98:2 *er*) as an oil and diastereomer B (17 mg, 18%, 98:2 *er*) as an amorphous solid.

Diastereomer A: $\boldsymbol{R_f}$ (10% EtOAc/petroleum ether) 0.34. $\boldsymbol{\delta_H}$ (400 MHz, $CDCl_3$): 1.24 – 1.35 (18H, m), 1.36 (3H, s), 1.97 (1H, td, *J* 11.4, 3.3), 2.73 (2H, m), 2.99 (2H, m), 3.16 (2H, hept., *J* 6.8), 3.87 (1H, br. d, *J* 4.0), 5.30 (1H, br. d, *J* 4.0), 7.01 (2H, s), 7.15 – 7.22 (3H, m), 7.25 – 7.36 (5H, m), 7.43 (2H, m, ArH). $\boldsymbol{\delta_C}$ (100 MHz, $CDCl_3$): 19.8 (CH_3), 24.0 (CH_3), 31.0 (CH), 31.5 (CH_2), 34.5 (CH), 38.5 (CH_2), 64.0 (4° C), 78.8 (CH), 121.3 (CH), 126.1 (CH), 127.9 (CH), 128.0 (CH), 128.2 (CH), 128.5 (CH), 128.6 (CH), 135.2 (4° C), 139.9 (4° C), 141.8 (4° C), 144.7 (4° C), 150.9 (4° C), 200.8 (C=O). $\boldsymbol{\nu_{max}}$ (neat): 1454 (Ar), 1649 (Ar), 1679 ($C{=}O_{TIB}$), 3400 (OH). **HRMS (ESI)** calc. for $[C_{33}H_{42}O_2S + Na]^+$ 525.2798. Found 525.2792. **Chiral SFC** (IB, 4% MeOH, 4 ml/min, 125 bar, rt) T_R 6.28 min (major), 6.82 (minor). $[\alpha]_D^{22}$ +3 (*c* 0.5, $CHCl_3$).

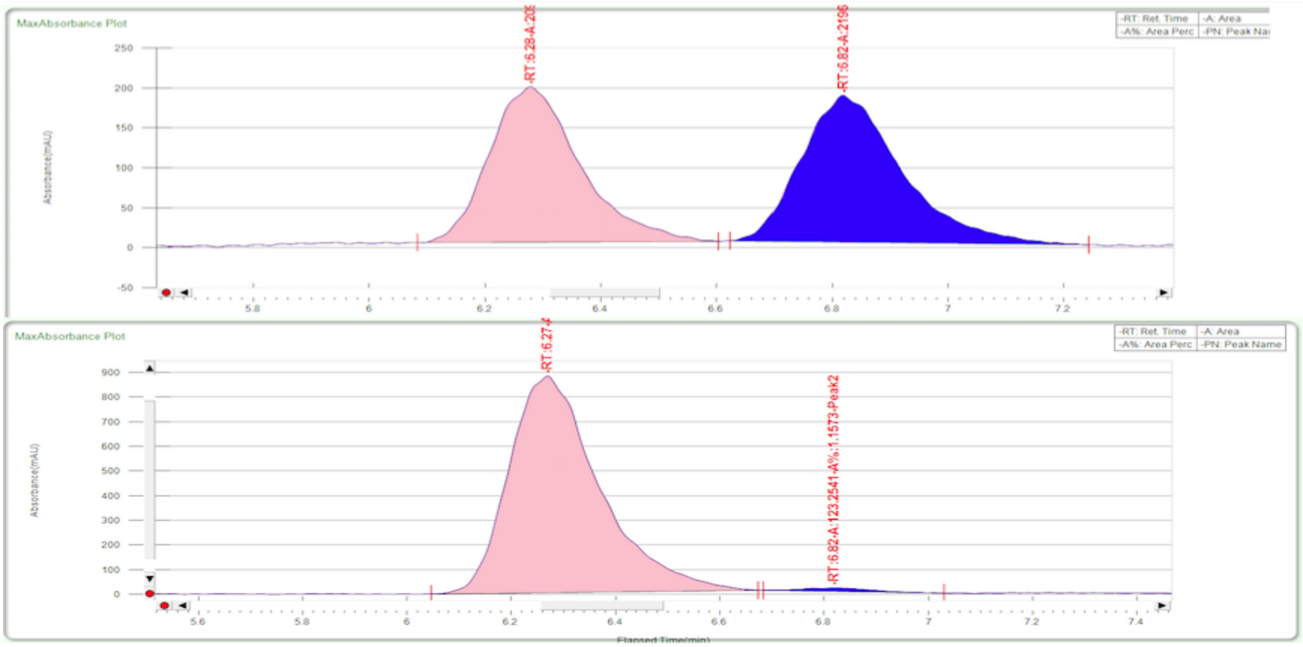

Diastereomer B: $\boldsymbol{R_f}$ (10% EtOAc/petroleum ether) 0.34. $\boldsymbol{\delta_H}$ (400 MHz, $CDCl_3$): 1.20 – 1.38 (18H, m), 1.66 (3H, s), 1.95 (1H, td, *J* 13.0, 5.2), 2.30 (1H, td, *J* 13.0, 4.4), 2.70 (1H, td, *J* 12.8, 5.2), 2.83 (1H, td, *J* 12.8, 4.4), 2.90 (1H, sept, *J* 6.8), 3.15 (2H, sept, *J* 6.8), 4.04 (1H, br. d, *J* 3.7), 5.29 (1H, br. d, *J* 3.7), 7.02 (2H, s), 7.12 – 7.22 (3H, m), 7.28 – 7.36 (5H, m), 7.44 (2H, m). $\boldsymbol{\delta_C}$ (100 MHz, $CDCl_3$): 22.3 (CH_3), 24.0 (CH_3), 31.1 (CH), 34.5 (CH_2), 36.5 (CH_2), 63.3 (4° C), 80.3 (CH), 121.2 (CH), 126.2 (CH), 127.8 (CH), 128.0 (CH), 128.2 (CH), 128.5 (CH), 128.6 (CH), 135.3 (4° C), 140.1 (4° C), 141.7 (4° C), 144.7 (4° C), 150.9 (4° C), 201.2 ($C{=}O_{TIB}$). $\boldsymbol{\nu_{max}}$ (neat): 1454 (Ar), 1671 (Ar) ($C{=}O_{TIB}$), 3388 (OH). **Chiral SFC** (IB, 4% MeOH, 4 ml/min, 125 bar, rt) T_R 7.88 min (minor), 8.66 (major). $[\alpha]_D^{22}$ +2 (*c* 0.5, $CHCl_3$).

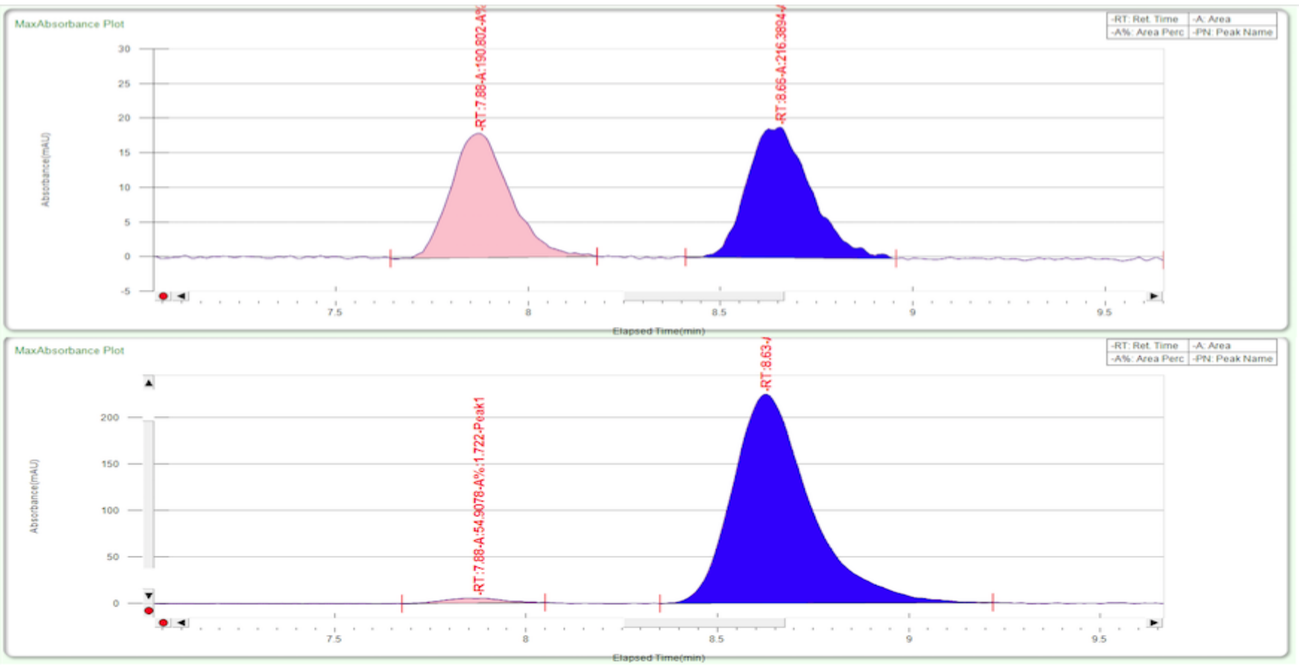

(*R*)-S-(4-Phenyl-2-(trimethylsilyl)butan-2-yl) 2,4,6-triisopropylbenzothioate 6ae

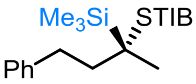

Following a **GP6** using (*R*)-*S*-benzothioate **4a** (99 mg, 0.25 mmol, >99:1 *er*) and trimethylsilyl chloride (60 µl, 0.50 mmol) and purification by flash chromatography (10% toluene/ pentane) gave thiobenzoate **6ae** (80 mg, 68%, 60:40 *er*) as an oil.

$\boldsymbol{R_f}$ (10% toluene/ pentane) 0.36. $\boldsymbol{\delta_H}$ (400 MHz, $CDCl_3$): 7.32 (2H, m, ArH), 7.25 – 7.19 (3H, m, ArH), 7.00 (2H, s, ArH), 3.23 (2H, hept., *J* 6.9, ArCH), 2.99 – 2.78 (3H, m, ArC*H*, ArCH_2), 2.33 (2H, m, $ArCH_2CH_2$), 1.70 (3H, s, CCH_3), 1.25 (18H, d, *J* 6.9, $ArCHCH_3$), 0.16 (9H, s, $SiCH_3$). $\boldsymbol{\delta_C}$ (100 MHz, $CDCl_3$): 199.5 (4° C), 150.3 (4° C), 144.6 (4° C), 142.7 (4° C), 136.6 (4° C), 128.5 (CH), 125.9 (CH) 121.1 (CH), 43.0 (4° C), 38.9 (CH_2), 34.5 (CH), 32.6 (CH_2), 30.7 (CH), 24.0 (CH_3), 21.1 (CH_3), -2.78 (CH_3). $\boldsymbol{\nu_{max}}$ (neat): 1459 (Ar), 1653 (Ar), 1672 ($C{=}O_{TIB}$). **HRMS (ESI)** calc. for $[C_{29}H_{44}OSSi + Na]^+$ 491.2774. Found 491.2777. **Chiral HPLC** (IA, 0.1% *i*PrOH/hexane, 0.5 ml/min, 0°C) T_R 12.4 min (major), 15.2 (minor).

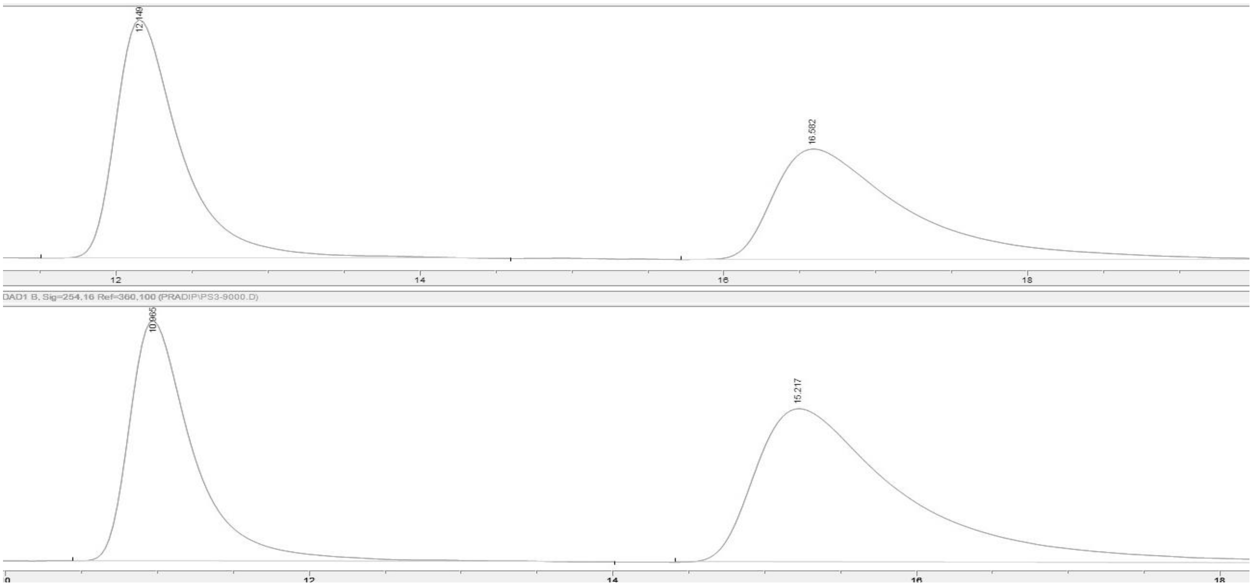

(*R*)-S-(4-Phenyl-2-(tributylstannyl)butan-2-yl) 2,4,6-triisopropylbenzothioate 6af

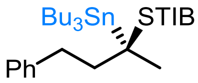

Following a **GP6** using (*R*)-*S*-benzothioate **4a** (30 mg, 0.076 mmol, >99:1 *er*) and tributyltin chloride (62 µl, 0.15 mmol) and purification by flash chromatography (2% toluene/ pentane) gave thiobenzoate **6af** (40 mg, 77%, 67:33 *er*) as an oil.

$\boldsymbol{R_f}$ (5%toluene/pentane) 0.52. $\boldsymbol{\delta_H}$ (400 MHz, $CDCl_3$): 0.92 (9H, t, *J* 7.2), 1.06 (6H, m), 1.24 (18H, br. d., *J* 6.9), 1.36 (6H, m), 1.56 (6H, m), 1.79 (3H, s), 2.23 (1H, td, *J* 13.2, 4.6), 2.41 (1H, td, *J* 13.7, 4.6), 2.65 (1H, td, *J* 13.2, 4.5), 2.80-2.94 (2H, m), 3.14 (2H, sept, *J* 6.9), 7.00 (2H, s), 7.20 (3H, m), 7.30 (2H, m). $\boldsymbol{\delta_C}$ (100 MHz, $CDCl_3$): 10.8 (CH_2, J_{Sn} 320), 13.7 (CH_3), 23.9 (CH_3), 24.4 (CH_3), 26.1 (CH_3), 27.7 (CH_3), 29.3 (CH_2), 30.5 (CH), 34.1 (CH_2), 34.4 (CH), 42.8 (4º C), 42.9 (CH_2), 121.0 (CH), 125.9 (CH), 128.3 (CH), 128.4 (CH), 135.5 (4º C), 142.1 (4º C), 144.6 (4º C), 150.2 (4º C), 200.8 (STIB C=O). $\boldsymbol{\nu_{max}}$ (neat): 901, 1459, 1646, 1673, 2957. **HRMS** (ESI) calc. for $[C_{38}H_{62}OSSn + Na]^+$ 709.3442. Found 709.3411. **Chiral HPLC** (IA, 0.1% *i*PrOH/hexane, 0.5 mL/min, 0 ºC) T_R 22.40 min (major), 24.15 min (minor). $[\alpha]_D^{22}$ +2 (*c* 0.5, $CHCl_3$). Absolute stereochemistry determined after tin-lithium exchange (nBuLi, TMEDA, TBME, –60 °C) and MeOD quench, followed by comparison of the Chiral HPLC with the starting material **4a**.

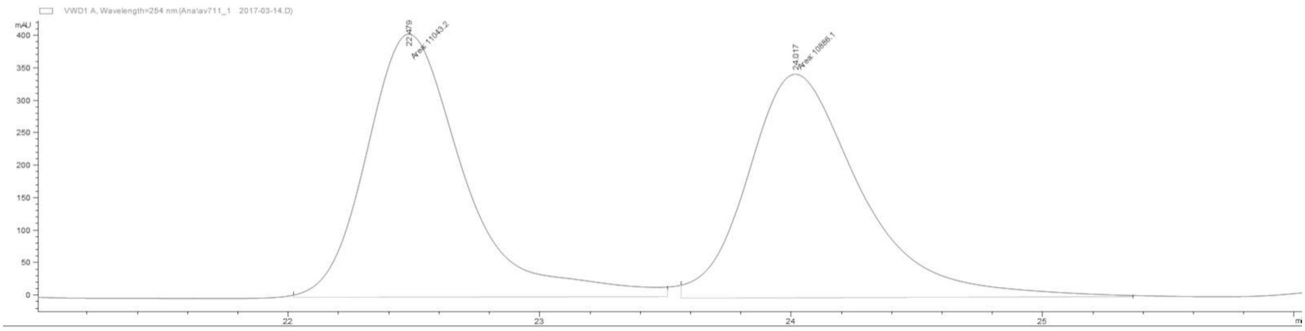

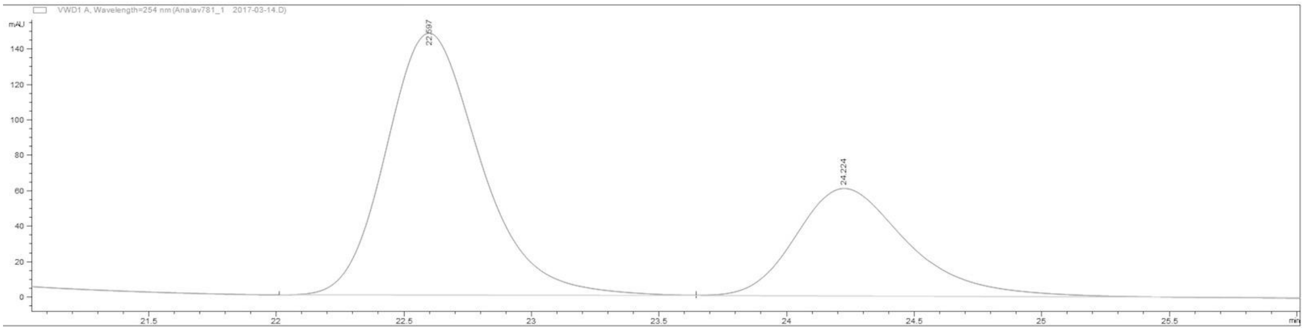

(*S*)-S-(4-Phenyl-2-(trimethylstannyl)butan-2-yl) 2,4,6-triisopropylbenzothioate 6ag

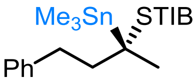

Following a **GP6** using (*R*)-*S*-benzothioate **4a** (99 mg, 0.25 mmol, >99:1 *er*) and trimethyltin chloride (57 µl, 0.50 mmol) and purification by flash chromatography (2% toluene/ pentane) gave thiobenzoate **6ag** (120 mg, 86%, 82:12 *er*) as a white solid.

$\boldsymbol{R_f}$ (5% toluene/ pentane) 0.44. $\boldsymbol{\delta_H}$ (400 MHz, $CDCl_3$) δ 7.29 (2H, m, ArH), 7.22 – 7.15 (3H, m, ArH), 6.99 (2H, s, ArH), 3.08 (2H, hept., *J* 6.9, ArC*H*), 2.88 (1H, hept., *J* 6.9, ArC*H*), 2.81 (1H, m, $ArCH_AH_B$), 2.66 (1H, m, $ArCH_AH_B$), 2.19 (2H, dd, *J* 9.8, 7.6, $ArCH_2CH_2$), 1.65 (3H, s, CCH_3), 1.30 – 1.20 (18H, br. d, *J* 6.9, $ArCHCH_3$), 0.28 (9H, s, $SnCH_3$). $\boldsymbol{\delta_C}$ (100 MHz, $CDCl_3$): 200.8 (4° C), 150.4 (4° C), 144.7 (4° C), 142.0 (4° C), 135.2 (4° C), 128.4 (CH), 128.3 (CH), 125.9 (CH), 121.0 (CH), 42.8 (CH_2), 40.7 (4° C), 34.4 (CH_2), 33.5 (CH), 30.5 (CH), 25.9 (CH_2), 24.4 (CH_3), 23.9 (CH_3), -7.59 (CH_3). $\boldsymbol{\nu_{max}}$ (neat): 1460 (Ar), 1654 (Ar), 1673 ($C{=}O_{TIB}$). $\boldsymbol{\nu_{max}}$ (neat): 903, 1264, 1460, 1644, 2961. **HRMS (ESI)** calc. for $[C_{17}H_{24}OSSn + Na]^+$ 583.2031. Found 583.2028. **Chiral HPLC** (IB with guard, 0.1% *i*PrOH/hexane, 0.5 mL/min, 0 °C T_R 25.0 min (major), 26.5 min (minor). $[\alpha]_D^{22}$ +6 (*c* 1.0, $CHCl_3$). Absolute stereochemistry determined after tin-lithium exchange (nBuLi, TMEDA, TBME, –60 °C) and MeOD quench, followed by comparison of the Chiral HPLC with the starting material **4a**.

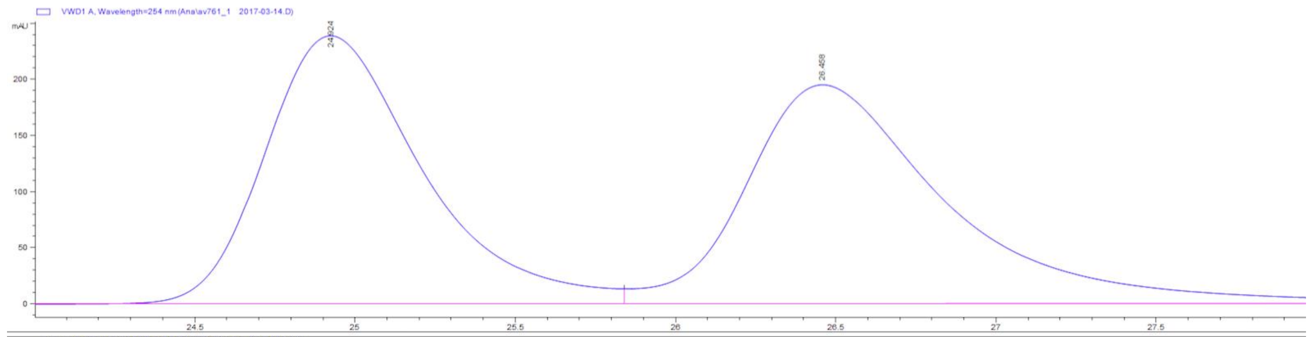

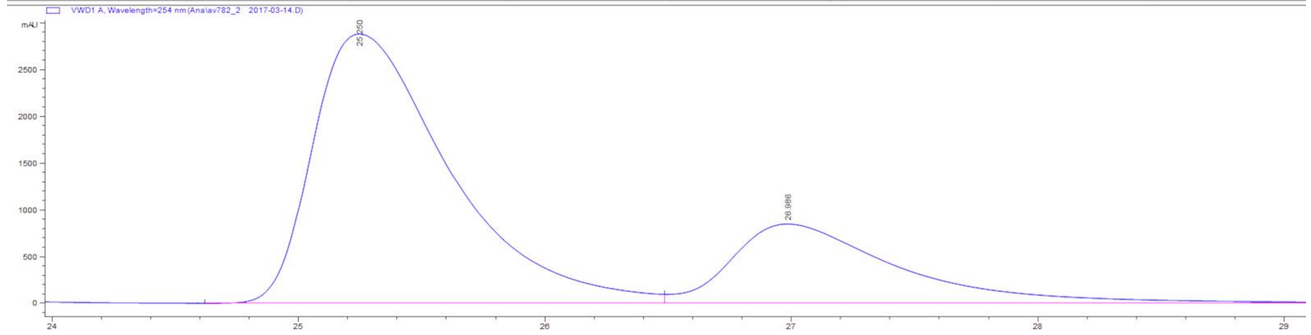

(*R*)-2-<ethyl-4-phenyl-2-((2,4,6-triisopropylbenzoyl)thio)butanoic acid 6ah

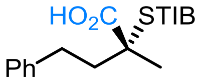

Following a **GP6** using (*R*)-*S*-benzothioate **4a** (99 mg, 0.25 mmol, >99:1 *er*) and *dry ice** and purification by flash chromatography (5% Et_2O/ pentane) gave thiobenzoate **6ah** (94 mg, 85%, > 97:3 *er*) as an oil.

Rf (1% MeOH/CH_2Cl_2). **δ_H** (400 MHz, $CDCl_3$): 1.24 (18H, br. d., *J* 6.7), 1.84 (3H, s), 2.29 (2H, m), 2.75 (2H, m), 2,88 (1H, sept, *J* 6.9), 3.13 (2H, m), 7.00 (2H, s), 7.17 (3H, m), 7.25 (2H, m). **δ_C** (100 MHz, $CDCl_3$): 23.3 (CH_3), 24.0 (CH_3), 30.5 (CH), 31.2 (CH_2), 34.5 (CH), 39.9 (CH_2), 56.2 (4° C), 121.3 (CH), 126.2 (CH) 128.5 (CH), 128.6 (CH), 134.4 (4° C), 140.9 (4° C), 145.1 (4° C), 178.8 (acid C=O), 197.6 (STIB C=O). **HRMS (ESI)** calc. for $[C_{27}H_{36}O_3S + Na]^+$ 463.2277. Found 447.2272. **Chiral SFC** (Chiracel IA, 4% IPA:hexane (1:1, v:v), 4 mL/min, 125 bar, 40 °C) TR = 5.10 min (major) and 5.59 min (minor). $[\alpha]_D^{22}$ +13.5 (*c* 1.0, $CHCl_3$). **Dry ice passed over dry $MgSO_4$.*

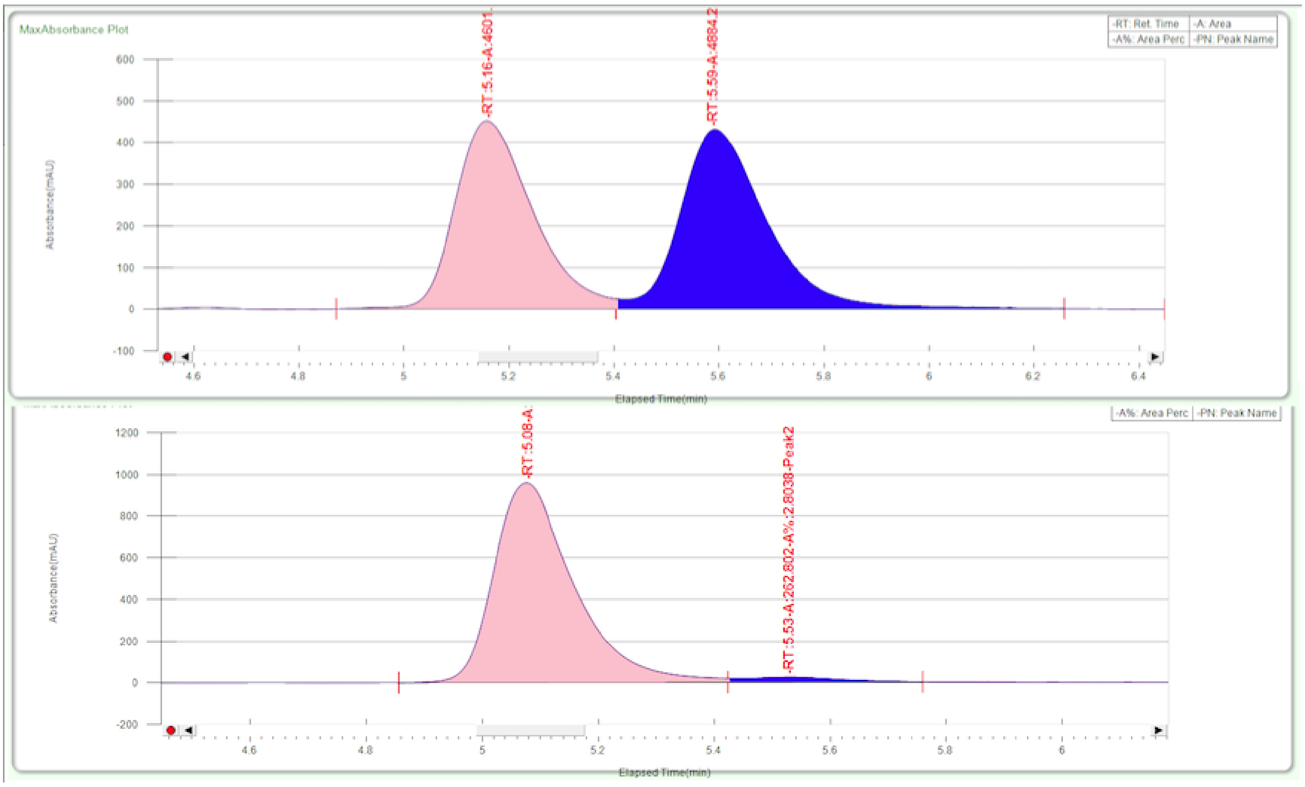

(*R*)-S-(1-((4-Bromophenyl)amino)-2-methyl-1-oxo-4-phenylbutan-2-yl) 2,4,6-triisopropylbenzothioate 6ba

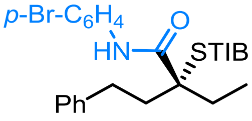

Following a **GP6** using (*R*)-*S*-benzothioate **4b** (50 mg, 0.12 mmol, >99:1 *er*) and 4-Bromophenyl isocyanate (47 mg, 0.24 mmol) and purification by flash chromatography (5% Et_2O/ pentane) gave thiobenzoate **6ba** (30 mg, 41%, 97:3 *er*) as an oil.

R_f (5%Et_2O/pentane) 0.31. **δ_H** (400 MHz, $CDCl_3$): 1.11 (3H, t, *J* 7.3, CH_2CH_3), 1.26 (18H, br. d., *J* 6.8, ArCHC*H*$_3$), 2.21 (1H, dq, *J* 14.6, 7.3, $CH_3CH_ACH_B$), 2.35-2.54 (3H, m, $CH_3CH_ACH_B$ & $ArCH_2CH_2$), 2.69 (1H, td, *J* 12.1, 5.1, $ArCH_ACH_B$), 2.82 (1H, td, *J* 12.1, 5.1, $ArCH_ACH_B$), 2.90 (1H, sept, *J* 6.8, ArC*H*CH_3), 2.99 (2H, sept, *J* 6.8, ArC*H*CH_3), 7.01 (2H, s, ArC*H*CH_3), 7.22 (3H. m, Ar*H*), 7.31 (2H, m, Ar*H*), 7.49 (4H, m, Ar*H*), 9.12 (N*H*). **δ_C** (100 MHz, $CDCl_3$): 8.6 (CH_3), 23.9 (CH_3), 26.6 (CH_2), 30.6 (CH_2), 30.9 (CH), 34.4 (CH), 34.8 (CH_2), 63.2 (4° C), 116.8 (4° C), 121.2 (CH), 121.4 (CH), 126.2 (CH), 128.3 (CH), 128.6 (CH), 131.9 (CH), 134.2 (4° C), 137.3 (4° C), 141.0 (4° C), 144.7 (4° C), 170.1 (amide C=O), 200.3 (STIB C=O).. **ν_{max}** (neat): 893, 1488, 1519, 1591, 1681, 2960, 3329. **HRMS** (ESI) calc. for $[C_{34}H_{42}BrNO_3S + Na]^+$ 630.2012. Found 630.2011. **Chiral HPLC** (IB with

guard, 1% *i*PrOH/hexane, 0.5 mL/min, T_R 12.36 min (minor), 12.90 min (major). $[\alpha]_D^{22}$ –2 (*c* 0.55, $CHCl_3$).

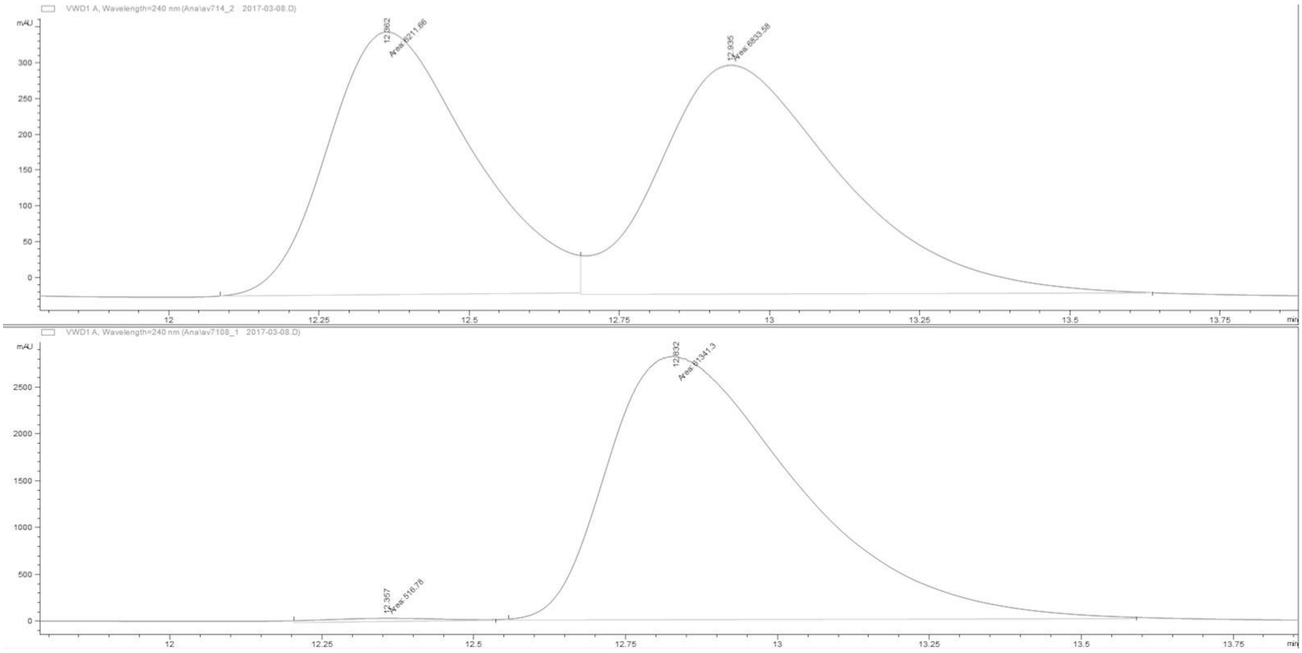

Methyl (*R*)-2-methyl-2-((2,4,6-triisopropylbenzoyl)thio)pentanoate 6ca

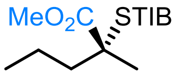

Following a **GP6** using (*R*)-*S*-benzothioate **4c** (41.0 mg, 0.12 mmol, 99:1 *er*) and methyl chloroformate (57.0 µl, 0.74 mmol) gave (*R*)-*S*-thiobenzoate **6ca** (42 mg, 89%, 92:8 *er*) as a colourless oil.

R$_f$ (4%Et_2O/pentane) 0.33. **δ_H** (400 MHz, $CDCl_3$): 0.93 (3H, t, *J* 7.4), 1.24 (18H, d, *J* 6.8), 1.41 (2H, m), 1.74 (3H, s), 1.87 (2H, m), 2.88 (1H, hept., *J* 6.8), 3.07 (2H, hept., *J* 6.8), 3.77 (3H, s), 6.98 (2H, s). **δ_C** (100 MHz, $CDCl_3$): 14.3 (CH_3), 18.1 (CH_2), 23.4 (CH_3), 24.0 (CH_3), 24.5 (CH_3), 30.4 (CH), 34.6 (CH), 40.4 (CH_2), 52.7 (CH_3), 56.2 (4° C), 121.1 (CH), 134.7 (4° C), 145.0 (4° C), 150.8 (4° C), 174.0 (4° C), 197.5 (4° C). **ν_{max}** (neat): 659, 897, 1240, 1460, 1678, 1738, 2959. **HRMS (ESI)** calc. for $[C_{23}H_{36}O_3S + Na]^+$ 415.2277. Found 415.2270. **Chiral HPLC** (IB with guard, 0.3% *i*PrOH/hexane, 0.5 ml/min, 0 °C) T_R 10.18 min (minor), 11.45 min (major). $[\alpha]_D^{22}$ –14.7 (*c* 0.55, $CHCl_3$).

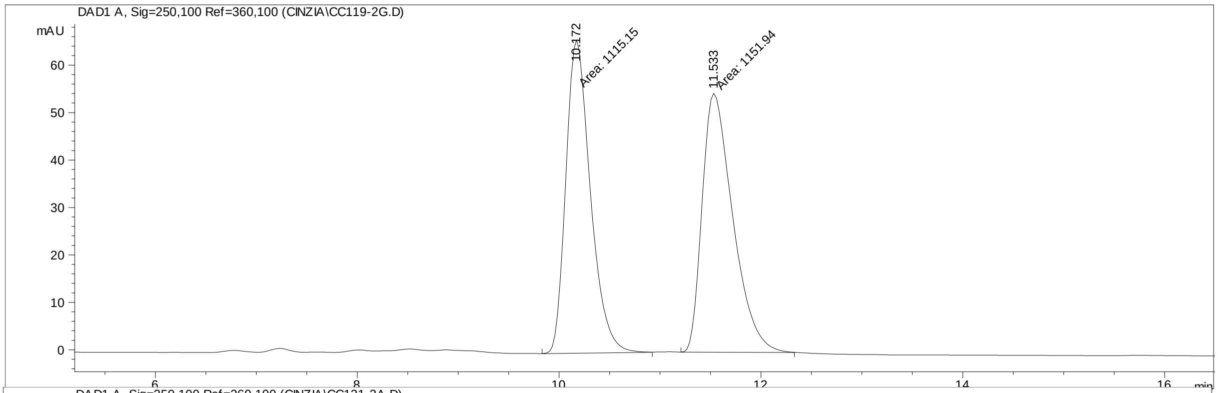

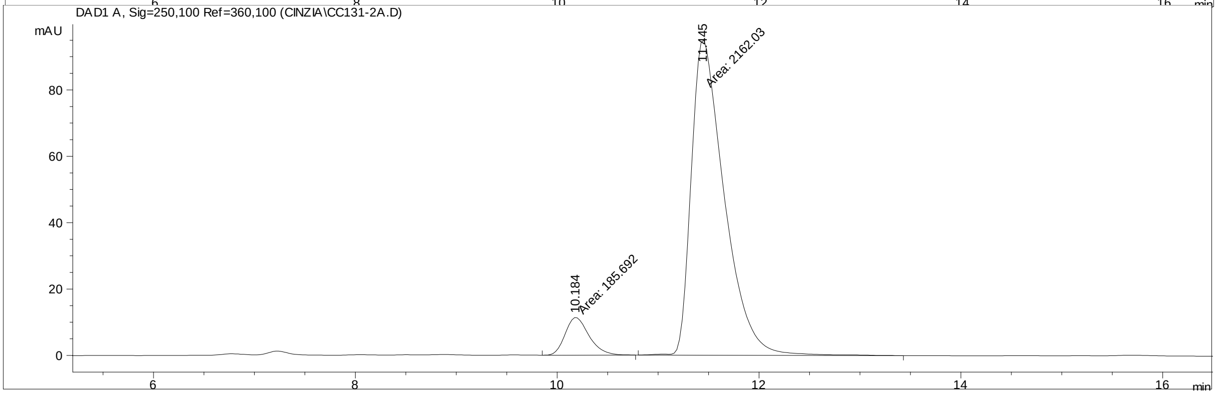

(*R*)-S-(1-((4-Bromophenyl)amino)-2-methyl-1-oxohex-5-en-2-yl) 2,4,6-triisopropylbenzothioate 6da

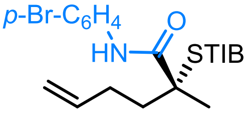

Following a **GP6** using (*R*)-*S*-benzothioate **4d** (50 mg, 0.14 mmol, >99:1 *er*) and 4-Bromophenyl isocyanate (55 mg, 0.28 mmol) and purification by flash chromatography (5% Et_2O/ pentane) gave thiobenzoate **6da** (68 mg, 87%, 96:4 *er*) as an amorphous solid.

$\boldsymbol{R_f}$ (5% Et_2O/pentane) 0.40. $\boldsymbol{\delta_H}$ (400 MHz, $CDCl_3$): 1.25 (18H, br. d., *J* 6.9), 1.74 (3H, s), 2.13-2.36 (4H, m), 5.06 (2H, m), 5.83 (1H, m), 7.00 (2H, s), 7.47 (4H, m), 9.17 (1H, s). $\boldsymbol{\delta_C}$ (100 MHz, $CDCl_3$): 22.1 (CH_3), 23.8 (CH_3), 28.9 (CH_2), 30.9 (CH), 34.4 (CH), 36.7 (CH_2), 58.4 (4° C), 115.6 (CH_2), 116.9 (4° C), 121.2 (CH), 121.3 (CH), 131.9 (CH), 133.9 (4° C), 136.9 (CH), 137.3 (4° C), 144.7 (4° C), 151.4 (4° C), 170.5 (amide C=O), 200.3 (STIB C=O). $\boldsymbol{\nu_{max}}$ (neat): 893, 1393, 1487, 1527, 1678, 2960, 3353. **HRMS** (ESI) calc. for $[C_{29}H_{38}BrNO_2S + Na]^+$ 566.1699. Found 566.1675. **Chiral HPLC**: (IB, 2% *i*PrOH/hexane, 0.5 mL/min, rt) T_R 10.62 min (minor), 11.41 min (major).. $[\alpha]_D^{22}$ +6 (*c* 1.0, $CHCl_3$).

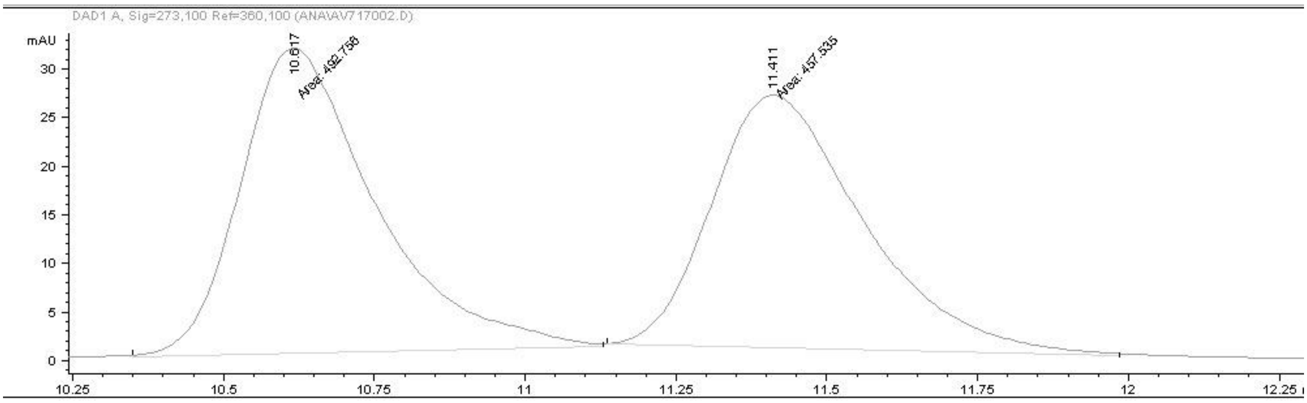

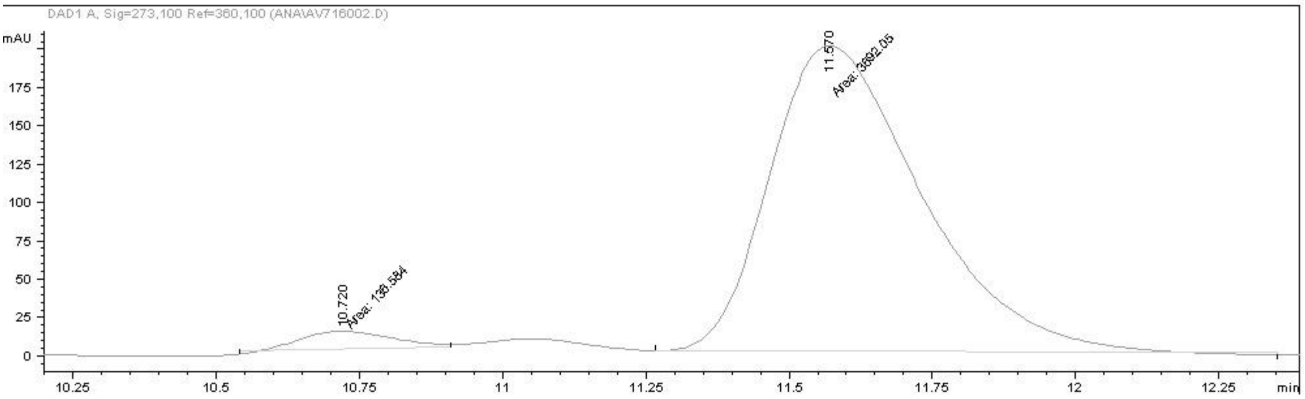

S-((2*R*)-1-((4-Bromophenyl)amino)-2-methyl-1-oxo-5-((tetrahydro-2H-pyran-2-yl)oxy)pentan-2-yl) 2,4,6-triisopropylbenzothioate 6ea

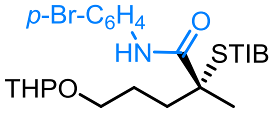

Following a **GP6** using (*R*)-*S*-benzothioate **4e** (30 mg, 0.07 mmol, >99:1 *er*) and 4-Bromophenyl isocyanate (27 mg, 0.14 mmol) and purification by flash chromatography (10% EtOAc/petroleum ether) gave thiobenzoate **6ea** (22 mg, 51%, 99:1 *er**) as an oil.

R_f (10%EtOAc/petroleum ether). 0.30. **δ_H** (400 MHz, $CDCl_3$): 1.05 – 1.21 (18H, m), 1.39 – 1.54 (4H, m), 1.60 – 1.81 (7H, m), 2.02 – 2.36 (2H, m), 2.76 – 2.92 (3H, m), 3.40 (2H, m), 3.74 (2H, m), 4.50 (1H, m), 6.92 (2H, s), 7.34 – 7.43 (4H, m), 9.10 (1H, s). **δ_C** (100 MHz, $CDCl_3$): 19.5 (CH_2), 22.1 (CH_3), 23.8 (CH_3), 25.0 (CH_2), 25.4 (CH_2), 30.6 (CH_2), 30.8 (CH), 34.3 (CH_2), 34.4 (CH), 58.4 (4° C), 62.3 (CH_2), 66.9 (CH_2), 98.9 (CH), 116.8 (4° C), 121.2 (2 x CH), 131.9 (CH), 134.0 (4° C), 137.3 (4° C), 144.7 (4° C), 151.3 (4° C), 170.6 (C=O), 200.4 (C=O). **ν_{max}** (neat): 892, 1072, 1489, 1590, 1673, 2960, 3318. **HRMS** (ESI) calc. for $[C_{33}H_{46}BrNO_4S + Na]^+$ 654.2223. Found 654.2206. **Chiral SFC*** (Chiracel IA, 20% IPA, 4 mL/min, 125 bar, 25 °C) T_R = 12.8 min (minor) and 15.5 min (major).

* e.r. determined after THP removal. $[\alpha]_D^{22}$ –6 (*c* 0.30, $CHCl_3$).

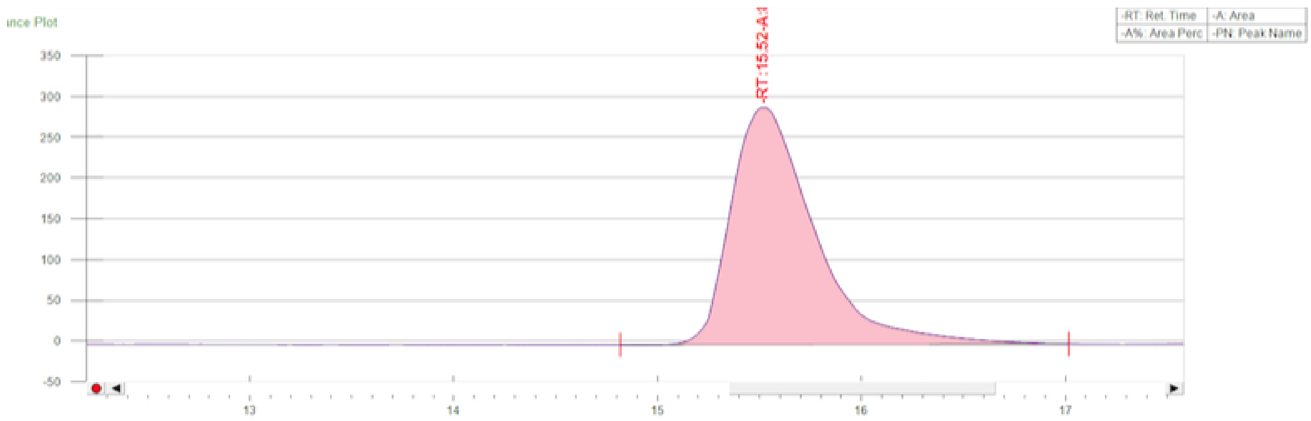

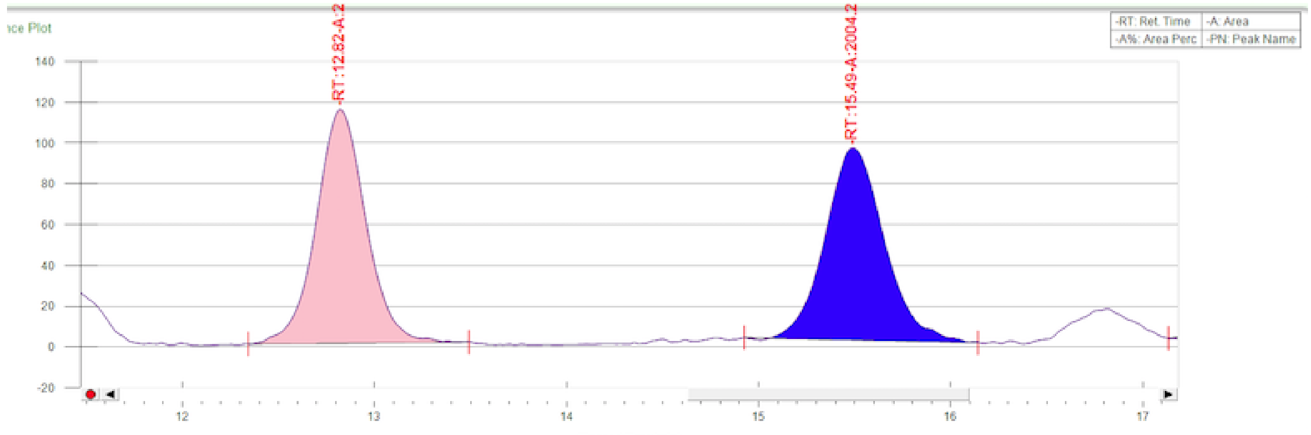

Methyl (3*R*,8*S*,9*S*,10*R*,13*R*,14*S*,17*R*)-10,13-dimethyl-17-((*R*)-6-methylheptan-2-yl)-3-((2,4,6-triisopropylbenzoyl)thio)-2,3,4,7,8,9,10,11,12,13,14,15,16,17-tetradecahydro-1H-cyclopenta[a]phenanthrene-3-carboxylate 9

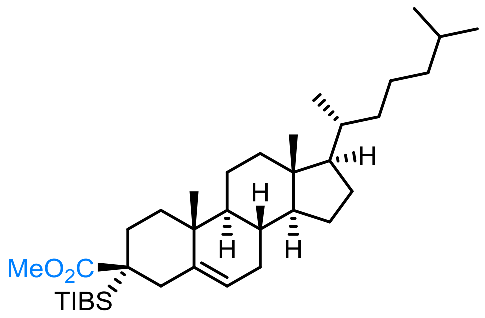

Following a **GP6** using (*R*)-*S*-benzothioate **8** (200 mg, 0.31 mmol) and methyl chloroformate (48.0 µl, 0.62 mmol) gave (*R*)-*S*-thiobenzoate **9** (173 mg, 81%) as a white foam.

***R**$_f$* (pentane:Et_2O 95:5) 0.35. **δ_H** (400 MHz, $CDCl_3$): 0.68 (3H, s), 0.87 (6H, dd, *J* 6.5, 1.7 Hz), 0.92 (3H, m), 0.99 (2H, m), 1.04 (3H, s), 1.12 (4H, m), 1.24 (20H,, m), 1.34 (4H, m), 1.47 (5H, m), 1.57 (2H, m), 1.81 (2H, m), 2.02 (2H, m), 2.14 (1H, m), 2.22 (1H, m), 2.60 (1H, dd, *J* 14.6, 2.1 Hz), 2.87 (1H, sept, *J* 6.5 Hz), 3.00 (1H, m), 3.06 (2H, m), 3.78 (3H, s), 5.37 (1H, m), 6.97 (2H, s). **δ_C** (100 MHz, $CDCl_3$): 11.8 (CH_3), 18.7 (CH_3), 19.4 (CH_3), 20.8 (CH_2), 22.5 (CH_3), 22.8 (CH_3), 23.8 (CH_2), 23.9 (2 x CH_3), 24.2 (CH_2), 25.2 (4 x CH_3), 28.0 (CH), 28.1 (CH_2), 29.7 (CH), 30.2 (2 x CH_2), 31.7 (CH), 32.0

(CH2), 34.4 (CH), 35.6 (CH2), 36.2 (CH2), 36.6 (4º C), 39.1 (CH2), 39.5 (CH2), 42.2 (4º C), 50.2 (CH), 52.6 (CH3), 56.0 (CH), 56.7 (CH), 59.2 (4º C), 120.9 (2 x CH), 134.8 (4º C), 138.0 (4º C), 144.9 (2 x 4º C), 150.6 (4º C), 174.1 (C=O), 197.2 (C=O). ν_{max} (neat): 2958, 1737, 1677, 1460, 1248, 897. **HRMS** (ESI) calc. for $[C_{45}H_{70}O_3S + Na]^+$ 713.4937. Found 713.4920. $[\alpha]_D^{22}$ +10 (*c* 1.0, $CHCl_3$).

Determination of stereochemistry of 9

The stereochemistry of the quaternary centre (carbon 1, Figure S1) in **9** was established by the values of two bond ^1H-^{13}C scalar coupling constants ($^2J_{HC}$), which have a karplus-type relationship with the dihedral angles between the protons of interest (H_b, Figure S1d) and the heteroatom attached to the carbon of interest (**C**-OH, Figure S1d).[3] When a proton and an electron withdrawing heteroatom (such as oxygen or sulphur) have a *gauche* orientation, a large $^2J_{HC}$ between the proton and the carbon attached to the heteroatom is measured whereas a small value is obtained for the corresponding *anti* orientation.

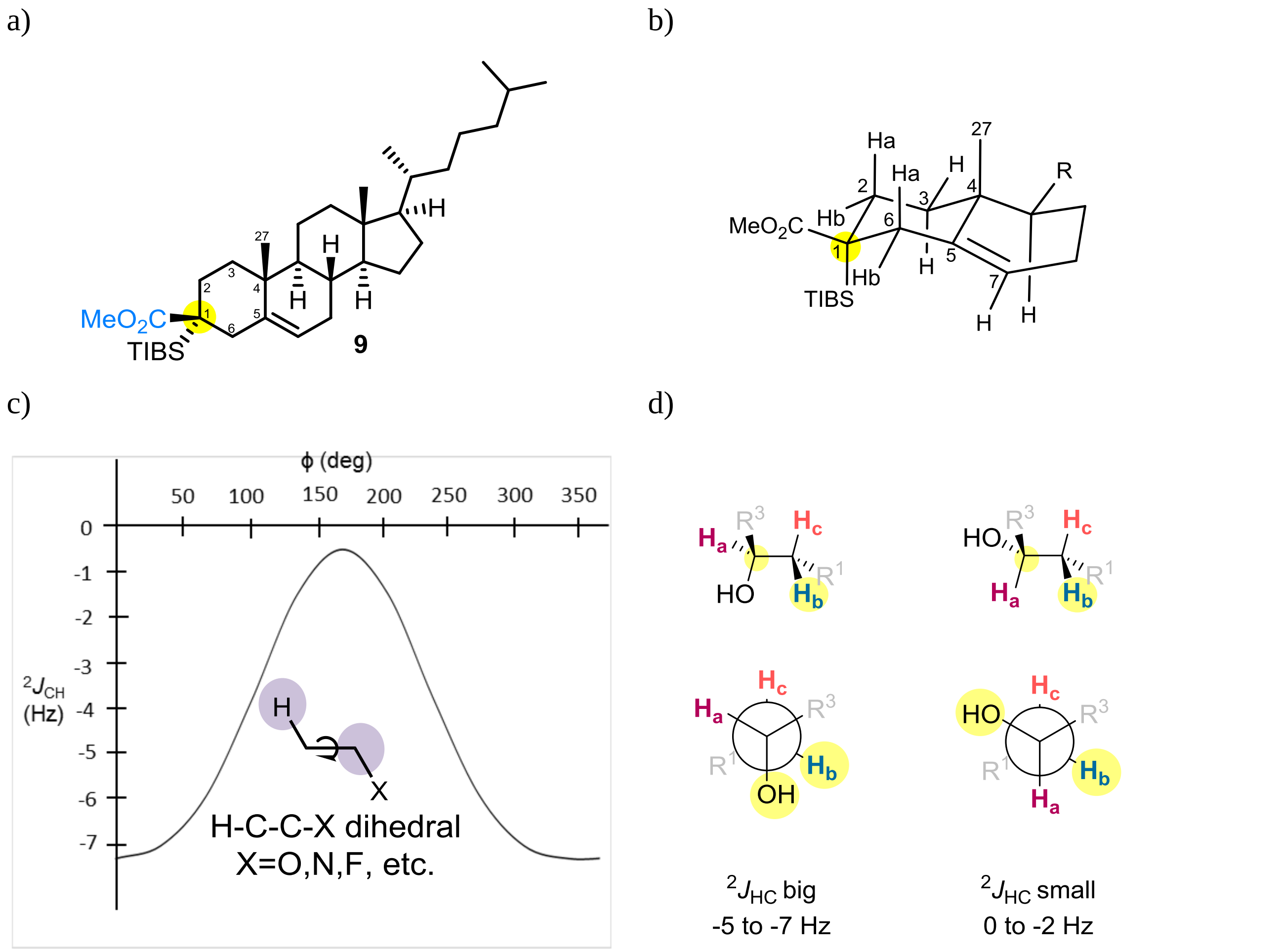

Figure S1. **a**, Structure of **9**. **b**, three dimensional structure of the motif of interest. **c**, $^2J_{HC}$ values as a function of dihedral angle ϕ. **d**, Typical big and small $^2J_{HC}$ values for a mono-oxygenated system.

If the STIB group did not epimerise to the equatorial position during lithiation, the subsequent electrophilic trapping of the lithiated species ($ClCO_2Me$ quench) should generate **9** (Figure S1a) as a single diastereomer with the bulky STIB group in the axial position (Figure S1b). If this is the case, H6a would be *anti* to the sulphur and H6b would be *gauche* to the sulphur and therefore a small $^2J_{H6a\text{-}C1}$ and a large $^2J_{H6b\text{-}C1}$ should be observed. In addition, a small $^2J_{H2a\text{-}C1}$ and a large $^2J_{H2b\text{-}C1}$ should also be observed.

The protons of methyl group 27 (H27) was first assigned to aid the assignment of the axial and equatorial protons (H6a/b and H2a/b) within the ring. In the ^{1}H NMR, the signal at 5.36 ppm was assigned to be the alkene proton H7, which has an HMBC correlation with both C6 and C4 (39.25 and 36.73 ppm respectively). The quaternary carbon C4 has three HMBC correlations: one to H7 (5.36 ppm), one to H6b (2.58 ppm), and one to a singlet at 1.04 ppm, which was assigned to be H27. The assignment was also supported by the HMBC correlation between C3 and H27.

Both axial (Ha) and equatorial (Hb) protons of H6 and H2 were assigned using NOESY correlation. Based on other 2D NMR data, protons at 2.99 and 2.57 ppm correlate to C6 and protons at 2.22 and 2.12 ppm correlate to C2. The axial protons H6a and H2a were assigned to be the peaks at 2.99 ppm and 2.22 ppm respectively due to the strong correlations between these protons and H27.

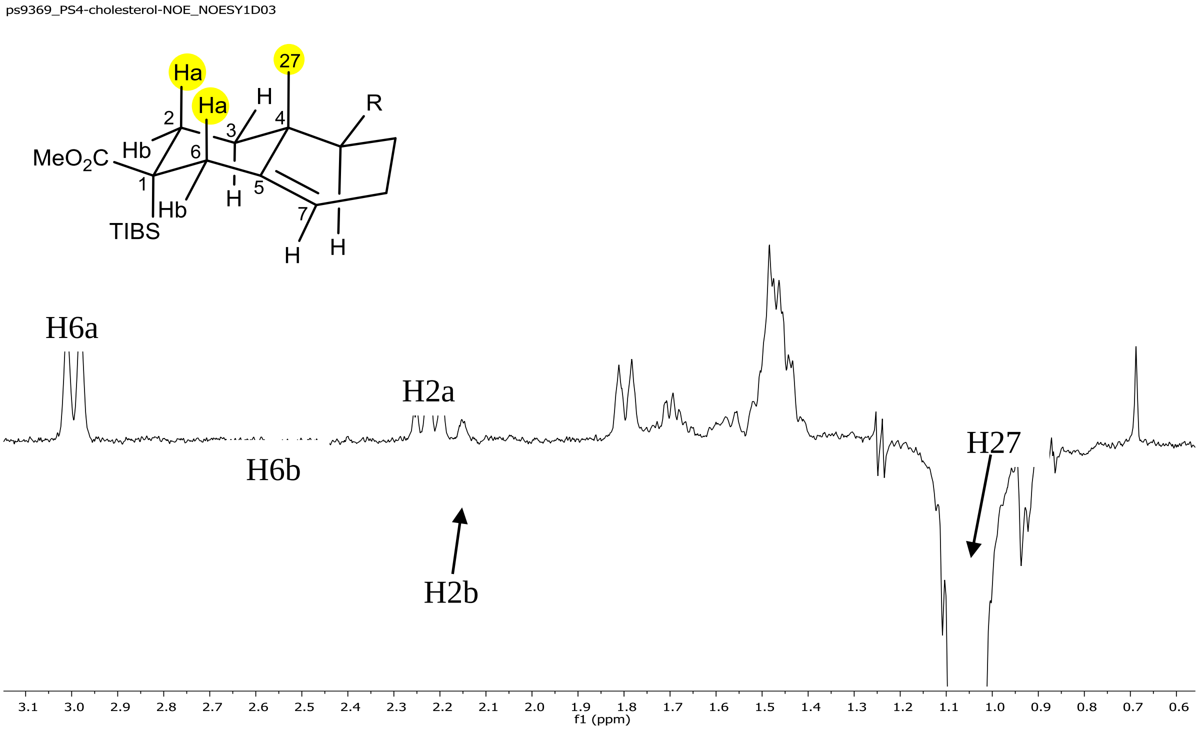

Figure S2. 1D NOESY spectrum irradiating proton 27 at 1.04 ppm.

Having established the assignment of H6a, H6b, H2a and H2b, Accordion In-Phase and Anti Phase (IPAPA) HSQMBC[4] spectra were recorded on **9** (12 scans, 1600 t1 increments and 8192 t2 data points,

f1 spectra width 219.6 ppm (27624.7 Hz) f2 spectra width 10.0 ppm (5005.0 Hz), matching J_{LR} from 3Hz to 8Hz). Both In-Phase (IP) and Anti-Phase (AP) spectra were recorded interleaved. The sum and difference spectra were obtained by adding and subtracting, respectively, the AP and IP spectra. The offset between the sum (red, Figure S3) and the difference (blue, Figure S3) spectra allows the extraction of the ^{1}H-^{13}C scalar coupling constants.

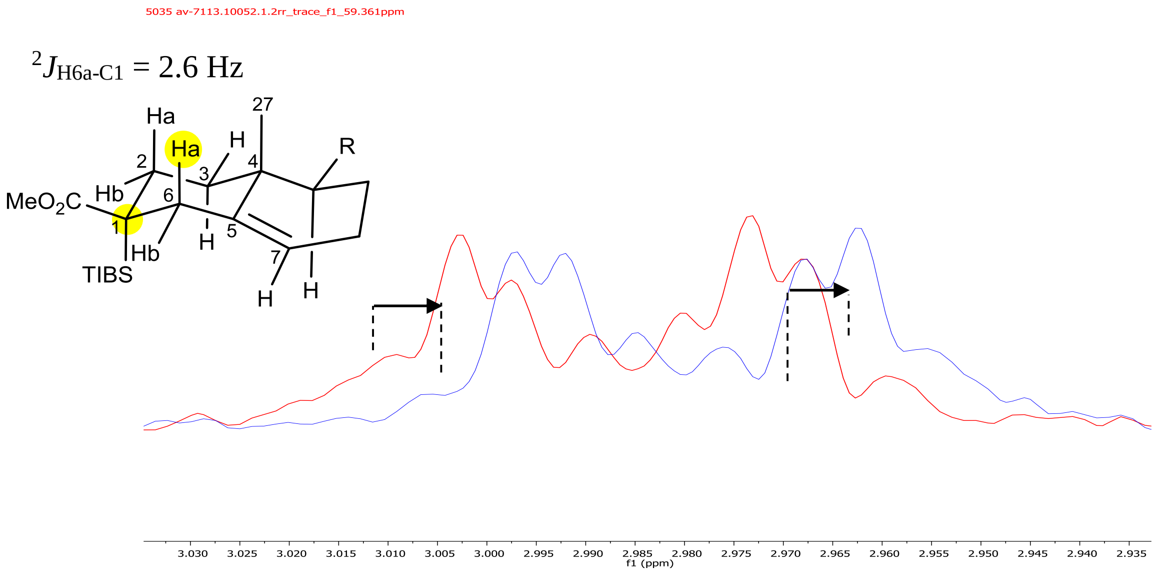

Figure S3. Overlay of the sum (red) and difference (blue) HSQMBC traces for C1 (59.31 ppm) showing a correlation to H6a (at 2.99 ppm). The offset between the traces gives a $^{2}J_{H6a\text{-}C1}$ = 2.6 Hz.

Table 1 summarises all the $^{2}J_{HC}$ extracted around the quaternary carbon. The magnitudes of the observed $^{2}J_{HC}$ values are in good agreement with the STIB group of **9** in the axial position.

Table 1 A summary of key $^{2}J_{HC}$ values extracted. For HSQMBC traces for the coupling between H6b-C1, H2a-C1 and H2b-C1, see Figure S5 and S6.

^{1}H (ppm)	^{13}C (ppm)	*J* values/ Hz	Stereochemical relationship to sulphur
6a (2.99)	C1 (59.31)	2.6	*anti*
6b (2.57)	C1 (59.31)	5.3	*gauche*
2a (2.22)	C1 (59.31)	1.9	*anti*
2b (2.12)	C1 (59.31)	5.1	*gauche*

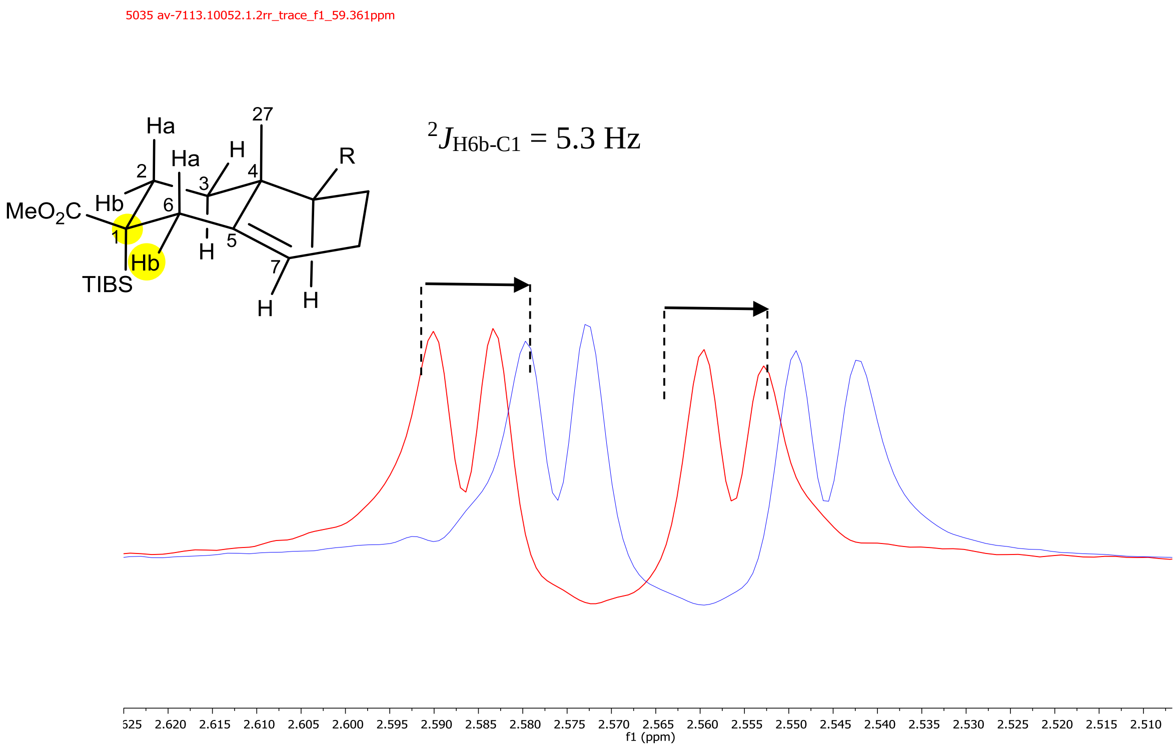

Figure S4. Overlay of the sum (red) and difference (blue) HSQMBC traces for C1 (59.31 ppm) showing a correlation to H6b (at 2.57 ppm) The offset between the traces suggest $^2J_{H6a\text{-}C1}$ = 5.3 Hz.

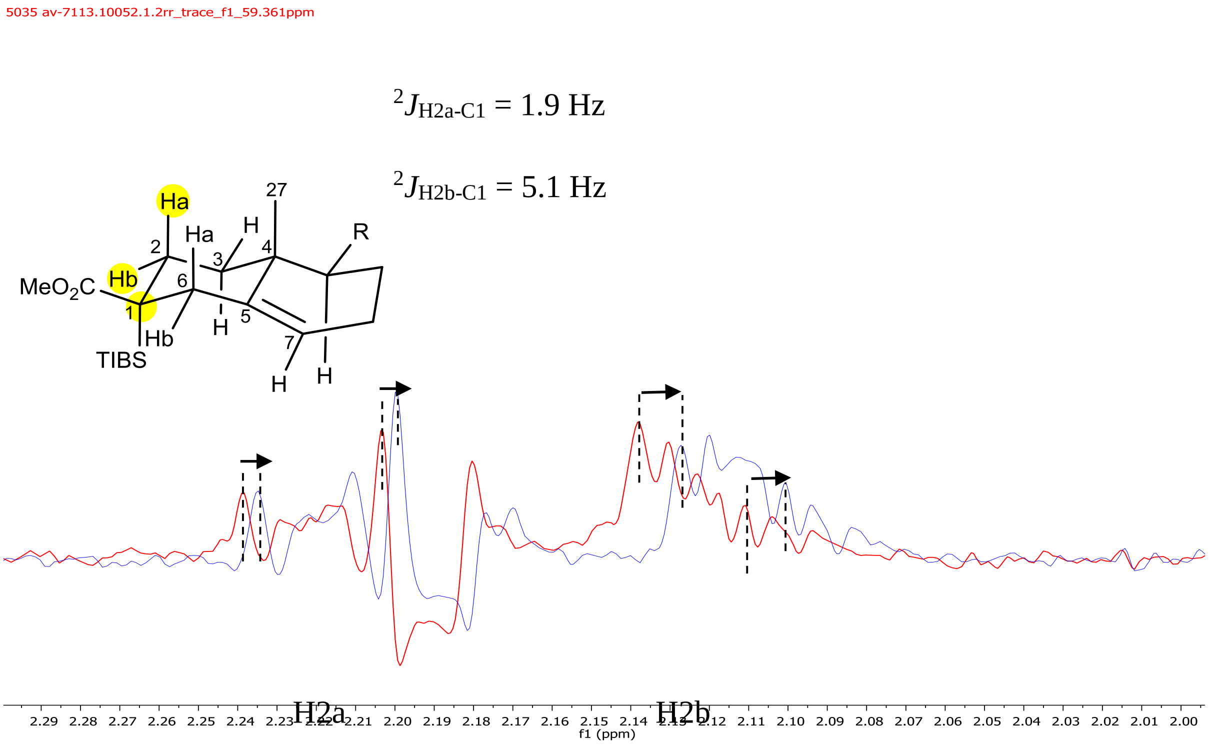

Figure S5. Overlay of the sum (red) and difference (blue) HSQMBC traces for C1 (59.31 ppm) showing a correlation to H2a (at 2.22 ppm) and H2b (2.12 ppm). The offset between the traces suggest $^2J_{H2a\text{-}C1}$ = 1.9 Hz and $^2J_{H2b\text{-}C1}$ = 5.1 Hz.

Removal of TIB Group in thiobenzoate 6ad

(1*R*,2*R*)-2-Mercapto-2-methyl-1,4-diphenylbutan-1-ol 7

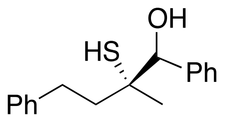

To a solution of $LiAlH_4$ (7.5 mg, 0.2 mmol, 5.0 equiv.) in THF (0.5 mL) was added a solution of thiobenzoate **6ad** (20 mg, 0.04 mmol, 1.0 equiv.) in THF (1 mL). The mixture was stirred at room temperature for 16 h. The solution was diluted with Et_2O (0.5 mL), and then water (0.02 mL) was added dropwise under vigorous stirring. 1.3 M NaOH (0.05 mL) was added and the mixture was stirred for 2 h. The resulting white precipitate was filtered on filter paper, washing with Et_2O. The solvent was evaporated under reduced pressure. The crude product was purified by column chromatography on silica gel, eluting with pentane:toluene:Et_2O 2:1:1, to give **7** (6 mg, 60%, 98:2 e.r.) as an oil.

R_f (pentane:toluene:Et_2O 2:1:1) 0.5. **δ_H** (400 MHz, $CDCl_3$): 1.28 (3H, s), 1.90 (2H, m), 2.71 (1H, m), 2.98 (1H, m), 4.62 (1H, s), 7.18 (2H, m), 7.24–7.41 (8H, m). **δ_C** (100 MHz, $CDCl_3$): 23.5 (CH_3), 31.2 (CH_2), 43.1 (CH_2), 54.9 (4° C), 80.8 (CH), 125.9 (CH), 127.8 (2 x CH), 128.0 (CH), 128.1 (2 x CH), 128.4 (2 x CH), 128.5 (2 x CH), 139.5 (4° C), 141.9 (4° C). **ν_{max}** (neat): 3370, 2921, 1456, 1260, 1018, 801. **HRMS** (ESI) calc. for $[C_{17}H_{20}OS + Na]^+$ 295.1127. Found 295.1120. **Chiral SFC** (Chiracel Whelk-O1, 20% IPA:hexane (1:1, v:v), 4 mL/min, 125 bar, 25 °C) T_R = 8.60 min (major) and 11.60 min (minor). $[\alpha]_D^{22}$. –4 (*c* 0.30, $CHCl_3$).

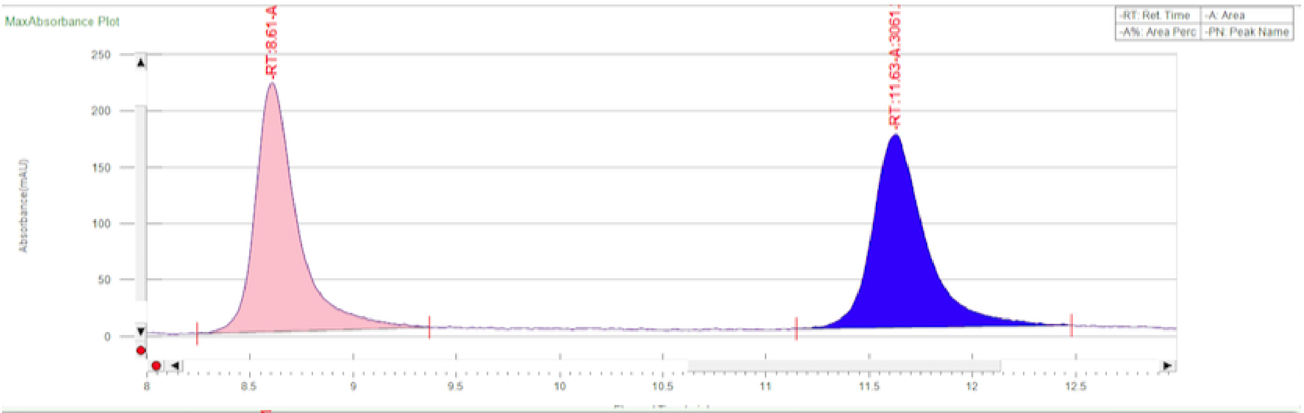

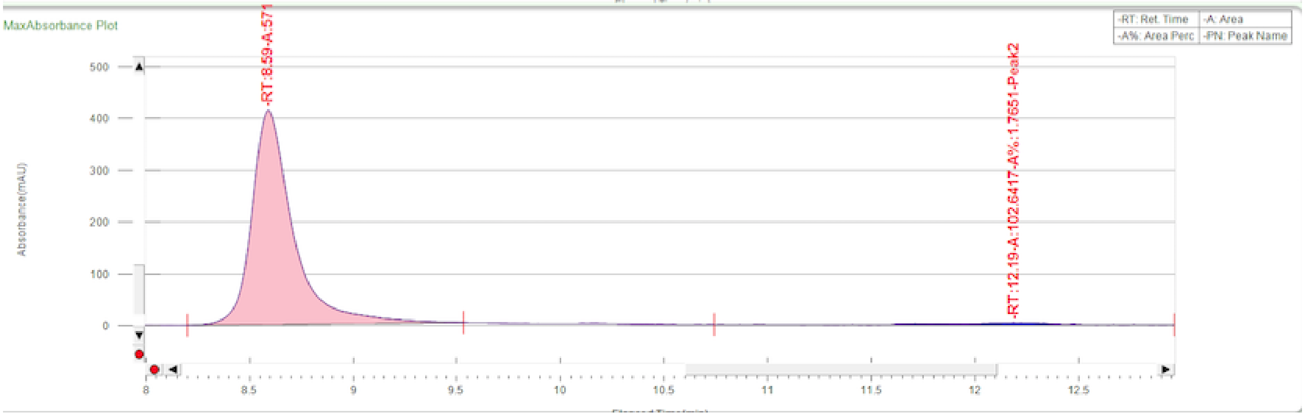

3.4 Studies on enantiomerisation of Li-4a

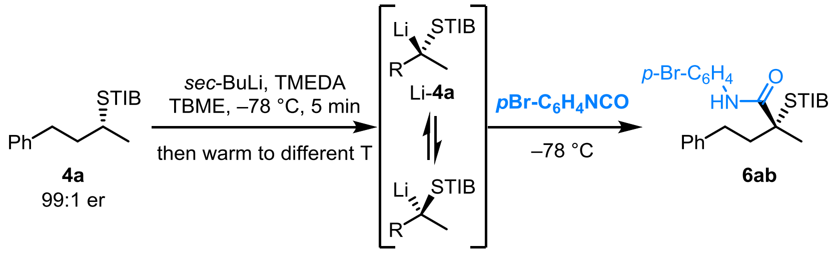

The kinetic data for the racemization were treated under reversible first-order conditions in **4a** using the following equation:[5]

$$0.5\ln([\text{Li}-\mathbf{4a}]-2[R]) = -kt + c$$

Table S1. Enantiomer ratios over time for racemization of Li-**4a** in the presence of 1.2 equiv. TMEDA in TBME

Temperature (K)	Time (s)	ee	0.5ln([4a]-2[R])
195	0	96.46	2.284564207320410
	1800	84.52	2.218494096373930
	3600	73.04	2.145503618143360
	7200	59.94	2.046672030944260
	10800	51.98	1.975429514606670
203	0	96.7298	2.285960762290720
	900	81.2442	2.198729717023230
	1800	64.6074	2.084164477655840
	2700	53.66	1.991356285025110
208	0	97.7934	2.291428545050500
	900	67.0112	2.102429884799850
	1800	44.8928	1.902138712771160
	2700	31.5288	1.725450706920640
	3600	22.9314	1.566253575195980
213	0	98.6046	2.295558956832190
	300	70.0694	2.124743089740040

	600	52.3138	1.978630099299020
	900	38.002	1.818819394960170
	1200	29.207	1.687204203285970

The results using this equation are displayed in Chart S1.

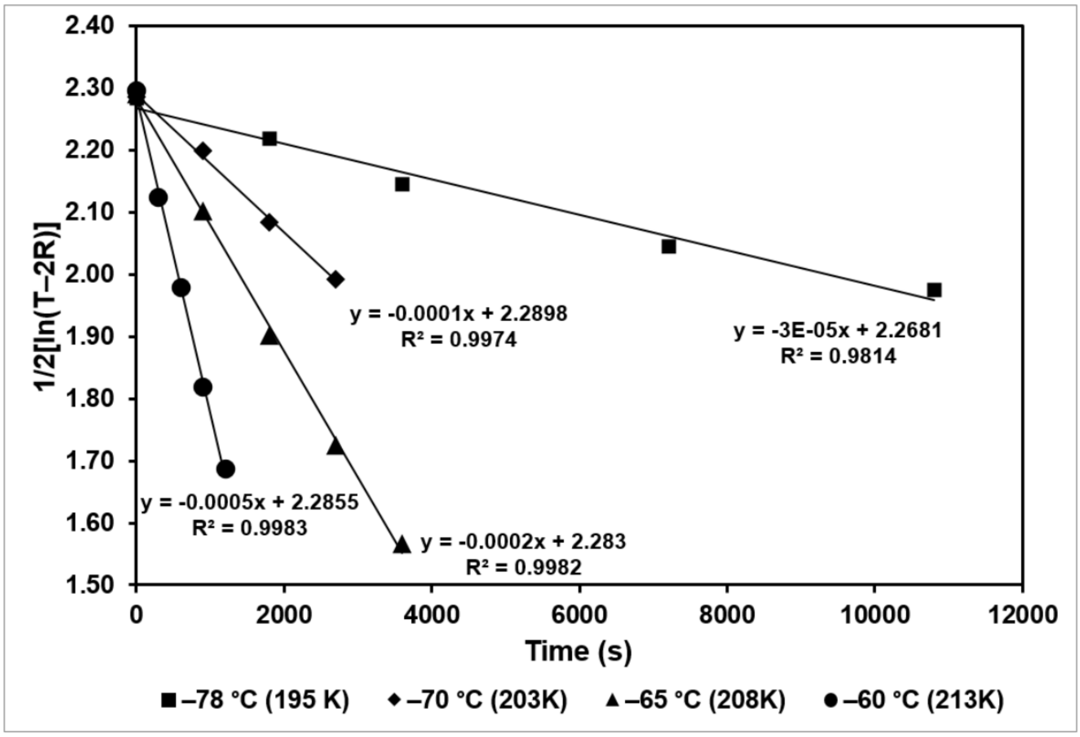

Chart S1. Log plot for the racemization of Li-**4a** with 1.2 equiv. of TMEDA in TBME.

Table S2. Parameters for enantiomerization of (*R*)-Li-**4a** with 1.2 equiv. of TMEDA in TBME.

	Temperature (K)			
**Parameter**	**195**	**203**	**208**	**213**
k_1	-0.000028615	-0.000110931	-0.000203037	-0.000507544
$\ln(k_1/T)$	-15.73458235	-14.41980848	-13.83966253	-12.94721853
$1/T$ (K^{-1})	0.005128205	0.004926108	0.004807692	0.004694836

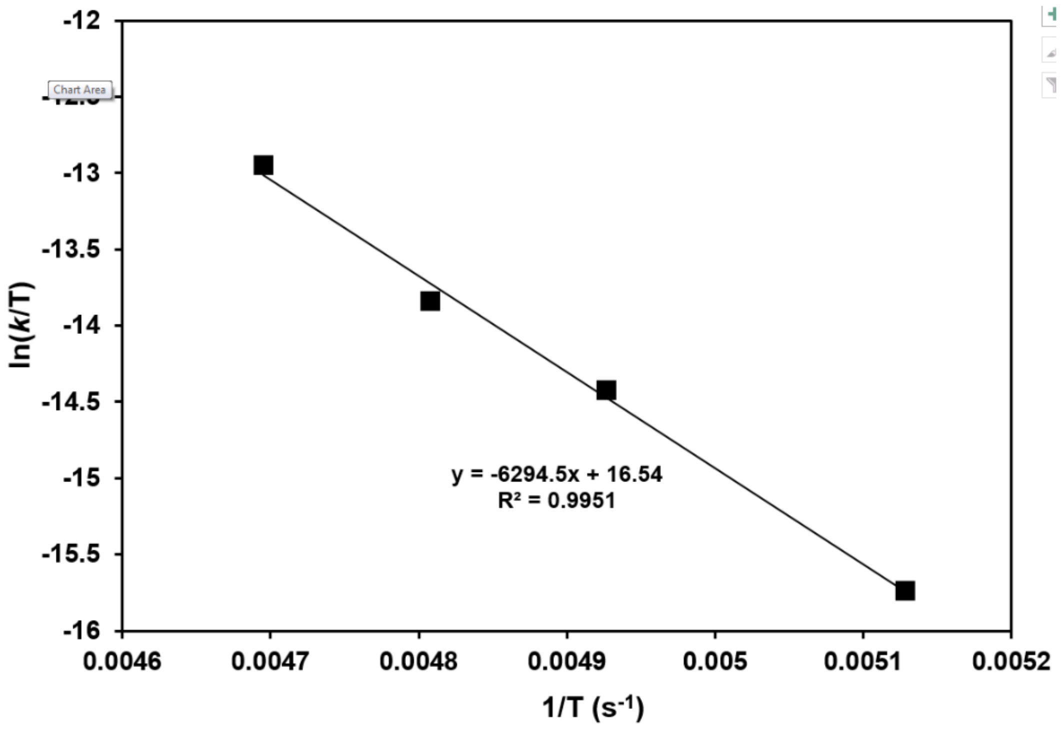

Chart S2. Eyring plot for the enantiomerization of (*R*)-Li-**4a** with 1.2 equiv. of TMEDA in TBME. Slope = -6294.544788; $\Delta H^{\ddagger}$ = –slope x R

Intercept = 16.5400357; $\Delta S^{\ddagger}$ = R x [intercept – $\ln(k_B/h)$

Table S3. Activation parameters for enantiomerization of (*R*)-Li-**4a** with 1.2 equiv. of TMEDA in TBME.

**$\Delta H^{\ddagger}$ (kcal/mol)**	+12.5 ± 0.6
**$\Delta S^{\ddagger}$ (cal/mol•K)**	+14.3 ± 3.0
**$\Delta G^{\ddagger}$ (kcal/mol) at 195K**	+9.7 ± 0.8

3.5 In situ IR studies

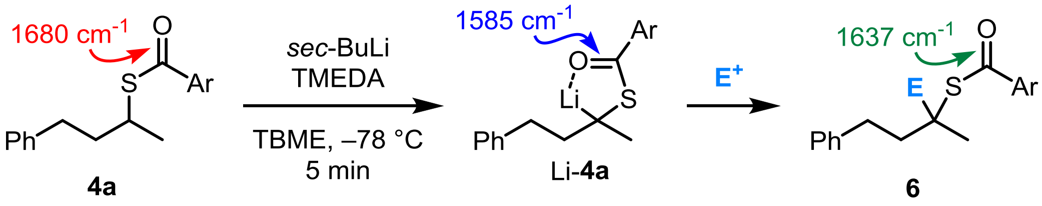

Ar = 2,4,6-triisopropylbenzene

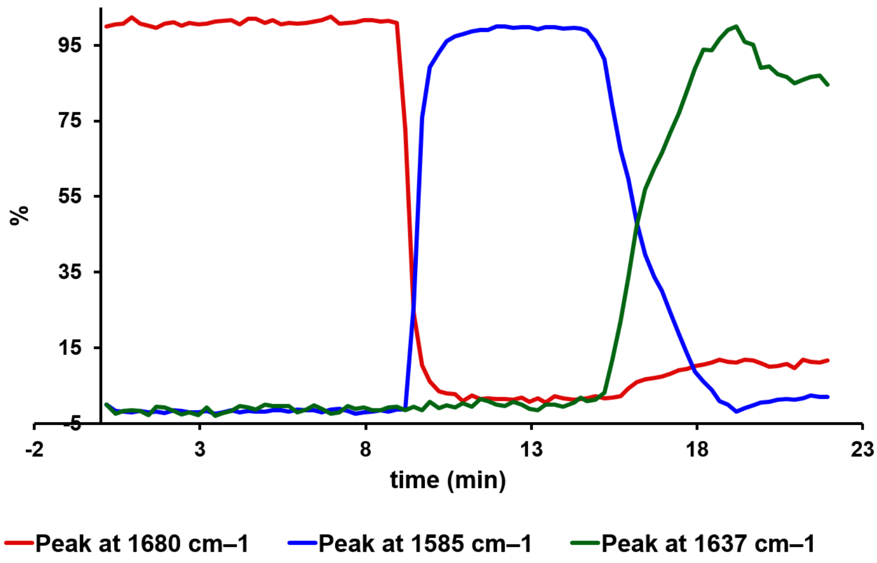

Figure S7. In situ IR studies in the trapping of Li-**4a** with $ClCO_2Me$.

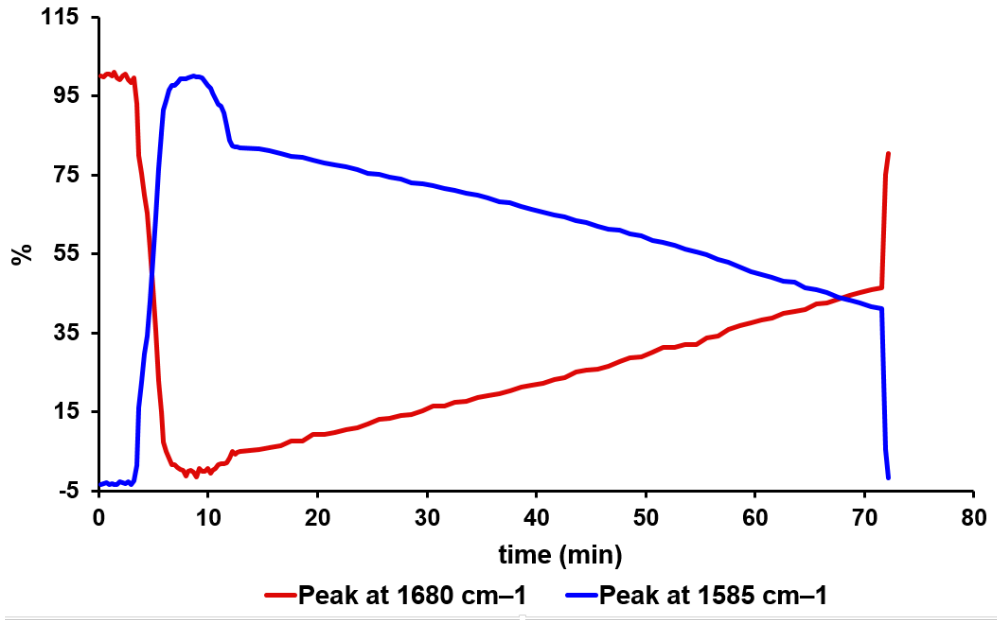

Figure S8. In situ IR studies in the trapping of Li-**4a** with $ClSiMe_3$.

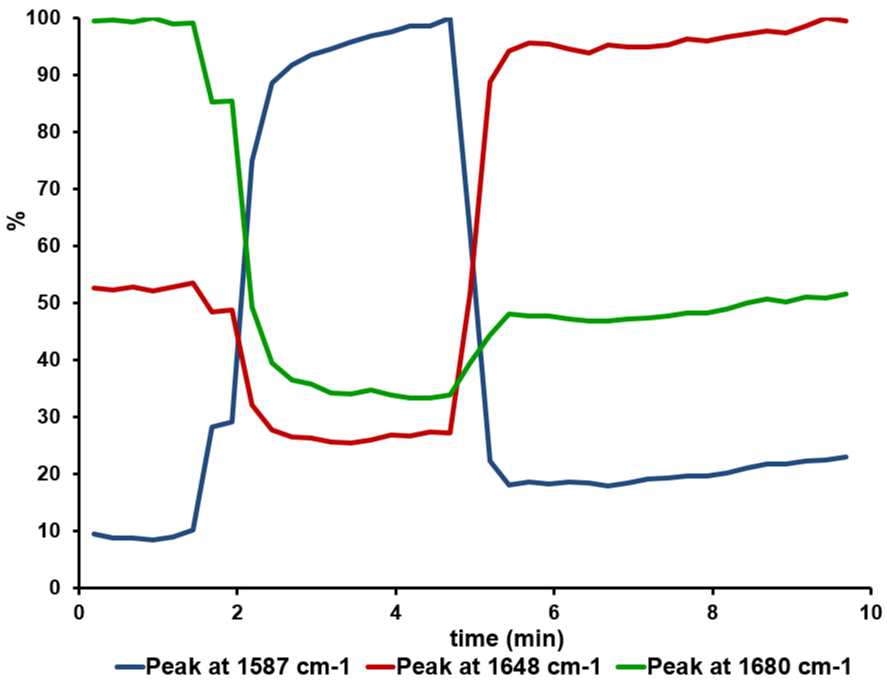

Figure S9. In situ IR studies in the trapping of Li-**4a** with $ClSnMe_3$.

4 REFERENCES

[1] T. R. Hoye, B. M. Eklov, M. Voloshin, *Org. Lett*. **2004**, *6*, 2576.
[2] A. P. Pulis, D. J. Blair, E. Torres and V. K. Aggarwal, *J. Am. Chem. Soc*. **2013**, *135*, 16054.
[3] G. Bifulco, P. Dambruoso, L. Gomez-Paloma, R. Riccio, *Chem. Rev*. **2017**, *107*, 3744.
[4] K. Zangger, I. M. Armitage, *Magn. Reson. Chem*. **2000**, *38*, 452.
[5] I. Coldham, D. Leonori, T. K. Beng, R. E. Gawley *Chem. Commun.* **2009**, *35*, 5239.

5 NMR Spectra

(*R*)-1-Methoxy-2-methyl-1-oxo-4-phenylbutan-2-yl 2,4,6-triisopropylbenzoate 2aa 53

(*R*)-2-Methyl-1-oxo-1,4-diphenylbutan-2-yl 2,4,6-triisopropylbenzoate 2ab 54

(*R*)-1-(Benzylamino)-2-methyl-1-oxo-4-phenylbutan-2-yl 2,4,6-triisopropylbenzoate 2ac 55

(*R*)-1-((4-Bromophenyl)amino)-2-methyl-1-oxo-4-phenylbutan-2-yl 2,4,6-triisopropylbenzoate 2ad 56

(3*R*,4*R*)-4-Hydroxy-3,5-dimethyl-1-phenylhexan-3-yl 2,4,6-triisopropylbenzoate 2ae 57

(1*R*,2*R*)-1-Hydroxy-2-methyl-1,4-diphenylbutan-2-yl 2,4,6-triisopropylbenzoate 2af 59

(*S*)-4-Phenyl-2-(tributylstannyl)butan-2-yl 2,4,6-triisopropylbenzoate 2ag 61

(*S*)-4-Phenyl-2-(trimethylstannyl)butan-2-yl 2,4,6-triisopropylbenzoate 2ah 62

(*S*)-4-Phenyl-2-(trimethylstannyl)pentan-2-yl 2,4,6-triisopropylbenzoate 2ba 63

(*S*)-2-(Trimethylstannyl)pentan-2-yl 2,4,6-triisopropylbenzoate 2ca 64

(*R*)-2-(Trimethylstannyl)hex-5-en-2-yl 2,4,6-triisopropylbenzoate 2da 65

(2*S*)-5-((Tetrahydro-2*H*-pyran-2-yl)oxy)-2-(trimethylstannyl)pentan-2-yl 2,4,6-triisopropylbenzoate 2ea 66

(1*R*,2*R*)-2-Methyl-1,4-diphenylbutane-1,2-diol 3 67

(*R*)-(1*R*,2*R*)-2-Hydroxy-2-methyl-1,4-diphenylbutyl 3,3,3-trifluoro-2-methoxy-2-phenylpropanoate SI12 68

2,4,6-Triisopropylbenzothioic *S*-acid SI13 69

(*R*)-*S*-(4-Phenylbutan-2-yl) 2,4,6-triisopropylbenzothioate 4a 70

(*R*)-*S*-(1-Phenylpentan-3-yl) 2,4,6-triisopropylbenzothioate 4b 71

(*R*)-S-Pentan-2-yl 2,4,6-triisopropylbenzothioate 4c 72

(*R*)-*S*-Hex-5-en-2-yl 2,4,6-triisopropylbenzothioate 4d 73

(*R*)-*S*-(5-Hydroxypentan-2-yl) 2,4,6-triisopropylbenzothioate SI16 74

S-((3*R*)-5-((Tetrahydro-2*H*-pyran-2-yl)oxy)pentan-2-yl) 2,4,6-triisopropylbenzothioate 4e 75

S-((3*R*,8*S*,9*S*,10*R*,13*R*,14*S*,17*R*)-10,13-Dimethyl-17-((*R*)-6-methylheptan-2-yl)-2,3,4,7,8,9,10,11,12,13,14,15,16,17-tetradecahydro-1*H*-cyclopenta[a]phenanthren-3-yl) 2,4,6-triisopropylbenzothioate 8 75

Methyl (*R*)-2-methyl-4-phenyl-2-((2,4,6-triisopropylbenzoyl)thio)butanoate 6aa 77

(*R*)-*S*-(1-((4-Bromophenyl)amino)-2-methyl-1-oxo-4-phenylbutan-2-yl) 2,4,6-triisopropylbenzothioate 6ab 78

(*R*)-*S*-(2-Methyl-1-oxo-4-phenylbutan-2-yl) 2,4,6-triisopropylbenzothioate 6ac 79

S-((1*R*,2*R*)-1-Hydroxy-2-methyl-1,4-diphenylbutan-2-yl) 2,4,6-triisopropylbenzothioate 6ad – Diastereomer A 80

S-((1*R*,2*R*)-1-Hydroxy-2-methyl-1,4-diphenylbutan-2-yl) 2,4,6-triisopropylbenzothioate 6ad – Diastereomer B 81

(*R*)-*S*-(4-Phenyl-2-(trimethylsilyl)butan-2-yl) 2,4,6-triisopropylbenzothioate 6ae 82

(*R*)-*S*-(4-Phenyl-2-(tributylstannyl)butan-2-yl) 2,4,6-triisopropylbenzothioate 6af 83

(S)-S-(4-phenyl-2-(trimethylstannyl)butan-2-yl) 2,4,6-triisopropylbenzothioate 6ag 84

(*R*)-2-Methyl-4-phenyl-2-((2,4,6-triisopropylbenzoyl)thio)butanoic acid 6ah 85

(*R*)-*S*-(3-((4-Bromophenyl)carbamoyl)-1-phenylpentan-3-yl) 2,4,6-triisopropylbenzothioate 6ba 86

(*R*)-Methyl 2-methyl-2-((2,4,6-triisopropylbenzoyl)thio)pentanoate 6ca 87

(*R*)-*S*-(1-((4-Bromophenyl)amino)-2-methyl-1-oxohex-5-en-2-yl) 2-ethyl-4,6-diisopropylbenzothioate 6da 88

S-((2*R*)-1-((4-Bromophenyl)amino)-2-methyl-1-oxo-5-((tetrahydro-2*H*-pyran-2-yl)oxy)pentan-2-yl) 2,4,6-triisopropylbenzothioate 6ea 89

Methyl (3R,8S,9S,10R,13R,14S,17R)-10,13-dimethyl-17-((R)-6-methylheptan-2-yl)-3-((2,4,6-triisopropylbenzoyl)thio)-2,3,4,7,8,9,10,11,12,13,14,15,16,17-tetradecahydro-1H-cyclopenta[a]phenanthrene-3-carboxylate 9 90

(1*R*,2*R*)-2-Mercapto-2-methyl-1,4-diphenylbutan-1-ol 7 91

(*R*)-1-Methoxy-2-methyl-1-oxo-4-phenylbutan-2-yl 2,4,6-triisopropylbenzoate 2aa

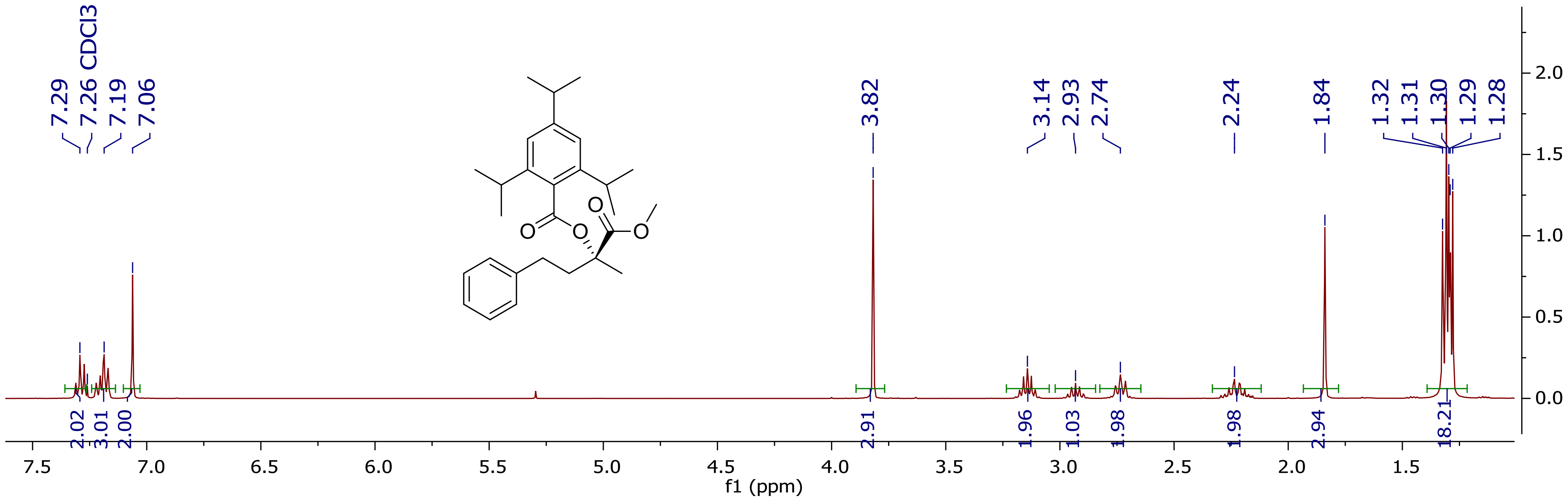

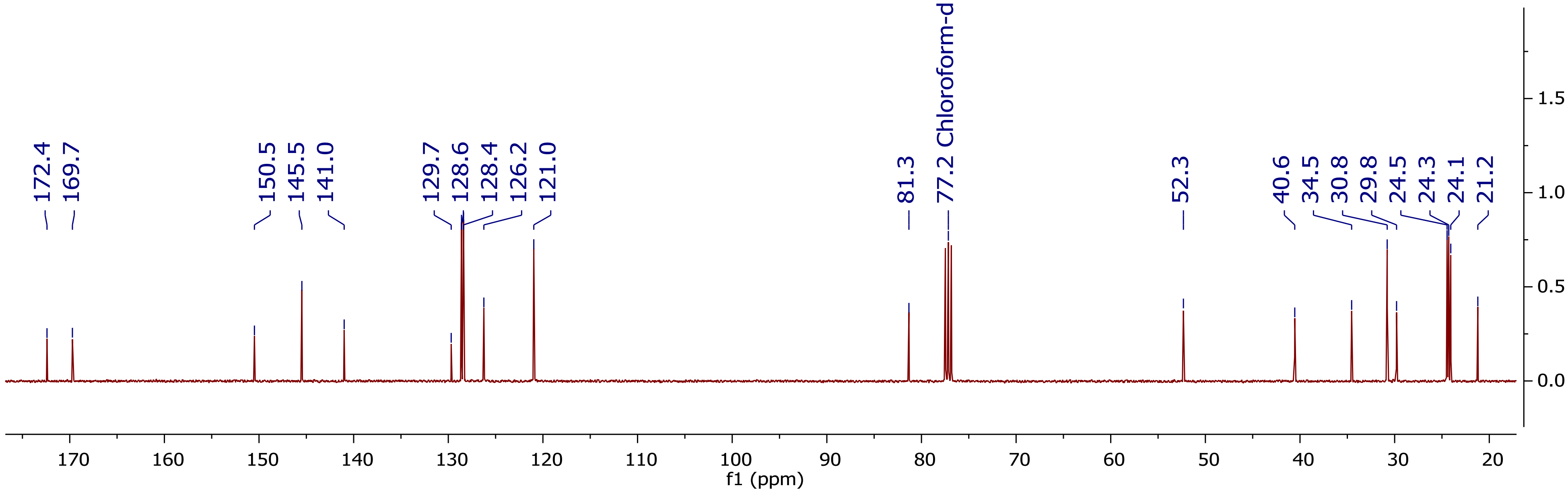

(*R*)-2-Methyl-1-oxo-1,4-diphenylbutan-2-yl 2,4,6-triisopropylbenzoate 2ab

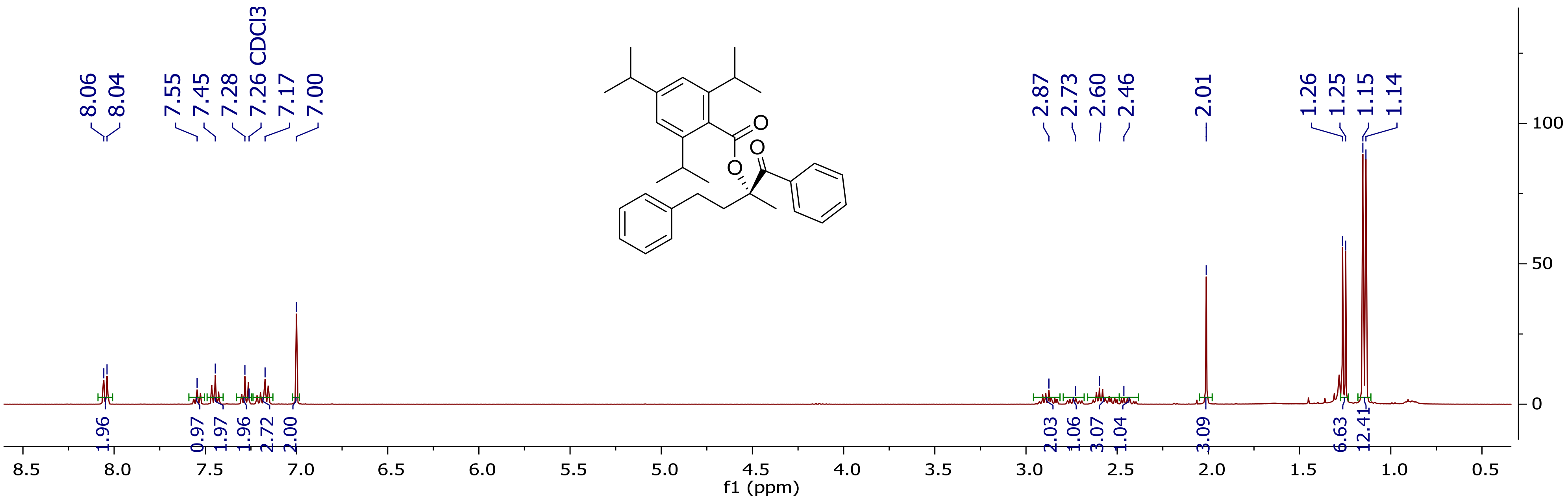

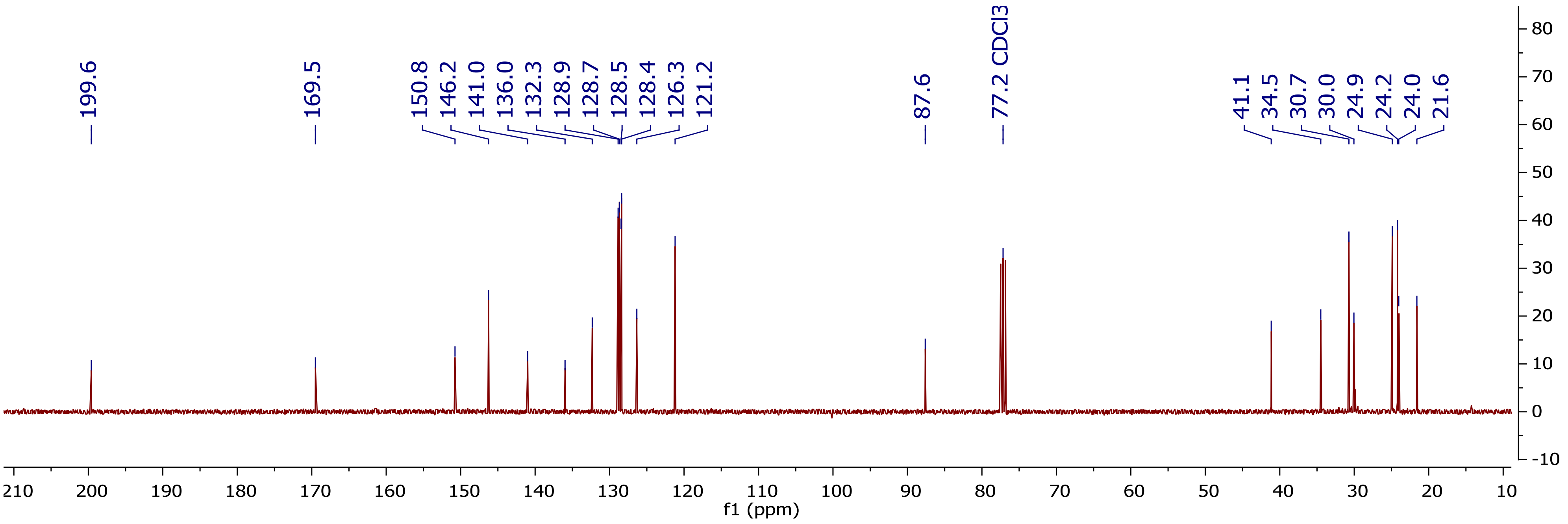

(*R*)-1-(Benzylamino)-2-methyl-1-oxo-4-phenylbutan-2-yl 2,4,6-triisopropylbenzoate 2ac

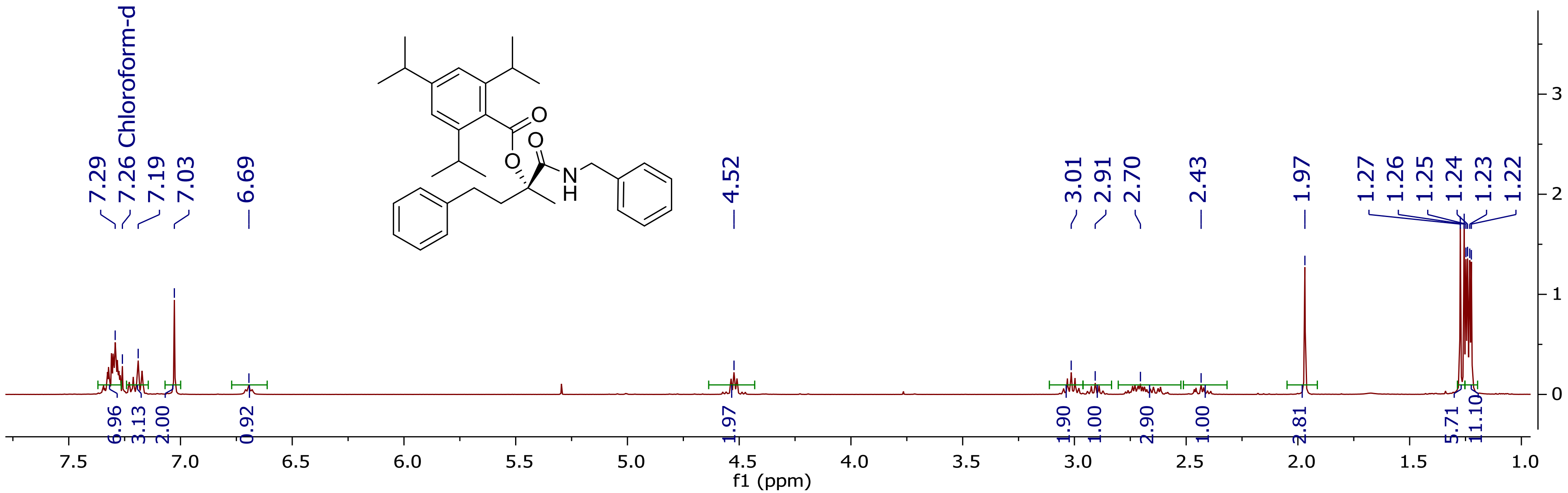

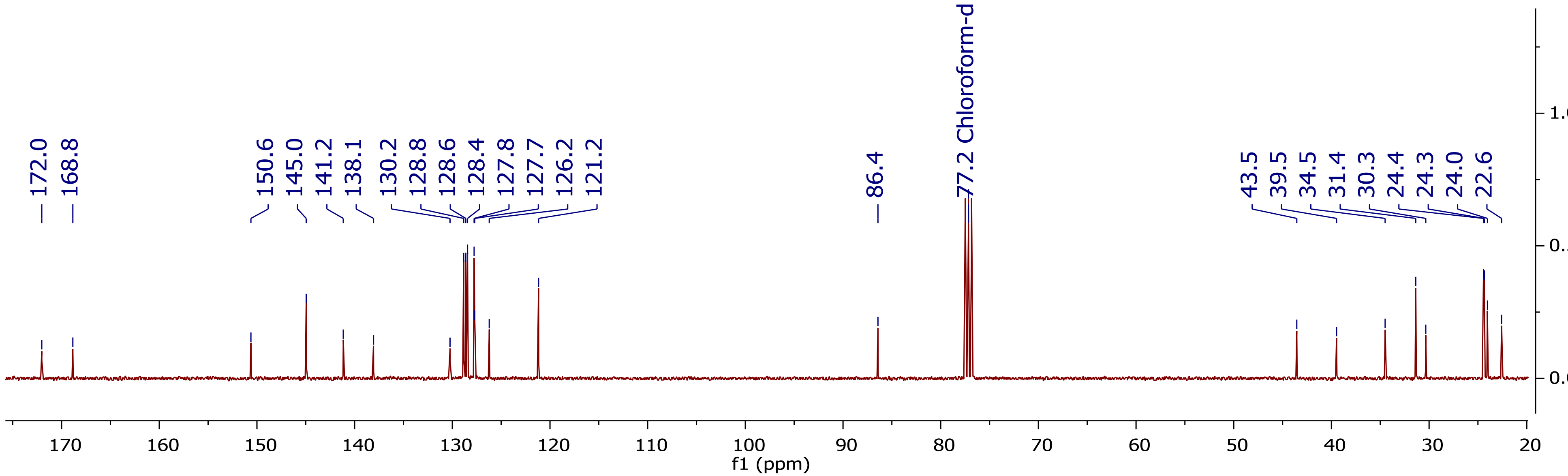

(*R*)-1-((4-Bromophenyl)amino)-2-methyl-1-oxo-4-phenylbutan-2-yl 2,4,6-triisopropylbenzoate 2ad

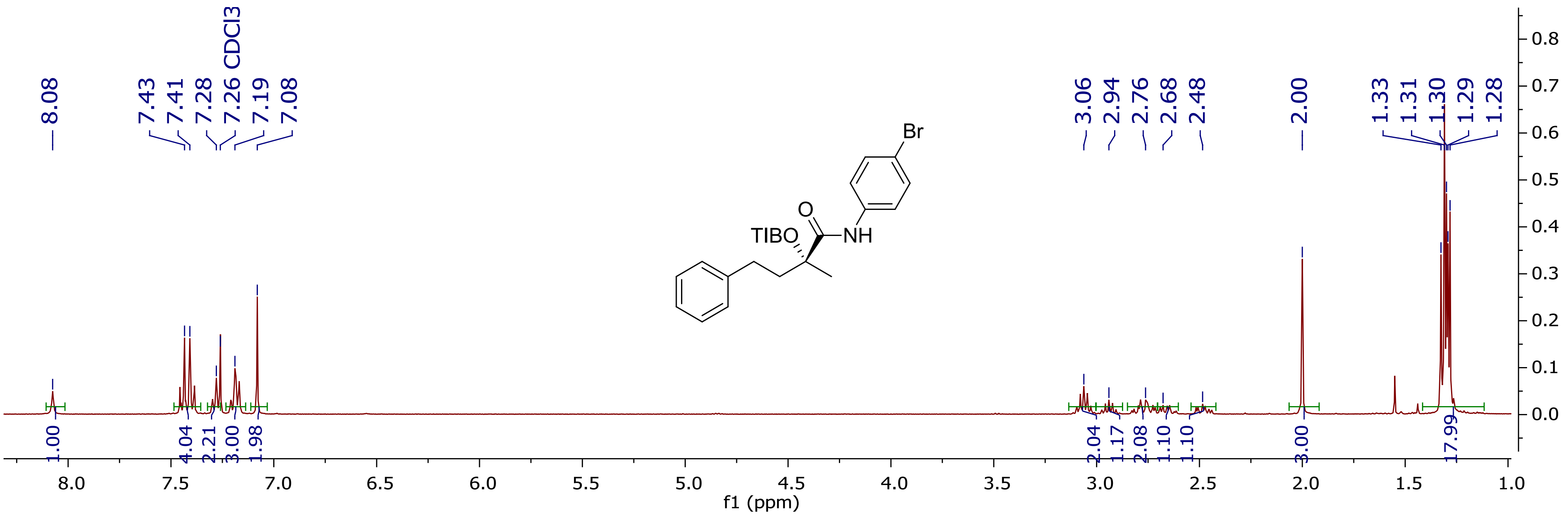

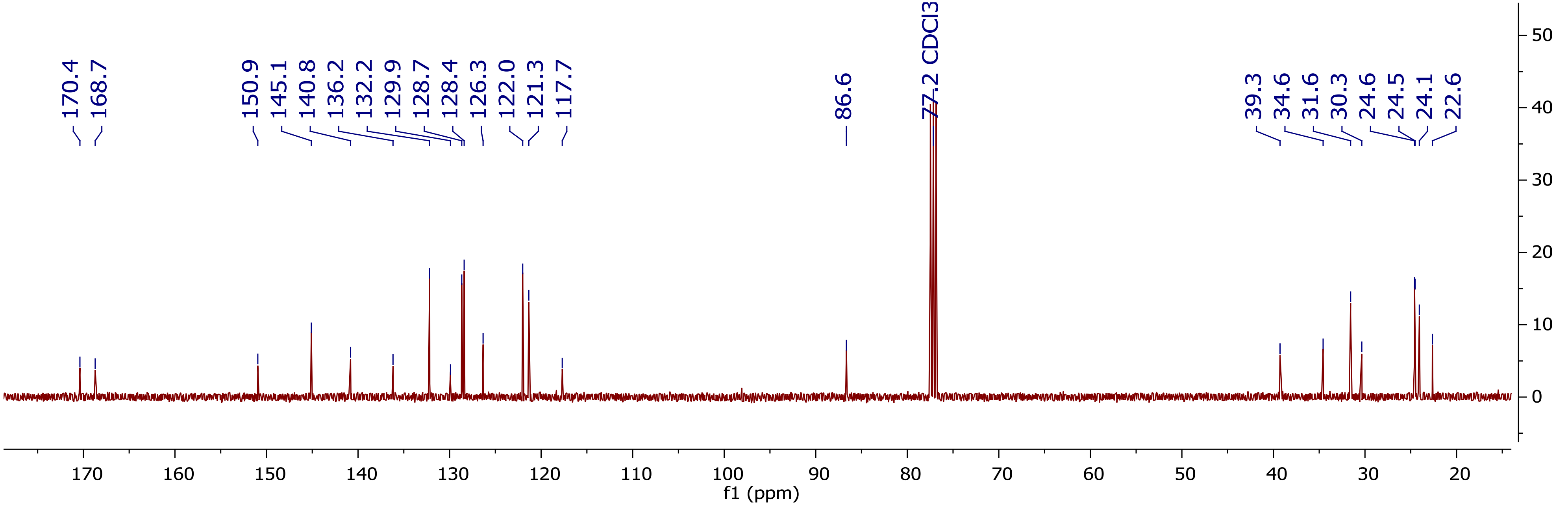

(3*R*,4*R*)-4-Hydroxy-3,5-dimethyl-1-phenylhexan-3-yl 2,4,6-triisopropylbenzoate 2ae

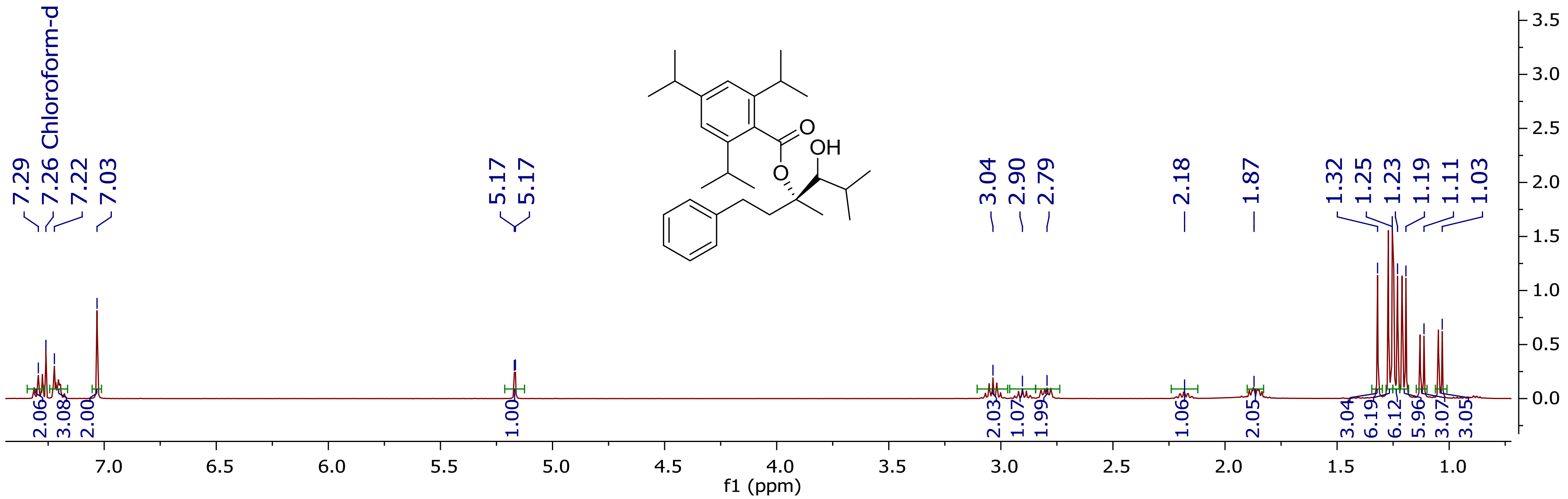

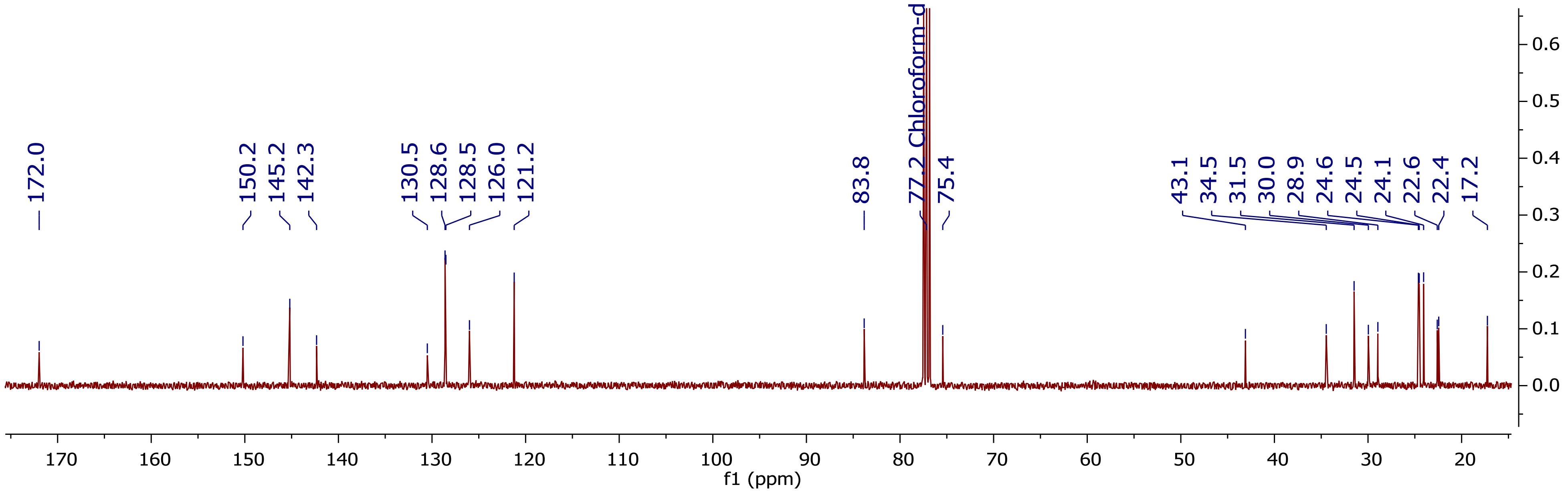

Crude NMR

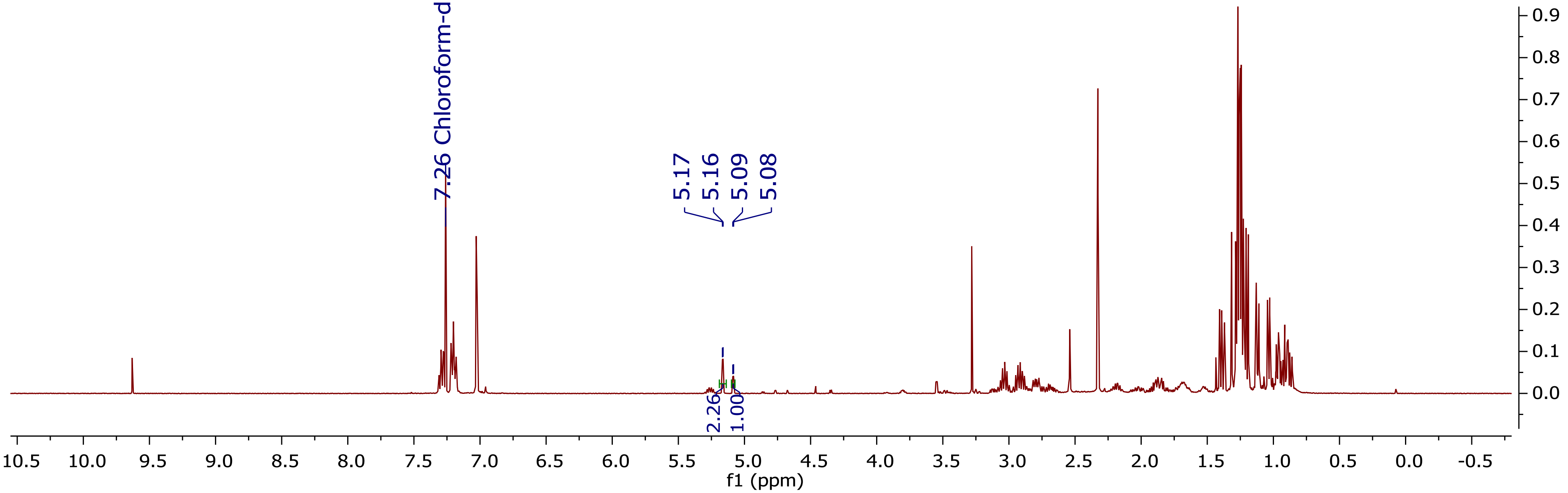

7.26 Chloroform-d
5.17
5.16
5.09
5.08
2.26
1.00
0.9
0.8
0.7
0.6
0.5
0.4
0.3
0.2
0.1
0.0
10.5 10.0 9.5 9.0 8.5 8.0 7.5 7.0 6.5 6.0 5.5 5.0 4.5 4.0 3.5 3.0 2.5 2.0 1.5 1.0 0.5 0.0 -0.5
f1 (ppm)

(1*R*,2*R*)-1-Hydroxy-2-methyl-1,4-diphenylbutan-2-yl 2,4,6-triisopropylbenzoate 2af

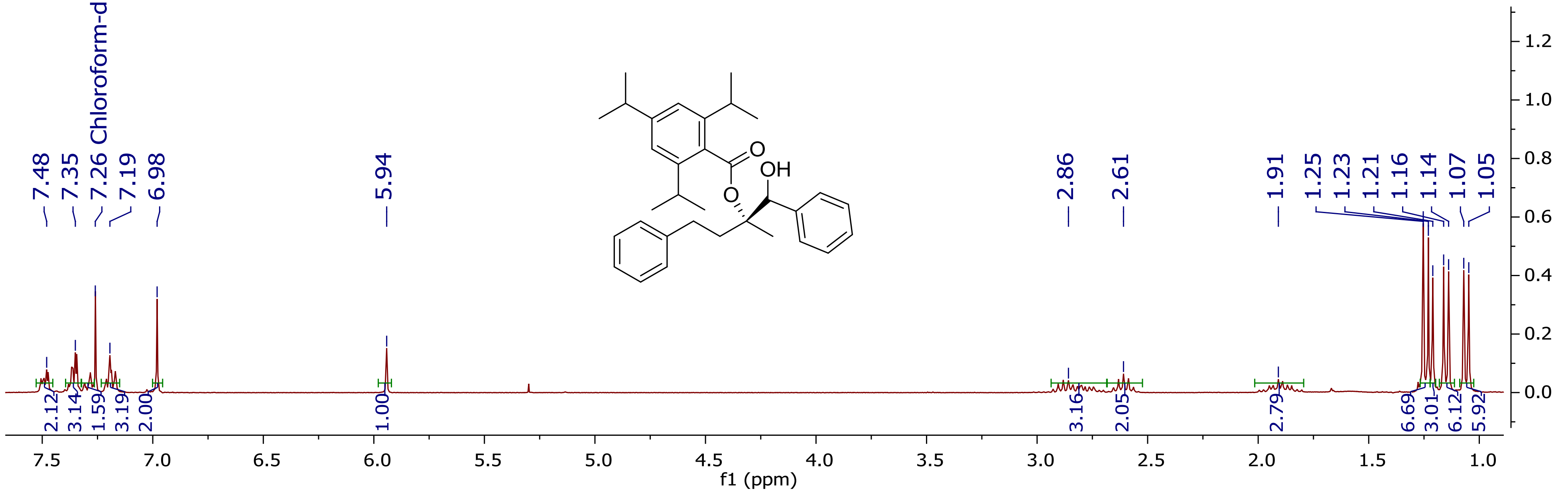

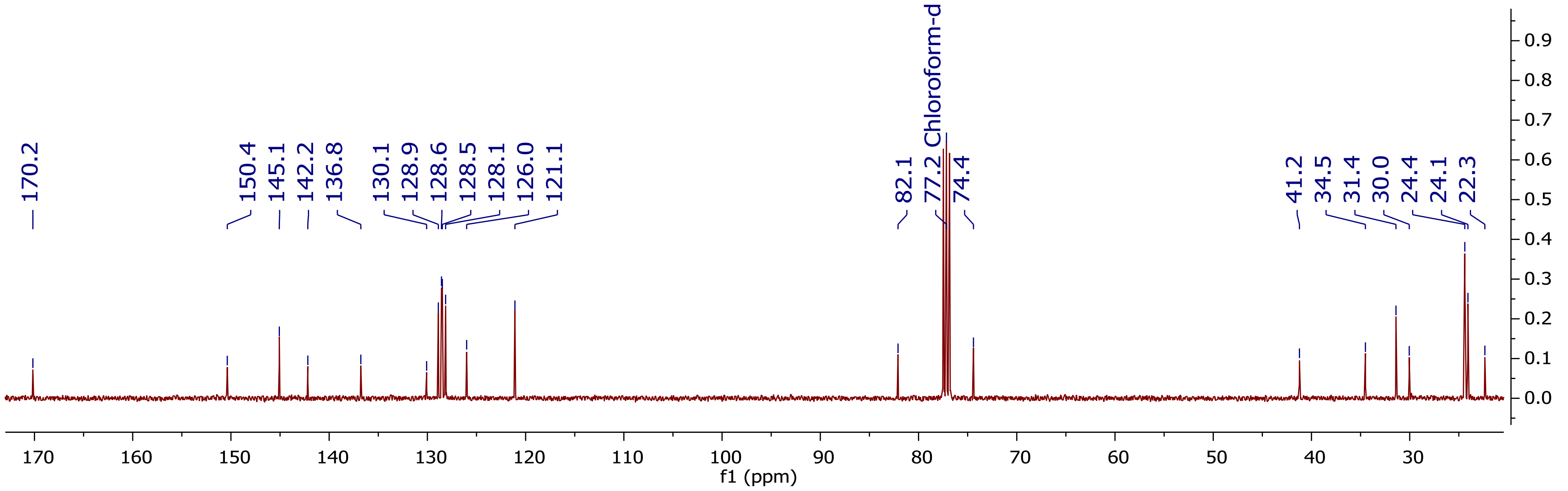

Crude NMR

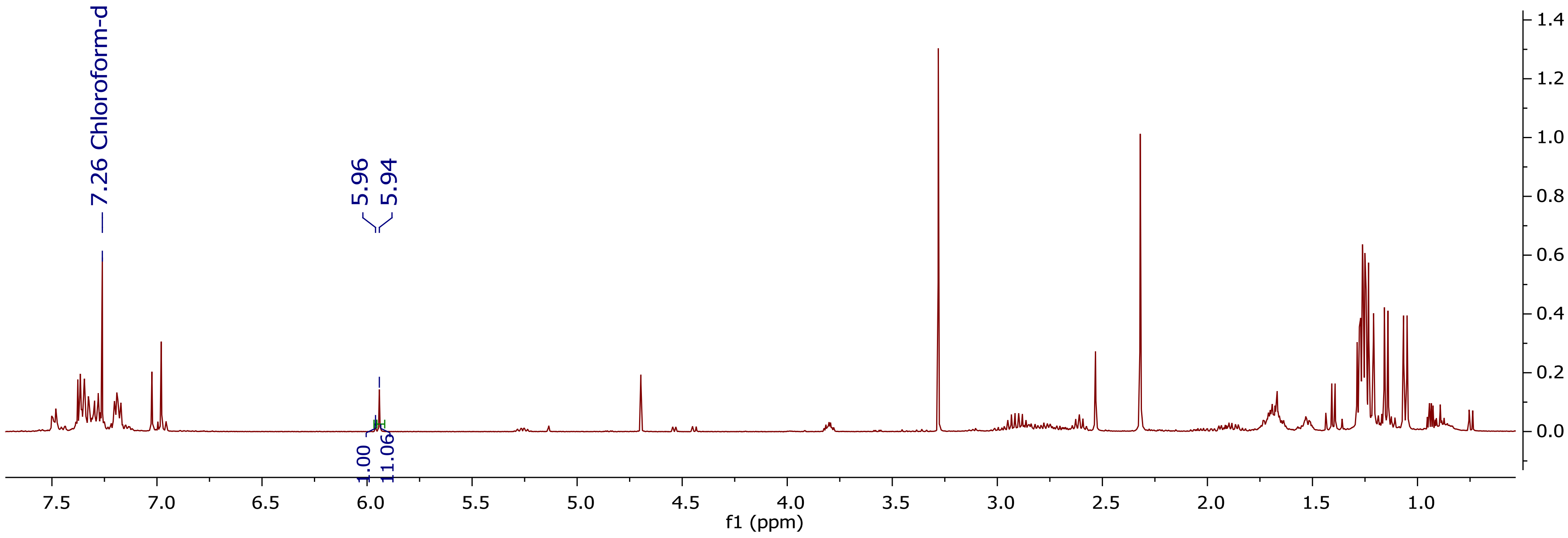

7.26 Chloroform-d
5.96
5.94
1.00
11.06
7.5
7.0
6.5
6.0
5.5
5.0
4.5
4.0
3.5
3.0
2.5
2.0
1.5
1.0
f1 (ppm)
1.4
1.2
1.0
0.8
0.6
0.4
0.2
0.0

(*S*)-4-Phenyl-2-(tributylstannyl)butan-2-yl 2,4,6-triisopropylbenzoate 2ag

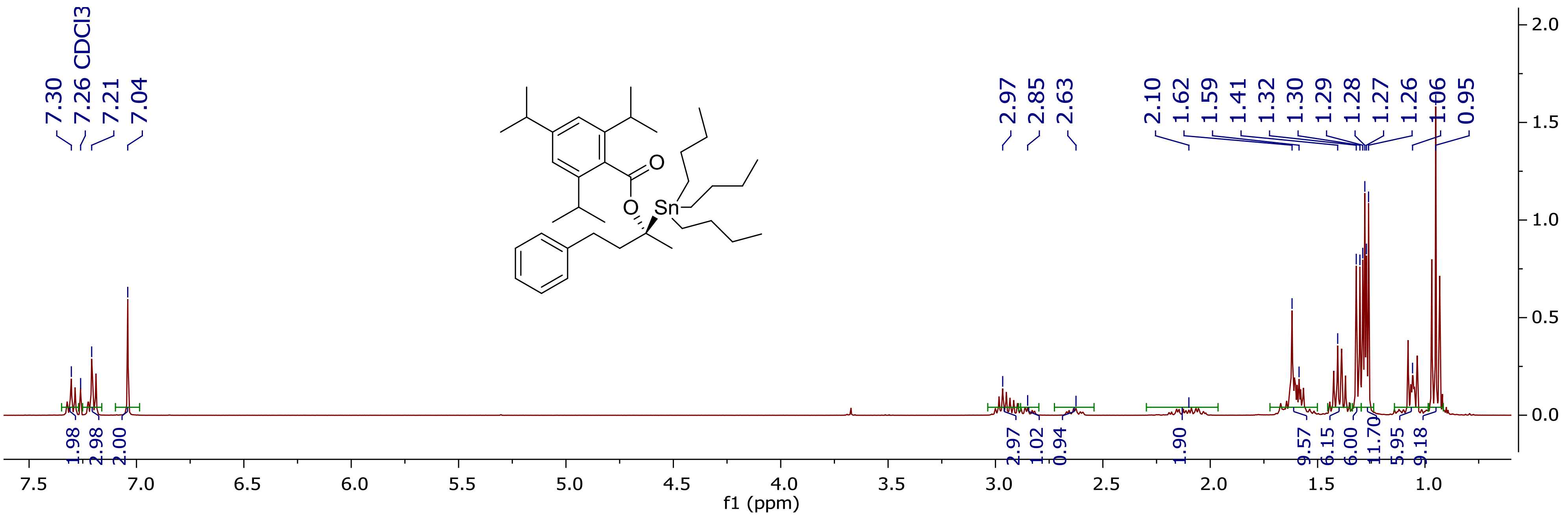

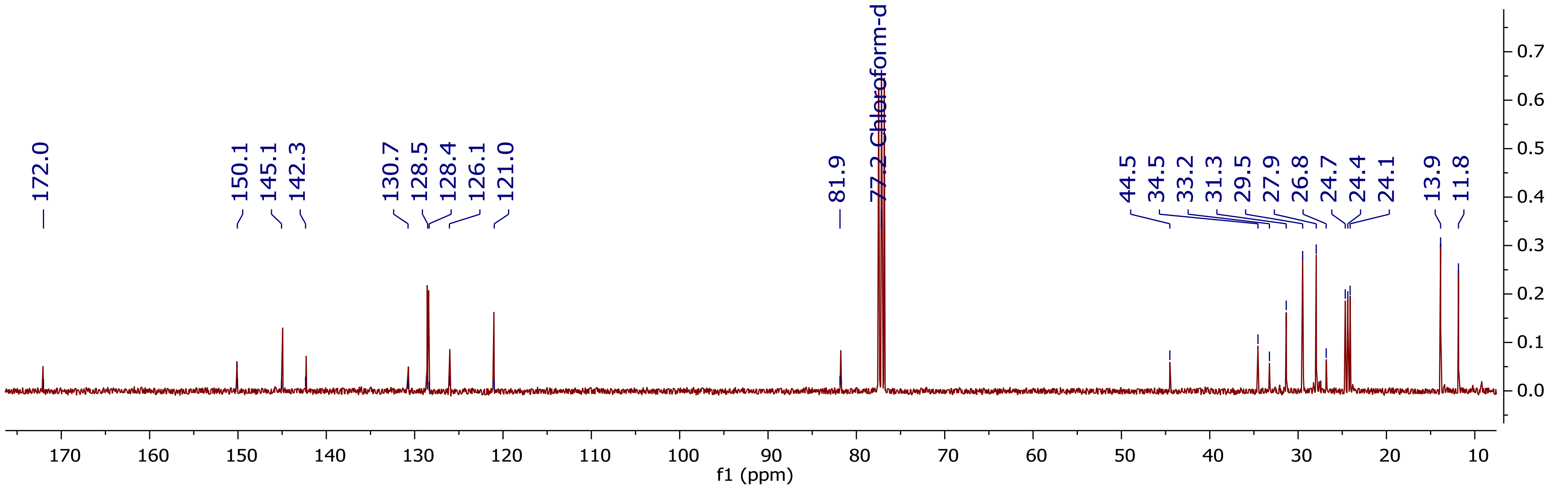

(*S*)-4-Phenyl-2-(trimethylstannyl)butan-2-yl 2,4,6-triisopropylbenzoate 2ah

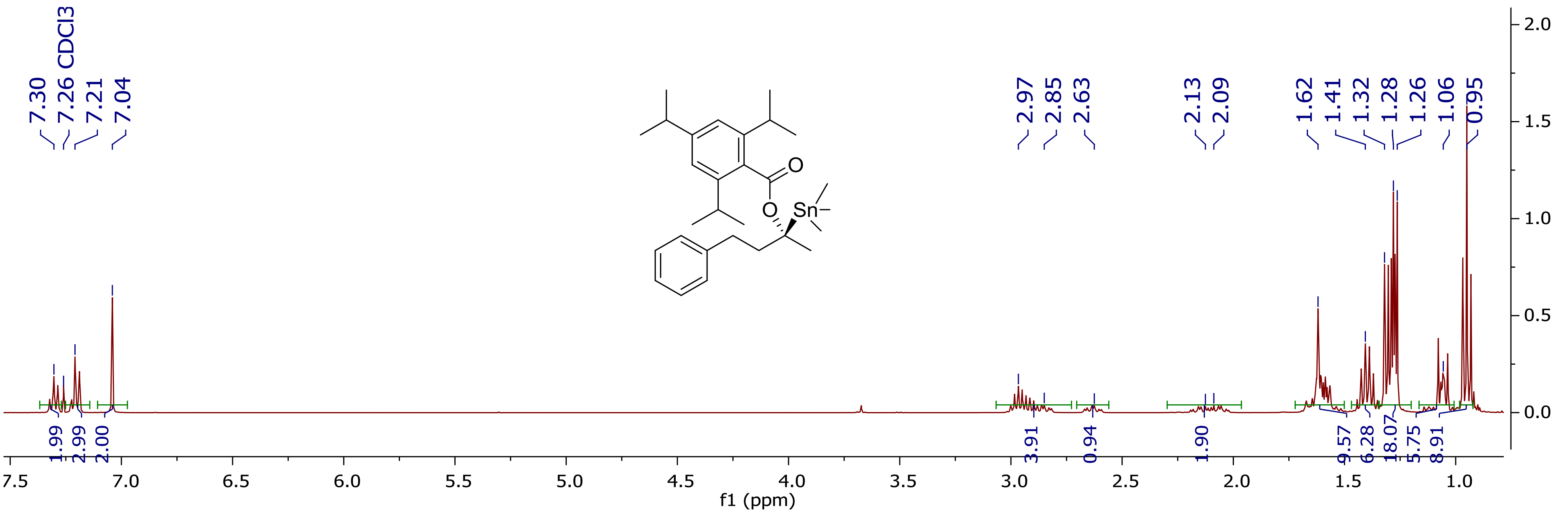

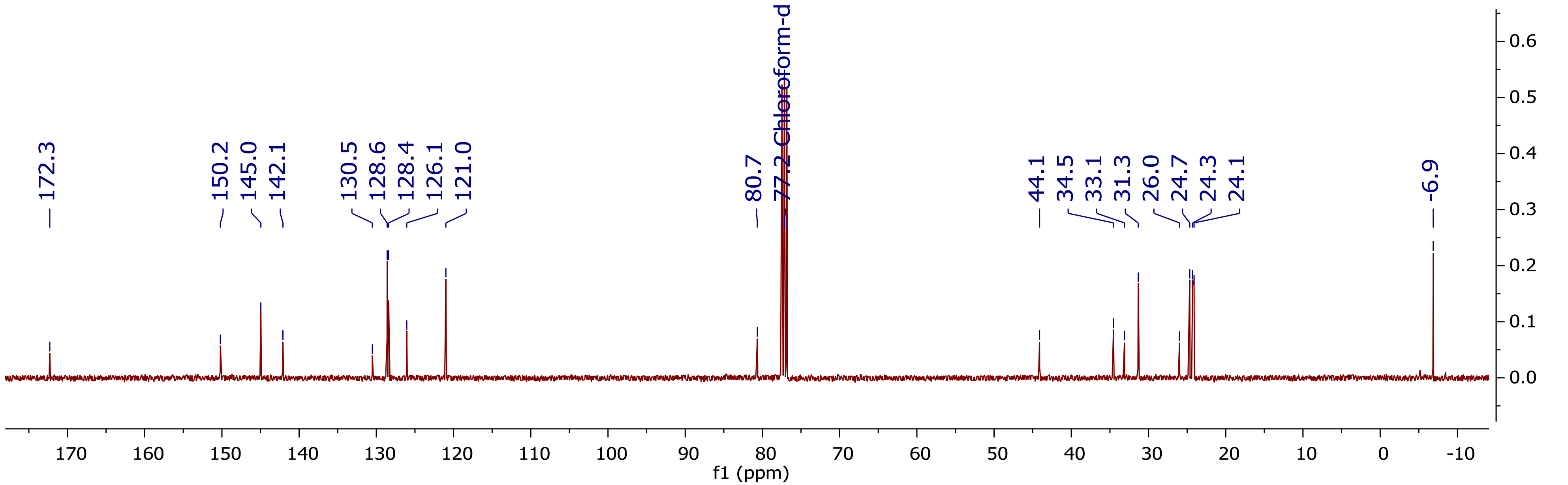

(*S*)-4-Phenyl-2-(trimethylstannyl)pentan-2-yl 2,4,6-triisopropylbenzoate 2ba

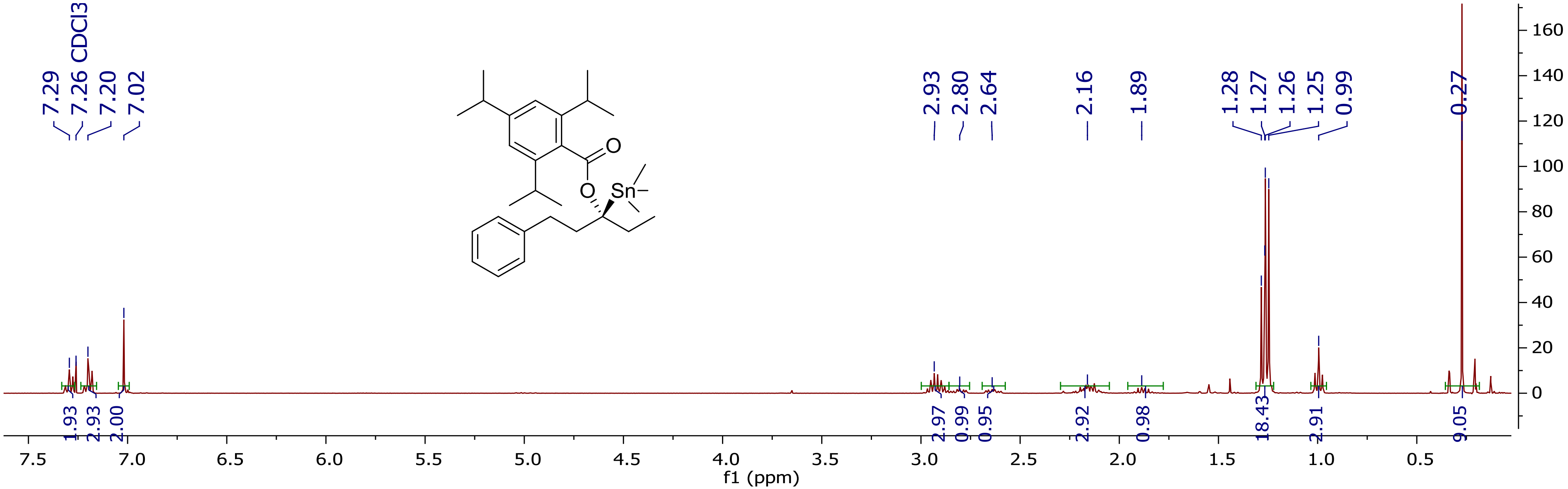

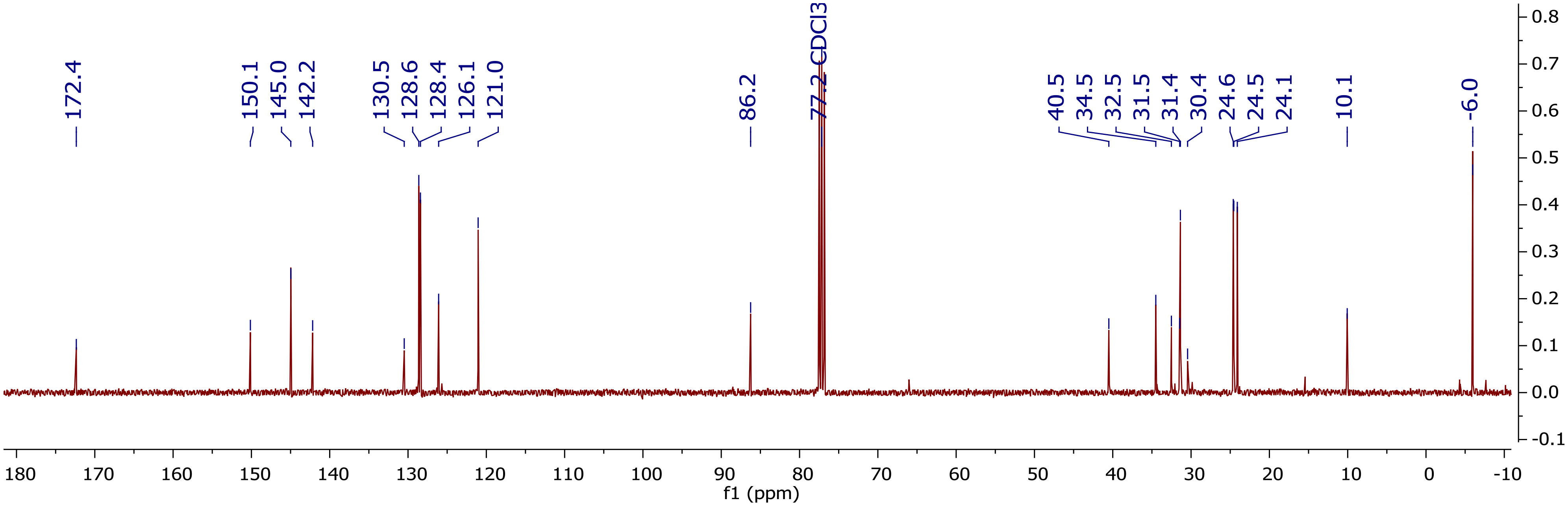

(*S*)-2-(Trimethylstannyl)pentan-2-yl 2,4,6-triisopropylbenzoate 2ca

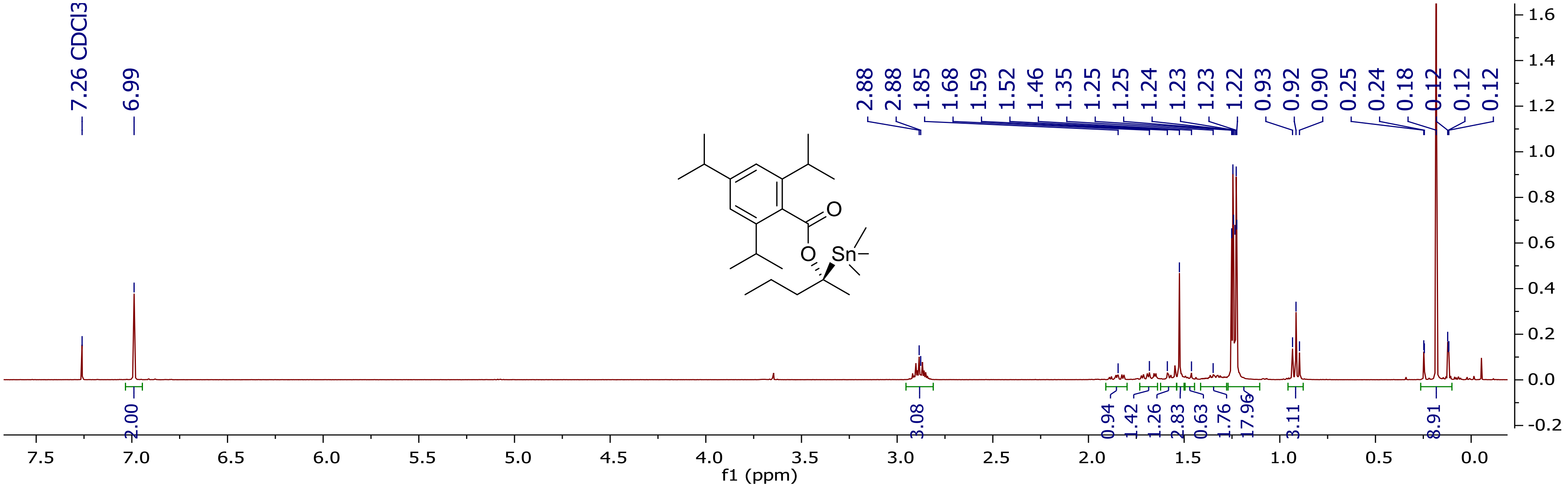

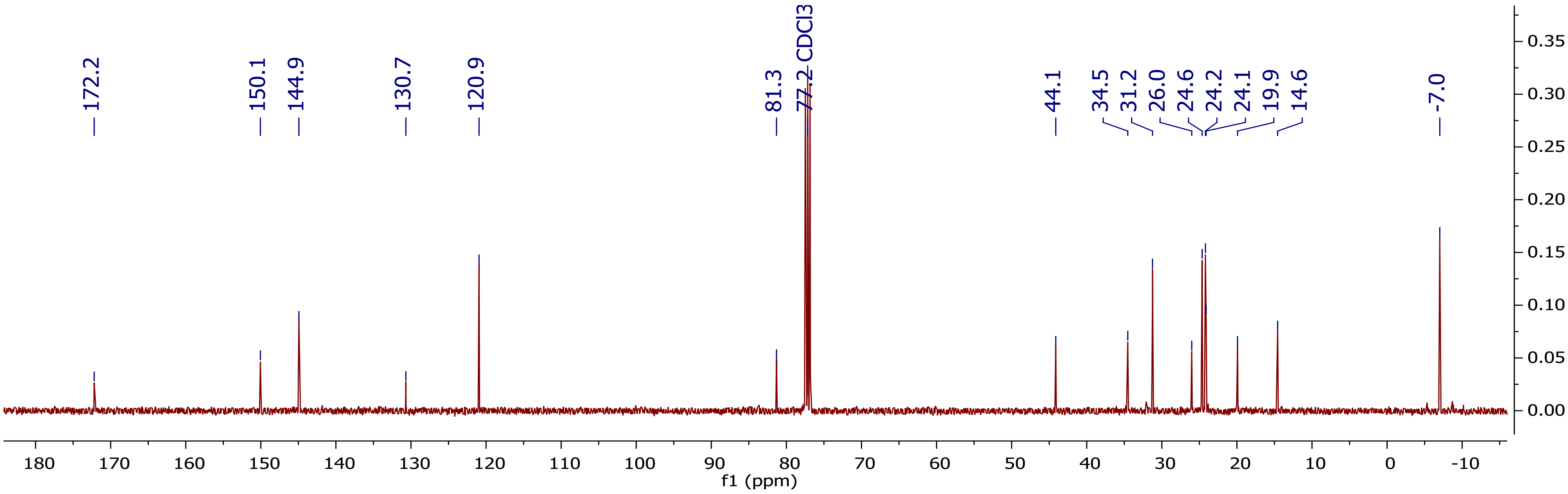

(*R*)-2-(Trimethylstannyl)hex-5-en-2-yl 2,4,6-triisopropylbenzoate 2da

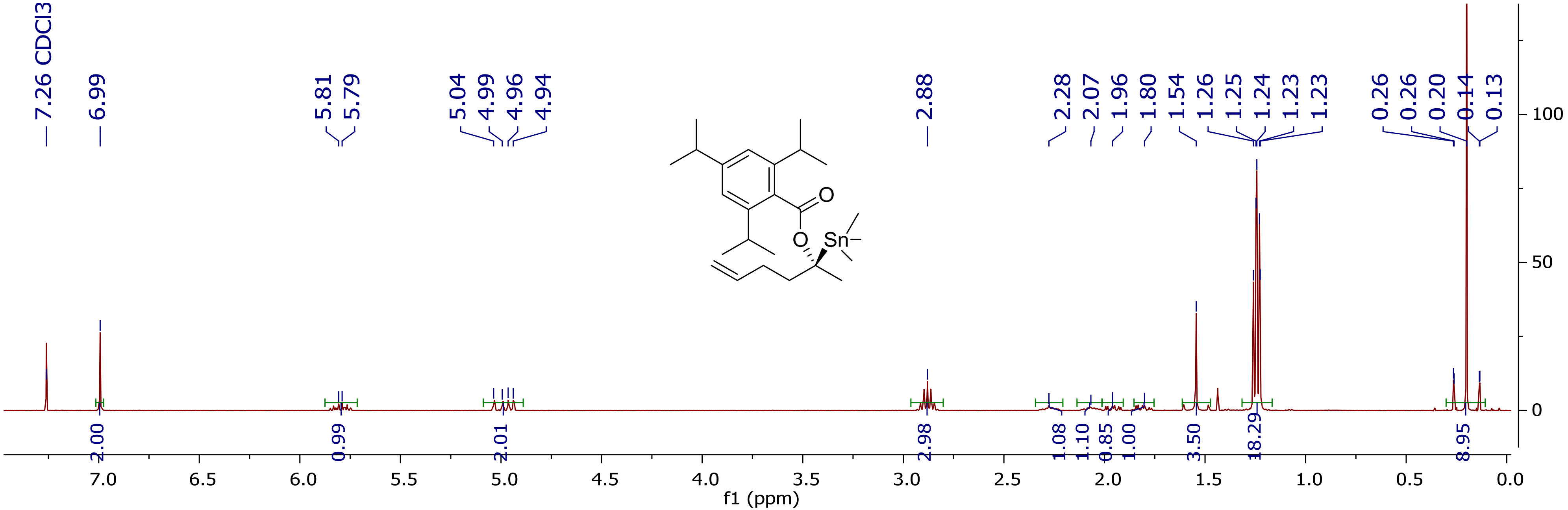

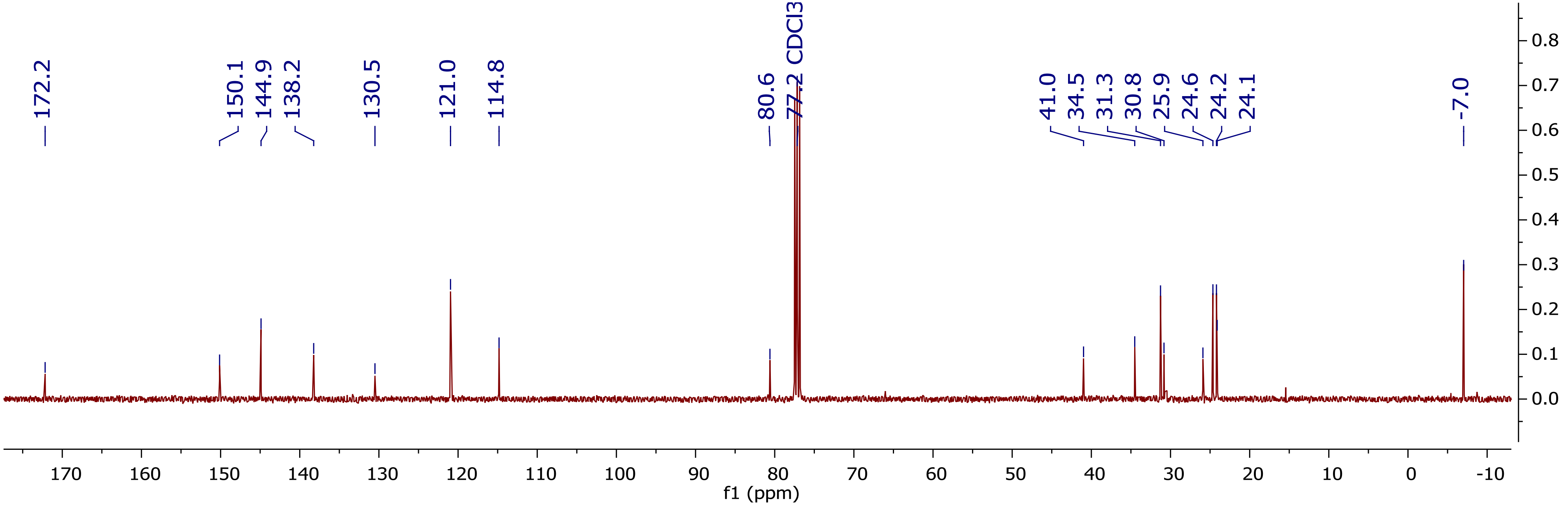

(2*S*)-5-((Tetrahydro-2*H*-pyran-2-yl)oxy)-2-(trimethylstannyl)pentan-2-yl 2,4,6-triisopropylbenzoate 2ea

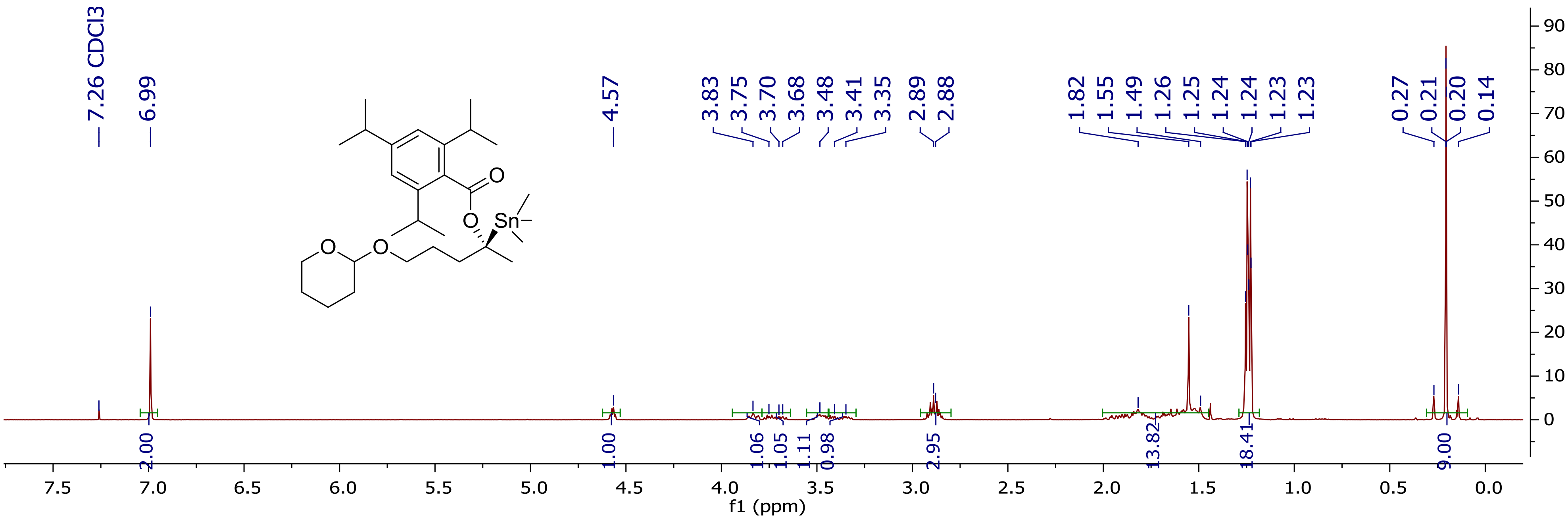

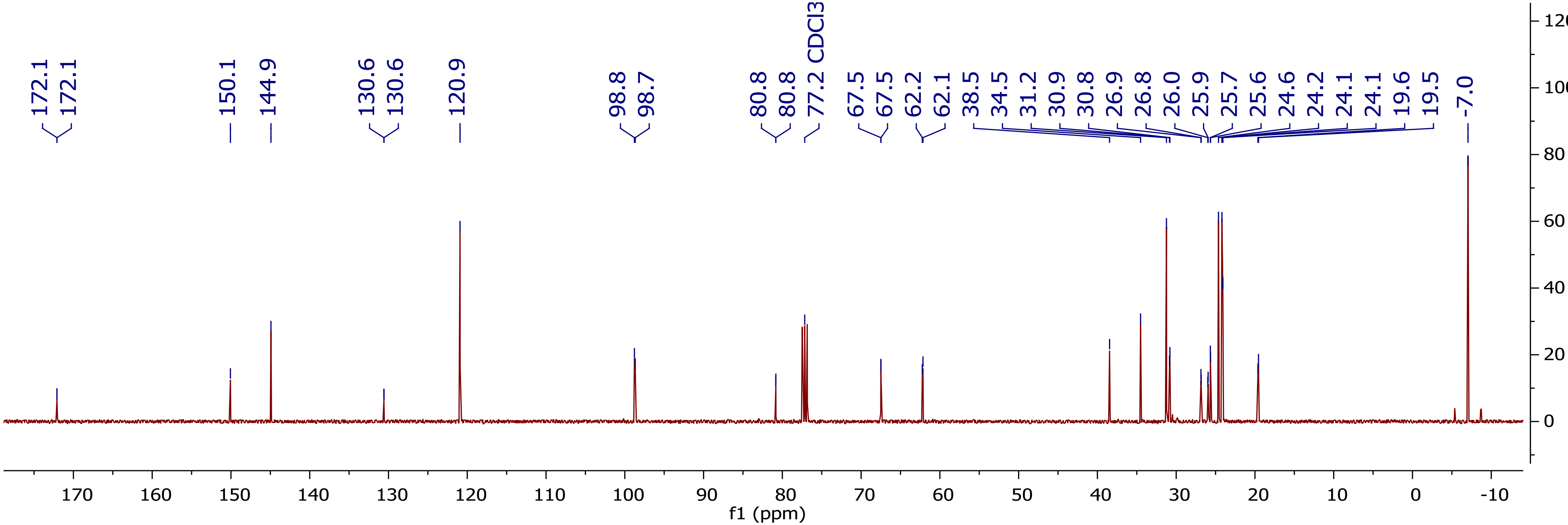

(1*R*,2*R*)-2-Methyl-1,4-diphenylbutane-1,2-diol 3

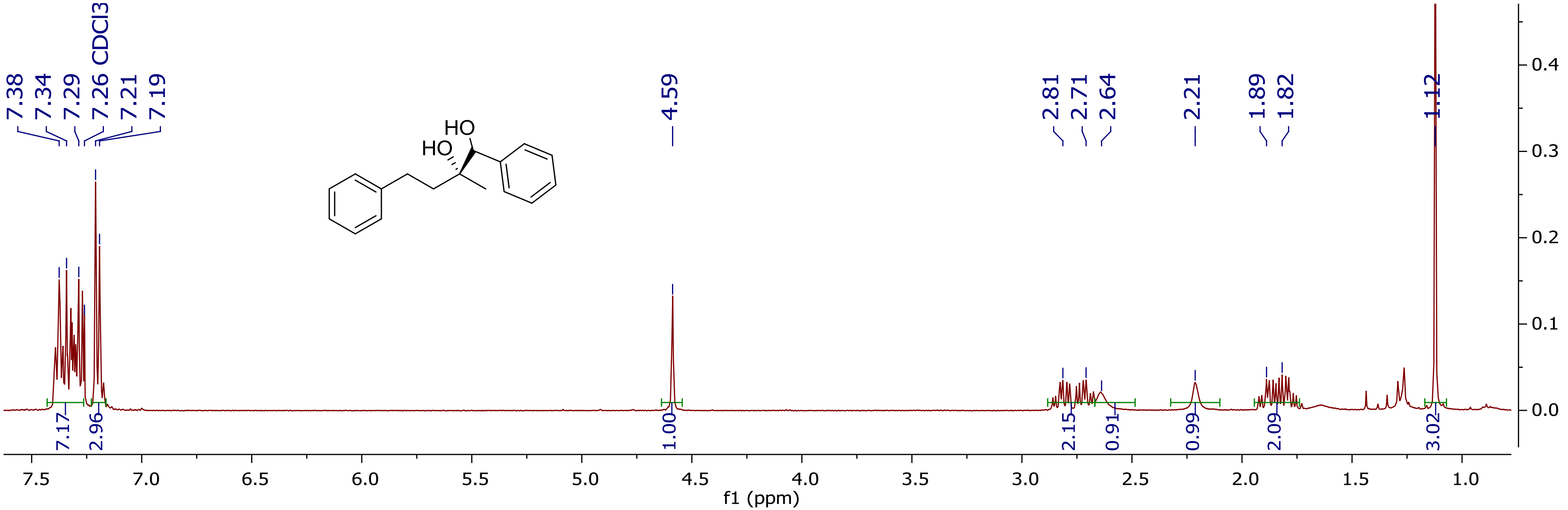

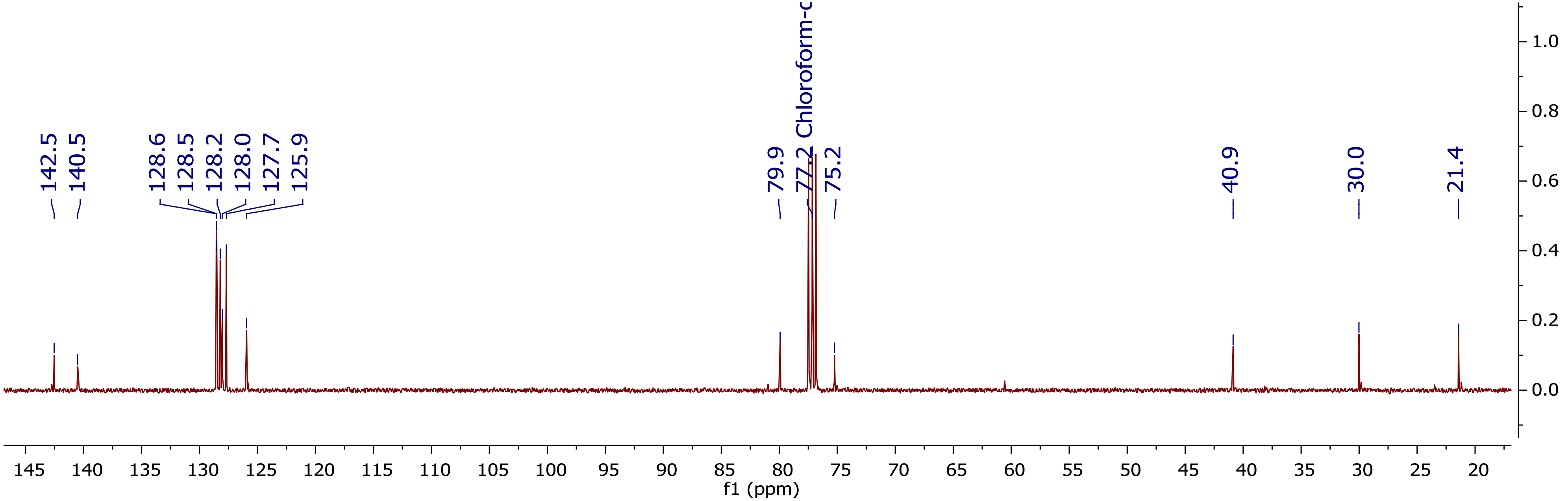

(*R*)-(1*R*,2*R*)-2-Hydroxy-2-methyl-1,4-diphenylbutyl 3,3,3-trifluoro-2-methoxy-2-phenylpropanoate SI12

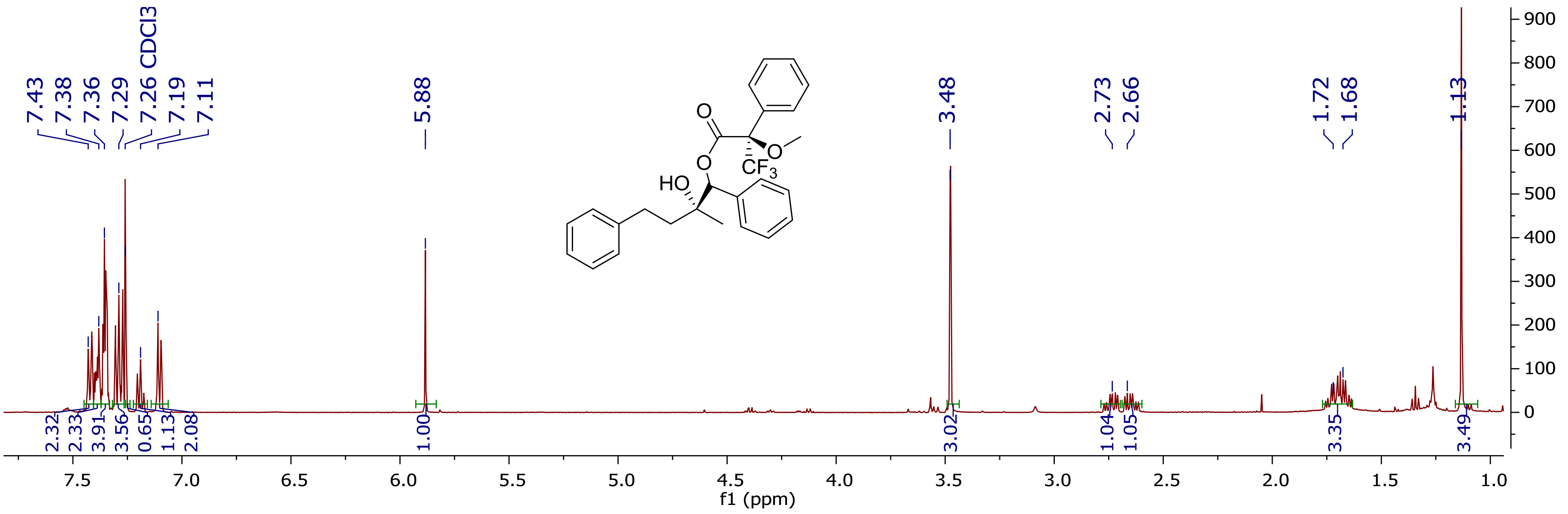

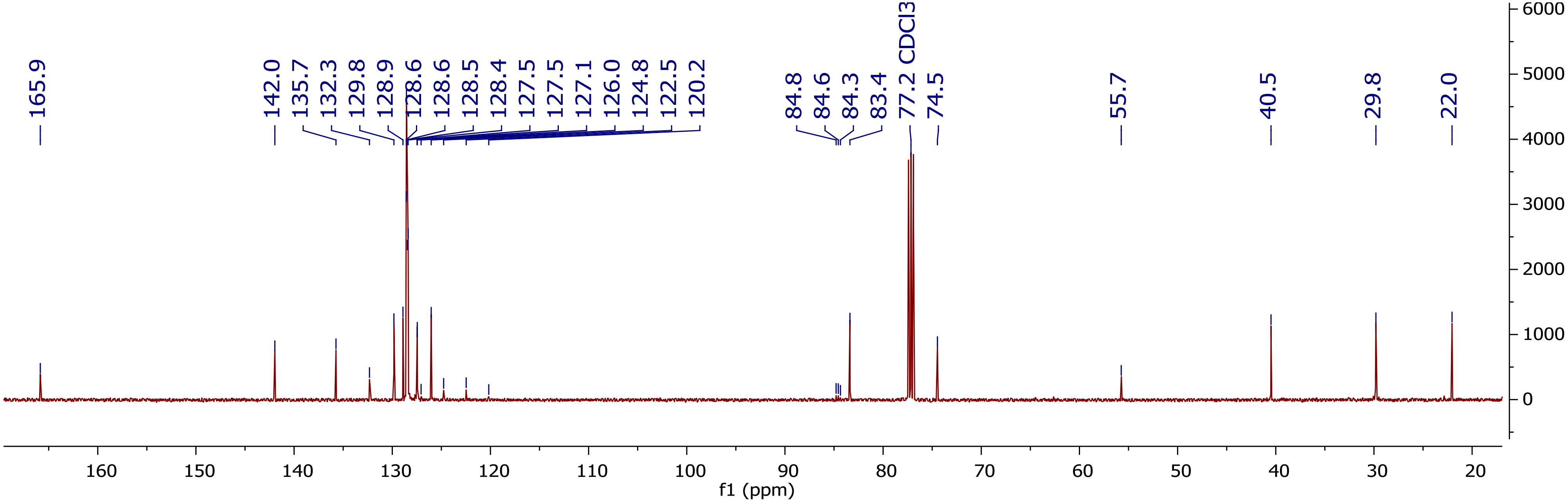

2,4,6-Triisopropylbenzothioic *S*-acid SI13

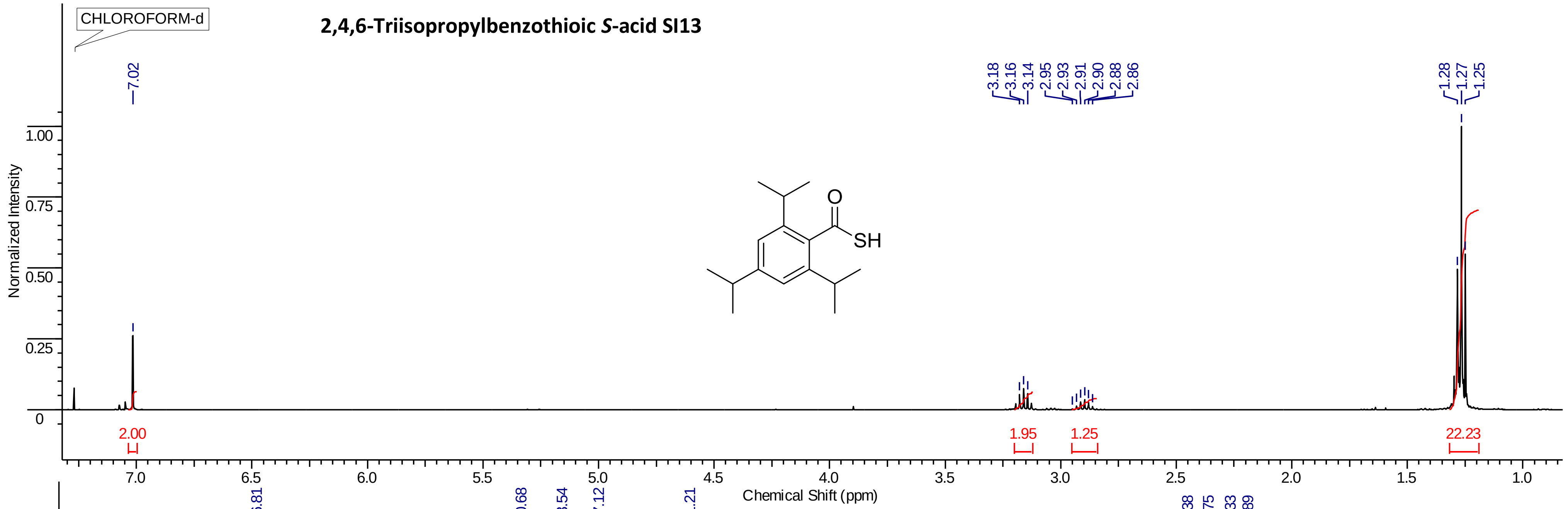

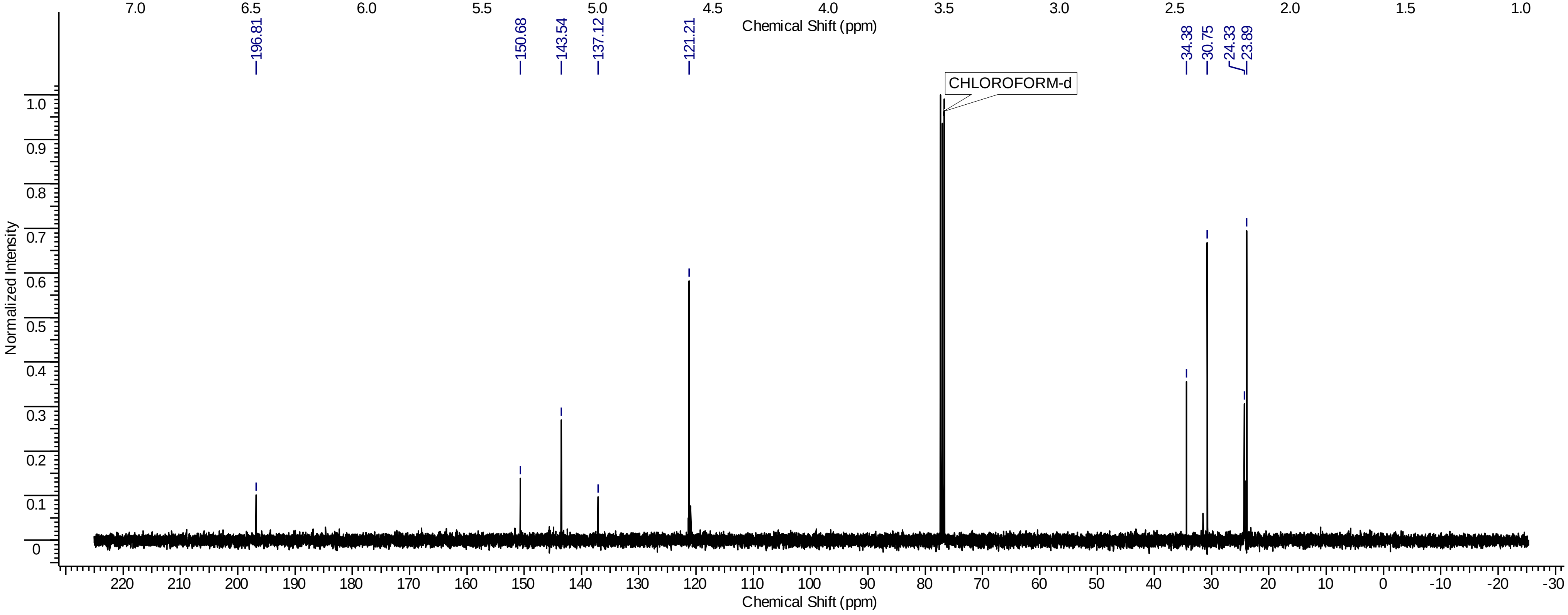

(*R*)-*S*-(4-Phenylbutan-2-yl) 2,4,6-triisopropylbenzothioate 4a

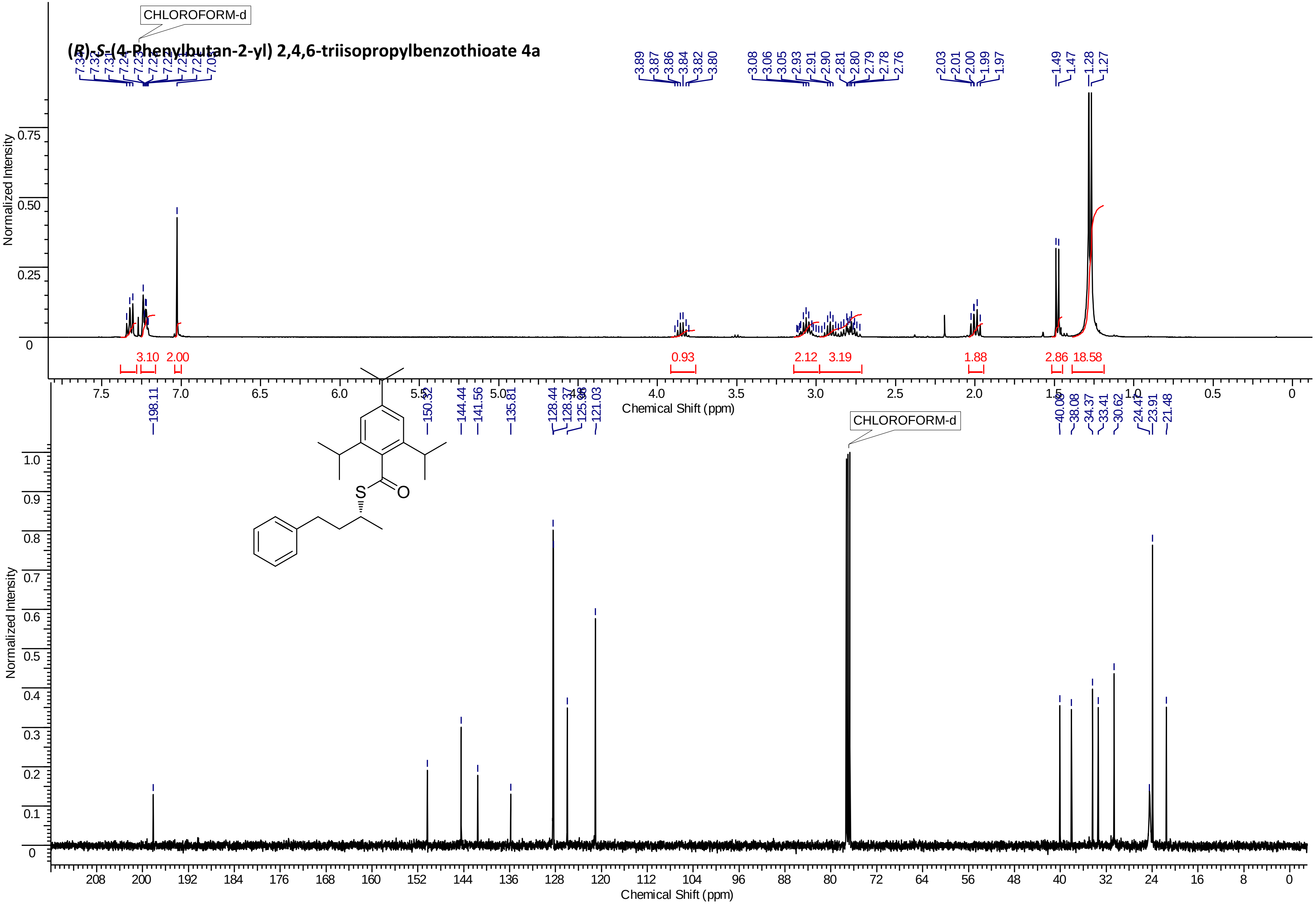

(*R*)-*S*-(1-Phenylpentan-3-yl) 2,4,6-triisopropylbenzothioate 4b

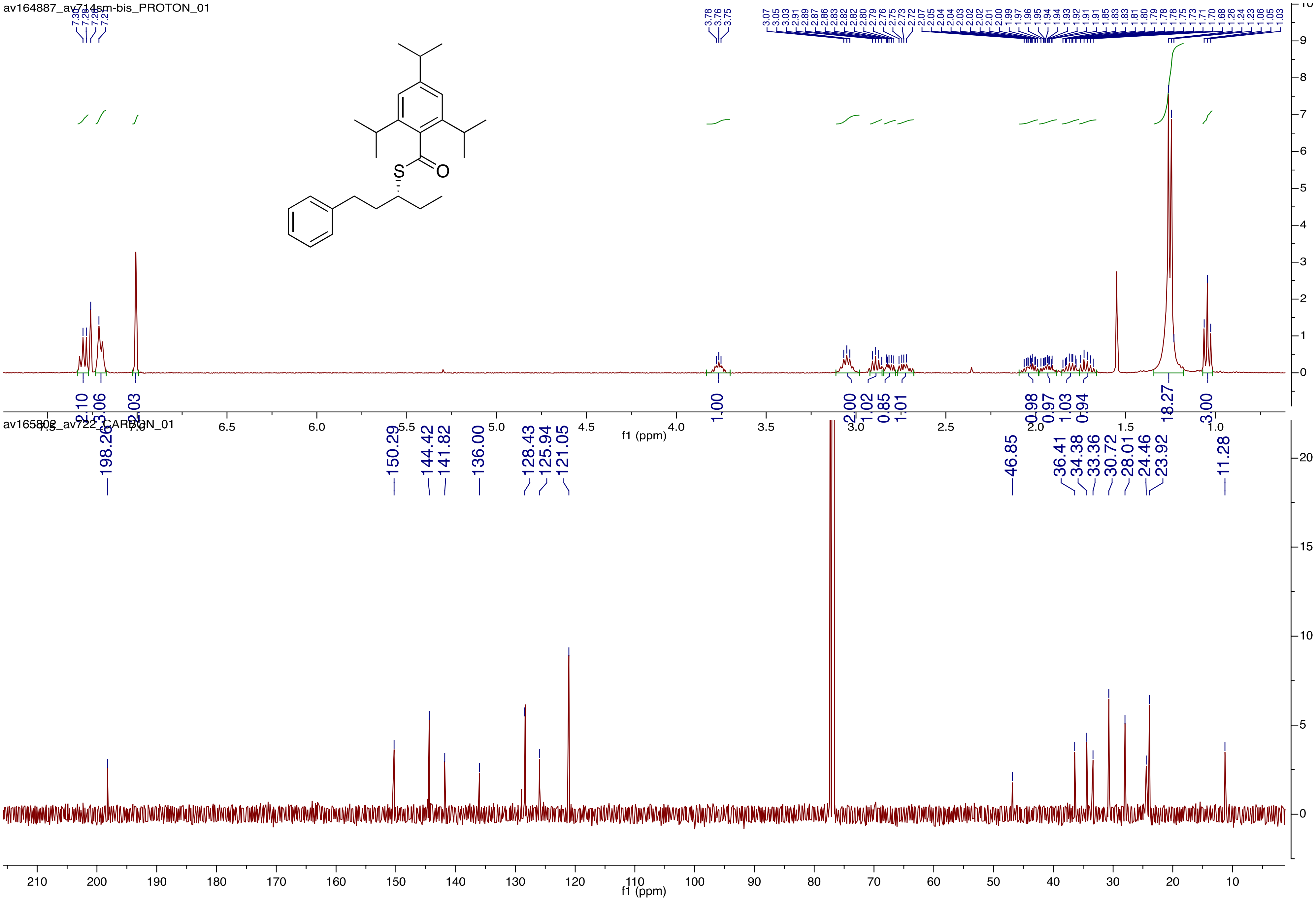

(*R*)-S-Pentan-2-yl 2,4,6-triisopropylbenzothioate 4c

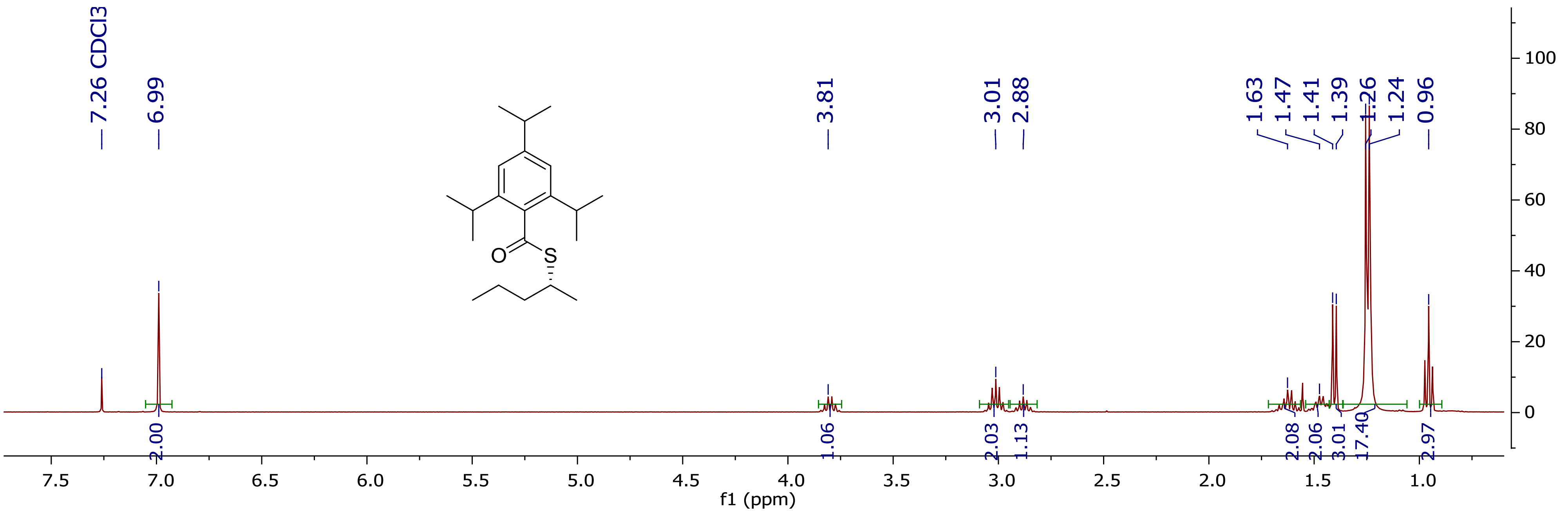

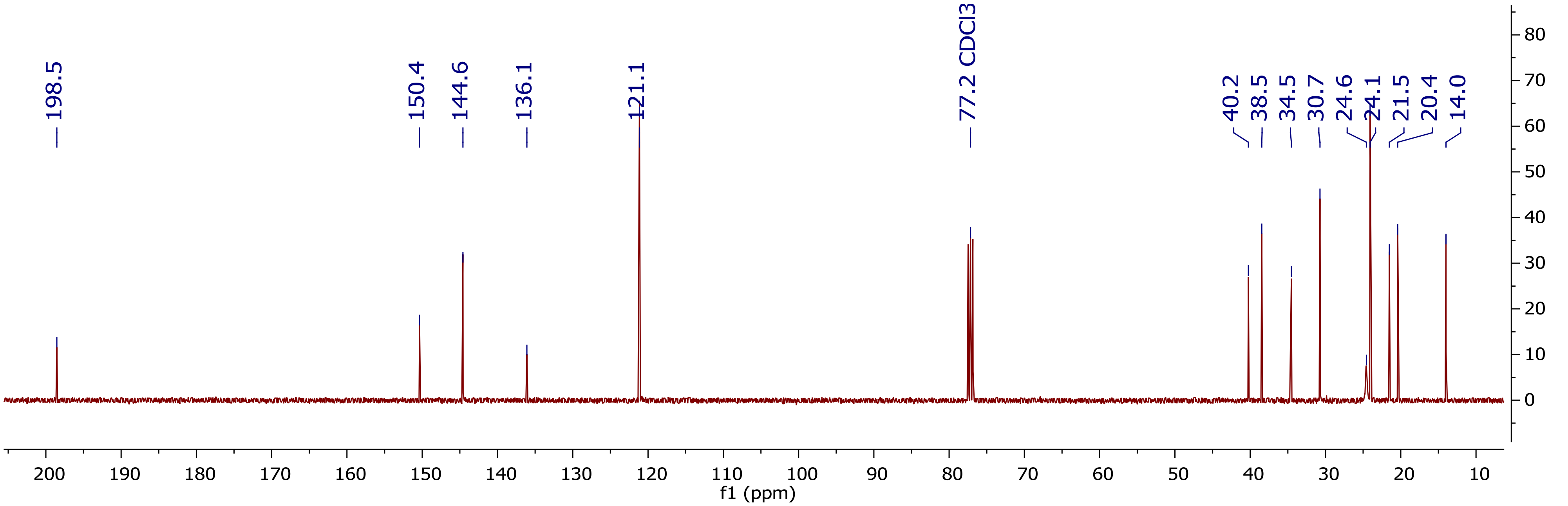

(*R*)-*S*-Hex-5-en-2-yl 2,4,6-triisopropylbenzothioate 4d

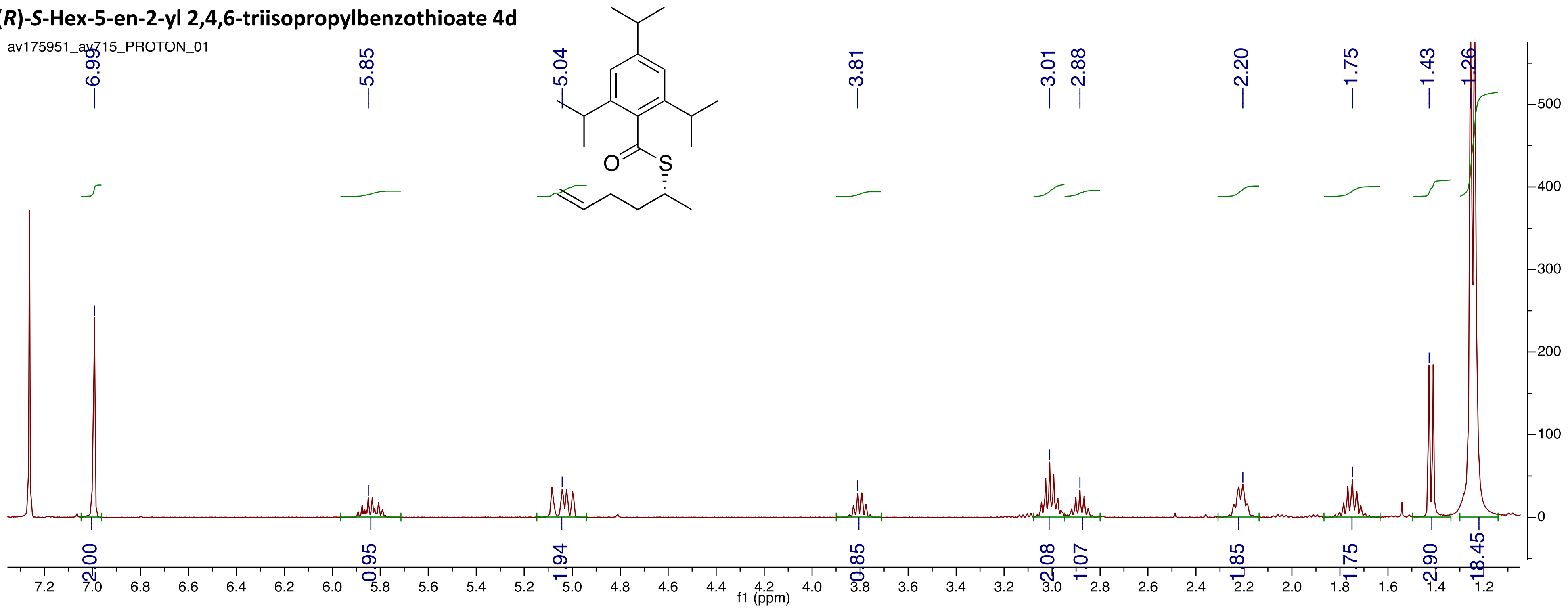

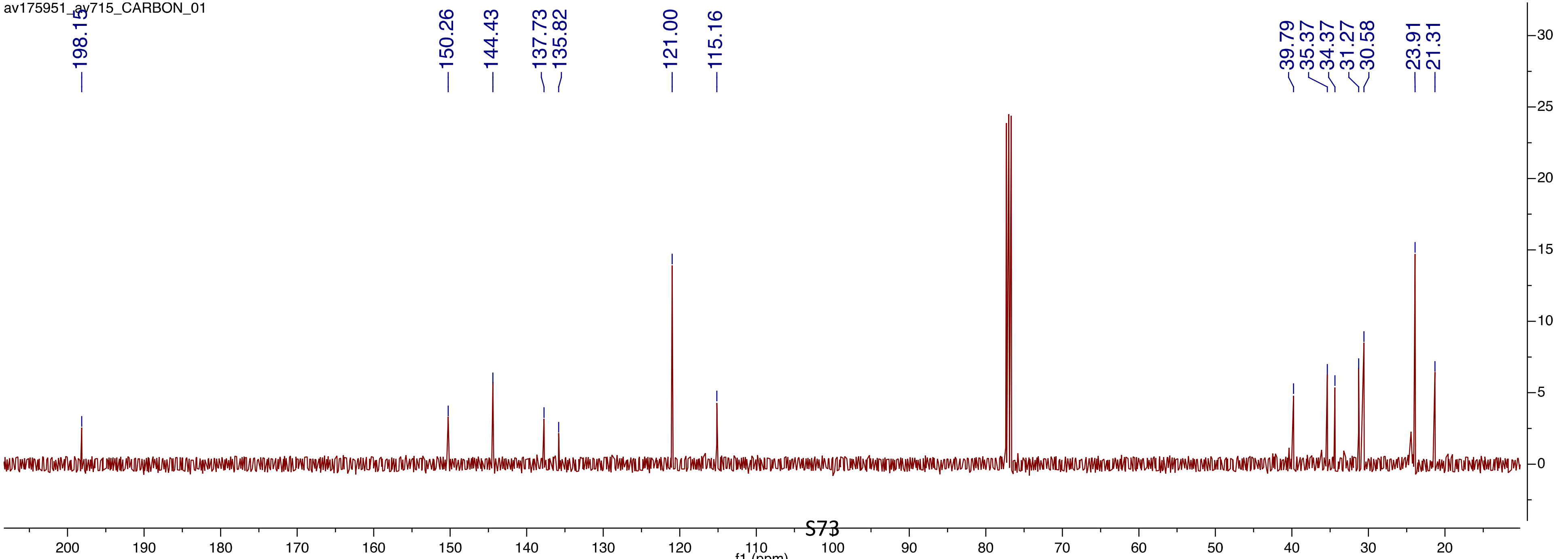

(*R*)-*S*-(5-Hydroxypentan-2-yl) 2,4,6-triisopropylbenzothioate SI16

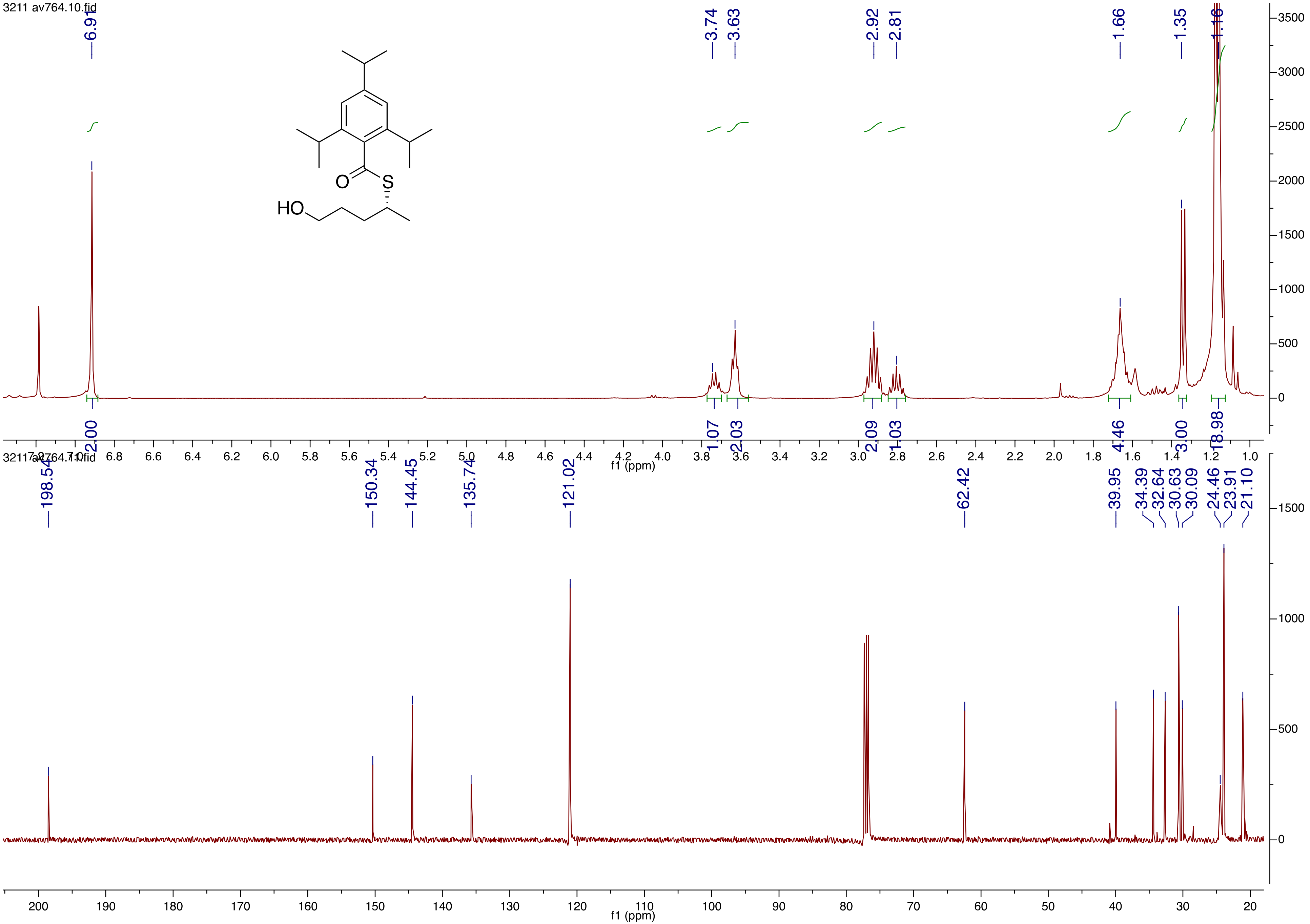

S-((3R)-5-((Tetrahydro-2H-pyran-2-yl)oxy)pentan-2-yl) 2,4,6-triisopropylbenzothioate 4e

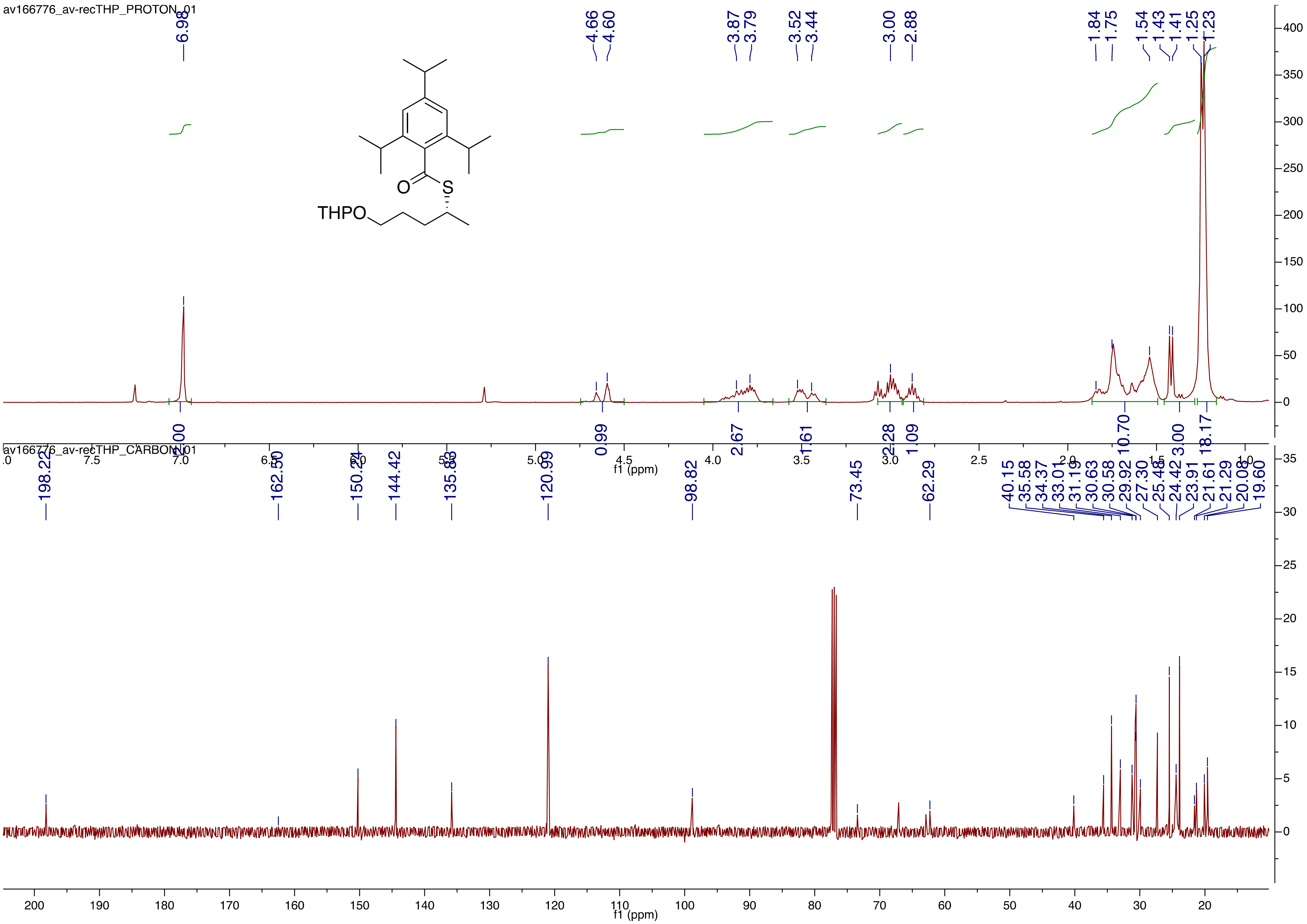

S-((3R,8S,9S,10R,13R,14S,17R)-10,13-Dimethyl-17-((R)-6-methylheptan-2-yl)-2,3,4,7,8,9,10,11,12,13,14,15,16,17-tetradecahydro-1H-cyclopenta[a]phenanthren-3-yl) 2,4,6-triisopropylbenzothioate 8

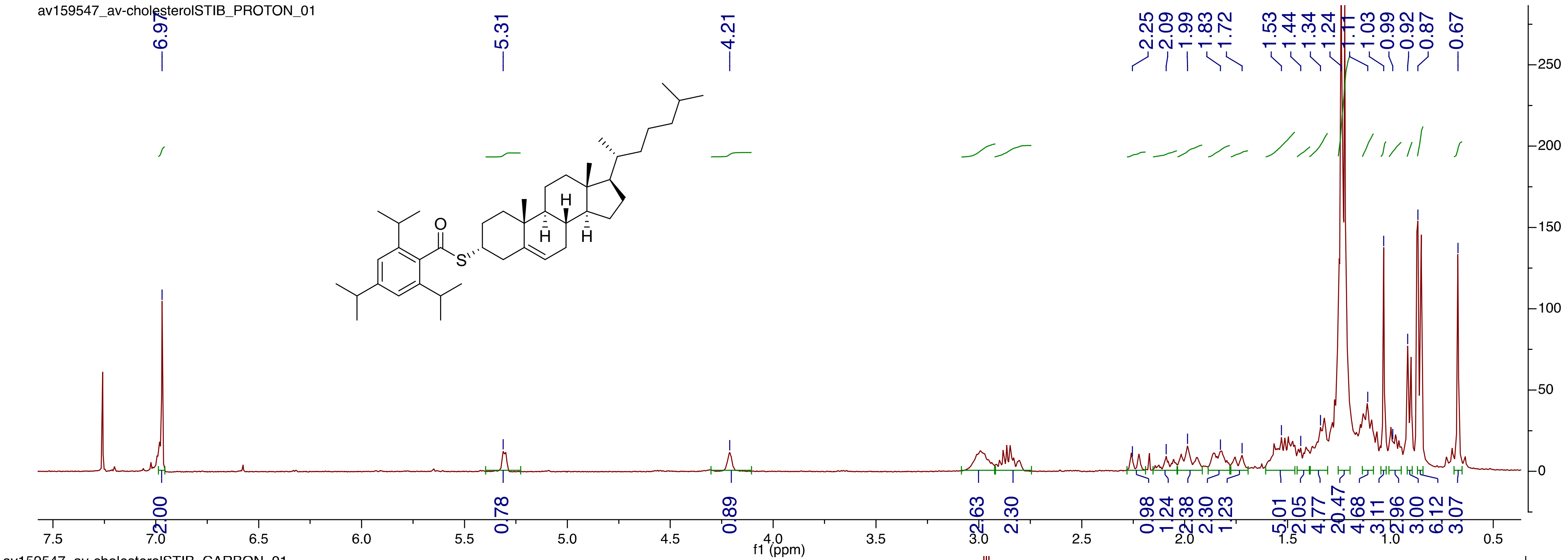

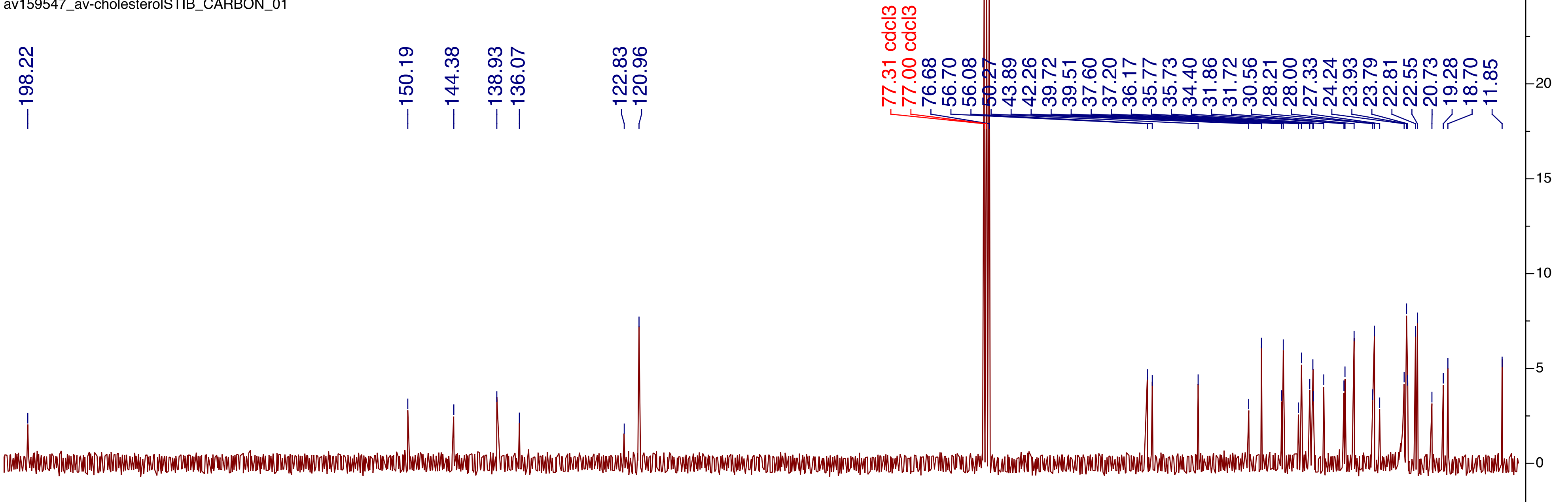

Methyl (*R*)-2-methyl-4-phenyl-2-((2,4,6-triisopropylbenzoyl)thio)butanoate 6aa

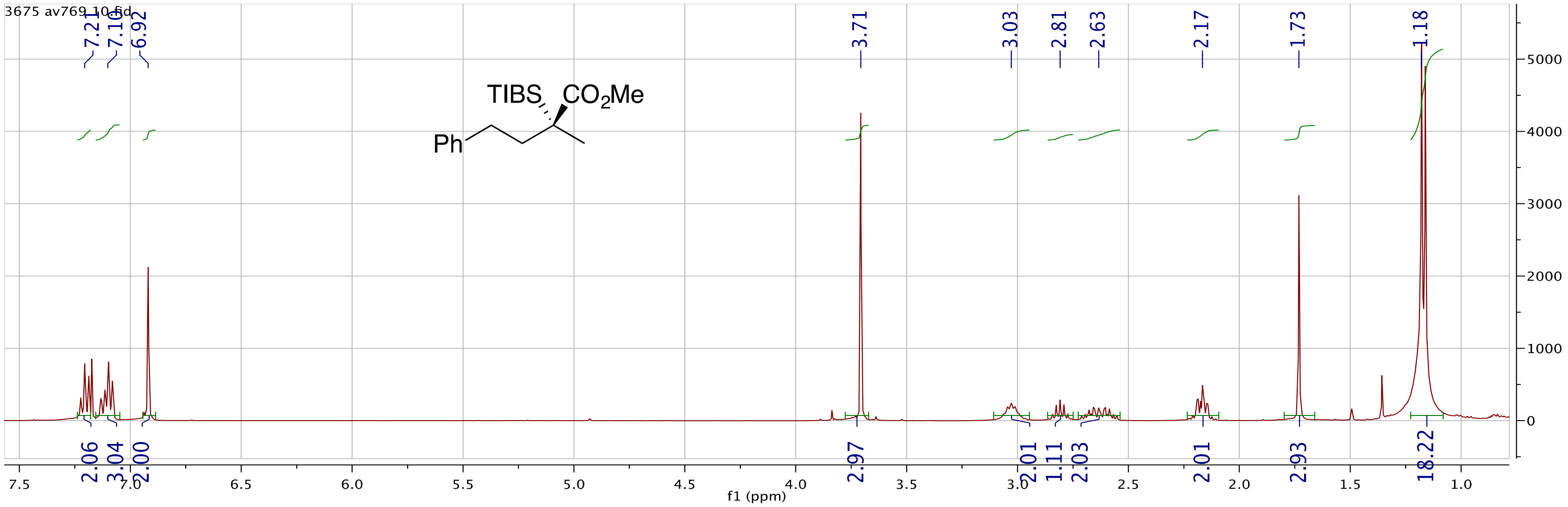

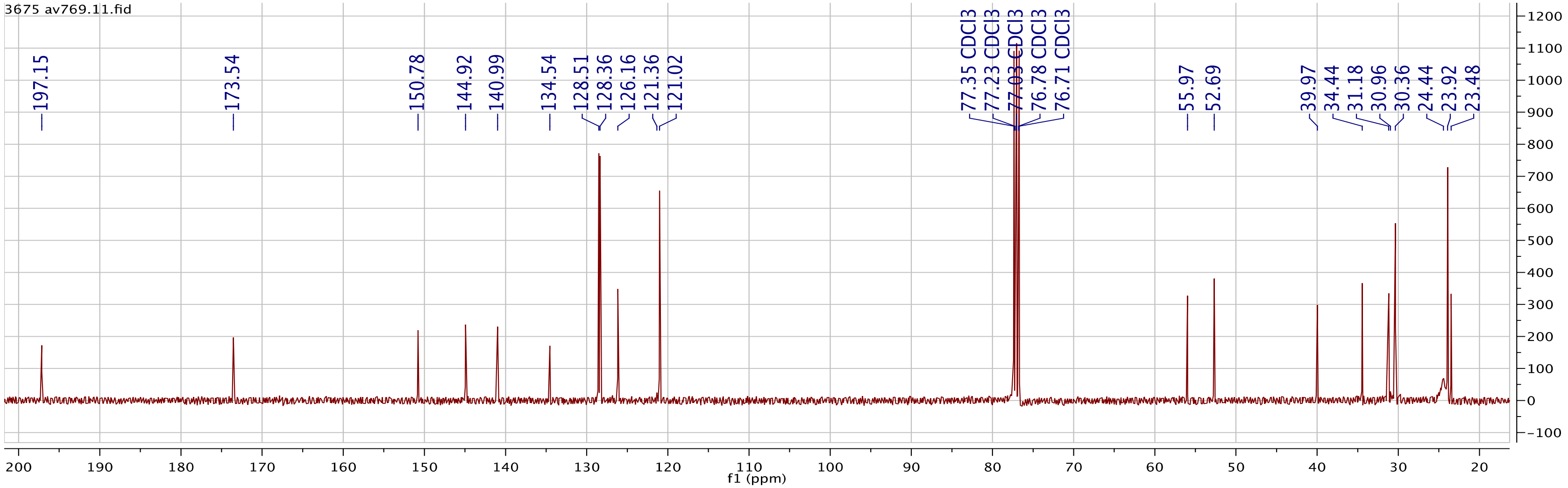

(*R*)-*S*-(1-((4-Bromophenyl)amino)-2-methyl-1-oxo-4-phenylbutan-2-yl) 2,4,6-triisopropylbenzothioate 6ab

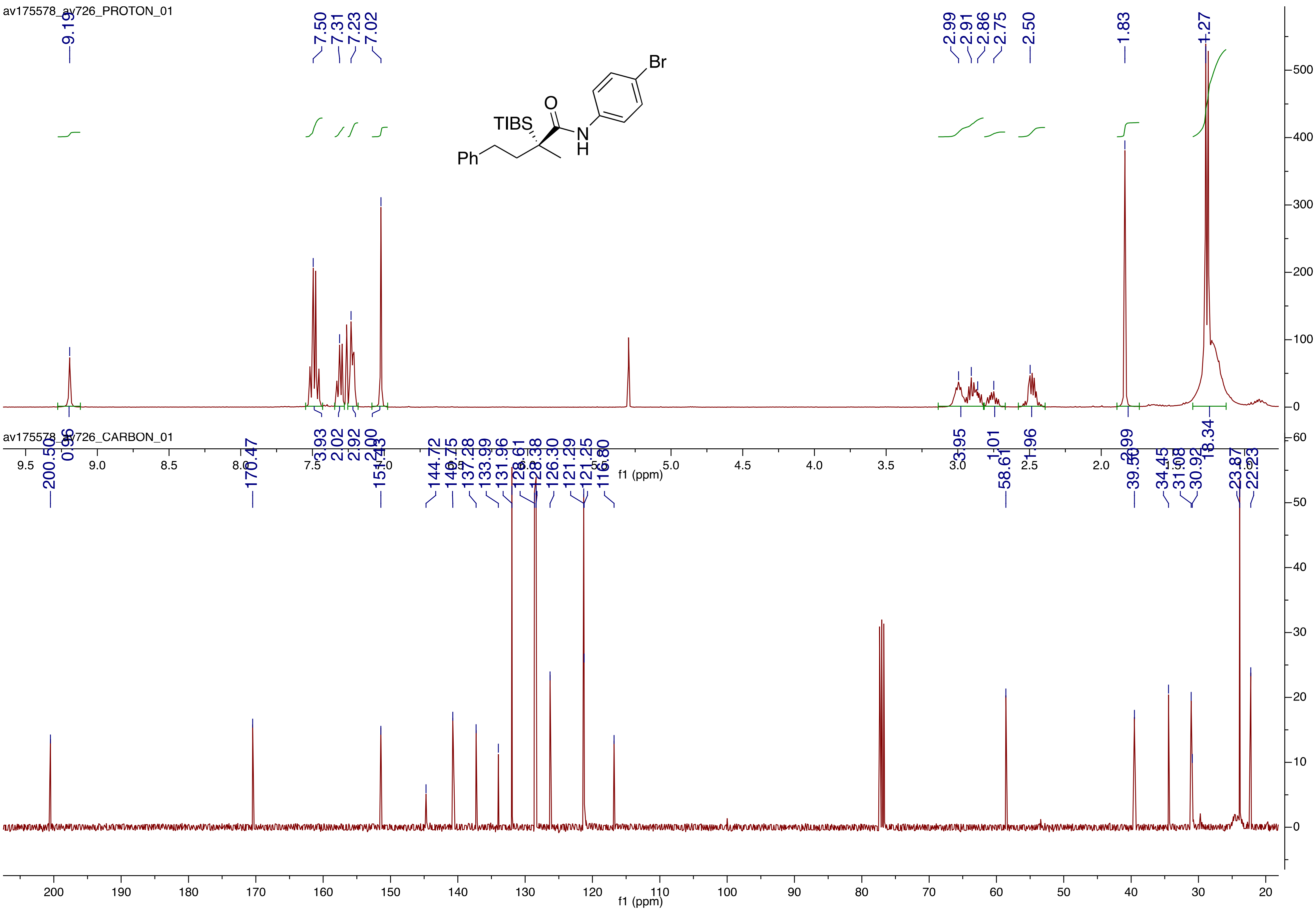

(*R*)-*S*-(2-Methyl-1-oxo-4-phenylbutan-2-yl) 2,4,6-triisopropylbenzothioate 6ac

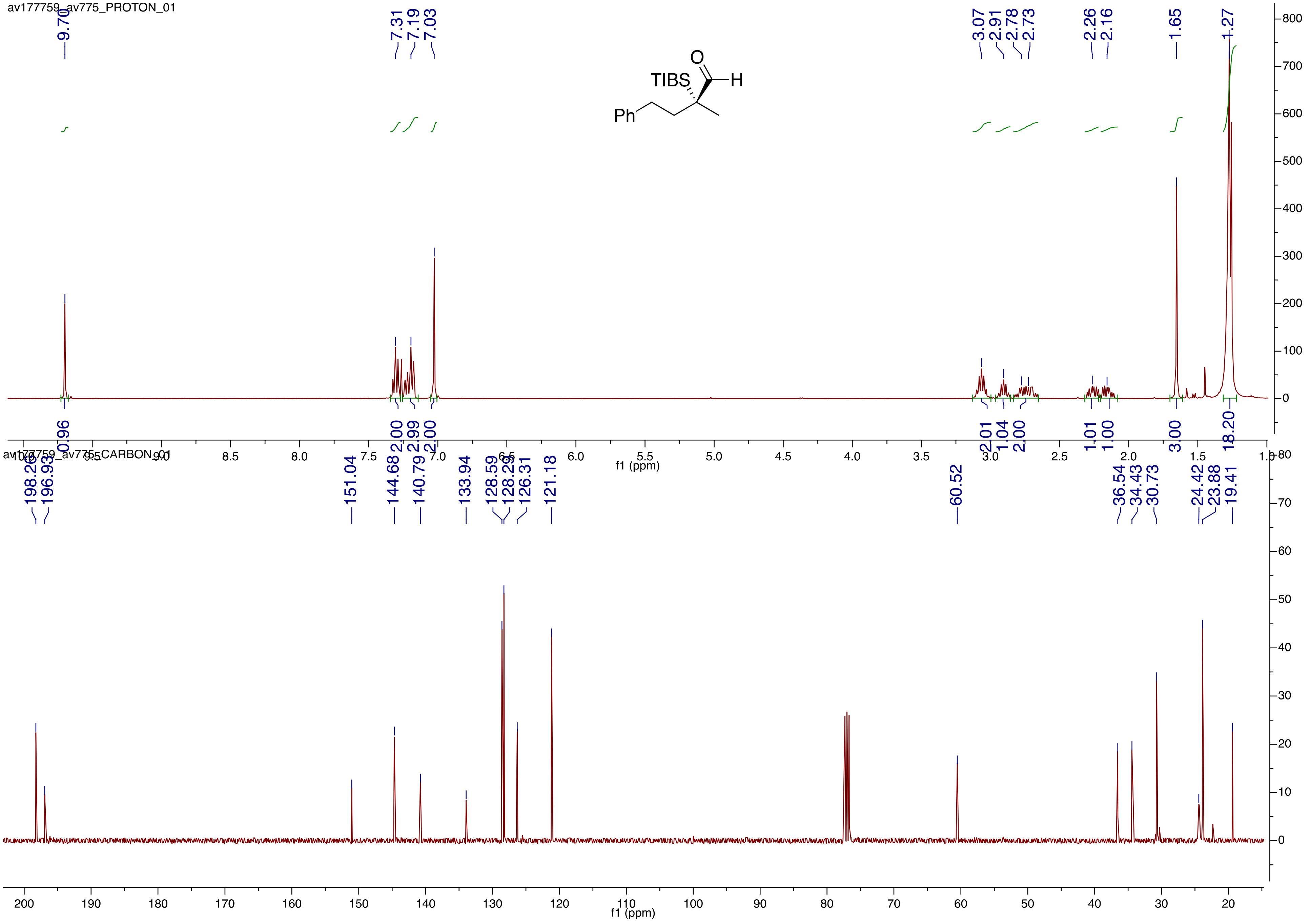

S-((1R,2R)-1-Hydroxy-2-methyl-1,4-diphenylbutan-2-yl) 2,4,6-triisopropylbenzothioate 6ad – Diastereomer A

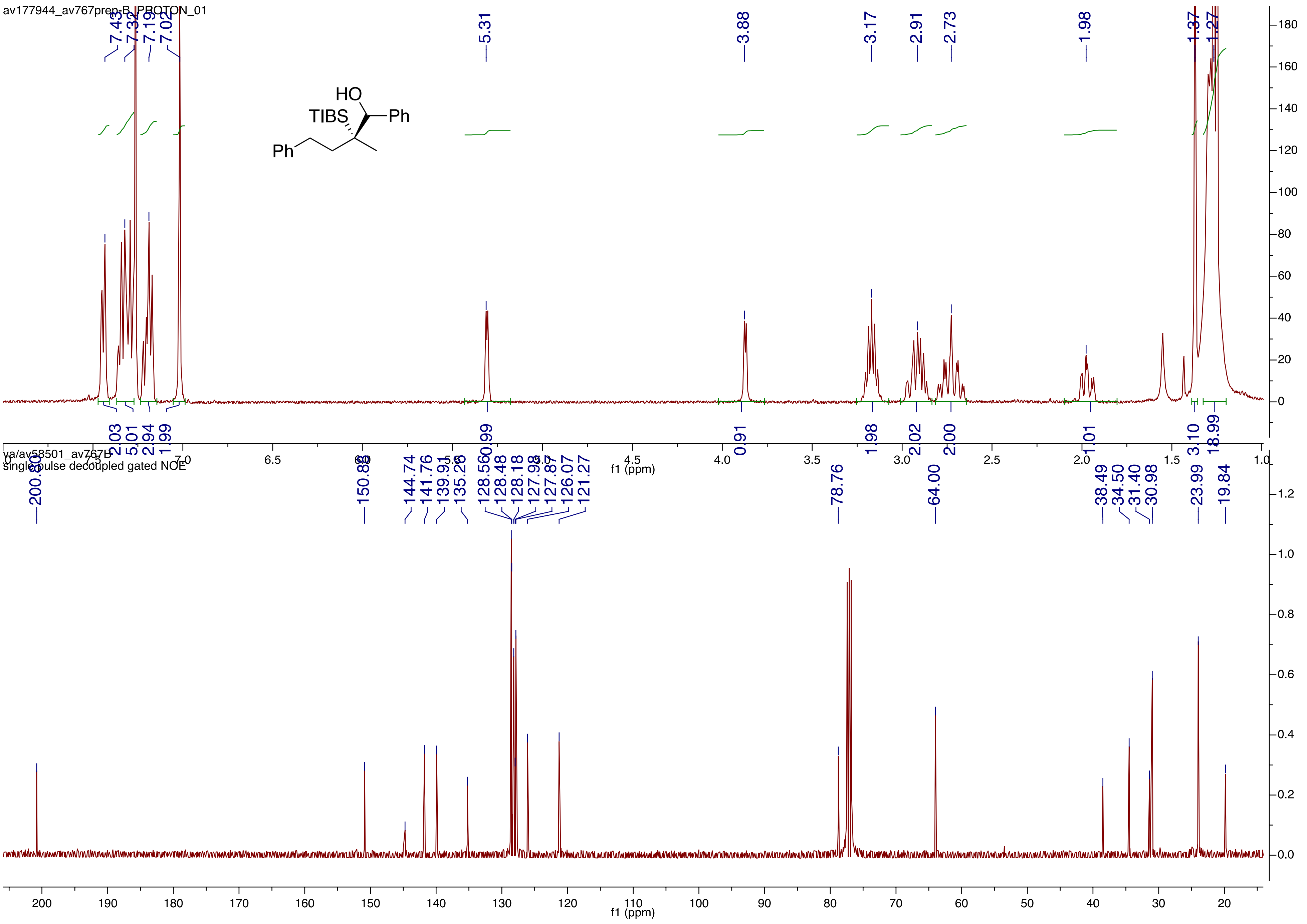

S-((1R,2R)-1-Hydroxy-2-methyl-1,4-diphenylbutan-2-yl) 2,4,6-triisopropylbenzothioate 6ad – Diastereomer B

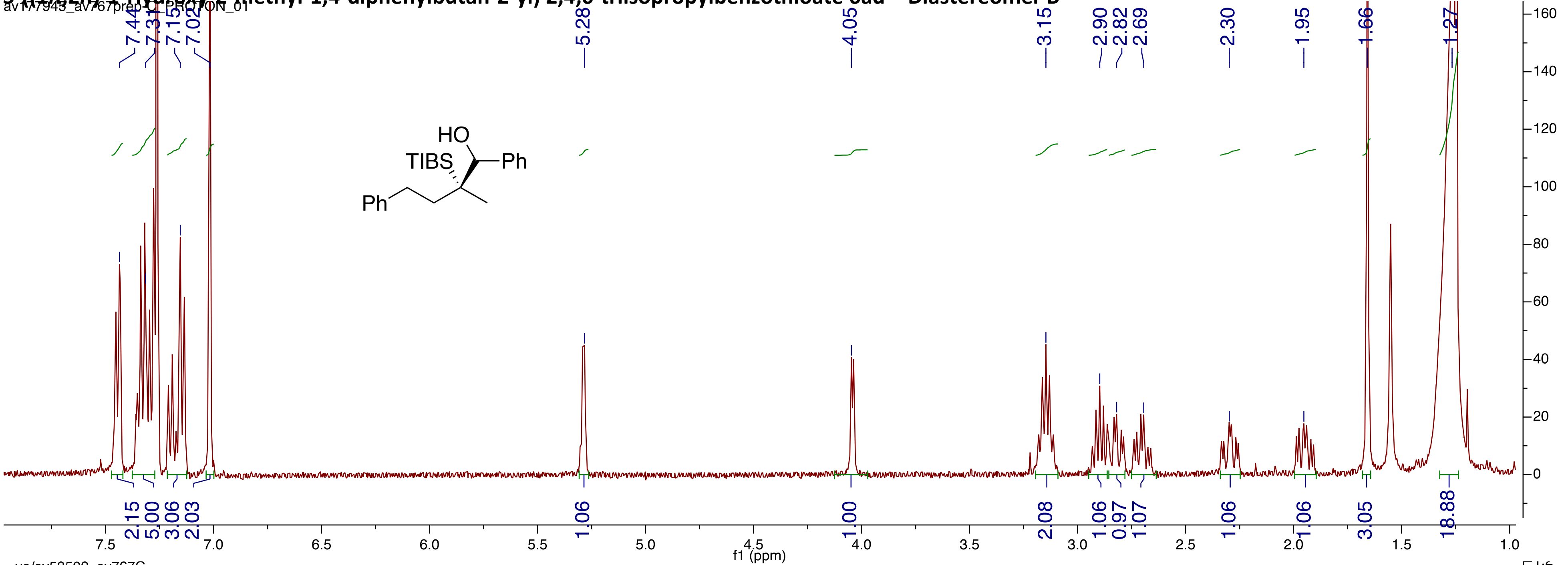

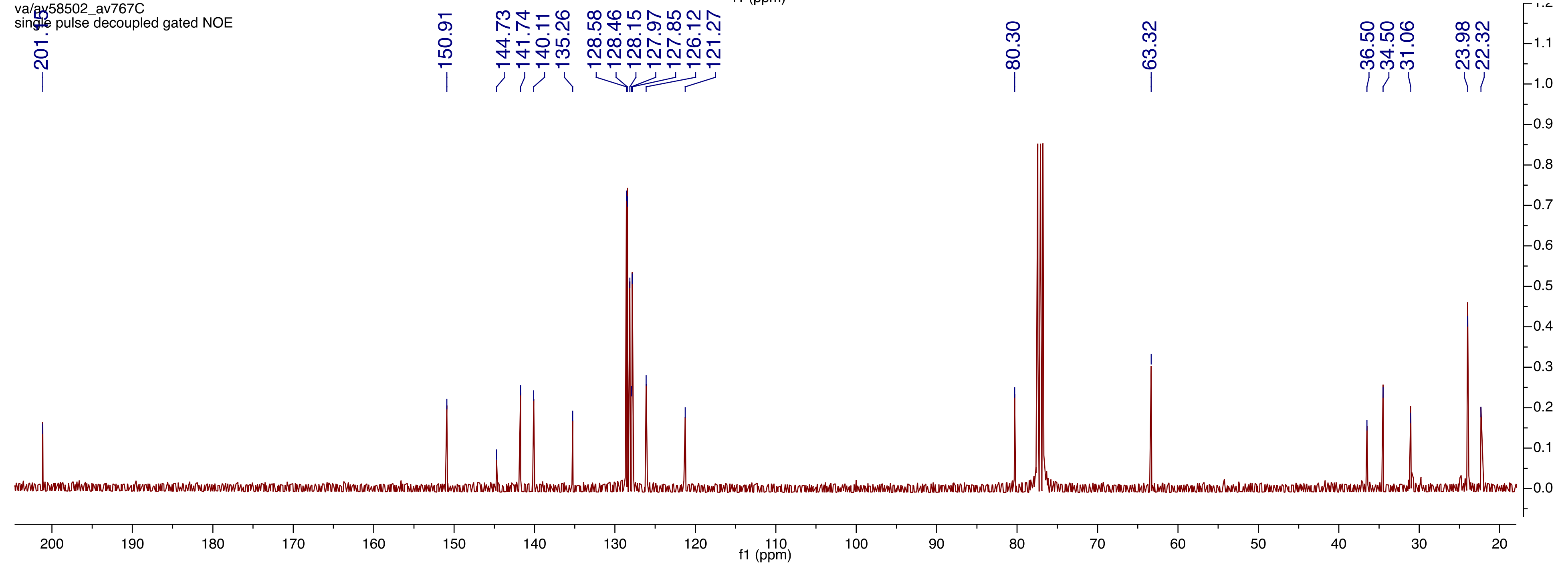

(*R*)-*S*-(4-Phenyl-2-(trimethylsilyl)butan-2-yl) 2,4,6-triisopropylbenzothioate 6ae

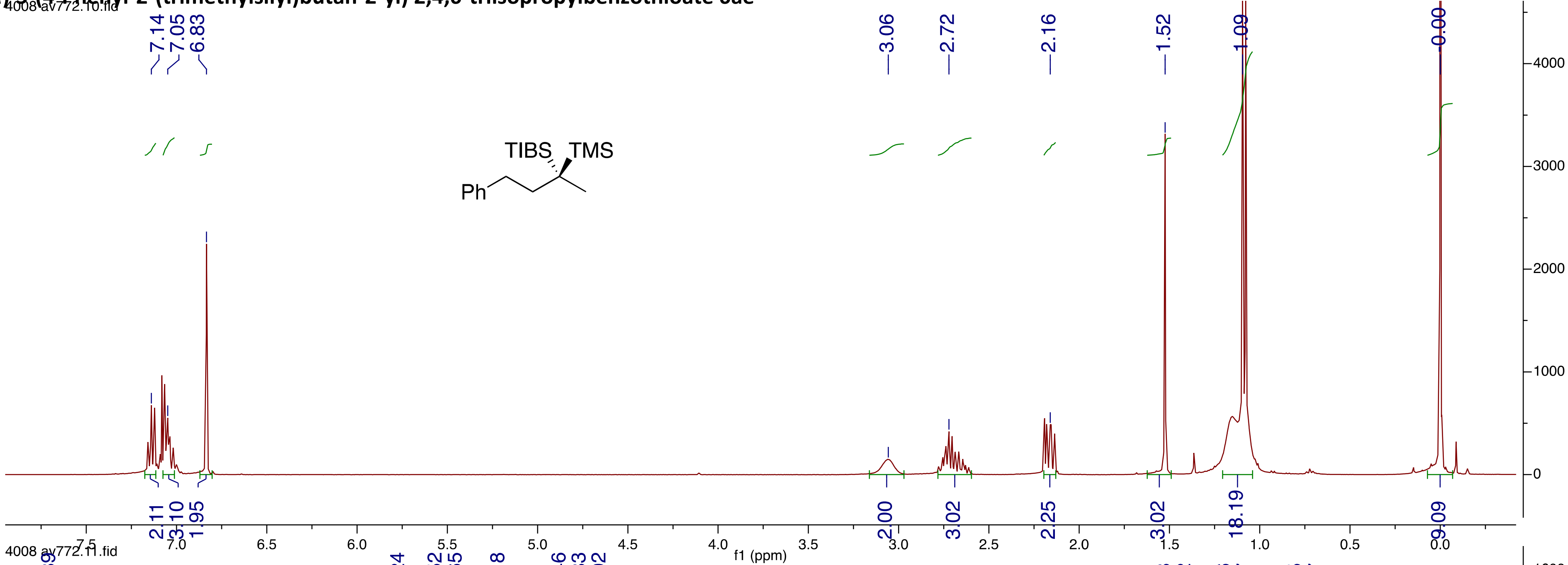

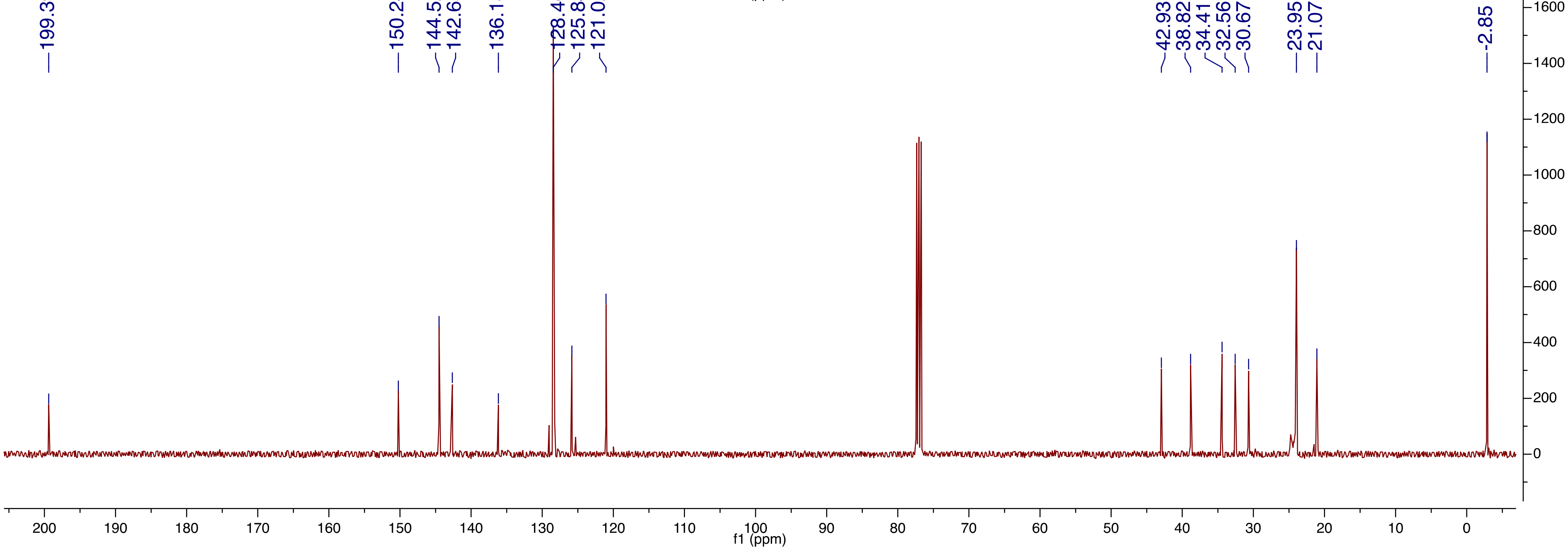

(*R*)-*S*-(4-Phenyl-2-(tributylstannyl)butan-2-yl) 2,4,6-triisopropylbenzothioate 6af

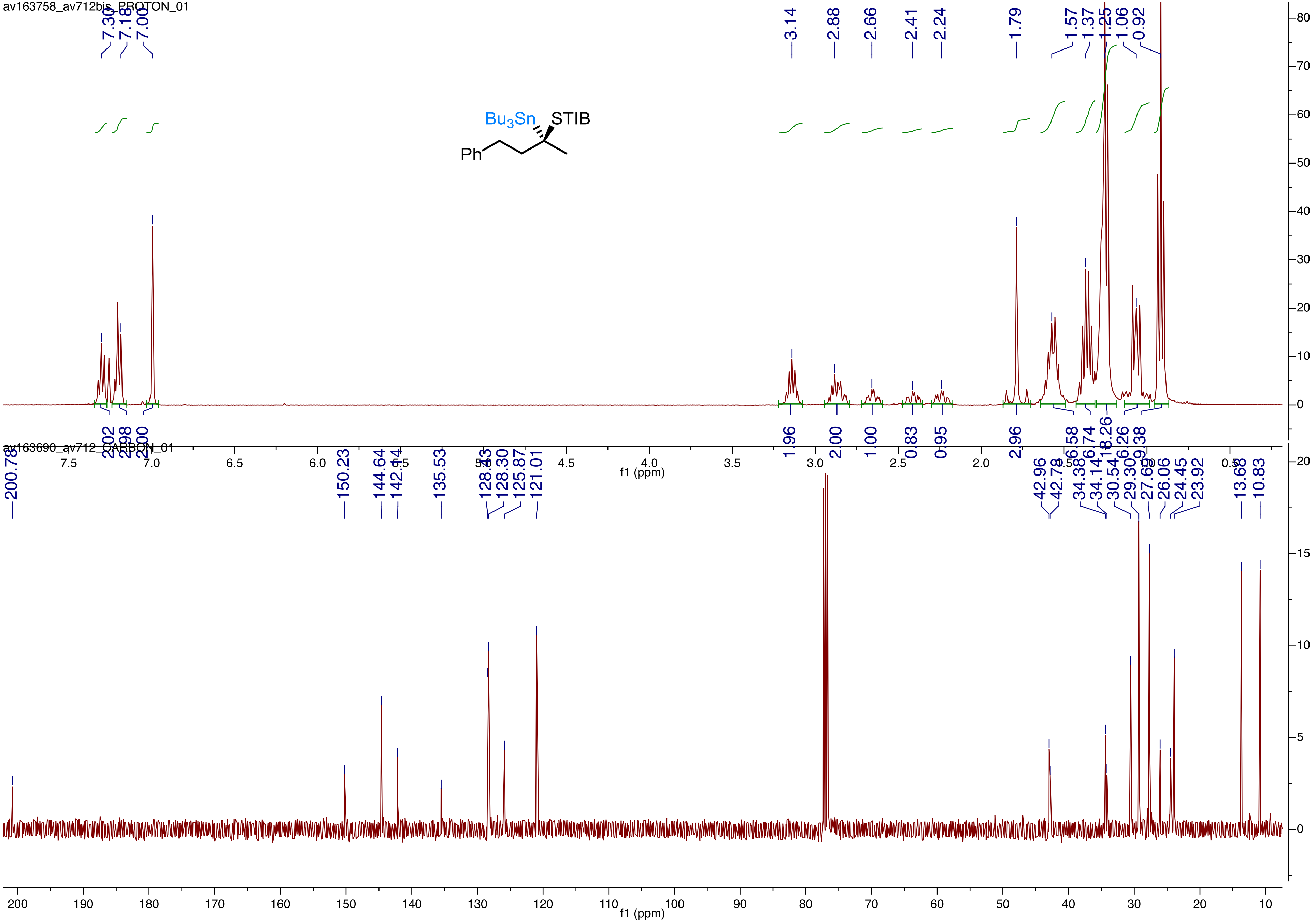

(S)-S-(4-phenyl-2-(trimethylstannyl)butan-2-yl) 2,4,6-triisopropylbenzothioate 6ag

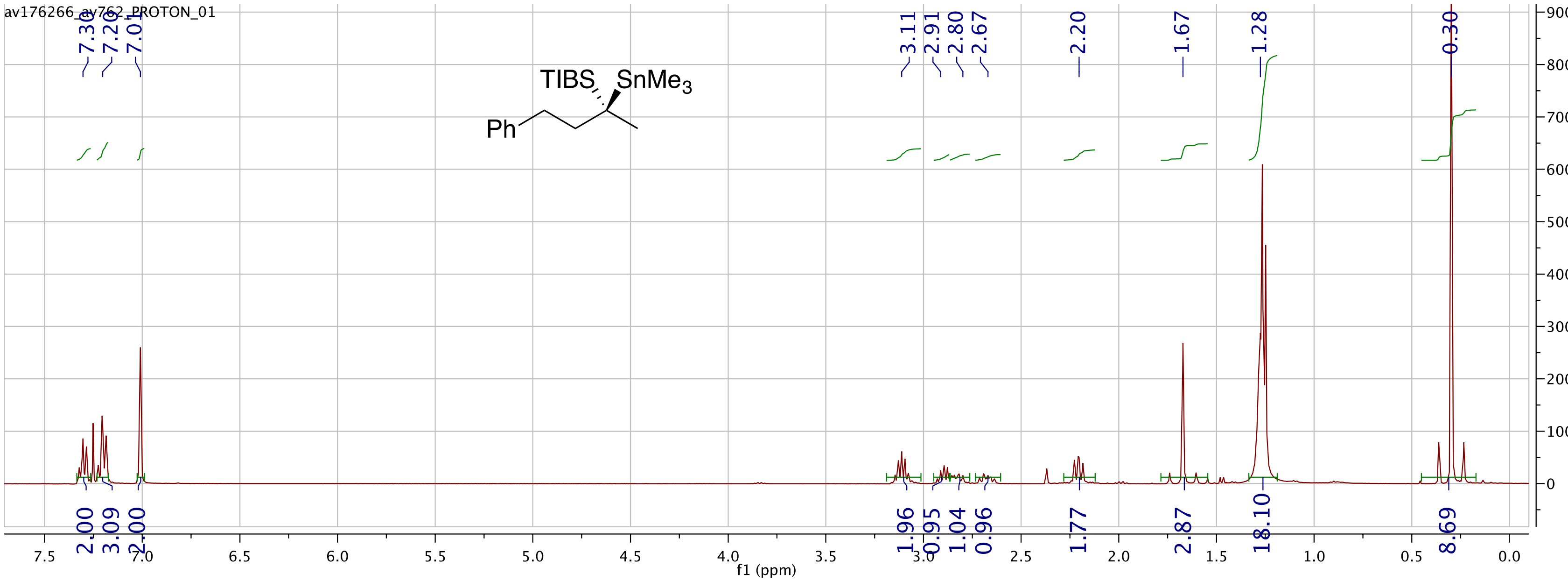

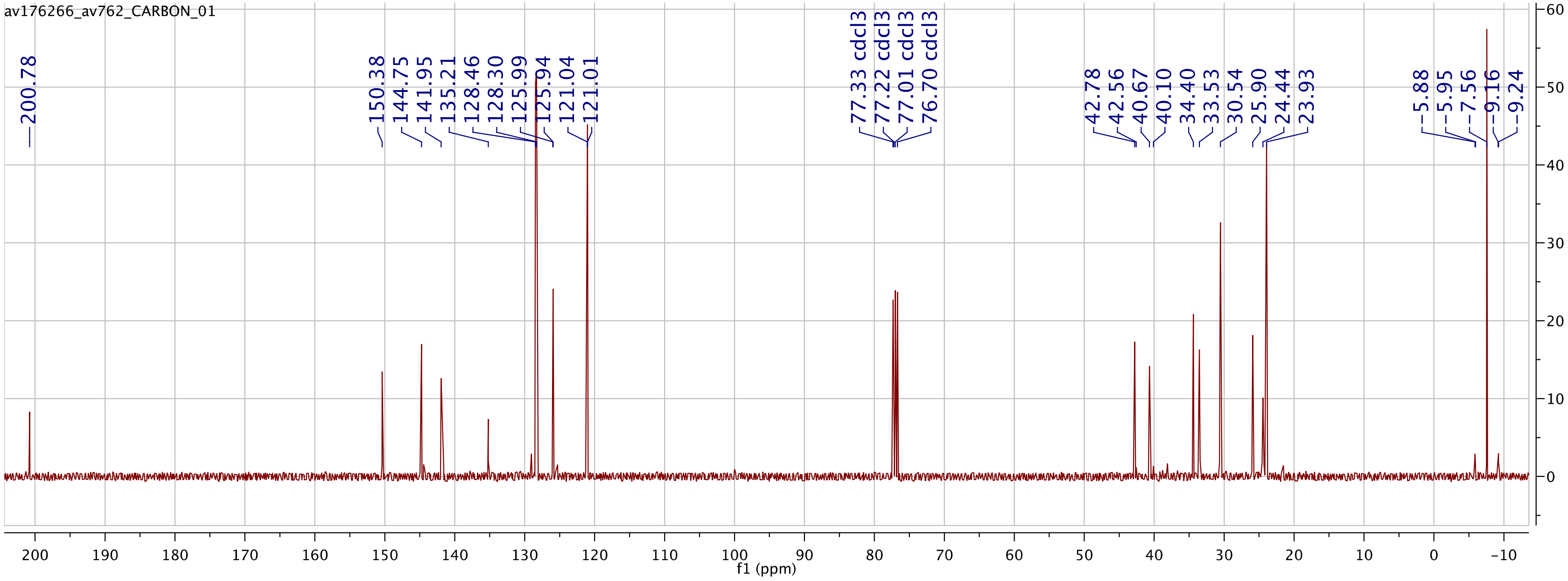

(*R*)-2-Methyl-4-phenyl-2-((2,4,6-triisopropylbenzoyl)thio)butanoic acid 6ah

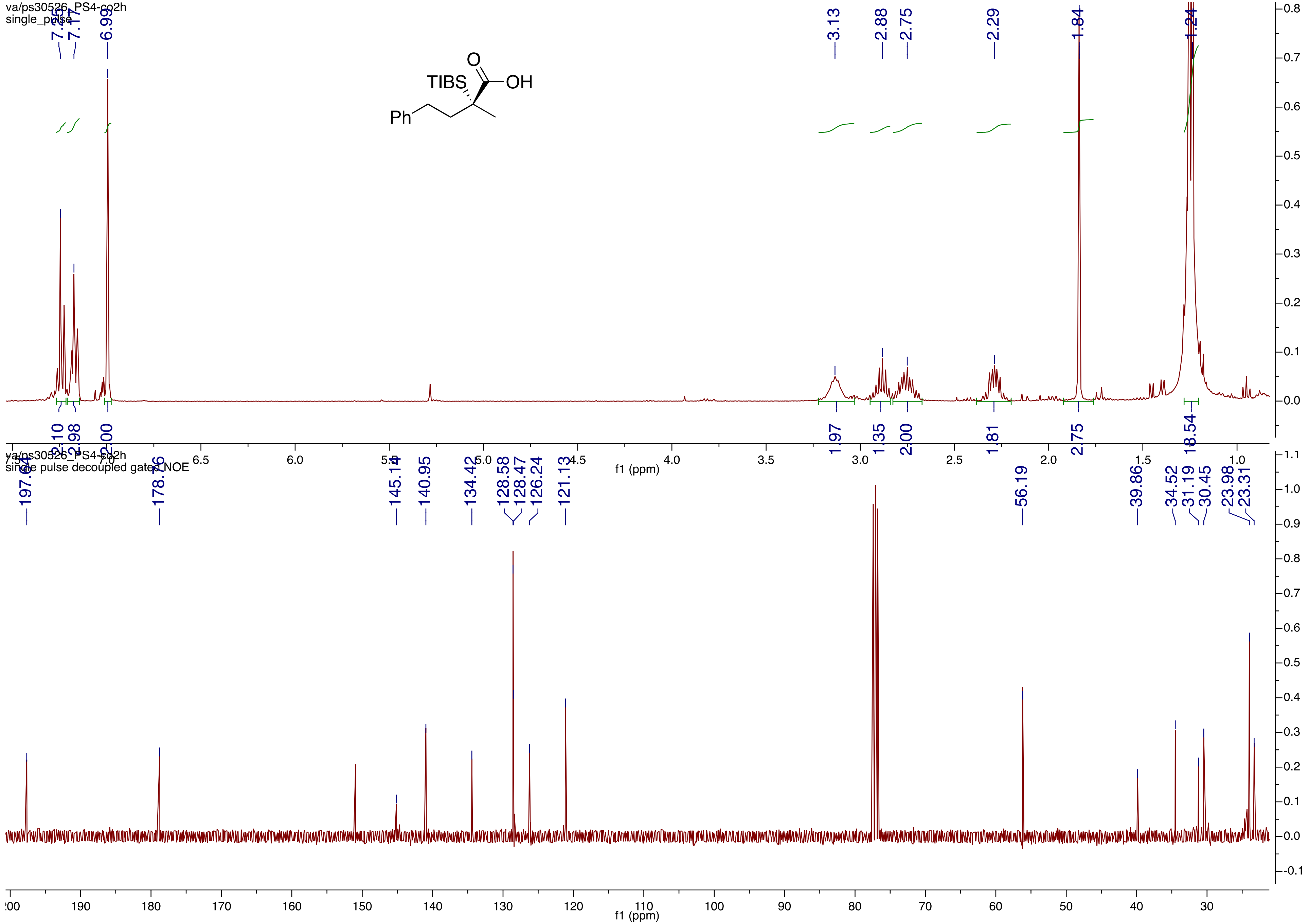

(*R*)-*S*-(3-((4-Bromophenyl)carbamoyl)-1-phenylpentan-3-yl) 2,4,6-triisopropylbenzothioate 6ba

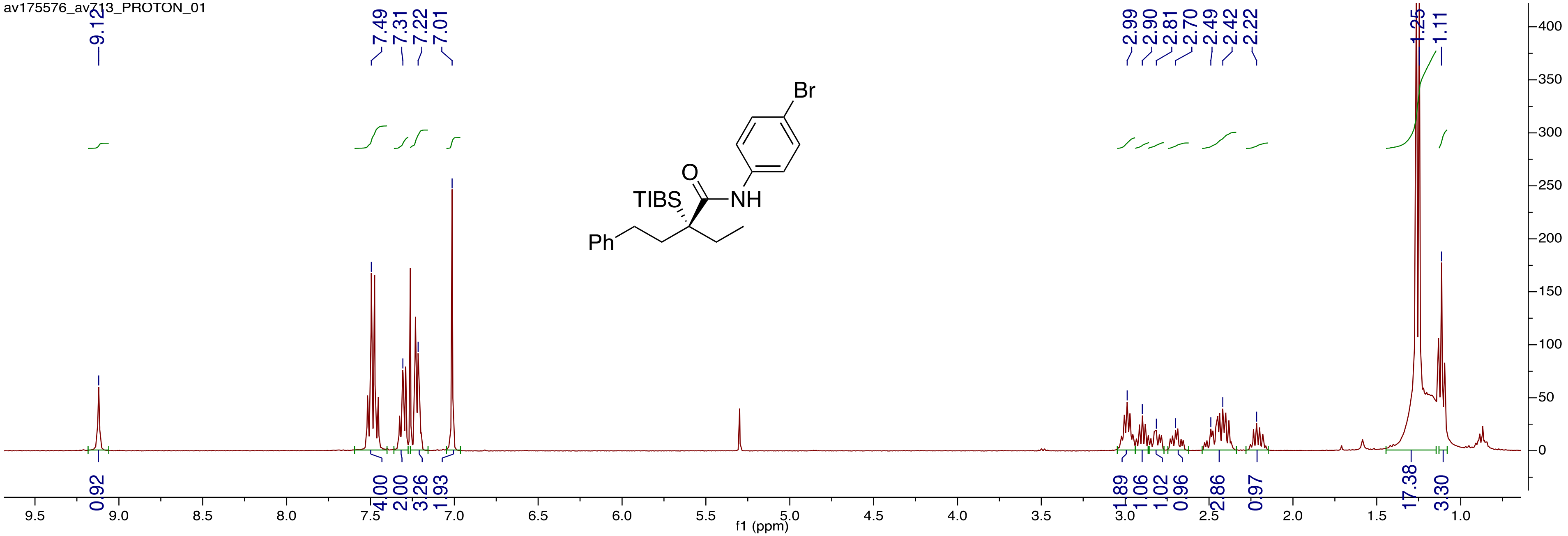

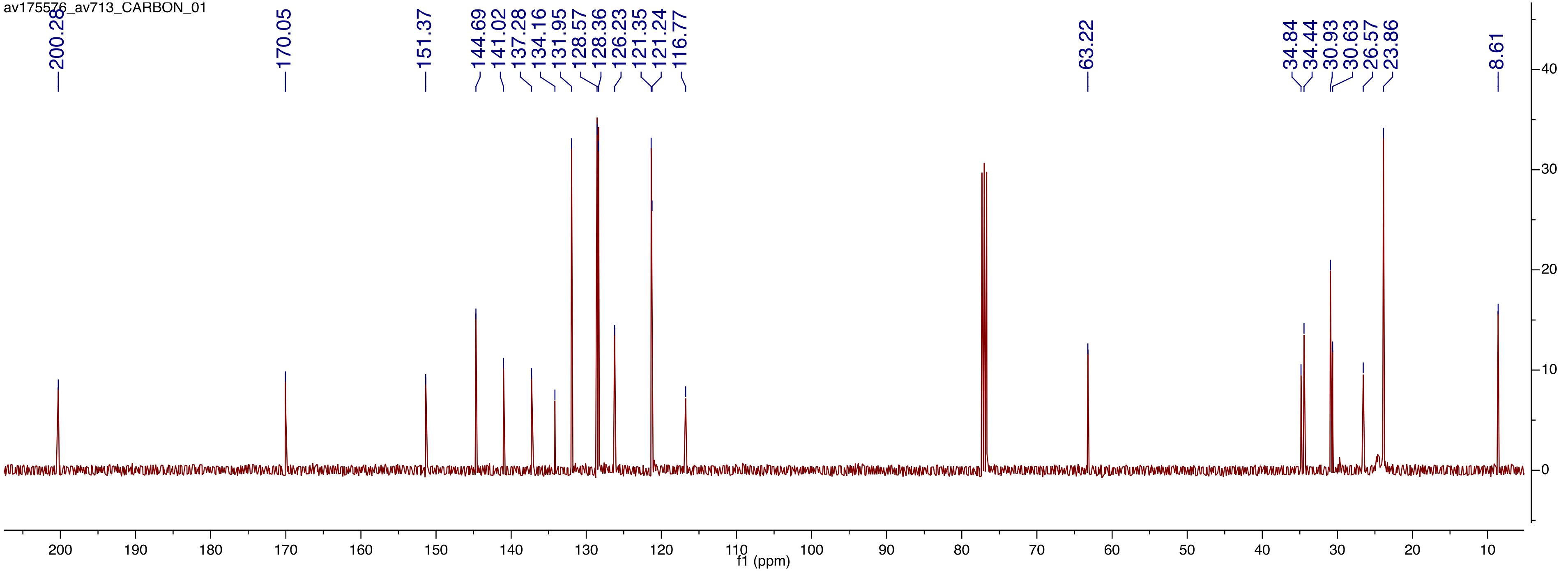

(*R*)-Methyl 2-methyl-2-((2,4,6-triisopropylbenzoyl)thio)pentanoate 6ca

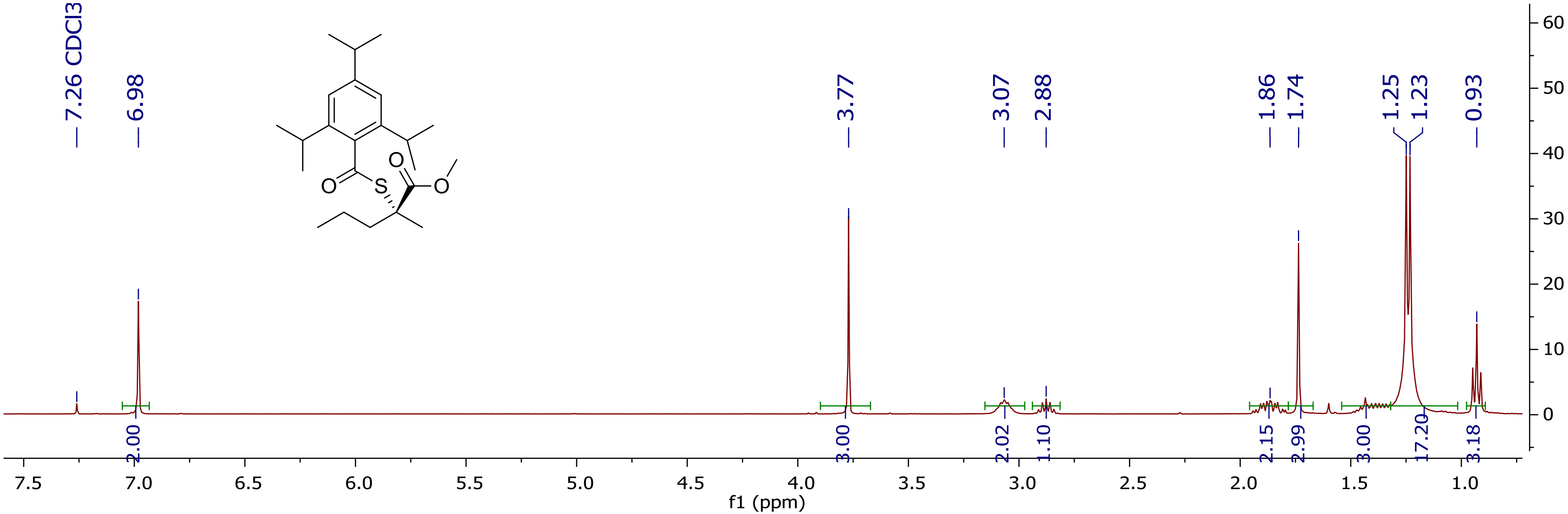

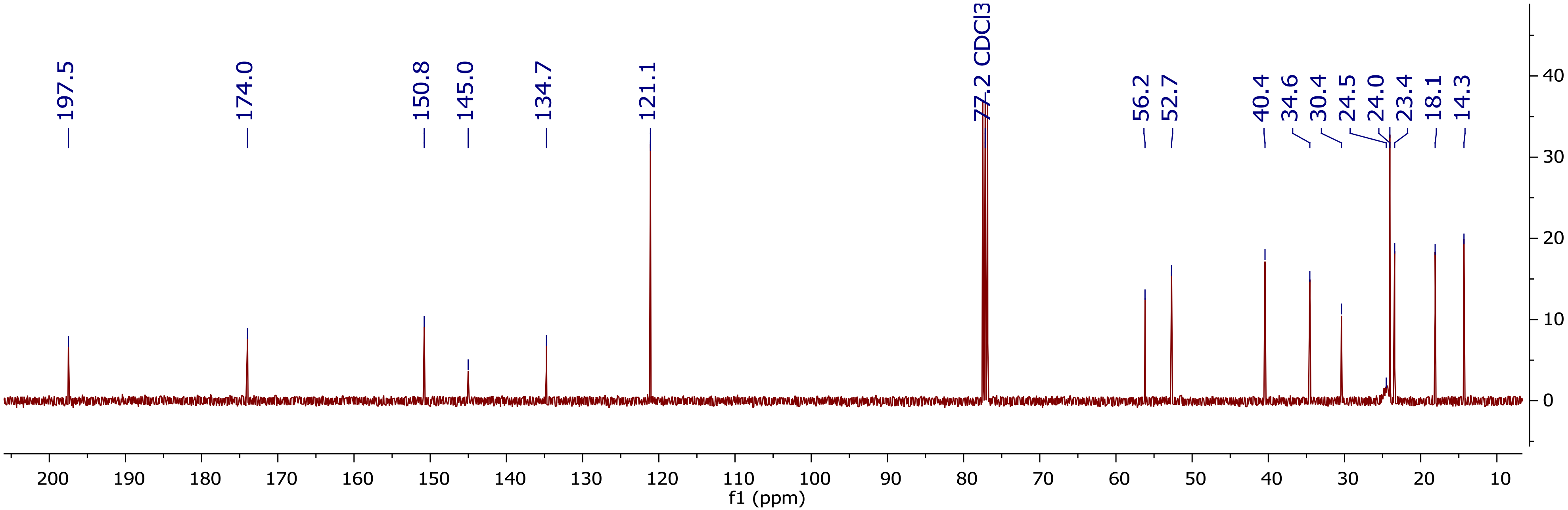

(*R*)-*S*-(1-((4-Bromophenyl)amino)-2-methyl-1-oxohex-5-en-2-yl) 2-ethyl-4,6-diisopropylbenzothioate 6da

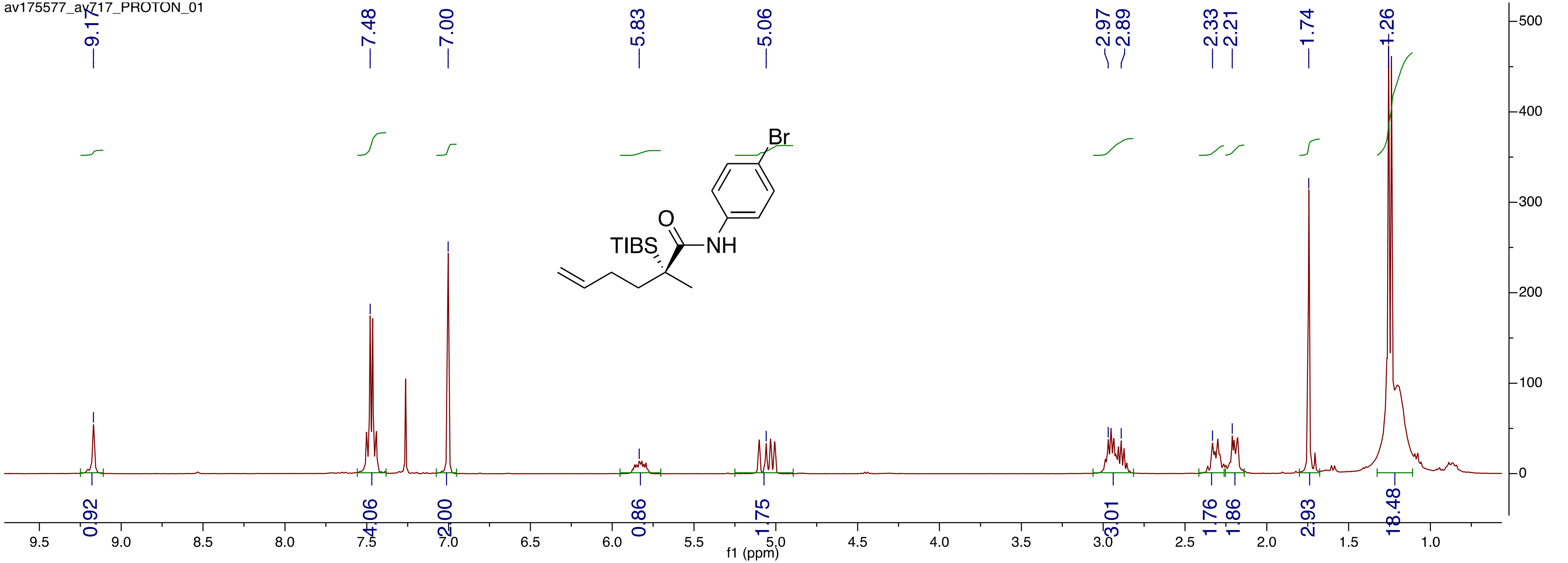

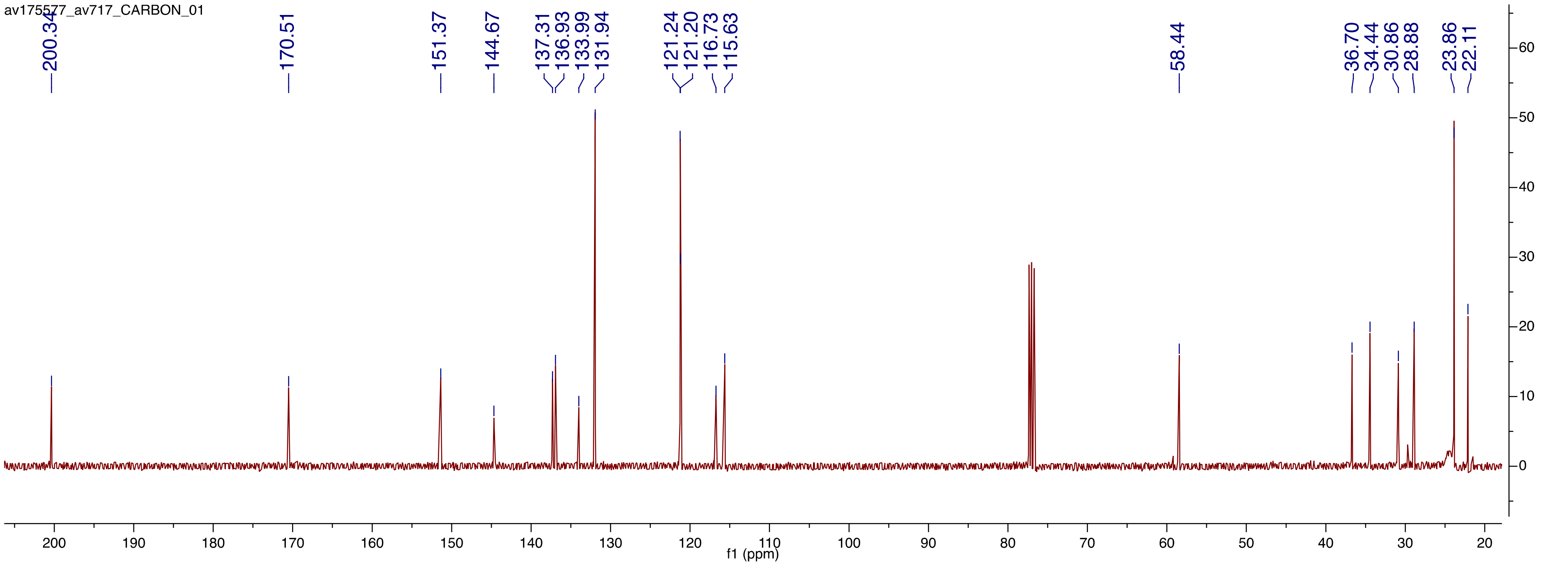

S-((2R)-1-((4-Bromophenyl)amino)-2-methyl-1-oxo-5-((tetrahydro-2H-pyran-2-yl)oxy)pentan-2-yl) 2,4,6-triisopropylbenzothioate 6ea

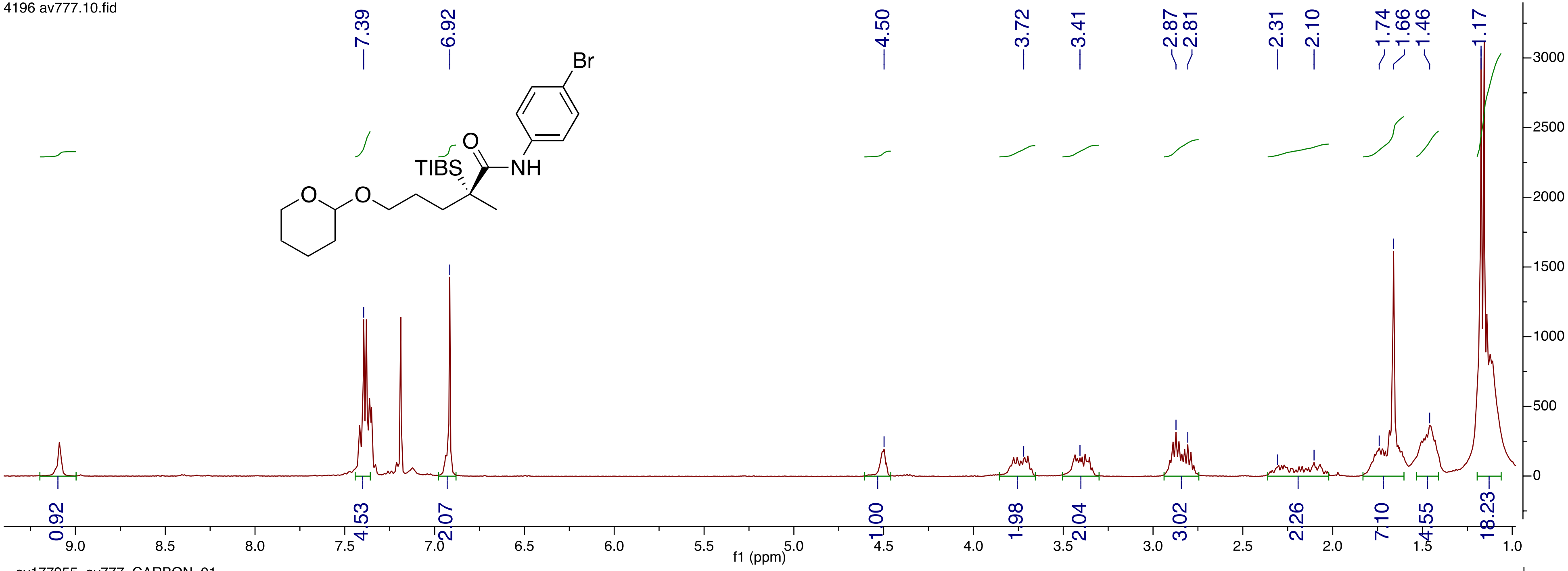

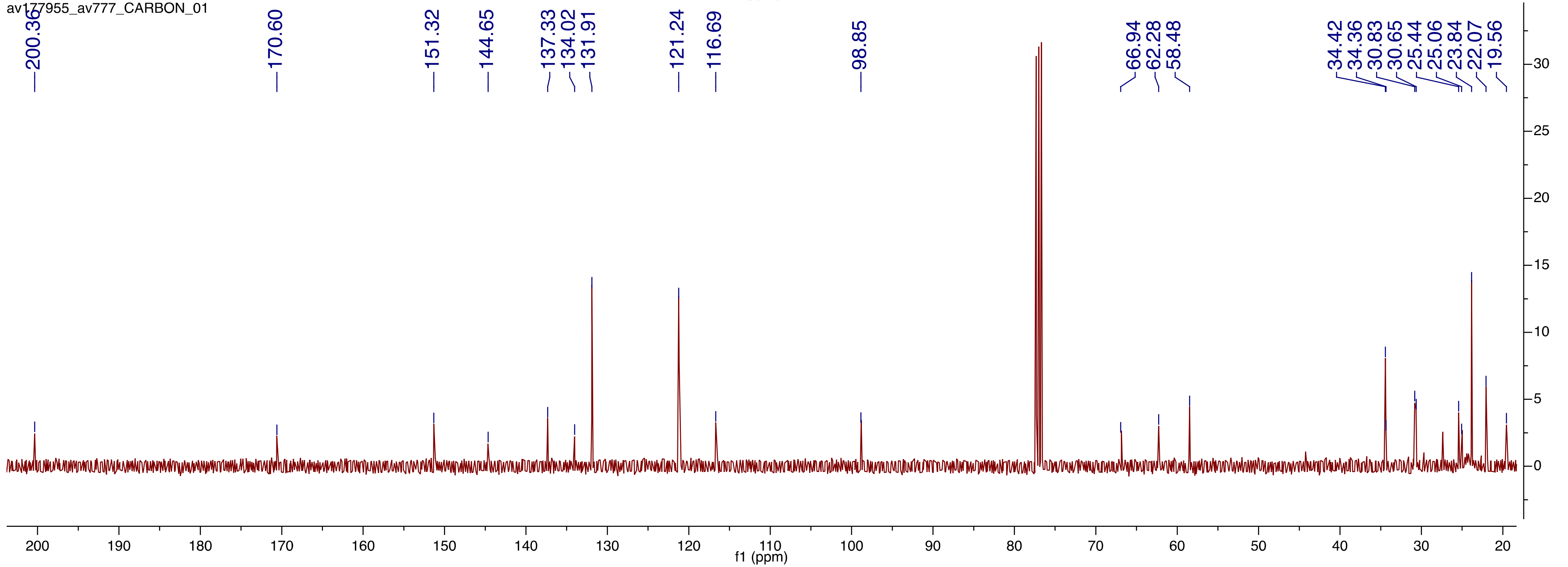

Methyl (3R,8S,9S,10R,13R,14S,17R)-10,13-dimethyl-17-((R)-6-methylheptan-2-yl)-3-((2,4,6-triisopropylbenzoyl)thio)-2,3,4,7,8,9,10,11,12,13,14,15,16,17-tetradecahydro-1H-cyclopenta[a]phenanthrene-3-carboxylate 9

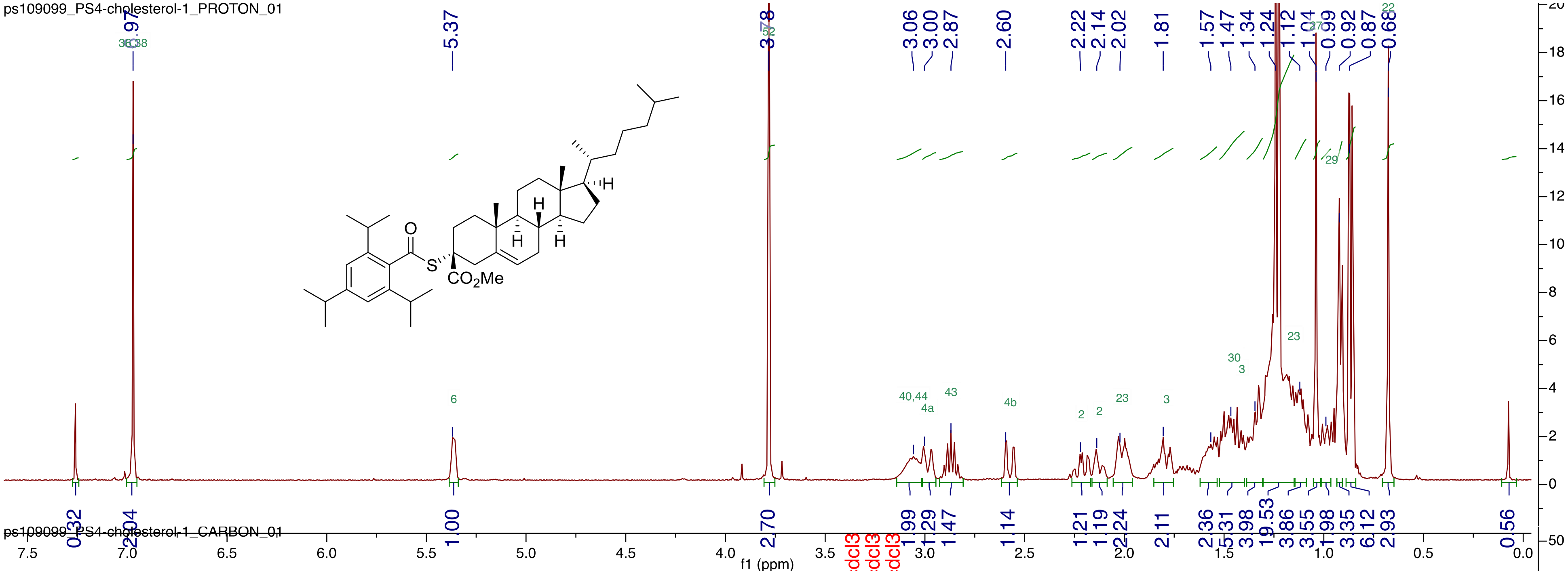

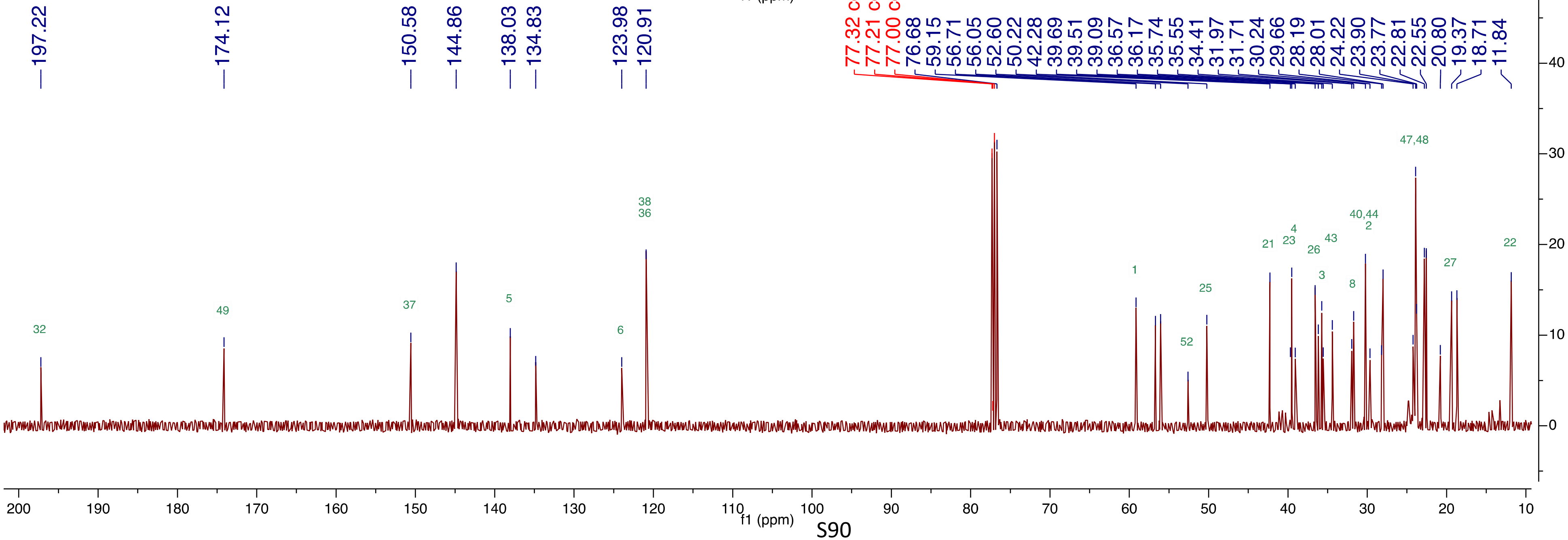

(1*R*,2*R*)-2-Mercapto-2-methyl-1,4-diphenylbutan-1-ol 7

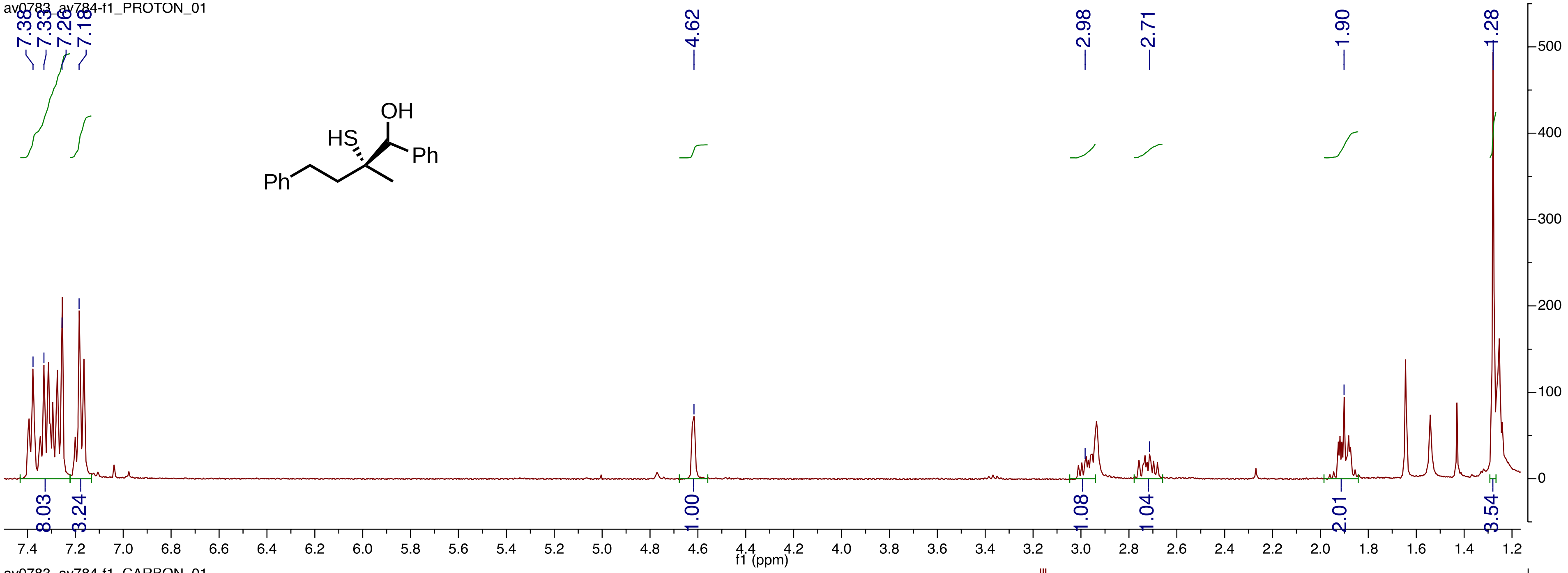

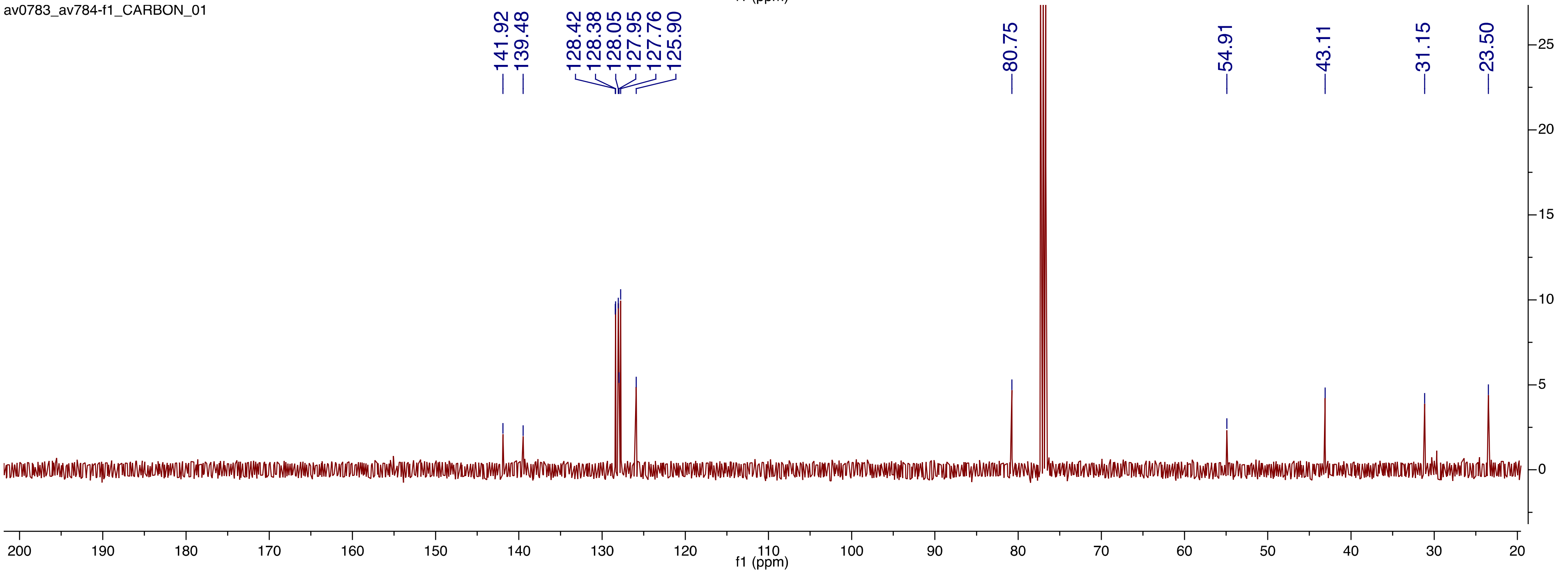

Supplement: Supplementary file 1 — Supplementary [file ANIE-56-10835-s001.pdf]
